# Ni-catalyzed migratory fluoro-alkenylation of unactivated alkyl bromides with *gem*-difluoroalkenes†
†Electronic supplementary information (ESI) available. See DOI: 10.1039/c8sc04162h


**DOI:** 10.1039/c8sc04162h

**Published:** 2018-11-09

**Authors:** Lu Zhou, Chuan Zhu, Peijia Bi, Chao Feng

**Affiliations:** a Institute of Advanced Synthesis , School of Chemistry and Molecular Engineering , Jiangsu National Synergetic Innovation Center for Advanced Materials , Nanjing Tech University , Nanjing 211816 , P. R. China . Email: iamcfeng@njtech.edu.cn

## Abstract

We describe a nickel-catalyzed highly regio- and stereoselective migratory fluoro-alkenylation of unactivated alkyl bromides. A unique catalytic cycle merging alkyl nickel chain-walking and defluorinative coupling enables the introduction of a broad array of fluoroalkenyl moieties into carbon chains. Control experiments with other halogenated alkenes demonstrated the essential role of fluorine atoms in this reaction. Notably, the reaction proceeds under mild conditions and allows for the synthesis of a variety of valuable monofluoroalkenes.

## Abstract 2

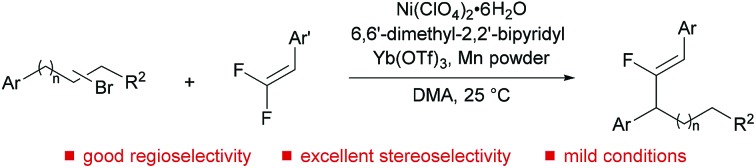
A migratory fluoro-alkenylation of unactivated alkyl bromides is reported; the reaction is enabled by fluorine effects and involves an alkyl nickel chain-walking mechanism.

## Introduction

**Scheme 1:**
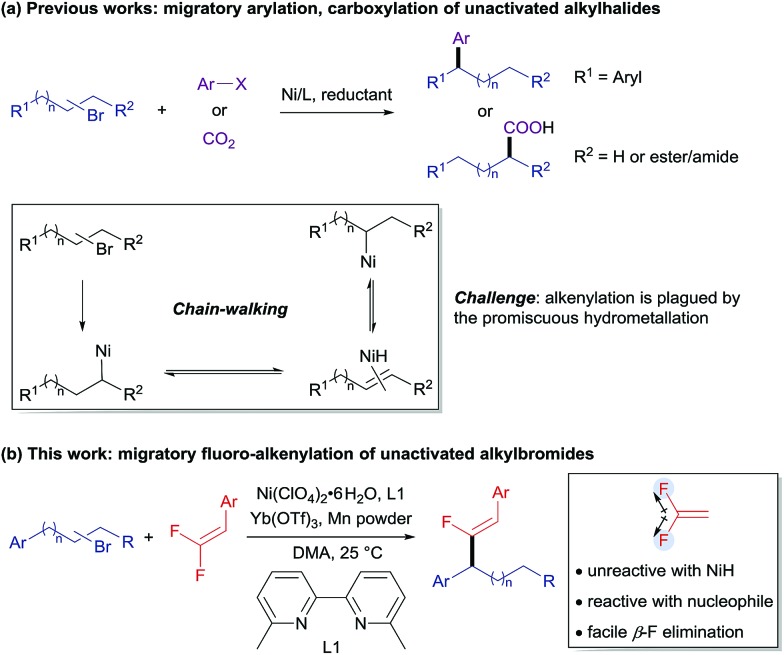
Migratory functionalization of unactivated alkyl halides.

Cross-electrophile coupling has found wide application in the construction of C–C bonds and serves as a powerful and reliable alternative to the classical nucleophile/electrophile procedures.^1^ In particular, C(sp^2^)–C(sp^3^) cross-coupling has been thoroughly explored using the combination of aryl/alkenyl and alkyl electrophiles, wherein β-hydride elimination is inhibited to minimize olefin by-products.^2^ Conversely, transition-metal-catalyzed remote functionalization exploits iterative β-hydride elimination and metal-H insertion, namely the “chain-walking” process, allowing introduction of functionality into an otherwise unreactive aliphatic position.^3^ Recently, significant progress in this field has been achieved by the groups of Martin,^4^ Zhu,^5^ and Yin.^6^ Remote arylation and carboxylation of unactivated alkyl halides have been well established (Scheme 1a). However, the reported reactions are still restricted to a relatively limited number of coupling partners, thus limiting the resulting molecular diversity of the products. A notable limitation remains: the corresponding alkenylation of unactivated alkyl halides has not been reported yet, although various alkenes are frequently found in pharmaceuticals or other functional molecules. The reason for the lack of a precedent probably lies in the potential addition of disassociated metal-H to the olefinic coupling component and even the alkene product during the chain-walking process.^5^,^7^ In this regard, the development of efficient and practical routes for metal-hydride-involving remote alkenylation has remained as a formidable challenge.

**Scheme 2:**
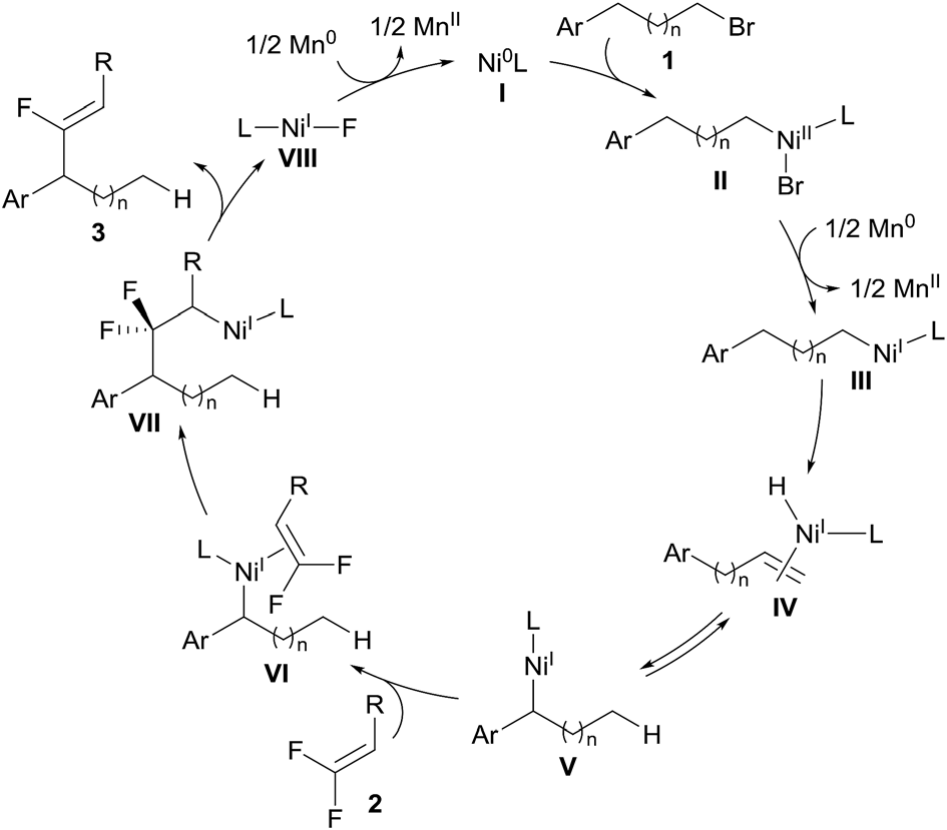
Reaction design.

To circumvent the problem of promiscuous hydrometallation, an alkenyl coupling partner inherently inert to metal-H would be required in a successful migratory alkenylation. Recently, *gem*-difluoroalkenes have been demonstrated by Cao, Toste, Fu, our group, and others as efficient fluoro-alkenylating reagents to access a wide range of monofluoroalkenes using radical or ionic manifolds.^8^ The strong electron-withdrawing nature of the two fluorine atoms renders *gem*-difluoroalkenes highly reactive toward nucleophilic attack on the fluorinated sp^2^-carbon, and fluoro-alkenylation could be accomplished through facile β-fluoride elimination. Furthermore, we considered that the fluorine-based polarization and p–π interaction would make the fluorinated olefins less favored in the hydrometallation compared with the non-fluorinated alkene intermediates and therefore amenable in the migratory alkenylation reactions.^9^ It could then be envisaged that *gem*-difluoroalkene **2** can coordinate to thermodynamically stable benzyl nickel **V** (generated through oxidative addition of the C–Br bond to the Ni^0^ and single-electron reduction followed by chain-walking) to form intermediate **VI**, which undergoes regioselective migratory insertion to produce a carbo-nickelation adduct (**VII**).^5^ Subsequently, β-fluoride elimination could lead to the formation of the monofluoroalkene **3**.^10^ Reduction of Ni–F (**VIII**) could then regenerate the catalytically active Ni^0^ species (**I**) (Scheme 2). However, we anticipated that a few challenges would have to be overcome to realize this strategy: (1) a nickel catalyst must be able to mediate all the elementary steps including chain-walking and defluorinative coupling; (2) the resulting monofluoroalkene product should be unreactive towards the Ni–H as well as Ni-alkyl species; (3) the migration of Ni–H along the carbon chain should be much faster than the alkenylation step to ensure good regioselectivity. In this report, we disclose such a convenient synthetic route for the migratory fluoro-alkenylation of unactivated alkyl bromides with high regio- and stereocontrol (Scheme 1b). Remarkably, by virtue of this operationally simple methodology a variety of structurally privileged monofluoroalkenes^11^ could be obtained from easily available materials.

## Results and discussion

To test our hypothesis, we selected *gem*-difluoroalkene **2a** as an alkenylating reagent to react with 1-bromo-2-phenylethane **1a** (Table 1). After careful evaluation of the reaction parameters, we found that a combination of inexpensive and bench-stable Ni(ClO_4_)_2_·6H_2_O as a pre-catalyst, 6,6′-dimethyl-2,2′-bipyridyl (**L1**) as a ligand, and Mn as a reducing agent to generate Ni^0^ in DMA at 25 °C gave the benzylic fluoro-alkenylation product **3a** with *Z*-configuration in 28% NMR yield within 12 hours (entry 1).^12^ Pleasingly, the product was obtained with excellent regioselectivity and stereoselectivity. Furthermore, it was found that introduction of MgCl_2_ as an additive^13^ gave an improved yield (44%), thus indicating that the Lewis acid has a positive effect on the reaction yield. Encouraged by this result, extensive screening of Lewis acid additives was conducted (Table S1†), and Yb(OTf)_3_ led to a favorable result (70% yield). The application of YbCl_3_ afforded **3a** in comparable yield (63%), reflecting the importance of the ytterbium cation (entry 4). On the other hand, changing the reducing agents to Zn, B_2_pin_2_ or HCOONa had a deleterious effect on the outcome of the reaction (Table S1†). Moreover, Ni(ClO_4_)_2_·6H_2_O proved to be the optimal catalyst after examination of various nickel salts (Table S1†). Subsequently, the ligand was further optimized. Increasing the steric profile of the substituent at the *ortho* position to the nitrogen in the bipyridyl scaffold (**L2**) hampered the reactivity of this alkenylation reaction, and no product was detected when the reaction was treated with bipyridyl (**L3**) as a ligand, suggesting the essential role of such substituents (entries 5 and 6). The structural analogue neocuproine (**L4**) also exhibited high catalytic efficiency while bathocuproine (**L5**) was less effective (entries 7 and 8). Slightly higher yields of **3a** were obtained when the reaction was treated with fewer equivalents of Mn (entry 9). Of particular note, the process is readily scaled up, the reaction of **1a** (2.5 mmol) with **2a** (1.0 mmol) gave **3a** in 70% yield (entry 10).

**Table 1:** Optimization of reaction conditions^*a*^

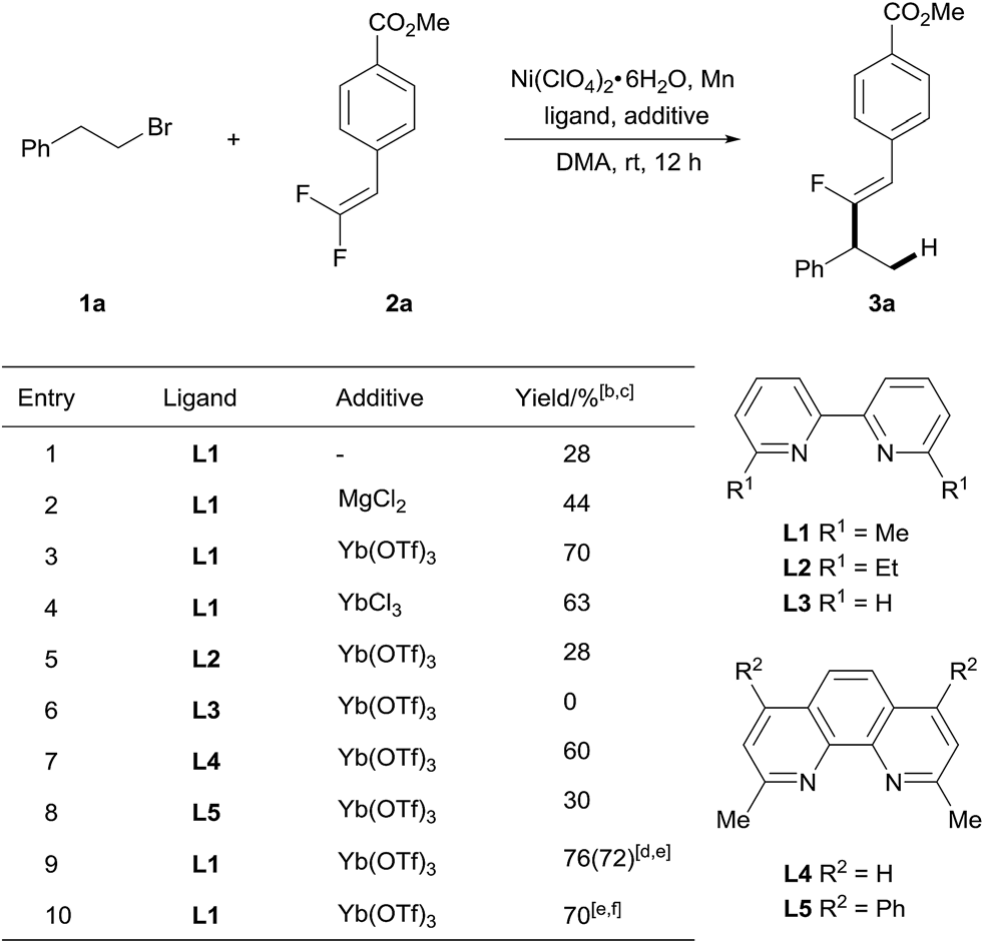

^*a*^Unless otherwise noted, reactions were carried out with 0.5 mmol of **1a**, 0.2 mmol of **2a**, 5 mol% of Ni(ClO_4_)_2_·6H_2_O, 6 mol% of ligand, 0.5 mmol of Mn powder and 0.1 mmol of additive in 1 mL of DMA at 25 °C for 12 h.

^*b*^Determined by ^1^H NMR *versus* an internal standard.

^*c*^The regioisomeric ratio was higher than 100 : 1 according to ^19^F NMR of the crude product.

^*d*^0.48 mmol of Mn powder was used.

^*e*^Isolated yield.

^*f*^1.0 mmol scale reaction.

**Scheme 3:**
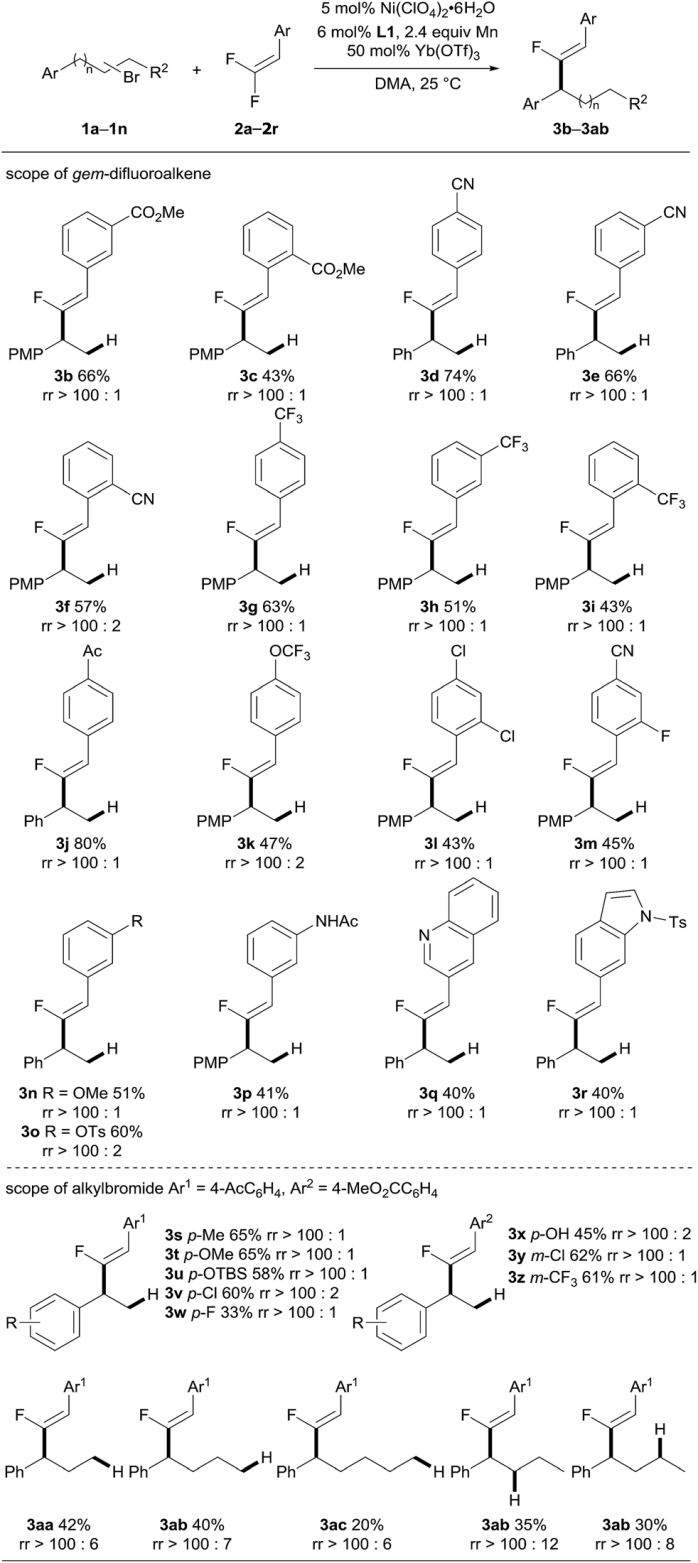
Substrate scope. See the ESI† for experimental details. Isolated yields are indicated. The regioisomeric ratio (rr, the ratio of the benzylic fluoro-alkenylation product to the other regioisomers) was determined by ^19^F NMR analysis of the crude product. PMP = *p*-methoxyphenyl. Ts = tosyl. TBS = *tert*-butyldimethylsilyl.

By using the optimized reaction conditions, the scope of this Ni-catalyzed migratory fluoro-alkenylation of unactivated alkyl bromide with *gem*-difluoroalkenes was evaluated. As shown in Scheme 3, the present protocol shows a remarkably broad scope with respect to the *gem*-difluoroalkene coupling partner. 1-Aryl-2-bromoethane was initially coupled with various aryl-*gem*-difluoroalkene derivatives to afford the fluoro-alkenylation products (**3a–r**). The method tolerated a variety of substitution patterns on the phenyl group. In general, the reaction displayed a noticeable preference for substrates bearing electron-withdrawing groups, such as cyano, trifluoromethyl, ester and ketone, which is in sharp contrast to radical-associated alkylation wherein electron-rich *gem*-difluoroalkenes were favored.8*i* For example, *para*-substituted phenyl-*gem*-difluoroalkenes were converted into the corresponding products (**3a**, **3d**, **3g**, **3j** and **3k**), mostly in moderate to good yields. The use of *meta*-substituted phenyl-*gem*-difluoroalkenes was also explored, which led to moderate yields of products (**3b**, **3e** and **3h**). Notably, sterically demanding starting materials that bear *ortho*-substituents on the phenyl ring were amenable under the optimized reaction conditions; however, the yields of the desired products were reduced (**3c**, **3f** and **3i**). Chlorine and fluorine substituents were also compatible in the present reaction (**3l** and **3m**). Moreover, aryl-*gem*-difluoroalkenes with electron-donating substitution, including OMe, OTs and even the protic amide NHAc, were employed, and these transformations took place smoothly, leading to the compounds **3n–3p** with moderate yields. Additionally, nitrogen-containing heterocycle derived substrates could also be converted by the catalytic system (**3q** and **3r**). We next aimed to extend the scope of alkyl bromides. A range of β-bromoethylarenes having Me, OMe, OTBS, Cl and CF_3_ groups on the phenyl ring were subjected to the alkenylation reaction with an acetyl or a methoxylcarbonyl phenyl-*gem*-difluoroalkene (**2a** and **2j**). The products **3s–3z** were obtained in useful yields with excellent regioselectivity and stereoselectivity. It is worth mentioning that an unprotected hydroxyl group was also tolerated, despite affording the product in decreased yield. To further expand the scope of the alkyl bromides, long range alkenylation was then examined. Alkenylation occurred favorably at the benzylic position, providing the corresponding fluoro-alkenylated propane, butane and even pentane derivatives (**3aa–ac**). The yields decreased progressively as the carbon chain increased which might be attributed to the increased bulkiness of the benzyl–nickel intermediate. The yields of other related regioisomers were only slightly increased according to ^19^F NMR analysis of the crude product. These results suggest that formation of the benzyl-Ni species is faster compared to the alkenylation step. Then the more challenging secondary alkyl bromide was subjected to an alkenylation reaction with **2j**, and proved to be competent in this reaction, providing the expected benzylic fluoro-alkenylation product with good regioselectivity. The relatively lower yield of **3aa** from the secondary alkyl bromide (35% and 30% from (2-bromobutyl)benzene and (3-bromobutyl)benzene, respectively) than the primary one (40% from (4-bromobutyl)benzene) may indicate that the oxidative addition of the alkyl bromide to nickel is sensitive to the steric bulkiness, thus attenuating the reaction efficiency.

**Scheme 4:**
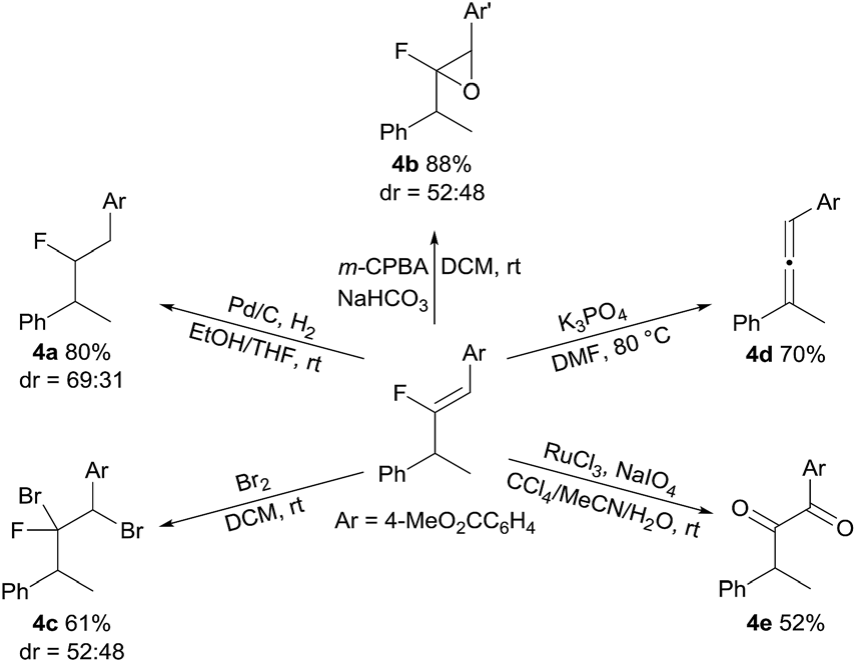
Synthetic applications.

The synthetic utility of the fluoro-alkenylation product was exemplified by further transformations of **3a** (Scheme 4). Hydrogenation of the fluoroalkene moiety contained within **3a** was carried out (H_2_, Pd/C), yielding **4a**. An epoxidation reaction using **3a** also proceeded well to give fluoroepoxide **4b** with high yield (88%). In addition, dibromination of the C=C double bond with bromine was executed and the addition product **4c** was formed uneventfully. Moreover, treatment of **3a** with a base to eliminate HF furnished the synthetically useful trisubstituted allene **4d** in 70% yield. This sequence allows *gem*-difluoroalkenes to serve as a vinylidene source, and thereby an expedient migratory vinylidenation was achieved. Finally, we found that **3a** could be converted into 1,2-diketone **4e** by oxidation.

**Scheme 5:**
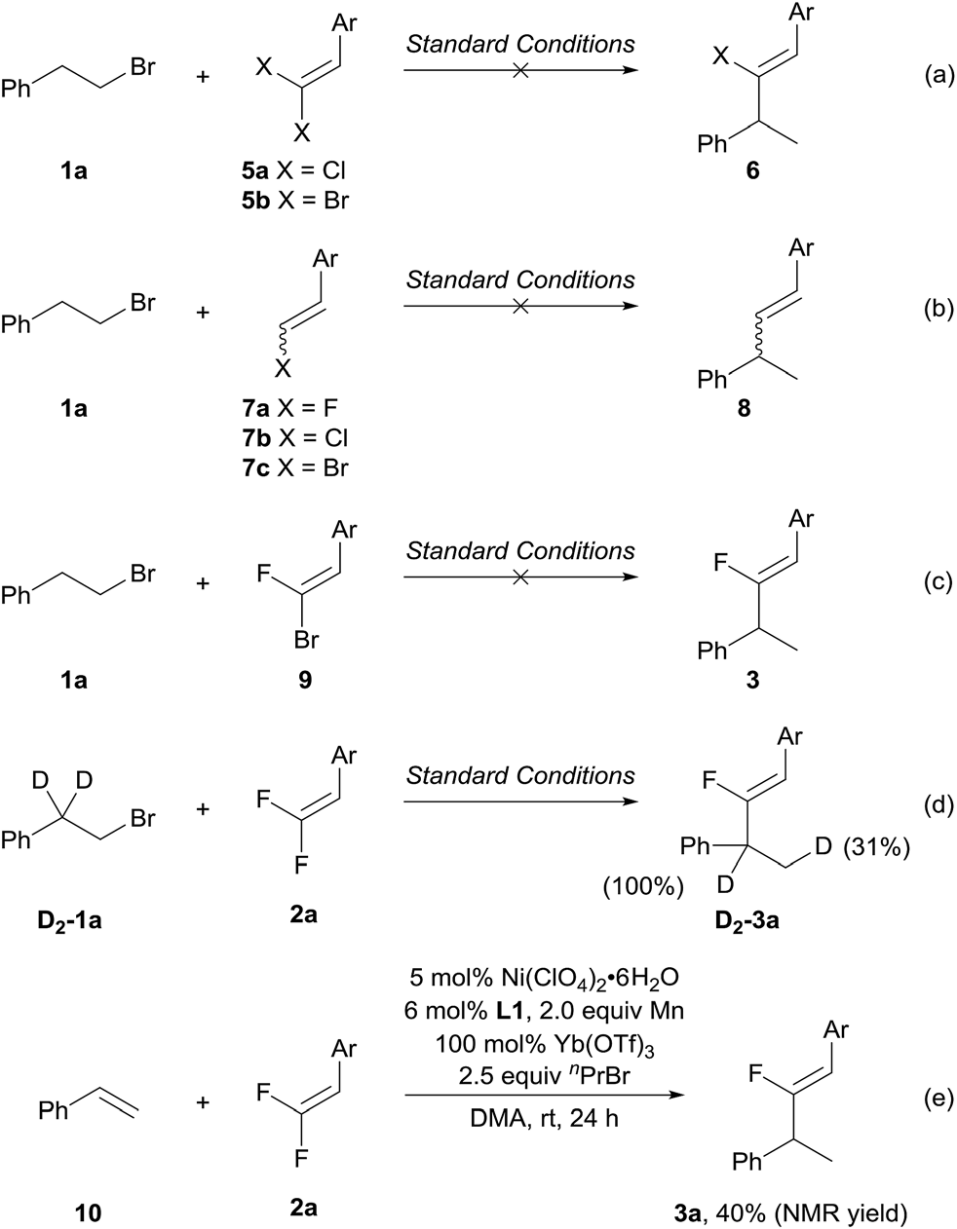
Control experiments (Ar = 4-MeOCOC_6_H_4_).

To gain preliminary insight into the unique fluorine effects^14^ that enable the migratory fluoro-alkenylation reaction, several control experiments were then performed. First, we carried out the alkenylation reaction with a series of halogenated alkenes (Scheme 5a–c). As shown in Scheme 5a, *gem*-chloroalkene **5a** and *gem*-dibromoalkene **5b** did not lead to the desired halo-alkenylation product. Second, we examined monohaloalkenes under the standard conditions. Not surprisingly, in these experiments no alkenylation product was detected again upon the consumption of the starting materials **7a–c** (Scheme 5b).^15^ The comparison with various halogenated alkenes highlights the prominent role of the two fluorine atoms which entail the unique reactivity in migratory fluoro-alkenylation. Regarding the C(sp^2^)–C(sp^3^) bond formation, an alternative pathway involving oxidative addition of the C(sp^2^)–F bond to the benzyl–Ni followed by reductive elimination is also possible.8c,e However, the 1-bromo-1-fluoroalkene **9** failed to produce the fluoro-alkenylation product which could tentatively rule out this mechanism (Scheme 5c).^16^ The use of deuterium labelled alkyl bromide **D_2_-1a** gave rise to the deuterium-shift product **D_2_-3a** exclusively, which strongly supports idea that a process involving β-hydride elimination and reinsertion is operative in the present transformation (Scheme 5d). Note that no significant further hydrogen/deuterium scrambling was found in **D_2_-3a**, revealing the thermodynamic preference of the benzylic-Ni intermediate in the migration process, which intrinsically dictates the regioselectivity of this transformation. Given the Ni-catalyzed chain-walking process does not require a Lewis acid additive,^4^–^6^ it is reasonable to attribute the role of Yb(OTf)_3_ in activating *gem*-difluoroalkene towards the nucleophilic addition^17^ or facilitating the reduction of Ni–F species.^18^ To obtain additional insight into the influence of Yb(OTf)_3_, reactions with a stoichiometric amount of Ni(ClO_4_)_2_·6H_2_O/**L1** were carried out (see the ESI† for details). In the reaction with Yb(OTf)_3_, the desired product (**3a**) was attained in 55% yield which is much higher than the ytterbium-free reaction (16%), implying that the Lewis acid should take effect in the nucleophilic addition step rather than the Ni–F reduction. To evaluate the ability of Ni–H to recognize the non-fluorinated alkenes and *gem*-difluoroalkenes, styrene **10** was subjected to the fluoro-alkenylation reaction with 1-bromopropane as the hydride source (Scheme 5e).^5^,^19^ With the same catalytic system, the desired product **3a** was formed in 40% NMR yield. This result clearly demonstrates the significant impact of fluorine substituents on the alkene moiety, which differentiate the two kinds of alkenes towards the Ni–H species as proposed in Scheme 1b.

## Conclusions

In conclusion, a general, Ni-catalyzed migratory fluoro-alkenylation of unactivated alkyl bromides with *gem*-difluoroalkenes has been developed, providing ready access to diversely functionalized monofluoroalkenes, which are valuable molecules in biological and materials science. More importantly, this work extends the boundaries of the highly attractive field of remote functionalization of unactivated alkyl electrophiles since it represents the first instance in which an alkene coupling partner has been used in a NiH-mediated process. It is also noteworthy that this C(sp^2^)–C(sp^3^) bond-forming reaction proceeds with excellent regio- and stereoselectivity under nonbasic conditions at room temperature, and an array of potentially reactive functional groups are tolerated.

## Conflicts of interest

There are no conflicts to declare.

## Supplementary Material

Supplementary information
