# Supplementary material for: Ni-catalyzed migratory fluoro-alkenylation of unactivated alkyl bromides with gem-difluoroalkenes
Source: Chem Sci. 2018 Nov 9;10(4):1144–9. doi: 10.1039/c8sc04162h (PMC6349022; doi:10.1039/c8sc04162h)

Electronic Supplementary Material (ESI) for Chemical Science.
This journal is © The Royal Society of Chemistry 2018

Supporting Information

Ni-Catalyzed Migratory Fluoro-Alkenylation of Unactivated Alkylbromides with *gem*-Difluoroalkenes

Lu Zhou,‡ Chuan Zhu,‡ Peijia Bi, and Chao Feng*

Institute of Advanced Synthesis (IAS), College of Chemistry and Molecular Engineering, Jiangsu National Synergetic Innovation Center for Advanced Materials (SICAM), Nanjing Tech University, 30 South Puzhu Road, Nanjing 211816, P. R. China

Table of Contents

1. **General information** ······ S2
2. **Procedure for the preparation of *gem*-difluoroalkenes** ······ S3
3. **Optimization of reaction conditions** ······ S4
4. **General procedure for Ni-catalyzed reaction and spectral data of products** ······ S5
5. **Synthetic applications** ······ S13
6. **Control experiments** ······ S16
7. **References** ······ S18
8. **NMR spectra** ······ S19
9. **Determination of regioisomeric ratio** ······ S77

1. General information

All operations were performed under a nitrogen atmosphere. ^{1}H, ^{13}C and ^{19}F-NMR spectra were recorded on a Bruker 400 (400 MHz for ^{1}H, 100 MHz for ^{13}C and 376 MHz for ^{19}F) or a JEOL ECX-400 (400 MHz for ^{1}H, 100 MHz for ^{13}C and 376 MHz for ^{19}F) spectrometer using residue solvent as internal reference. Silica gel (200~300 mesh) was used for flash column chromatography. High resolution mass analyses (ESI+) were performed on a Waters mass spectrometer.

Reagents: Unless otherwise noted, commercial reagents were used as received. Dehydrated DMA was purchased from Energy®. THF, toluene, acetonitrile and dichloromethane were purified by Vigor® solvent purification system. Alkylbromide **1a–1k**,[1-2] *gem*-difluoroalkenes **2a–2n**,[3] **2p**,[4] **2q**[3] and **5**,[5] **5a–5b**,[6-7] **7a–7c**,[8-10] **D$_2$-1a**[11] were prepared according to literature procedures.

1.1 Structure of alkylbromides **1a-m**

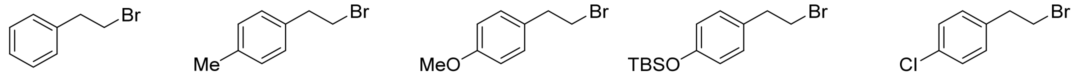

1a **1b** **1c** **1d** **1e**

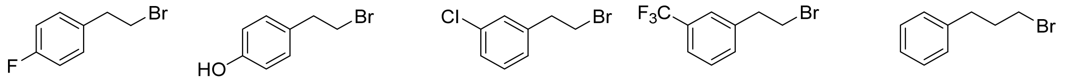

1f **1g** **1h** **1i** **1j**

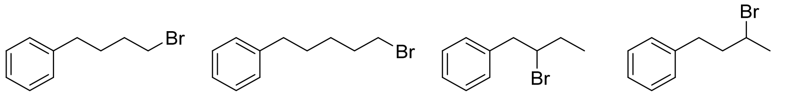

1k **1l** **1m** **1n**

1.2 Structure of *gem*-difluoroalkenes **2a-q**

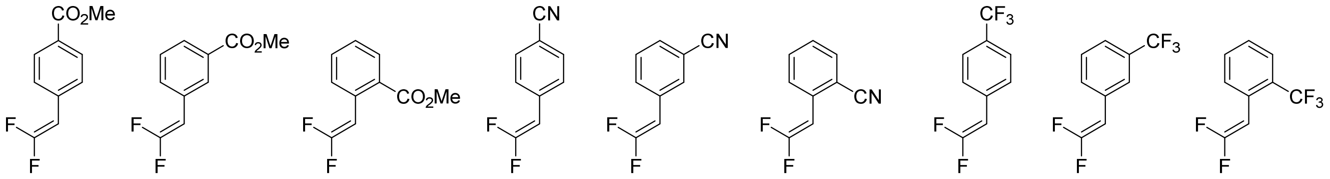

2a **2b** **2c** **2d** **2e** **2f** **2g** **2h** **2i**

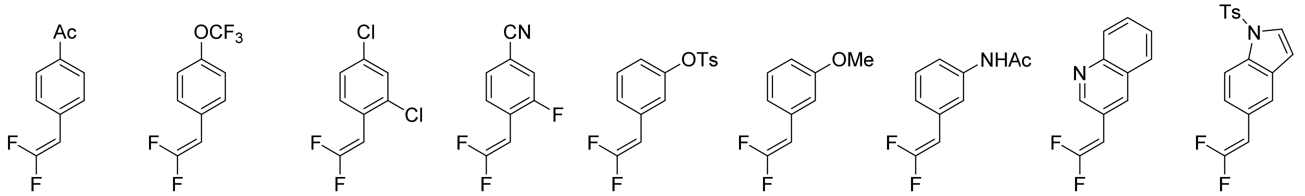

2j **2k** **2l** **2m** **2n** **2n** **2o** **2p** **2q**

1.3 Unsuccessful substrates

gem-difluoroalkenes

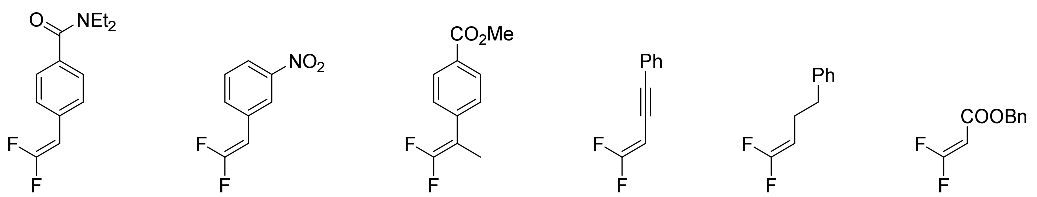

alkylbromides

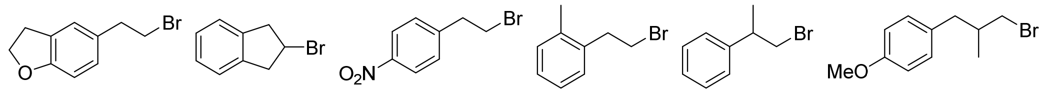

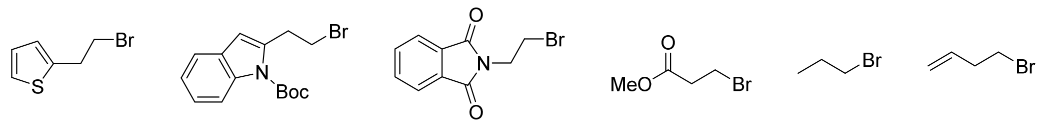

2. Procedure for the preparation of *gem*-difluoroalkenes

Preparation of *gem*-difluoroalkene **2o**

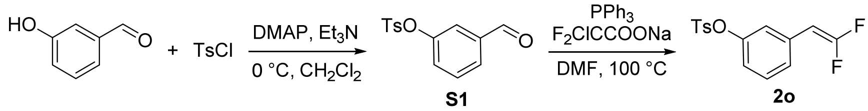

Step 1: To a stirred solution of 3-hydroxybenzaldehyde (1.2 g, 10 mmol, 1.0 equiv) in CH_2Cl_2 (50 mL) at 0 °C was added TsCl (4.2 g, 22 mmol, 1.1 equiv), DMAP (0.2 g, 2.0 mmol, 0.1 equiv). Then Et_3N (3.0 g, 30 mmol, 1.5 equiv) was added dropwise. At the end of the addition, the homogeneous solution was allowed to stir at room temperature for additional 2 h. Then the reaction mixture was quenched with 1M HCl and extracted with CH_2Cl_2. The combined organic layers were washed with brine and dried over Na_2SO_4. After solvent was removed under reduced pressure, the crude residue was purified by column chromatography on silica gel (petroleum ether/ethyl acetate = 5 : 1) to afford **S1** (4.4 g, 16.0 mmol) in 80% yield as white solid.

Step 2 : A solution of **S1** (2.7 g, 10 mmol, 1.0 equiv) and PPh_3 (5.2 g, 20 mmol, 2.0 equiv) in DMF (20 mL) was heated to 100 °C. To the reaction mixture at 100 °C was added $F_2ClCCOONa$ (3.0 g, 20 mmol, 2.0 equiv) slowly. After the reaction was completed according to the TLC (about 30 min), the reaction mixture was cooled to room temperature, quenched with water and extracted with ethyl acetate. The combined organic layers were washed with H_2O_2 (30 wt% in water, 10 mL), brine and dried over Na_2SO_4. After the solvent was removed under reduced pressure, the residual mixture was purified by column chromatography on silica gel (petroleum ether/ethyl acetate = 20 : 1) to afford compound **2o** (2.5 g, 8.1 mmol) in 81% yield as white solid. **^{1}H NMR (400 MHz, $CDCl_3$):** δ = 7.72 (d, *J* = 8.3 Hz, 2H), 7.32 (d, *J* = 8.1 Hz, 2H), 7.28-7.22 (m, 1H), 7.21-7.17 (m, 1H), 6.94 (t, *J* = 2.0 Hz, 1H), 6.86 (dt, *J* = 7.9, 1.3 Hz, 1H), 5.20 (dd, *J* = 25.6, 3.5 Hz, 1H), 2.45 (s, 3H). **^{19}F NMR (376 MHz, $CDCl_3$):** δ = -80.62 (t, *J* = 26.5 Hz), -82.45 (dd, *J* = 27.1, 3.4 Hz). **^{13}C NMR (100 MHz, $CDCl_3$):** δ = 156.4 (dd, *J* = 299.3, 289.8 Hz), 149.8, 145.5, 132.2, 132.1 (dd, *J* = 7.2, 6.1 Hz), 129.7 (d, *J* = 1.4 Hz), 128.5, 126.2 (dd, *J* = 6.3, 3.6 Hz), 121.4 (dd, *J* = 7.0, 3.4 Hz), 120.9 (t, *J* = 1.9 Hz), 81.5 (dd, *J* = 30.2, 13.2 Hz), 21.7; **HRMS (ESI, m/z):** calcd. for $C_{15}H_{12}F_2O_3SNa$ $[M+H]^+$: 333.0373, found: 333.0366.

Preparation of *gem*-difluoroalkene **2r**

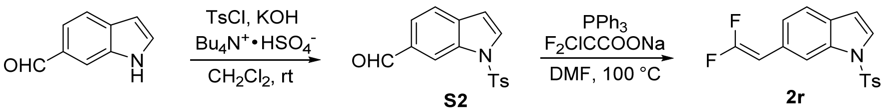

Step1: After a suspension of 1*H*-indole-6-carbaldehyde (1.5 g, 10 mmol, 1.0 equiv), TBAHS (0.5 g, 1.5 mmol, 0.15 equiv) and KOH (1.1 g, 20 mmol, 2.0 equiv) was stirred for 20 min. TsCl (2.9 g, 15 mmol, 1.5 equiv) was added and the mixture was stirred at room temperature for 12 h. The reaction mixture was quenched with H_2O and extracted with CH_2Cl_2. The organic layer was dried over anhydrous Na_2SO_4, filtered and concentrated. The product was purified by column chromatograph on silica gel (eluent = petroleum ether/ethyl acetate = 5 : 1) to afford **S2** (2.5 g, 8.3 mmol) in 83% yield as white solid.

Step 2: A solution of **S2** (1.5 g, 5.0 mmol, 1.0 equiv) and PPh_3 (2.6 g, 10 mmol, 2.0 equiv) in DMF (20 mL) was heated to 100 °C. To the reaction mixture at 100 °C was added $F_2ClCCOONa$ (1.5 g, 10 mmol, 2.0 equiv) slowly. After the reaction finished according to the TLC (about 30 min), the reaction mixture was cooled to room temperature, quenched with water and extracted with ethyl acetate. The combined organic layers were washed with H_2O_2 (30 wt% in water, 20 mL), brine and dried over Na_2SO_4. After solvent was removed under reduced pressure, the residual mixture was purified by column chromatography on silica gel (eluent = petroleum ether/ethyl acetate = 20 : 1) to afford **2r** (1.2 g, 3.5 mmol) in 70% yield as white solid. **^{1}H NMR (400 MHz, $CDCl_3$):** δ = 7.95 (s, 1H), 7.76 (d, *J* = 8.4 Hz, 2H), 7.56 (d, *J* = 3.7 Hz, 1H), 7.47 (d, *J* = 8.2 Hz, 1H), 7.23

(d, J = 8.2 Hz, 2H), 7.20 (d, J = 8.3 Hz, 1H), 6.62 (dd, J = 3.7, 0.9 Hz, 1H), 5.40 (dd, J = 25.9, 3.9 Hz, 1H), 2.35 (s, 3H). **^{19}F NMR (376 MHz, $CDCl_3$):** δ = -82.56 (dd, J = 32.2, 25.8 Hz), -84.29 (dd, J = 32.1, 4.0 Hz). **^{13}C NMR (100 MHz, $CDCl_3$):** δ = 156.1 (dd, J = 297.9, 288.0 Hz), 145.0, 135.0, 135.0, 129.9, 129.6 (t, J = 1.7 Hz), 126.8 (t, J = 3.1 Hz), 126.78 , 123.0 (dd, J = 6.3, 3.1 Hz), 121.4, 112.4 (dd, J = 7.0, 3.9 Hz), 108.8, 82.6 (dd, J = 29.6, 13.3 Hz), 21.5. **HRMS (ESI, m/z):** calcd. for $C_{17}H_{14}F_2NO_2S$ $[M+H]^+$: 334.0713, found: 334.0717.

Preparation of compound **7b**[8]

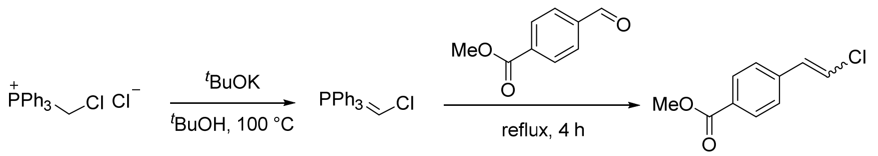

S3 **7b**

To *tert*-butyl alcohol (20 mL) was added *t*-BuOK (0.7 g, 6.0 mmol, 1.8 equiv) and the mixture was heated at reflux for 1 h to afford potassium *t*-butoxide solution. Then (chloromethyl)triphenylphosphonium chloride (1.7 g, 5.0 mmol, 1.5 equiv) was added and the resulting mixture was sitrred for 90 min to afford the phosphonium ylide (**S3**). To the solution of **S3** was added methyl 4-formylbenzoate (0.54 g, 3.3 mmol, 1.0 equiv) and the mixture was stirred for another 4 h at reflux. Upon the completion of the reaction, the reaction mixture was quenched by adding water and extracted with ethyl acetate. The combined organic layers were washed with brine and dried over Na_2SO_4. After solvent was removed under reduced pressure, the residual mixture was purified by column chromatography on silica gel (petroleum ether/ethyl acetate = 20 : 1) to afford **7b** (0.4 g, 2.0 mmol, E/Z = 58/42) in 60% yield as white solid. **^{1}H NMR (400 MHz, $CDCl_3$)**: δ = 7.95 (s, 1H), 7.76 (d, J = 8.4 Hz, 2H), 7.56 (d, J = 3.7 Hz, 1H), 7.47 (d, J = 8.2 Hz, 1H), 7.23 (d, J = 8.2 Hz, 2H), 7.20 (d, J = 8.3 Hz, 1H), 6.62 (dd, J = 3.7, 0.9 Hz, 1H), 5.40 (dd, J = 25.9, 3.9 Hz, 1H), 2.35 (s, 3H).

3. Optimization of reaction conditions

Table S1. Optimization of reaction conditions.[a]

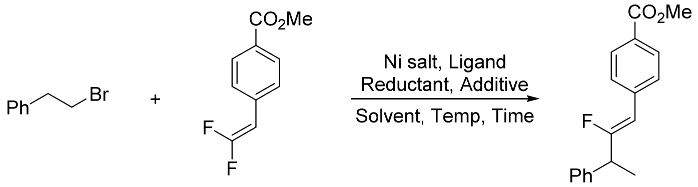

1a **2a** **3a**

Entry	Ni salt	Ligand	Reductant	Additive	Solvent	Temp./°C	Time/h	Yield/%	rr
1	$Ni(ClO_4)_2·6H_2O$	**L1**	Mn	-	DMA	25	12	28	100 : 1
2	$Ni(ClO_4)_2·6H_2O$	**L1**	Mn	TMSCl	DMA	25	12	11	100 : 19
3	$Ni(ClO_4)_2·6H_2O$	**L1**	Mn	CF_3COOH	DMA	25	12	32	100 : 2
4	$Ni(ClO_4)_2·6H_2O$	**L1**	Mn	TfOH	DMA	25	12	14	100 : 1
5	$Ni(ClO_4)_2·6H_2O$	**L1**	Mn	$B(C_6F_5)_3$	DMA	25	12	26	100 : 1
6	$Ni(ClO_4)_2·6H_2O$	**L1**	Mn	$MgCl_2$	DMA	25	12	44	100 : 3
7	$Ni(ClO_4)_2·6H_2O$	**L1**	Mn	$Zn(OTf)_2$	DMA	25	12	6	100 : 2
8	$Ni(ClO_4)_2·6H_2O$	**L1**	Mn	$In(OTf)_3$	DMA	25	12	10	100 : 1
9	$Ni(ClO_4)_2·6H_2O$	**L1**	Mn	TBAB	DMA	25	12	23	100 : 2
10	$Ni(ClO_4)_2·6H_2O$	**L1**	Mn	$Cu(OTf)_2$	DMA	25	12	30	100 : 2
11	$Ni(ClO_4)_2·6H_2O$	**L1**	Mn	ZrF_4	DMA	25	12	40	100 : 1
12	$Ni(ClO_4)_2·6H_2O$	**L1**	Mn	TBAI	DMA	25	12	24	100 : 12

13	$Ni(ClO_4)_2 \cdot 6H_2O$	**L1**	Mn	$YbCl_3$	DMA	25	12	63	100 : 1
13	$Ni(ClO_4)_2 \cdot 6H_2O$	**L1**	Mn	$Yb(OTf)_3$	DMA	25	12	70	100 : 1
14[b]	$Ni(ClO_4)_2 \cdot 6H_2O$	**L1**	Mn	$Yb(OTf)_3$	DMA	25	12	31	100 : 1
15[c]	$Ni(ClO_4)_2 \cdot 6H_2O$	**L1**	Mn	$Yb(OTf)_3$	DMA	25	12	48	100 : 1
16[d]	$Ni(ClO_4)_2 \cdot 6H_2O$	**L1**	Mn	$Yb(OTf)_3$	DMA	25	12	63	100 : 1
17	$NiCl_2$	**L1**	Mn	$Yb(OTf)_3$	DMA	25	12	10	100 : 2
18	$NiBr_2$	**L1**	Mn	$Yb(OTf)_3$	DMA	25	12	24	100 : 2
19	$Ni(OTf)_2$	**L1**	Mn	$Yb(OTf)_3$	DMA	25	12	6	100 : 1
20	$Ni(OAc)_2 \cdot 4H_2O$	**L1**	Mn	$Yb(OTf)_3$	DMA	25	12	42	100 : 2
21	$Ni(COD)_2$	**L1**	Mn	$Yb(OTf)_3$	DMA	25	12	0	-
22	$Ni(ClO_4)_2 \cdot 6H_2O$	**L2**	Mn	$Yb(OTf)_3$	DMA	25	12	28	100 : 2
23	$Ni(ClO_4)_2 \cdot 6H_2O$	**L3**	Mn	$Yb(OTf)_3$	DMA	25	12	0	-
24	$Ni(ClO_4)_2 \cdot 6H_2O$	**L4**	Mn	$Yb(OTf)_3$	DMA	25	12	60	100 : 2
25	$Ni(ClO_4)_2 \cdot 6H_2O$	**L5**	Mn	$Yb(OTf)_3$	DMA	25	12	30	100 : 2
26	$Ni(ClO_4)_2 \cdot 6H_2O$	**L6**	Mn	$Yb(OTf)_3$	DMA	25	12	5	100 : 11
27	$Ni(ClO_4)_2 \cdot 6H_2O$	**L7**	Mn	$Yb(OTf)_3$	DMA	25	12	0	-
28	$Ni(ClO_4)_2 \cdot 6H_2O$	**L8**	Mn	$Yb(OTf)_3$	DMA	25	12	0	-
29	$Ni(ClO_4)_2 \cdot 6H_2O$	**L1**	Zn	$Yb(OTf)_3$	DMA	25	12	5	20 : 30
30	$Ni(ClO_4)_2 \cdot 6H_2O$	**L1**	HCOONa	$Yb(OTf)_3$	DMA	25	12	0	-
31	$Ni(ClO_4)_2 \cdot 6H_2O$	**L1**	B_2Pin_2/K_3PO_4	$Yb(OTf)_3$	DMA	25	12	4	100 : 4
32	$Ni(ClO_4)_2 \cdot 6H_2O$	**L1**	Mn	$Yb(OTf)_3$	DMA	25	12	70	100 : 1
33	$Ni(ClO_4)_2 \cdot 6H_2O$	**L1**	Mn	$Yb(OTf)_3$	DMF	25	12	6	100 : 8
34	$Ni(ClO_4)_2 \cdot 6H_2O$	**L1**	Mn	$Yb(OTf)_3$	THF	25	12	0	-
35	$Ni(ClO_4)_2 \cdot 6H_2O$	**L1**	Mn	$Yb(OTf)_3$	DMA	40	12	26	100 : 3
36	$Ni(ClO_4)_2 \cdot 6H_2O$	**L1**	Mn	$Yb(OTf)_3$	DMA	0	12	0	-
37	$Ni(ClO_4)_2 \cdot 6H_2O$	**L1**	Mn	$Yb(OTf)_3$	DMA	25	6	63	100 : 1
38	$Ni(ClO_4)_2 \cdot 6H_2O$	**L1**	Mn	$Yb(OTf)_3$	DMA	25	9	63	100 : 1
39	$Ni(ClO_4)_2 \cdot 6H_2O$	**L1**	Mn	$Yb(OTf)_3$	DMA	25	24	45	100 : 1
40[e]	$Ni(ClO_4)_2 \cdot 6H_2O$	**L1**	Mn	$Yb(OTf)_3$	DMA	25	12	76	100 : 1
41[f]	$Ni(ClO_4)_2 \cdot 6H_2O$	**L1**	Mn	$Yb(OTf)_3$	DMA	25	12	66	100 : 1

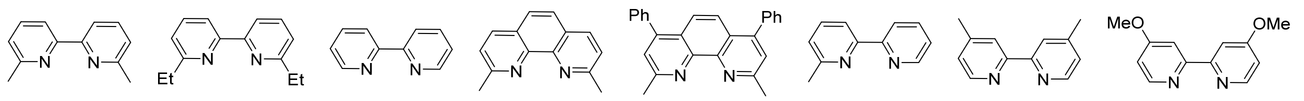

L1 **L2** **L3** **L4** **L5** **L6** **L7** **L8**

[a] Unless otherwise noted, the reaction was carried out with **1a** (0.2 mmol), **2a** (0.5 mmol), Ni salt (0.01 mmol), ligand (0.012 mmol), additive (50 mmol %) and solvent (1 mL) under N_2 at rt. Yield was determined by ^1H NMR using CH_2Br_2 as an internal standard. [b] 10 mol % of $Yb(OTf)_3$ was used. [c] 25 mol % of $Yb(OTf)_3$ was used. [d] 75 mol % of $Yb(OTf)_3$ was used. [e] 2.4 equiv of Mn was used. [f] 2.2 equiv of Mn was used. rr refers to regioisomeric ratio, represents the ratio of the benzylic fluoro-alkenylation product to the other isomer as determined by ^{19}F NMR analysis of the crude product.

4. General procedure for Ni-catalyzed reaction and spectral data of products

To an oven-dried schlenk tube equipped with a magnetic stir bar was added $Ni(ClO_4)_2 \cdot 6H_2O$ (3.7 mg, 0.01 mmol, 5.0 mol%), **L1** (2.2 mg, 0.012 mmol, 6.0 mol%) and $Yb(OTf)_3$ (62 mg, 0.1 mmol, 50 mol% for **3a-3z**; 124 mg, 0.2 mmol, 100 mol% for **3aa-3ac**). The Schlenk tube was evacuated and filled with nitrogen for three times. To these solids, DMA (1.0 mL) was added

under N_2 atmosphere. After stirring at room temperature for 10 min, the alkyl bromide **1** (0.50 mmol, 2.5 equiv), the *gem*-difluoroalkene **2** (0.20 mmol, 1.0 equiv) and Mn powder (26 mg, 0.48 mmol, 2.4 equiv) were added under nitrogen and stirred at room temperature for 12 to 20 h. The reaction mixture was diluted with ethyl acetate (10 mL) and filtered through a short pad of silica gel. The filtrate was washed with water (10 mL × 2), brine (10 mL) and dried over Na_2SO_4. After solvent was removed under reduced pressure, the crude residue was purified by column chromatography or preparative TLC on silica gel (petroleum ether/ethyl acetate = 50 : 1) to afford the desired product.

methyl (*Z*)-4-(2-fluoro-3-phenylbut-1-en-1-yl)benzoate (3a)

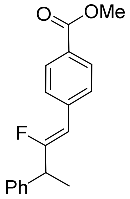

3a

Following general procedure, the reaction mixture was stirred for 12 h and **3a** was obtained as white solid (36 mg, 0.13 mmol, 72%, rr > 100:1). **^{1}H NMR (400 MHz, $CDCl_3$):** δ = 7.89 (d, *J* = 8.5 Hz, 1H), 7.45 (d, *J* = 8.5 Hz, 1H), 7.32 – 7.15 (m, 3H), 5.55 (d, *J* = 38.9 Hz, 1H), 3.82 (s, 3H), 3.69 (dq, *J* = 14.5, 7.2 Hz, 1H), 1.48 (d, *J* = 7.1 Hz, 3H). **^{19}F NMR (376 MHz, $CDCl_3$):** δ = -99.70 (dd, *J* = 38.9, 15.6 Hz). **^{13}C NMR (100 MHz, $CDCl_3$):** δ = 166.8, 164.9 (d, *J* = 272.6 Hz), 141.5, 138.2 (d, *J* = 2.7 Hz), 129.7, 128.7, 128.3 (d, *J* = 7.9 Hz), 128.1 (d, *J* = 2.4 Hz), 127.5, 127.1, 105.1 (d, *J* = 8.3 Hz), 52.0, 43.4 (d, *J* = 25.6 Hz), 18.7 (d, *J* = 4.2 Hz); **HRMS (ESI, m/z):** calcd. for $C_{18}H_{17}FO_2$ $[M+H]^+$: 285.1291, found: 285.1297.

methyl (*Z*)-3-(2-fluoro-3-(4-methoxyphenyl)but-1-en-1-yl)benzoate (3b)

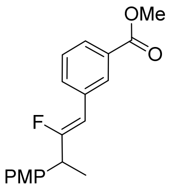

3b

Following general procedure, the reaction mixture was stirred for 16 h and **3b** was obtained as colourless liquid (41.6 mg, 0.13 mmol, 66%, rr > 100:1). **^{1}H NMR (400 MHz, $CDCl_3$):** δ = 8.01 (t, *J* = 1.8 Hz, 1H), 7.79 (dt, *J* = 7.9, 1.4 Hz, 1H), 7.61 (dt, *J* = 7.9, 1.5 Hz, 1H), 7.29 (t, *J* = 7.8 Hz, 1H), 7.21 – 7.14 (m, 2H), 6.84 – 6.78 (m, 2H), 5.51 (d, *J* = 38.9 Hz, 1H), 3.82 (s, 3H), 3.72 (s, 3H), 3.63 (dq, *J* = 14.5, 7.2 Hz, 1H), 1.45 (d, *J* = 7.2 Hz, 3H). **^{19}F NMR (376 MHz, $CDCl_3$):** δ = -102.36 (dd, *J* = 39.0, 15.3 Hz). **^{13}C NMR (100 MHz, $CDCl_3$):** δ = 167.0, 164.4 (d, *J* = 270.2 Hz), 158.6, 133.9 (d, *J* = 2.3 Hz), 133.6, 132.6 (d, *J* = 8.4 Hz), 130.2, 129.5 (d, *J* = 6.8 Hz), 128.4(overlap), 127.8 (d, *J* = 2.1 Hz), 114.0, 104.6 (d, *J* = 8.6 Hz), 55.2, 52.1, 42.4 (d, *J* = 25.9 Hz), 18.7 (d, *J* = 4.0 Hz); **HRMS (ESI, m/z):** calcd. for $C_{19}H_{20}FO_3$ $[M+H]^+$: 315.1396, found: 315.1402.

methyl (*Z*)-2-(2-fluoro-3-(4-methoxyphenyl)but-1-en-1-yl)benzoate (3c)

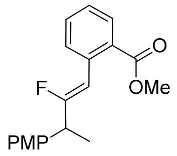

3c

Following general procedure, the reaction mixture was stirred 17 h and **3c** was obtained as colourless liquid (27.1 mg, 0.09 mmol, 43%, rr > 100:1). **^{1}H NMR (400 MHz, $CDCl_3$):** δ = 7.83 (d, *J* = 7.9 Hz, 1H), 7.63 (d, *J* = 7.9 Hz, 1H), 7.37 (td, *J* = 7.7, 1.5 Hz, 1H), 7.27 – 7.14 (m, 3H), 6.91 – 6.76 (m, 2H), 6.38 (d, *J* = 38.6 Hz, 1H), 3.78 (s, 3H), 3.72 (s, 3H), 3.65 (dq, *J* = 14.7, 7.5 Hz, 1H), 1.46 (d, *J* = 7.4 Hz, 3H). **^{19}F NMR (376 MHz, $CDCl_3$):** δ = -106.39 (dd, *J* = 38.8, 15.0 Hz). **^{13}C NMR (100 MHz, $CDCl_3$):**δ = 167.9, 163.4 (d, *J* = 268.6 Hz), 158.5, 134.3, 134.1, 131.8, 130.7 (d, *J* = 9.8 Hz), 130.5, 128.5, 126.6, 114.0, 103.4 (d, *J* = 7.0 Hz), 55.3, 52.0, 42.5 (d, *J* = 26.3 Hz), 19.0 (d, *J* = 4.3 Hz); **HRMS (ESI, m/z):** calcd. for $C_{19}H_{20}FO_3$ $[M+H]^+$: 315.1396, found: 315.1400.

(*Z*)-4-(2-fluoro-3-phenylbut-1-en-1-yl)benzonitrile (3d)

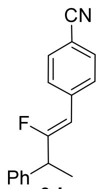

3d

Following general procedure, the reaction mixture was stirred for 12 h and **3d** was obtained as white solid (44.1 mg, 0.15 mmol, 74%, rr > 100:1). **^{1}H NMR (400 MHz, $CDCl_3$):** δ = 7.59 – 7.51 (m, 4H), 7.40 – 7.24 (m, 5H), 5.61 (d, *J* = 38.2 Hz, 1H), 3.77 (dq, *J* = 14.6, 7.2 Hz, 1H), 1.56 (d, *J* = 7.2 Hz, 3H). **^{19}F NMR (376 MHz, $CDCl_3$):** δ = -98.09 (dd, *J* = 38.2, 15.7 Hz). **^{13}C NMR (100 MHz, $CDCl_3$):** δ = 165.9 (d, *J* = 274.1 Hz), 141.1, 138.2 (d, *J* = 2.6 Hz), 132.1, 128.8 (d, *J* = 8.2 Hz), 128.7, 127.4, 127.2, 119.0, 110.0 (d, *J* = 2.9 Hz), 104.6 (d, *J* = 8.1 Hz), 43.4 (d, *J* = 25.3 Hz), 18.6 (d, *J* = 4.2 Hz). **HRMS (ESI, m/z):** calcd. for $C_{17}H_{15}FN$ $[M+H]^+$: 252.1189, found: 252.1186.

(*Z*)-3-(2-fluoro-3-phenylbut-1-en-1-yl)benzonitrile (3e)

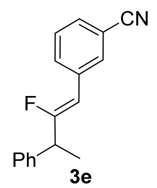

3e

Following general procedure, the reaction mixture was stirred for 12 h and **3e** was obtained as colourless liquid (32.8 mg, 0.13 mmol, 66%, rr > 100:1). **^{1}H NMR (400 MHz, CDCl$_3$):** δ = 7.77 (s, 1H), 7.66 (d, *J* = 7.9 Hz, 1H), 7.47 (d, *J* = 7.7 Hz, 1H), 7.42 – 7.24 (m, 6H), 5.57 (d, *J* = 38.1 Hz, 1H), 3.76 (dq, *J* = 14.6, 7.1 Hz, 1H), 1.55 (d, *J* = 7.2 Hz, 3H). **^{19}F NMR (376 MHz, CDCl$_3$):** δ = -100.16 (dd, *J* = 38.1, 15.6 Hz). **^{13}C NMR (100 MHz, CDCl$_3$):** δ = 165.2 (d, *J* = 272.1 Hz), 141.3, 134.8 (d, *J* = 2.2 Hz), 132.6 (d, *J* = 7.6 Hz), 131.8 (d, *J* = 8.4 Hz), 130.2 (d, *J* = 2.2 Hz), 129.2, 128.7, 127.5, 127.2, 118.8, 112.6, 104.0 (d, *J* = 8.4 Hz), 43.3 (d, *J* = 25.4 Hz), 18.6 (d, *J* = 4.2 Hz). **HRMS (ESI, m/z):** calcd. for $C_{17}H_{15}FN$ $[M+H]^+$: 252.1189, found: 252.1167.

(*Z*)-2-(2-fluoro-3-(4-methoxyphenyl)but-1-en-1-yl)benzonitrile (3f)

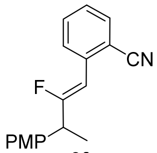

3f

Following general procedure, the reaction mixture was stirred for 16 h and **3f** was obtained as white solid (32.1 mg, 0.11 mmol, 57%, rr > 100:2). **^{1}H NMR (400 MHz, CDCl$_3$):** δ = 7.81 (d, *J* = 8.1 Hz, 1H), 7.54 (dd, *J* = 7.8, 1.5 Hz, 1H), 7.44 (td, *J* = 7.9, 1.4 Hz, 1H), 7.26 – 7.15 (m, 3H), 6.86 – 6.79 (m, 2H), 5.97 (d, *J* = 37.2 Hz, 1H), 3.72 (s, 3H), 3.67 (dq, *J* = 16.9, 7.0 Hz, 1H), 1.48 (d, *J* = 7.1 Hz, 3H). **^{19}F NMR (376 MHz, CDCl$_3$):** δ = -98.91 (dd, *J* = 37.2, 17.3 Hz). **^{13}C NMR (100 MHz, CDCl$_3$):** δ = 166.4 (d, *J* = 274.9 Hz), 158.7, 136.8 (d, *J* = 3.1 Hz), 133.0, 132.64 (d, *J* = 3.3 Hz), 129.1 (d, *J* = 13.8 Hz), 128.4, 126.9 (d, *J* = 1.9 Hz), 118.0, 114.0, 111.0, 101.5 (d, *J* = 7.9 Hz), 55.2, 42.6 (d, *J* = 25.0 Hz), 18.6 (d, *J* = 4.1 Hz). **HRMS (ESI, m/z):** calcd. for $C_{18}H_{17}FNO$ $[M+H]^+$: 282.1294, found: 282.1300.

(*Z*)-1-(2-fluoro-3-(4-methoxyphenyl)but-1-en-1-yl)-4-(trifluoromethyl)benzene (3g)

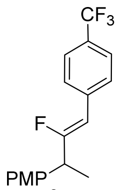

3g

Following general procedure, the reaction mixture was stirred for 16 h and **3g** was obtained as colourless liquid (40.9 mg, 0.13 mmol, 63%, rr > 100:1). **^{1}H NMR (400 MHz, CDCl$_3$):** δ = 7.52 – 7.42 (m, 4H), 7.20 – 7.14 (m, 2H), 6.85 – 6.79 (m, 2H), 5.51 (d, *J* = 38.7 Hz, 1H), 3.72 (s, 3H), 3.64 (dq, *J* = 14.5, 7.1 Hz, 1H), 1.45 (d, *J* = 7.1 Hz, 3H). **^{19}F NMR (376 MHz, CDCl$_3$):** δ = -62.47, -100.55 (dd, *J* = 38.4, 15.5 Hz). **^{13}C NMR (100 MHz, CDCl$_3$):** δ = 165.3 (d, *J* = 271.9 Hz), 158.6, 137.2, 133.4, 128.5 (d, *J* = 8.0 Hz), 128.5, 125.2 (q, *J* = 3.8 Hz), 124.2 (q, *J* = 271.2 Hz), 114.0, 104.4 (d, *J* = 8.4 Hz), 55.3, 42.5 (d, *J* = 25.9 Hz), 18.7 (d, *J* = 4.0 Hz). **HRMS (ESI, m/z):** calcd. for $C_{18}H_{17}F_4O$ $[M+H]^+$: 325.1216, found: 325.1220.

(*Z*)-1-(2-fluoro-3-(4-methoxyphenyl)but-1-en-1-yl)-3-(trifluoromethyl)benzene (3h)

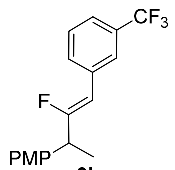

3h

Following general procedure, the reaction mixture was stirred for 17 h and **3h** was obtained as colourless liquid (32.5 mg, 0.10 mmol, 51%, rr > 100:1). **^{1}H NMR (400 MHz, CDCl$_3$):** δ = 7.70 (s, 1H), 7.62 (d, *J* = 7.5 Hz, 1H), 7.47 – 7.36 (m, 2H), 7.27 – 7.22 (m, 2H), 5.57 (d, *J* = 38.5 Hz, 1H), 3.80 (s, 3H), 3.71 (dq, *J* = 14.5, 7.1 Hz, 1H), 1.52 (d, *J* = 7.2 Hz, 3H). **^{19}F NMR (376 MHz, CDCl$_3$):** δ = -62.74 , -101.54 (dd, *J* = 38.5, 15.5 Hz). **^{13}C NMR (100 MHz, CDCl$_3$):** δ = 164.9 (d, *J* = 271.0 Hz), 158.6, 134.3 (d, *J* = 2.4 Hz), 133.5, 131.5 (dq, *J* = 7.8, 1.4 Hz), 130.7 (q, *J* = 31.3Hz). 128.8, 128.5, 125.1 (dq, *J* = 7.8, 3.9 Hz), 124.1 (q, *J* = 272.7Hz), 123.44 – 123.26 (m), 114.0, 104.3 (d, *J* = 8.4 Hz), 55.3, 42.4 (d, *J* = 25.9 Hz), 18.7 (d, *J* = 4.3 Hz). **HRMS (ESI, m/z):** calcd. for $C_{18}H_{17}F_4O$ $[M+H]^+$: 325.1216, found: 325.1201.

(*Z*)-1-(2-fluoro-3-(4-methoxyphenyl)but-1-en-1-yl)-2-(trifluoromethyl)benzene (3i)

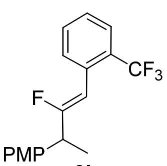

3i

Following general procedure, the reaction mixture was stirred for 17 h and **3i** was obtained as colourless liquid (27.9 mg, 0.08 mmol, 43%, rr > 100:1). **^{1}H NMR (400 MHz, CDCl$_3$):** δ = 8.14 (s, 1H), 7.78 (d, 2H), 7.55 (d, *J* = 3.6 Hz, 1H), 7.45 (d, *J* = 8.2 Hz, 1H), 7.40 – 7.36 (m, 5H), 7.30 (dq, *J* = 8.5, 4.5, Hz, 1H), 7.22 (d, *J* = 8.1 Hz, 2H), 6.61 (d, *J* = 3.5 Hz, 1H), 5.73 (d, *J* = 38.5 Hz, 1H), 3.80 (dq, *J* = 14.4, 7.1 Hz, 1H), 2.34 (s, 3H), 1.59 (d, *J* = 7.1 Hz, 3H). **^{19}F NMR (376 MHz, CDCl$_3$):** δ = -60.24, -104.13 (dd, *J* = 36.5, 15.1 Hz). **^{13}C NMR (100 MHz, CDCl$_3$):** δ = 164.4 (d, *J* = 271.2 Hz), 158.6, 133.5, 131.7 (t, *J* = 1.5Hz), 131.5 (q, *J* = 1.0 Hz), 130.97 (d, *J* = 10.2 Hz), 128.4, 127.4 (q, *J* = 30.0 Hz) 126.6 (d, *J* = 1.0 Hz), 125.6 (q, *J* = 5.8 Hz), 124.3 (q, *J* = 272 Hz), 114.0, 101.1 (dq, *J* = 8.4, 2.1 Hz), 55.3, 42.4 (d, *J* = 25.8 Hz), 18.8 (d, *J* = 4.3 Hz). **HRMS (ESI, m/z):** calcd. for $C_{18}H_{17}F_4O$ [M+H]$^+$: 325.1216, found: 325.1212.

(*Z*)-1-(4-(2-fluoro-3-phenylbut-1-en-1-yl)phenyl)ethan-1-one (3j)

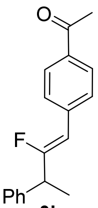

3j

Following general procedure, the reaction mixture was stirred for 12 h and **3j** was obtained as white solid (42.9 mg, 0.16 mmol, 80%, rr > 100:1). **^{1}H NMR (400 MHz, CDCl$_3$):** δ = 7.90 (d, *J* = 8.4 Hz, 2H), 7.55 (d, *J* = 8.4 Hz, 2H), 7.39 – 7.26 (m, 5H), 5.64 (d, *J* = 38.8 Hz, 1H), 3.77 (dq, *J* = 14.6, 7.2 Hz, 1H), 2.58 (s, 3H), 1.57 (d, *J* = 7.2 Hz, 3H). **^{19}F NMR (376 MHz, CDCl$_3$):** δ = -99.33 (dd, *J* = 38.8, 15.8 Hz). **^{13}C NMR (100 MHz, CDCl$_3$):** δ = 197.6, 165.2 (d, *J* = 272.9 Hz), 141.5, 138.5 (d, *J* = 2.9 Hz), 135.2, 128.7, 128.54, 128.46, 127.5, 127.2, 105.1 (d, *J* = 8.4 Hz), 43.4 (d, *J* = 25.7 Hz), 26.6, 18.7 (d, *J* = 4.3 Hz). **HRMS (ESI, m/z):** calcd. for $C_{18}H_{18}FO$ [M+H]$^+$: 269.1342, found: 269.1341.

((*Z*)-1-(2-fluoro-3-(4-methoxyphenyl)but-1-en-1-yl)-4-(trifluoromethoxy)benzene (3k)

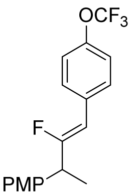

3k

Following general procedure, the reaction mixture was stirred for 16 h and **3k** was obtained as colourless liquid (32.0 mg, 0.09 mmol, 47%, rr > 100:2). **^{1}H NMR (400 MHz, CDCl$_3$):** δ = 7.59 – 7.50 (m, 4H), 7.28 – 7.21 (m, 2H), 6.91 – 6.86 (m, 2H), 5.58 (d, *J* = 38.7 Hz, 1H), 3.80 (s, 3H), 3.71 (dq, *J* = 14.6, 7.0 Hz, 1H), 1.52 (d, *J* = 7.2 Hz, 3H). **^{19}F NMR (376 MHz, CDCl$_3$):** δ = -57.83, -103.32 (dd, *J* = 38.9, 15.3 Hz). **^{13}C NMR (100 MHz, CDCl$_3$):** δ = 165.3 (d, *J* = 271.9 Hz), 158.6, 137.2 (q, *J* = 1.4 Hz), 133.4, 128.5 (d, *J* = 7.9 Hz), 128.5, 126.5 (q, *J* = 270.4 Hz), 125.5, 125.2 (q, *J* = 3.8 Hz), 114.0, 104.4 (d, *J* = 8.5 Hz), 55.3, 42.5 (d, *J* = 25.6 Hz), 18.7 (d, *J* = 4.1 Hz). **HRMS (ESI, m/z):** calcd. for $C_{18}H_{17}F_4O_2$ [M+H]$^+$: 341.1165, found: 341.1170.

(*Z*)-2,4-dichloro-1-(2-fluoro-3-(4-methoxyphenyl)but-1-en-1-yl)benzene (3l)

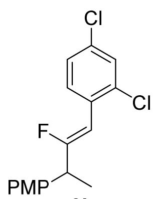

3l

Following general procedure, the reaction mixture was stirred for 16 h and **3l** was obtained as colourless liquid (32.5 mg, 0.10 mmol, 50%, rr > 100:1). **^{1}H NMR (400 MHz, CDCl$_3$):** δ = 7.62 (d, *J* = 8.6 Hz, 1H), 7.29 (d, *J* = 2.2 Hz, 1H), 7.22 – 7.14 (m, 2H), 7.10 (dd, *J* = 8.6, 2.2 Hz, 1H), 6.81 (d, *J* = 8.5 Hz, 2H), 5.86 (d, *J* = 38.1 Hz, 1H), 3.73 (s, 3H), 3.64 (dq, *J* = 14.6, 7.3 Hz, 1H), 1.46 (d, *J* = 7.2 Hz, 3H). **^{19}F NMR (376 MHz, CDCl$_3$):** δ = -102.78 (dd, *J* = 38.1, 15.8 Hz). **^{13}C NMR (100 MHz, CDCl$_3$):** δ = 165.0 (d, *J* = 271.8 Hz), 158.6, 133.4, 133.1, 132.7 (d, *J* = 2.4 Hz), 130.9 (d, *J* = 12.8 Hz), 130.0 (d, *J* = 2.6 Hz), 129.0, 128.4, 127.0, 114.0, 100.6 (d, *J* = 7.4 Hz), 55.3, 42.6 (d, *J* = 25.7 Hz), 18.8 (d, *J* = 4.2 Hz). **HRMS (ESI, m/z):** calcd. for $C_{17}H_{16}Cl_2FO$ [M+H]$^+$: 325.0562, found: 325.0560.

(*Z*)-3-fluoro-4-(2-fluoro-3-(4-methoxyphenyl)but-1-en-1-yl)benzonitrile (3m)

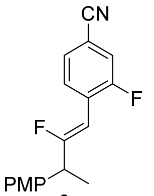

3m

Following general procedure, the reaction mixture was stirred for 20 h and **3m** was obtained as white solid (24.3 mg, 0.09 mmol, 45%, rr > 100:1). **^{1}H NMR (400 MHz, CDCl$_3$):** δ = 7.81 (t, *J* = 7.8 Hz, 1H), 7.32 – 7.27 (m, 1H), 7.23 (dd, *J* = 9.9, 1.7 Hz, 1H), 7.18 – 7.14 (m, 2H), 6.84 – 6.79 (m, 2H), 5.79 (d, *J* = 38.1 Hz, 1H), 3.72 (s, 3H), 3.71 – 3.58 (m, 1H), 1.47 (d, *J* = 7.2 Hz, 3H). **^{19}F NMR (376 MHz, CDCl$_3$):** δ = -96.71 (ddd, *J* = 38.1, 16.1, 3.8 Hz), -114.27 (ddd, *J* = 10.6, 7.4, 3.7 Hz). **^{13}C NMR (100 MHz, CDCl$_3$):** δ = 167.5 (dd, *J* = 276.0, 2.4 Hz), 158.8, 158.5 (dd, *J* = 251.7, 1.2 Hz), 132.8, 130.6 (dd, *J* = 14.9, 3.4 Hz), 128.4, 128.0 (d, *J* = 3.9 Hz), 126.9 (dd, *J* = 12.0, 2.8 Hz), 118.7 (d, *J* = 25.7 Hz), 117.7 (d, *J* = 2.9 Hz), 114.1, 111.0 (dd, *J* = 9.8, 2.3 Hz), 95.7 (t, *J* = 7.3 Hz), 55.2, 42.8 (d, *J* = 25.0 Hz), 18.6 (d, *J* = 4.2 Hz). **HRMS (ESI, m/z):** calcd. for $C_{18}H_{16}F_2NO$ [M+H]$^+$: 300.1200, found: 300.1203.

(*Z*)-1-(2-fluoro-3-phenylbut-1-en-1-yl)-3-methoxybenzene (3n)

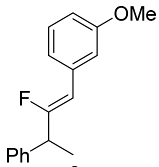

3n

Following general procedure, the reaction mixture was stirred for 16 h and **3n** was obtained as colourless liquid (25.6 mg, 0.10 mmol, 51%, rr > 100:1). **^{1}H NMR (400 MHz, CDCl$_3$):** δ = 7.29-7.25 (m, 4H), 7.24 – 7.15 (m, 1H), 7.15 (t, *J* = 7.9 Hz, 1H), 7.04 – 6.94 (m, 2H), 6.71 (ddd, *J* = 8.3, 2.6, 0.9 Hz, 1H), 5.51 (d, *J* = 39.2 Hz, 1H), 3.72 (s, 3H), 3.72-3.63(dq,*J* = 15.5, 7.3 Hz, 1H), 1.47 (d, *J* = 7.2 Hz, 3H). **^{19}F NMR (376 MHz, CDCl$_3$):** δ = -102.80 (dd, *J* = 39.2, 15.5 Hz). **^{13}C NMR (100 MHz, CDCl$_3$):** δ = 163.3 (d, *J* = 269.2 Hz), 159.5, 141.9, 134.8 (d, *J* = 2.5 Hz), 129.3, 128.6, 127.5, 126.9, 121.1 (d, *J* = 6.9 Hz), 113.6 (d, *J* = 8.4 Hz), 112.9 (d, *J* = 2.1 Hz), 105.6 (d, *J* = 8.4 Hz), 55.1, 43.3 (d, *J* = 26.1 Hz), 18.8 (d, *J* = 4.4 Hz). **HRMS (ESI, m/z):** calcd. for $C_{17}H_{17}FO$ [M+H]$^+$: 257.1342, found: 257.1349.

(*Z*)-3-(2-fluoro-3-phenylbut-1-en-1-yl)phenyl 4-methylbenzenesulfonate (3o)

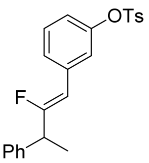

3o

Following general procedure, the reaction mixture was stirred for 12 h and **3o** was obtained as colourless liquid (39.0 mg, 0.12 mmol, 60%, rr > 100:2). **^{1}H NMR (400 MHz, CDCl$_3$):** δ = 7.71 (d, *J* = 8.3 Hz, 2H), 7.32 (m, 8H), 7.20 (t, *J* = 8.0 Hz, 1H), 6.80 (dd, *J* = 8.1, 2.3 Hz, 1H), 5.52 (d, *J* = 39.2 Hz, 1H), 3.72 (dq, *J* = 14.6, 7.2 Hz, 1H), 2.41 (s, 3H), 1.52 (d, *J* = 7.2 Hz, 3H). **^{19}F NMR (376 MHz, CDCl$_3$):** δ = -101.23 (dd, *J* = 38.6, 15.4 Hz). **^{13}C NMR (100 MHz, CDCl$_3$):** δ = 164.2 (d, *J* = 271.2 Hz), 149.6, 145.3, 141.5, 135.3 (d, *J* = 2.4 Hz), 132.3, 129.7, 129.3, 128.6, 128.5, 127.4, 127.07, 127.04 (d, *J* = 7.6 Hz), 122.3 (d, *J* = 8.2 Hz), 120.5 (d, *J* = 2.2 Hz), 104.5 (d, *J* = 8.2 Hz), 43.2 (d, *J* = 25.6 Hz), 21.6, 18.7 (d, *J* = 4.3 Hz). **HRMS (ESI, m/z):** calcd. for $C_{23}H_{22}FO_3S$ [M+H]$^+$: 397.1274, found: 397.1271.

(*Z*)-N-(3-(2-fluoro-3-(4-methoxyphenyl)but-1-en-1-yl)phenyl)acetamide (3p)

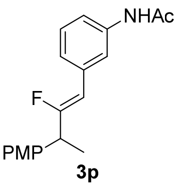

3p

Following general procedure, the reaction mixture was stirred for 24 h and **3p** was obtained as colourless liquid (25.1 mg, 0.08 mmol, 41%, rr > 100:1). **^{1}H NMR (400 MHz, CDCl$_3$):** δ = 7.56 (s, 1H), 7.43 – 7.39 (m, 2H), 7.25 – 7.19 (m, 4H), 6.87 (d, *J* = 8.6 Hz, 2H), 5.52 (d, *J* = 39.3 Hz, 1H), 3.80 (s, 3H), 3.68 (dq, *J* = 14.5, 7.1 Hz, 1H), 2.14 (s, 3H), 1.50 (d, *J* = 7.1 Hz, 3H). **^{19}F NMR (376 MHz, CDCl$_3$):** δ = -102.84 (dd, *J* = 39.2, 15.1 Hz). **^{13}C NMR (100 MHz, CDCl$_3$):** δ = 168.5, 163.8 (d, *J* = 269.3 Hz), 158.5, 137.9, 134.3 (d, *J* = 2.4 Hz), 133.8, 128.9, 128.4, 124.4 (d, *J* = 7.7 Hz), 119.7 (d, *J* = 7.6 Hz), 118.5 (d, *J* = 1.7 Hz), 113.9, 105.1 (d, *J* = 8.4 Hz), 55.2, 42.4 (d, *J* = 26.0 Hz), 24.5, 18.8 (d, *J* = 4.0 Hz). **HRMS (ESI, m/z):** calcd. for $C_{19}H_{21}FNO_2$ [M+H]$^+$: 314.1556, found: 314.1566.

(*Z*)-3-(2-fluoro-3-phenylbut-1-en-1-yl)quinoline (3q)

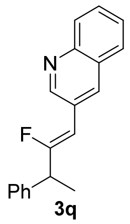

3q

Following general procedure, the reaction mixture was stirred for 24 h and **3q** was obtained as white solid (22.2 mg, 0.08 mmol, 40%, rr > 100:1). **^{1}H NMR (400 MHz, CDCl$_3$):** δ = 8.85 (d, *J* = 2.2 Hz, 1H), 8.25 (d, *J* = 2.1 Hz, 1H), 7.98 (d, *J* = 8.4 Hz, 1H), 7.69 (dd, *J* = 8.1, 1.4 Hz, 1H), 7.59 (ddd, *J* = 8.4, 6.9, 1.5 Hz, 1H), 7.44 (ddd, *J* = 8.1, 6.8, 1.2 Hz, 1H), 7.30 (m, 4H), 7.26 – 7.19 (m, 1H), 5.68 (d, *J* = 39.1 Hz, 1H), 3.76 (dq, *J* = 14.5, 7.1 Hz, 1H), 1.53 (d, *J* = 7.2 Hz, 3H). **^{19}F NMR (376 MHz, CDCl$_3$):** δ = -99.58 (dd, *J* = 39.2, 15.3 Hz). **^{13}C NMR (100 MHz, CDCl$_3$):** δ = 165.5 (d, *J* = 271.1 Hz), 150.9 (d, *J* = 5.7 Hz), 146.6 (d, *J* = 2.1 Hz), 141.4, 134.3 (d, *J* = 10.5 Hz), 129.2, 128.9, 128.7, 128.0, 127.9, 127.5, 127.2, 126.8, 102.6 (d, *J* = 9.5 Hz), 43.4 (d, *J* = 25.5 Hz), 29.7, 18.7 (d, *J* = 4.2 Hz). **HRMS (ESI, m/z):** calcd. for $C_{19}H_{17}FN$ [M+H]$^+$: 278.1345, found: 278.1347.

(*Z*)-6-(2-fluoro-3-phenylbut-1-en-1-yl)-1-tosyl-1*H*-indole (3r)

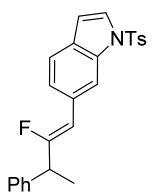

3r

Following general procedure, the reaction mixture was stirred for 12 h and **3r** was obtained as colourless liquid (33.6 mg, 0.08 mmol, 40%, rr > 100:1). **^{1}H NMR (400 MHz, CDCl$_3$):** δ = 8.14 (s, 1H), 7.78 (d, 2H), 7.55 (d, *J* = 3.6 Hz, 1H), 7.45 (d, *J* = 8.2 Hz, 1H), 7.40 – 7.36 (m, 5H), 7.30 (dq, *J* = 8.5, 4.5, Hz, 1H), 7.22 (d, *J* = 8.1 Hz, 2H), 6.61 (d, *J* = 3.5 Hz, 1H), 5.73 (d, *J* = 38.5 Hz, 1H), 3.80 (dq, *J* = 14.4, 7.1 Hz, 1H), 2.34 (s, 3H), 1.59 (d, *J* = 7.1 Hz, 3H). **^{19}F NMR (376 MHz, CDCl$_3$):** δ = -103.56 (dd, *J* = 39.1, 15.4 Hz). **^{13}C NMR (100 MHz, CDCl$_3$):** δ = 163.1 (d, *J* = 268.7 Hz), 145.0, 142.2, 135.3 (d, *J* = 9.2 Hz), 130.4 (d, *J* = 2.8 Hz), 130.0, 129.6 (d, *J* = 2.1 Hz), 128.8, 127.6, 127.1, 127.0, 126.8, 124.24 (d, *J* = 7.6 Hz), 121.2, 113.4 (d, *J* = 8.2 Hz), 109.0, 106.1 (d, *J* = 8.2 Hz), 43.5 (d, *J* = 26.0 Hz), 21.7, 19.1 (d, *J* = 4.4 Hz). **HRMS (ESI, m/z):** calcd. for $C_{25}H_{23}FNO_2S$ [M+H]$^+$: 420.1434, found: 420.1431.

(*Z*)-1-(4-(2-fluoro-3-(p-tolyl)but-1-en-1-yl)phenyl)ethan-1-one (3s)

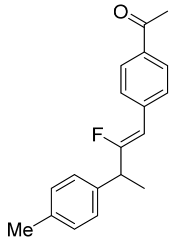

3s

Following general procedure, the reaction mixture was stirred for 12 h and **3s** was obtained as white solid (36.8 mg, 0.13 mmol, 65%, rr > 100:1). **^{1}H NMR (400 MHz, CDCl$_3$):** δ = 7.81 (d, *J* = 8.5 Hz, 1H), 7.46 (d, *J* = 8.5 Hz, 1H), 7.14 (d, *J* = 8.1 Hz, 1H), 7.08 (d, *J* = 7.8 Hz, 1H), 5.54 (d, *J* = 38.9 Hz, 1H), 3.65 (dq, *J* = 14.5, 7.1 Hz, 1H), 2.50 (s, 2H), 2.26 (s, 2H), 1.46 (d, *J* = 7.2 Hz, 2H). **^{19}F NMR (376 MHz, CDCl$_3$):** δ = -99.35 (dd, *J* = 38.8, 15.5 Hz). **^{13}C NMR (100 MHz, CDCl$_3$):** δ = 197.6, 165.4 (d, *J* = 273.1 Hz), 138.5 (d, *J* = 2.8 Hz), 138.4, 136.8, 135.1 (d, *J* = 2.2 Hz), 129.3, 128.5, 128.4, 127.3, 104.9 (d, *J* = 8.6 Hz), 43.0 (d, *J* = 25.5 Hz), 26.5, 21.0, 18.7 (d, *J* = 4.0 Hz). **HRMS (ESI, m/z):** calcd. for $C_{19}H_{20}FO$ [M+H]$^+$: 283.1498, found: 283.1506.

(*Z*)-1-(4-(2-fluoro-3-(4-methoxyphenyl)but-1-en-1-yl)phenyl)ethan-1-one (3t)

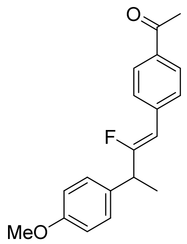

3t

Following general procedure, the reaction mixture was stirred for 20 h and **3t** was obtained as white solid (36.8 mg, 0.13 mmol, 65%, rr > 100:1). **^{1}H NMR (400 MHz, CDCl$_3$):**δ = 7.81 (d, *J* = 8.5 Hz, 2H), 7.46 (d, *J* = 8.5 Hz, 2H), 7.14 (d, *J* = 8.1 Hz, 2H), 7.08 (d, *J* = 7.8 Hz, 2H), 5.54 (d, *J* = 38.9 Hz, 1H), 3.65 (dq, *J* = 14.5, 7.1 Hz, 1H), 2.50 (s, 3H), 2.26 (s, 3H), 1.46 (d, *J* = 7.2 Hz, 3H). **^{19}F NMR (376 MHz, CDCl$_3$):** δ = -99.61 (dd, *J* = 38.9, 15.3 Hz). **^{13}C NMR (100 MHz, CDCl$_3$):** δ = 197.5, 165.5 (d, *J* = 273.0 Hz), 158.6, 138.5 (d, *J* = 2.7 Hz), 135.2 (d, *J* = 2.2 Hz), 133.4, 128.5, 128.4, 128.4, 114.0, 104.8 (d, *J* = 8.4 Hz), 55.2, 42.6 (d, *J* = 25.6 Hz), 26.5, 18.7 (d, *J* = 3.9 Hz). **HRMS (ESI, m/z):** calcd. for $C_{19}H_{20}FO_2$ [M+H]$^+$: 299.1447, found: 299.1454.

(*Z*)-1-(4-(3-(4-((tert-butyldimethylsilyl)oxy)phenyl)-2-fluorobut-1-en-1-yl)phenyl)ethan-1-one (3u)

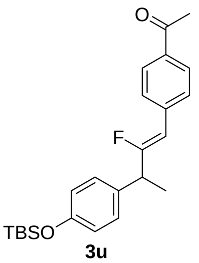

3u

Following general procedure, the reaction mixture was stirred for 12 h and **3u** was obtained as colourless liquid (46.3 mg, 0.12 mmol, 58%, rr > 100:1). **^{1}H NMR (400 MHz, CDCl$_3$):** δ = 7.89 (d, *J* = 8.5 Hz, 2H), 7.54 (d, *J* = 8.5 Hz, 2H), 7.18 (d, *J* = 8.5 Hz, 2H), 6.82 (d, *J* = 8.5 Hz, 2H), 5.59 (d, *J* = 38.9 Hz, 1H), 3.71 (dq, *J* = 14.5, 7.1 Hz, 1H), 2.58 (s, 3H), 1.53 (d, *J* = 7.2 Hz, 3H), 0.99 (s, 9H), 0.20 (s, 6H). **^{19}F NMR (376 MHz, CDCl$_3$):** δ = -99.47 (dd, *J* = 38.9, 15.4 Hz). **^{13}C NMR (100 MHz, CDCl$_3$):** δ = 197.6, 165.6 (d, *J* = 273.2 Hz), 154.6, 138.6 (d, *J* = 2.7 Hz), 135.1 (d, *J* = 2.1 Hz), 133.9, 128.48, 128.4 (d, *J* = 7.8 Hz), 128.39, 120.1, 104.8 (d, *J* = 8.4 Hz), 42.6 (d, *J* = 25.6 Hz), 26.5, 25.6, 18.7 (d, *J* = 3.9 Hz), 18.1, -4.4. **HRMS (ESI, m/z):** calcd. for $C_{24}H_{31}FNaO_2Si$ [M+Na]$^+$:421.1975, found: 421.1976.

(*Z*)-1-(4-(3-(4-chlorophenyl)-2-fluorobut-1-en-1-yl)phenyl)ethan-1-one (3v)

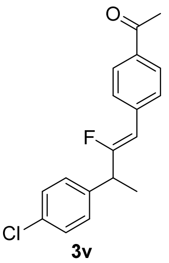

3v

Following general procedure, the reaction mixture was stirred for 12 h and **3v** was obtained as white solid (36.2 mg, 0.12 mmol, 60%, rr > 100:2). **^{1}H NMR (400 MHz, CDCl$_3$):** δ = 7.82 (d, *J* = 8.4 Hz, 2H), 7.46 (d, *J* = 8.4 Hz, 2H), 7.27 – 7.21 (m, 2H), 7.21 – 7.16 (m, 2H), 5.55 (d, *J* = 38.8 Hz, 1H), 3.66 (dq, *J* = 14.5, 7.1 Hz, 1H), 2.50 (s, 3H), 1.46 (d, *J* = 7.1 Hz, 3H). **^{19}F NMR (376 MHz, CDCl$_3$):** δ = -99.98 (dd, *J* = 38.8, 15.6 Hz). **^{13}C NMR (100 MHz, CDCl$_3$):** δ = 197.5, 164.4 (d, *J* = 272.6 Hz), 139.9, 138.1 (d, *J* = 2.7 Hz), 135.3 (d, *J* = 2.2 Hz), 132.9, 128.81, 128.79, 128.5, 128.4, 105.3 (d, *J* = 8.2 Hz), 42.8 (d, *J* = 25.9 Hz), 26.5, 18.6 (d, *J* = 4.1 Hz). **HRMS (ESI, m/z):** calcd. for $C_{18}H_{17}ClFO$ [M+H]$^+$: 303.0952, found: 303.0955.

(*Z*)-1-(4-(2-fluoro-3-(4-fluorophenyl)but-1-en-1-yl)phenyl)ethan-1-one (3w)

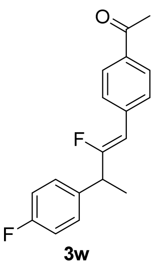

3w

Following general procedure, the reaction mixture was stirred for 12 h and **3w** was obtained as white solid (20.1 mg, 0.07 mmol, 33%, rr > 100:1). **^{1}H NMR (400 MHz, CDCl$_3$):** δ = 7.82 (d, *J* = 8.5 Hz, 2H), 7.46 (d, *J* = 8.5 Hz, 2H), 7.21 (dd, *J* = 8.6, 5.4 Hz, 2H), 6.96 (t, *J* = 8.7 Hz, 2H), 5.55 (d, *J* = 38.8 Hz, 1H), 3.68 (dq, *J* = 14.5, 7.2 Hz, 1H), 2.50 (s, 3H), 1.46 (d, *J* = 7.2 Hz, 3H). **^{19}F NMR (376 MHz, CDCl$_3$):** δ = -99.97 (dd, *J* = 38.8, 15.3 Hz), -115.62 (ddd, *J* = 13.9, 8.8, 5.2 Hz). **^{13}C NMR (100 MHz, CDCl$_3$):** δ = 197.5, 164.8 (d, *J* = 272.8 Hz), 161.9 (d, *J* = 245.4 Hz), 138.2 (d, *J* = 2.7 Hz), 137.1 (d, *J* = 3.2 Hz), 135.3 (d, *J* = 2.2 Hz), 129.0 (d, *J* = 8.0 Hz), 128.5, 128.4, 115.5 (d, *J* = 21.3 Hz), 105.1 (d, *J* = 8.3 Hz), 42.7 (d, *J* = 25.8 Hz), 26.5, 18.8 (d, *J* = 4.0 Hz). **HRMS (ESI, m/z):** calcd. for $C_{18}H_{17}F_2O$ [M+H]$^+$: 287.1247, found: 287.1247.

methyl (*Z*)-4-(2-fluoro-3-(4-hydroxyphenyl)but-1-en-1-yl)benzoate (3x)

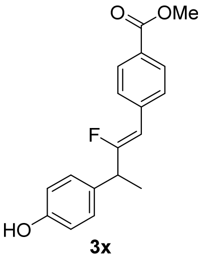

3x

Following general procedure, the reaction mixture was stirred for 20 h and **3x** was obtained as white solid (27.0 mg, 0.09 mmol, 45%, rr > 100:2). **^{1}H NMR (400 MHz, CDCl$_3$)**: δ 7.96 (d, *J* = 8.4 Hz, 2H), 7.52 (d, *J* = 8.4 Hz, 2H), 7.20 (d, *J* = 8.5 Hz, 2H), 6.82 (d, *J* = 8.5 Hz, 2H), 5.59 (d, *J* = 38.9 Hz, 1H), 3.90 (s, 3H), 3.70 (dq, *J* = 14.5, 7.2 Hz, 1H), 1.52 (d, *J* = 7.2 Hz, 3H). **^{19}F NMR (376 MHz, CDCl$_3$):** δ = -99.82 (dd, *J* = 38.9, 15.4 Hz). **^{13}C NMR (100 MHz, CDCl$_3$):** δ = 167.0, 165.3 (d, *J* = 272.5 Hz), 154.7, 138.3 (d, *J* = 2.7 Hz), 133.6, 129.7, 128.7, 128.3 (d, *J* = 8.0 Hz), 128.0 (d, *J* = 2.4 Hz), 115.5, 104.8 (d, *J* = 8.2 Hz), 52.1, 42.5 (d, *J* = 25.7 Hz), 18.7 (d, *J* = 4.1 Hz). **HRMS (ESI, m/z):** calcd. for $C_{18}H_{18}FO_3$ [M+H]$^+$: 301.1240, found: 301.1243.

(*Z*)-1-(4-(3-(3-chlorophenyl)-2-fluorobut-1-en-1-yl)phenyl)ethan-1-one (3y)

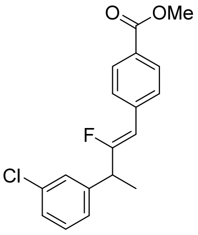

3y

Following general procedure, the reaction mixture was stirred for 12 h and **3y** was obtained as colourless liquid (34.0 mg, 0.12 mmol, 62%, rr > 100:1). **^{1}H NMR (400 MHz, CDCl$_3$):** δ = 7.92 – 7.87 (m, 2H), 7.56 – 7.53 (m, 2H), 7.31 (t, *J* = 1.9 Hz, 1H), 7.30 – 7.24 (m, 2H), 7.21 (tt, *J* = 7.0, 1.9 Hz, 1H), 5.65 (d, *J* = 38.7 Hz, 1H), 3.73 (dq, *J* = 14.5, 7.4 Hz, 1H), 2.57 (s, 3H), 1.53 (d, *J* = 7.2 Hz, 3H). **^{19}F NMR (376 MHz, CDCl$_3$):** δ = -99.92 (dd, *J* = 41.6, 17.7 Hz). **^{13}C NMR (100 MHz, CDCl$_3$):** δ = 197.5, 164.1 (d, *J* = 272.8 Hz), 143.4, 138.1 (d, *J* = 2.8 Hz), 135.3 (d, *J* = 2.2 Hz), 134.4, 129.9, 128.52 (d, *J* = 4.0 Hz), 128.47 (d, *J* = 4.0 Hz), 127.6, 127.3, 125.7, 105.4 (d, *J* = 8.2 Hz), 43.1 (d, *J* = 25.9 Hz), 26.5, 18.5 (d, *J* = 4.3 Hz). **HRMS (ESI, m/z):** calcd. for $C_{18}H_{17}FOCl$ $[M+H]^+$: 319.0901, found: 319.0907.

methyl (*Z*)-4-(2-fluoro-3-(3-(trifluoromethyl)phenyl)but-1-en-1-yl)benzoate (3z)

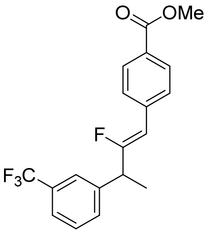

3z

Following general procedure, the reaction mixture was stirred for 12 h and **3z** was obtained as colourless liquid (43.1 mg, 0.12 mmol, 61%, rr > 100:1). **^{1}H NMR (400 MHz, CDCl$_3$)**:δ = 8.02 – 7.96 (m, 2H), 7.58 (s, 1H), 7.57 – 7.51 (m, 4H), 7.50 – 7.44 (m, 1H), 5.68 (d, *J* = 38.7 Hz, 1H), 3.91 (s, 3H), 3.87 – 3.77 (dq, J = 16, 7.4 Hz), 1.58 (d, *J* = 7.2 Hz, 3H). **^{19}F NMR (376 MHz, CDCl$_3$):** δ = -62.37, -100.66 (dd, *J* = 38.8, 16.0 Hz). **^{13}C NMR (100 MHz, CDCl$_3$):** δ = 166.8, 163.7 (d, *J* = 272.1 Hz), 142.5, 137.8 (d, *J* = 2.9 Hz), 131.0 (q, *J* = 32.2 Hz), 130.9, 129.7, 129.2, 128.4 (d, *J* = 2.4 Hz), 128.4 (d, *J* = 7.8 Hz), 124.2 (q, *J* = 3.8 Hz), 124.1 (q, *J* = 3.8 Hz), 124.0 (q, *J* = 271.7 Hz), 105.6 (d, *J* = 8.2 Hz), 52.1, 43.3 (d, *J* = 25.9 Hz), 18.6 (d, *J* = 4.2 Hz). **HRMS (ESI, m/z):** calcd. for $C_{19}H_{17}F_4O_2$ $[M+H]^+$: 353.1165, found: 353.1169.

(*Z*)-1-(4-(2-fluoro-3-phenylpent-1-en-1-yl)phenyl)ethan-1-one (3aa)

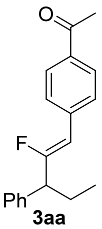

3aa

Following general procedure, the reaction mixture was stirred for 16 h and **3aa** was obtained as colourless liquid (23.8 mg, 0.08 mmol, 42%, rr > 100:6). **^{1}H NMR (400 MHz, CDCl$_3$):**δ = 7.81 (d, *J* = 8.5 Hz, 2H), 7.47 (d, *J* = 8.5 Hz, 2H), 7.33 – 7.15 (m, 5H), 5.59 (d, *J* = 38.9 Hz, 1H), 3.35 (dt, *J* = 21.2, 7.7 Hz, 1H), 2.51 (s, 3H), 1.91 (ddq, *J* = 80.3, 13.7, 7.4 Hz, 2H), 0.89 (t, *J* = 7.4 Hz, 3H). **^{19}F NMR (376 MHz, CDCl$_3$):** δ = -101.41 (dd, *J* = 38.9, 21.2 Hz). **^{13}C NMR (100 MHz, CDCl$_3$):** δ = 197.6, 163.9 (d, *J* = 274.1 Hz), 140.3, 138.5 (d, *J* = 2.6 Hz), 135.1 (d, *J* = 2.1 Hz), 128.6, 128.5, 128.4 (d, *J* = 8.0 Hz), 127.9 , 127.1, 105.7 (d, *J* = 8.4 Hz), 51.5 (d, *J* = 24.1 Hz), 26.5, 25.6 (d, *J* = 3.3 Hz), 12.2. **HRMS (ESI, m/z):** calcd. for $C_{19}H_{20}FO$ $[M+H]^+$: 283.1498, found: 283.1494.

(*Z*)-1-(4-(2-fluoro-3-phenylhex-1-en-1-yl)phenyl)ethan-1-one (3ab) (from (4-bromobutyl)benzene **1k**)

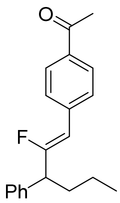

3ab

Following general procedure, the reaction mixture was stirred for 20 h and **3ab** was obtained as white solid (23.8 mg, 0.08 mmol, 40%, rr > 100:7). **^{1}H NMR (400 MHz, CDCl$_3$):**δ = 7.81 (d, *J* = 8.3 Hz, 2H), 7.46 (d, *J* = 8.4 Hz, 2H), 7.30 – 7.17 (m, 5H), 5.58 (d, *J* = 38.9 Hz, 1H), 3.45 (dt, *J* = 21.3, 7.7 Hz, 1H), 2.50 (s, 3H), 2.03 – 1.68 (m, 2H), 1.40 – 1.19 (m, 2H), 0.87 (t, *J* = 7.4 Hz, 3H). **^{19}F NMR (376 MHz, CDCl$_3$):** δ = -101.28 (dd, *J* = 38.8, 21.2 Hz). **^{13}C NMR (100 MHz, CDCl$_3$):** δ = 197.5, 164.1 (d, *J* = 274.2 Hz), 140.5, 138.5 (d, *J* = 2.7 Hz), 135.2 (d, *J* = 2.2 Hz), 128.6, 128.5, 128.4 (d, *J* = 8.1 Hz), 127.9 , 127.1, 105.6 (d, *J* = 8.5 Hz), 49.5 (d, *J* = 24.2 Hz), 34.6 (d, *J* = 2.8 Hz), 26.5, 20.7, 13.9. **HRMS (ESI, m/z):** calcd. for $C_{20}H_{22}FO$ $[M+H]^+$: 297.1655, found: 297.1661.

(*Z*)-1-(4-(2-fluoro-3-phenylhept-1-en-1-yl)phenyl)ethan-1-one (3ac)

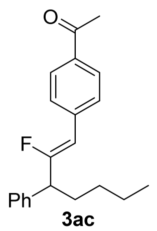
3ac

Following general procedure, the reaction mixture was stirred for 20 h and **3ac** was obtained as white solid (12.4 mg, 0.04 mol, 20%, rr > 100:6). **^{1}H NMR (400 MHz, CDCl$_3$):** δ = 7.80 (d, *J* = 8.4 Hz, 2H), 7.46 (d, *J* = 8.2 Hz, 2H), 7.31 – 7.14 (m, 5H), 5.58 (d, *J* = 38.9 Hz, 1H), 3.42 (dt, *J* = 21.3, 7.7 Hz, 1H), 2.49 (s, 3H), 2.14 – 1.67 (m, 2H), 1.42 – 1.09 (m, 2H), 0.81 (t, *J* = 6.8 Hz, 3H). **^{19}F NMR (376 MHz, CDCl$_3$):** δ = -101.32 (dd, *J* = 38.9, 21.2 Hz). **^{13}C NMR (100 MHz, CDCl$_3$):** δ = 197.5, 164.1 (d, *J* = 274.1 Hz), 140.5, 138.5 (d, *J* = 2.7 Hz), 135.1 (d, *J* = 2.2 Hz), 128.6, 128.46, 128.40 (d, *J* = 8.0 Hz), 127.8, 127.1, 105.6 (d, *J* = 8.5 Hz), 49.7 (d, *J* = 24.2 Hz), 32.1 (d, *J* = 2.9 Hz), 29.7, 26.5, 22.5, 13.9. **HRMS (ESI, m/z):** calcd. for $C_{21}H_{24}FO$ $[M+H]^+$: 311.1811, found: 298.1819.

(*Z*)-1-(4-(2-fluoro-3-phenylhex-1-en-1-yl)phenyl)ethan-1-one (3ab) (from (2-bromobutyl)benzene **1m**)

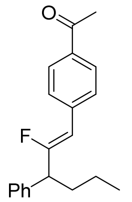
3ab

Following general procedure, the reaction mixture was stirred for 20 h and **3ab** was obtained as white solid (20.8 mg, 0.07 mmol, 35%, rr > 100:12). **^{1}H NMR (400 MHz, CDCl$_3$):**δ = 7.81 (d, *J* = 8.3 Hz, 2H), 7.46 (d, *J* = 8.4 Hz, 2H), 7.30 – 7.17 (m, 5H), 5.58 (d, *J* = 38.9 Hz, 1H), 3.45 (dt, *J* = 21.3, 7.7 Hz, 1H), 2.50 (s, 3H), 2.03 – 1.68 (m, 2H), 1.40 – 1.19 (m, 2H), 0.87 (t, *J* = 7.4 Hz, 3H). **^{19}F NMR (376 MHz, CDCl$_3$):** δ = -101.28 (dd, *J* = 38.8, 21.2 Hz). **^{13}C NMR (100 MHz, CDCl$_3$):** δ = 197.5, 164.1 (d, *J* = 274.2 Hz), 140.5, 138.5 (d, *J* = 2.7 Hz), 135.2 (d, *J* = 2.2 Hz), 128.6, 128.5, 128.4 (d, *J* = 8.1 Hz), 127.9 , 127.1, 105.6 (d, *J* = 8.5 Hz), 49.5 (d, *J* = 24.2 Hz), 34.6 (d, *J* = 2.8 Hz), 26.5, 20.7, 13.9. **HRMS (ESI, m/z):** calcd. for $C_{20}H_{22}FO$ $[M+H]^+$: 297.1655, found: 297.1661.

(*Z*)-1-(4-(2-fluoro-3-phenylhex-1-en-1-yl)phenyl)ethan-1-one (3ab) (from (3-bromobutyl)benzene **1n**)

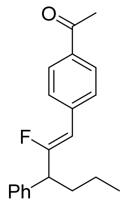
3ab

Following general procedure, the reaction mixture was stirred for 20 h and **3ab** was obtained as white solid (18.0 mg, 0.06 mmol, 30%, rr > 100:8). **^{1}H NMR (400 MHz, CDCl$_3$):**δ = 7.81 (d, *J* = 8.3 Hz, 2H), 7.46 (d, *J* = 8.4 Hz, 2H), 7.30 – 7.17 (m, 5H), 5.58 (d, *J* = 38.9 Hz, 1H), 3.45 (dt, *J* = 21.3, 7.7 Hz, 1H), 2.50 (s, 3H), 2.03 – 1.68 (m, 2H), 1.40 – 1.19 (m, 2H), 0.87 (t, *J* = 7.4 Hz, 3H). **^{19}F NMR (376 MHz, CDCl$_3$):** δ = -101.28 (dd, *J* = 38.8, 21.2 Hz). **^{13}C NMR (100 MHz, CDCl$_3$):** δ = 197.5, 164.1 (d, *J* = 274.2 Hz), 140.5, 138.5 (d, *J* = 2.7 Hz), 135.2 (d, *J* = 2.2 Hz), 128.6, 128.5, 128.4 (d, *J* = 8.1 Hz), 127.9 , 127.1, 105.6 (d, *J* = 8.5 Hz), 49.5 (d, *J* = 24.2 Hz), 34.6 (d, *J* = 2.8 Hz), 26.5, 20.7, 13.9. **HRMS (ESI, m/z):** calcd. for $C_{20}H_{22}FO$ $[M+H]^+$: 297.1655, found: 297.1661.

5. Synthetic applications

Hydrogenation of **3a**

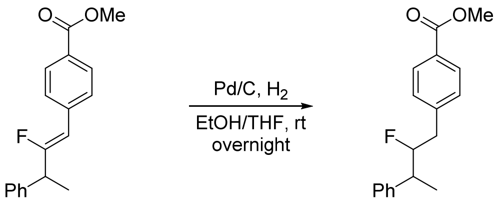

3a, 0.1 mmol   **4a**, 80% yield

An 25 mL two-neck round bottom flask was charged with **3a** (28.4 mg, 0.10 mmol, 1.0 equiv) and Pd/C (15%Wt) and EtOH/THF (1.0 mL, v/v 1:1). The RBF was purged three times with H_2 and sealed, then the reaction mixture was allowed to stirred at room temperature. After the reaction was completed, the H_2 was released and the solvent was evaporated under reduced pressure. The crude product was purified by column chromatography on silica gel (petroleum ether/ethyl acetate = 50 : 1) to produce the product **4a** (23.0 mg, 0.08 mmol, dr = 69:31) in 80% yield as colourless liquid.

Major isomer (16.1 mg, 0.025 mmol, colourless liquid)

^{1}H NMR (400 MHz, CDCl$_3$): δ = 8.02 – 7.91 (m, 1H), 7.38 – 7.32 (m, 2H), 7.31 – 7.22 (m, 5H), 4.91 – 4.72 (m, 1H), 3.90 (s, 3H), 2.96 (m, 1H), 2.89 – 2.78 (m, 2H), 1.41 (d, *J* = 7.2 Hz, 3H). **^{19}F NMR (376 MHz, CDCl$_3$):** δ = -184.60 – -184.96 (m). **^{13}C NMR (100 MHz, CDCl$_3$):** δ = 167.0, 143.1 (d, *J* = 2.9 Hz), 141.6 (d, *J* = 2.5 Hz), 129.7, 129.3 (d, *J* = 0.7 Hz), 128.5, 128.4, 128.4 (d, *J* = 1.5 Hz), 126.8, 97.2 (d, *J* = 177.9 Hz), 52.0, 43.9 (d, *J* = 20.1 Hz), 39.1 (d, *J* = 22.1 Hz), 17.6 (d, *J* = 5.3 Hz). **HRMS (ESI, m/z):** calcd. for $C_{18}H_{20}FO_2$ [M+H]$^+$: 287.1447, found: 287.1445.

Minor isomer (7.2 mg, 0.055 mmol, colourless liquid)

^{1}H NMR (400 MHz, CDCl$_3$): δ = 7.93 (d, *J* = 8.1 Hz, 2H), 7.33 (t, *J* = 7.3 Hz, 2H), 7.26 (d, *J* = 8.1 Hz, 1H), 7.20 (d, *J* = 7.8 Hz, 4H), 4.74 (dtd, *J* = 48.2, 7.2, 4.7 Hz, 1H), 3.89 (s, 3H), 2.94 (tt, *J* = 15.1, 7.4 Hz, 1H), 2.87 – 2.75 (m, 2H), 1.40 (d, *J* = 6.9 Hz, 3H). **^{19}F NMR (376 MHz, CDCl$_3$):** δ = -183.60 – -183.94 (m). **^{13}C NMR (100 MHz, CDCl$_3$):** δ = 167.0, 143.1 (d, *J* = 1.6 Hz), 142.8 (d, *J* = 6.6 Hz), 129.6, 129.3 (d, *J* = 0.8 Hz), 128.7, 128.4, 127.7, 126.9, 97.5 (d, *J* = 178.4 Hz), 52.0, 44.4 (d, *J* = 20.3 Hz), 39.4 (d, *J* = 21.5 Hz), 17.2 (d, *J* = 5.4 Hz). **HRMS (ESI, m/z):** calcd. for $C_{18}H_{20}FO_2$ [M+H]$^+$: 287.1447, found: 287.1445.

Epoxidation of **3a**

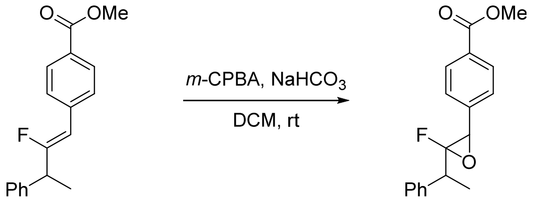

3a, 0.1 mmol — **4b**, 88% yield

In an oven-dried 10 mL Schlenk tube with a stir bar, **3a** (28.4 mg, 0.10 mmol, 1.0 equiv), *m*-CPBA (34.5 mg, 0.20 mmol, 2.0 equiv), $NaHCO_3$ (16.8 mg, 0.20 mmol, 2.0 equiv) and anhydrous DCM (1.0 mL) were added. After stirring at room temperature for 12 h, the mixture was passed through a short pad of celite and rinsed with EtOAc. The solvent was evaporated under reduced pressure, and the crude product was purified by column chromatography on silica gel (petroleum ether/ethyl acetate = 20 : 1) to afford the product **4b** (26.4 mg, 0.088 mmol, dr = 52:48) in 88% yield as off-white solid. **^{1}H NMR (400 MHz, CDCl$_3$):** δ = 8.05 – 7.99 (m, 2H), 7.44 – 7.39 (m, 2H), 7.38 – 7.34 (m, 4H), 7.32 – 7.28 (m, 1H), (3.911, 3.908) (s, 3H), (3.97, 3.78) (d, *J* = 2.0 Hz, 1H), (3.46, 3.32) (dq, *J* = 12.0, 7.2 Hz, 1H), (1.57, 1.55) (d, *J* = 7.2 Hz, 3H). **^{19}F NMR (376 MHz, CDCl$_3$):** δ = (-146.23, -149.07) (d, *J* = 12.2 Hz), -149.07 (d, *J* = 16.4 Hz). **^{13}C NMR (100 MHz, CDCl$_3$):** δ = 166.68, 166.67, 139.5, 139.0 (d, *J* = 3.8 Hz), 137.6 (d, *J* = 4.5 Hz), 137.5 (d, *J* = 4.5 Hz), 130.2, 129.4, 128.65, 128.59, 128.1, 127.91 (d, *J* = 1.2 Hz), 127.5, 127.4, 127.1 (d, *J* = 1.5 Hz), 101.3 (d, *J* = 19.9 Hz), 98.56 (d, *J* = 20.2 Hz), 61.4 (d, *J* = 19.3 Hz), 61.2 (*J* = 19.5 Hz), 52.2, 42.7 (d, *J* = 28.3 Hz), 42.3 (d, *J* = 29.1 Hz), 15.8 (d, *J* = 2.3 Hz), 15.2 (d, *J* =3.9 Hz). **HRMS (ESI, m/z):** calcd. for $C_{18}H_{17}FO_3Na$ [M+Na]$^+$: 323.1059, found: 323.1059.

Dibromination of **3a**

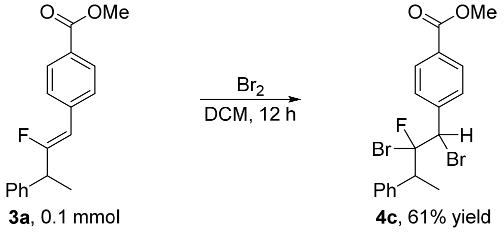

3a, 0.1 mmol — **4c**, 61% yield

To a stirred solution of **3a** (28.4 mg, 0.10 mmol, 1.0 eqiuv) in CH_2Cl_2 (1 mL) at room temperature, the bromine (120 μL, 0.12 mmol, 1.2 equiv) was added in the dark. The reaction mixture was stirred until **3a** was consumed completely. Then the mixture was concentrated under reduced pressure and residue was purified by column chromatography on silica gel (petroleum ether/ethyl acetate = 50 : 1) to give the desired product **4c** (27.0 mg, 0.06 mmol, dr = 52:48) in 61% yield as white solid.

Minor isomer (13 mg, 0.029 mmol, white solid)

^{1}H NMR (400 MHz, CDCl$_3$): δ = 7.97 (d, *J* = 8.7 Hz, 2H), 7.69 – 7.65 (m, 2H), 7.51 – 7.35 (m, 5H), 4.81 (d, *J* = 27.4 Hz, 1H), 4.04 (p, *J* = 6.6 Hz, 1H), 3.90 (s, 3H), 1.62 (d, *J* = 6.8 Hz, 3H). **^{19}F NMR (376 MHz, CDCl$_3$):** δ = - 113.18 (dd, *J* = 27.4, 5.8 Hz). **^{13}C NMR (100 MHz, CDCl$_3$):** δ = 166.5, 143.2 (d, *J* = 1.9 Hz), 138.3 (d, *J* = 8.8 Hz), 130.4, 129.8 (d, *J* = 3.1 Hz), 129.4, 129.3 (d, *J* = 0.8 Hz), 128.5, 128.2, 115.9 (d, *J* = 269.7 Hz), 55.6 (d, *J* = 19.8 Hz), 52.2, 47.9 (d, *J* = 20.9 Hz), 17.6 (d, *J* = 3.2 Hz). **HRMS (ESI, m/z):** calcd. for $C_{18}H_{18}Br_2FO_2$ [M+H]$^+$: 442.9658, found: 442.9661.

Major isomer (14 mg, 0.031 mmol, white solid)

^{1}H NMR (400 MHz, CDCl$_3$): δ = 8.02 (d, *J* = 8.5 Hz, 1H), 7.58 (d, *J* = 7.5 Hz, 2H), 7.46 – 7.29 (m, 4H), 5.34 (d, *J* = 24.7 Hz, 1H), 4.00 – 3.93 (m, 1H), 3.92 (s, 3H), 1.57 (d, *J* = 7.0 Hz, 3H). **^{19}F NMR (376 MHz, CDCl$_3$):** δ = -112.05 (dd, *J* = 24.0, 9.3 Hz). **^{13}C NMR (100 MHz, CDCl$_3$):** δ = 166.4, 142.2, 138.6, 130.6, 130.1 (d, *J* = 3.1 Hz), 130.0 (d, *J* = 1.4 Hz), 129.4, 127.9, 127.7, 114.8 (d, *J* = 267.0 Hz), 55.9 (d, *J* = 21.4 Hz), 52.2, 48.5 (d, *J* = 18.6 Hz), 16.6 (d, *J* = 5.9 Hz). **HRMS (ESI, m/z):** calcd. for $C_{18}H_{18}Br_2FO_2$ [M+H]$^+$: 442.9658, found: 442.9652.

Preparation of allene **4d**

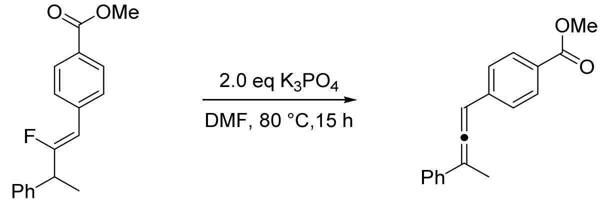

3a, 0.15 mmol **4d**, 70% yield

In an oven-dried 10 mL Schlenk tube with a stir bar, **3a** (42.6 mg, 0.15 mmol, 1.0 equiv), K_3PO_4 (63.7 mg, 0.30 mmol, 2.0 equiv) and anhydrous DMF (1.0 mL) were added, then the reaction tube was purged with N_2 (3 times). After stirring at 80 °C for 12 h, the mixture was passed through a short pad of celite and rinsed with EtOAc. The filtrate was washed with water, brine and dried over Na_2SO_4 and the solvent was evaporated under reduced pressure. The crude product was purified by column chromatography on silica gel (petroleum ether/ethyl acetate = 20 : 1) to afford the product **4d** (25.4 mg, 0.096 mmol) in 64% yield as colourless liquid. **^{1}H NMR (400 MHz, CDCl$_3$)**: δ = 7.98 (dt, *J*= 8.43, 1.80 Hz, 2 H), 7.46 (dt, *J* = 8.2, 1.7 Hz, 2H), 7.39 (dd, *J* = 8.4, 1.7 Hz, 2H), 7.40 – 7.29 (m, 2H), 7.26 (tt, *J* = 6.6, 1.2 Hz, 1H), 6.52 (q, *J* = 2.8 Hz, 1H), 3.91 (s, 3H), 2.26 (d, *J* = 2.9 Hz, 3H). **^{13}C NMR (100 MHz, CDCl$_3$)**: δ = 208.1, 166.9, 139.6, 135.7, 130.0, 128.5, 128.5, 127.3, 126.7, 125.9, 105.1, 96.2, 52.0, 16.6. **HRMS (ESI, m/z)**: calcd. for $C_{18}H_{17}O_2$ [M+H]$^+$: 265.1229, found: 265.1231.

Preparation of 1,2-diketone **4e**

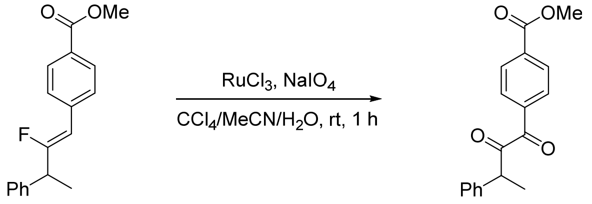

3a, 0.15 mmol **4e**, 52% yield

To an 25 mL round bottom flask with a stir bar, **3a** (42.6 mg, 0.15 mmol, 1.0 equiv), CCl_4 (1.0 mL), MeCN (1.0 mL), H_2O (1.5 mL), $NaIO_4$ (160 mg, 0.75 mmol, 5.0 equiv), and $RuCl_3$ (1.6 mg, 7.5 μmol, 5 mol%) were added, then the reaction tube was purged with N_2 (3 times). After stirring at room temperature for 1 h, the mixture was quenched by saturated aqueous $NaHCO_3$ and extracted with ethyl acetate (5 mL × 3). The combined organic layers were dried over Na_2SO_4 and the solvent was evaporated under reduced pressure. The crude product was purified by column chromatography on silica gel (petroleum ether/ethyl acetate = 20 : 1) to afford the product **4e** (23.2 mg, 0.078 mmol) in 52% yield as yellow oil. **^{1}H NMR (400 MHz, CDCl$_3$)**: δ = 8.01 (d, *J* = 8.8 Hz, 2H), 7.78 (d, *J* = 8.8 Hz, 2H), 7.31 – 7.23 (m, 4H), 7.22 – 7.16 (m, 1H), 4.66 (q, *J* = 7.2 Hz, 1H), 3.92 (s, 3H), 1.57 (d, *J* = 7.2 Hz, 3H). **^{13}C NMR (100 MHz, CDCl$_3$)**: δ = 200.4, 192.4, 165.9, 136.7, 135.6, 134.7, 129.7, 129.6, 129.2, 128.6, 107.8, 52.5, 47.5, 15.7. **HRMS (ESI, m/z)**: calcd. for $C_{18}H_{16}O_4Na$ [M+Na]$^+$: 319.0946, found: 319.0949.

6. Control experiments

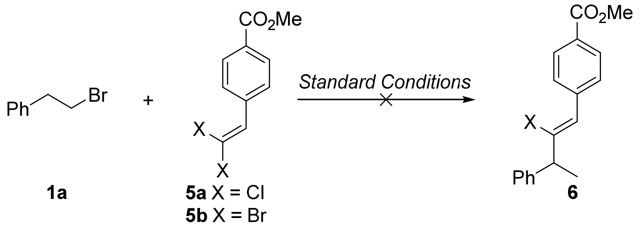

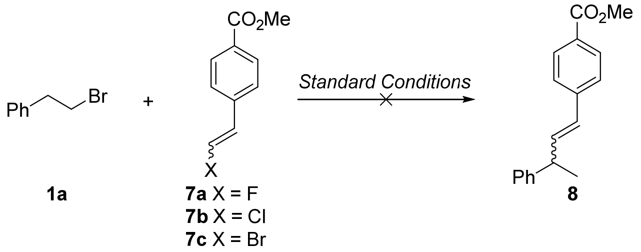

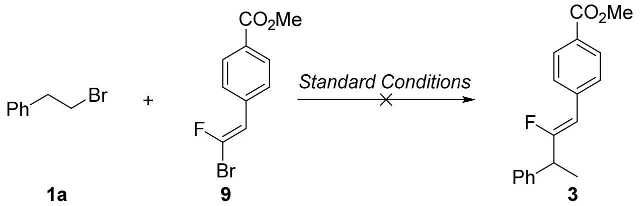

The reactions were carried out according to the general procedure for the fluoro-alkenylation. The crude mixture was analyzed by ^{1}H and ^{19}F NMR, however, no any desired product was detected.

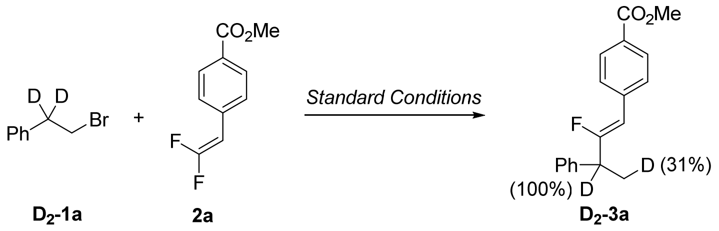

The reaction was carried out according to the general procedure for the fluoro-alkenylation. The crude product was purified by column chromatography on silica gel (petroleum ether : ethyl acetate = 50 : 1) to give the product **D_2-3a** (24 mg) in 43% yield as a white solid.

^{1}H NMR (400 MHz, $CDCl_3$): δ = 7.97 (d, *J* = 8.5 Hz, 2 H), 7.53 (d, *J* = 8.5 Hz, 2 H), 7.43 – 7.32 (m, 4H), 7.31 – 7.25 (m, 1H), 5.63 (d, *J* = 38.9 Hz, 1H), 3.91 (s, 3H), 1.54 (s, 2H). **^{19}F NMR (376 MHz, $CDCl_3$):** δ = -99.78 (d, *J* = 38.8 Hz). **^{13}C NMR (100 MHz, $CDCl_3$):** δ = 166.9, 164.9 (d, *J* = 272.7 Hz), 141.4, 138.2 (d, *J* = 2.6 Hz), 129.7, 128.7, 128.3 (d, *J* = 7.9 Hz), 128.1 (d, *J* = 2.4 Hz), 127.4, 127.1, 105.1 (d, *J* = 8.2 Hz), 52.0, 42.9 (dd, *J* = 19.5, 6.3 Hz), 18.55 – 18.00 (m). **HRMS (ESI, m/z):** calcd. for $C_{18}H_{16}D_2FO_2$ $[M+H]^+$: 287.1416, found: 287.1414.

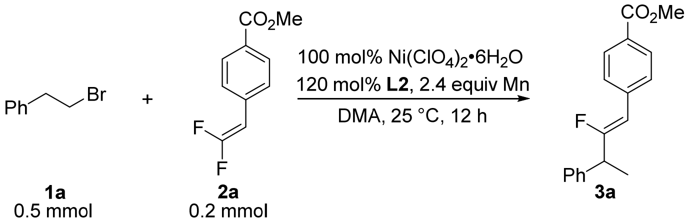

	conversion of **2a**	yield of **3a**
with 0.5 equiv $Yb(OTf)_3$	88%	55%
without $Yb(OTf)_3$	69%	12%

The reactions were carried out according to the general procedure for the fluoro-alkenylation. Conversion of **2a** and yield of **3a** were determined by ^{1}H NMR analysis of the crude residue.

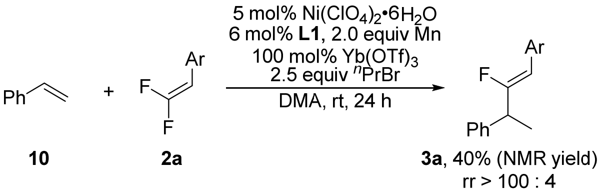

To an oven-dried schlenk tube equipped with a magnetic stir bar was added $Ni(ClO_4)_2·6H_2O$ (3.7 mg, 0.01 mmol, 5.0 mol%), **L1** (2.2 mg, 0.012 mmol, 6.0 mol%) and $Yb(OTf)_3$ (124 mg, 0.2 mmol, 100 mol%). The Schlenk tube was evacuated and filled with nitrogen for three times. To these solids, DMA (1.0 mL) was added under N_2 atmosphere. After stirring at room temperature for 10 min, the styrene **10** (0.4 mmol, 2.0 equiv), 1-bromopropane (0.50 mmol, 2.5 equiv), and *gem*-difluoroalkene **2a** (0.20 mmol, 1.0 equiv) and Mn powder (22 mg, 0.40 mmol, 2.0 equiv) were added under nitrogen and stirred at room temperature for 24 h. The reaction mixture was diluted with ethyl acetate (10 mL) and filtered through a short pad of silica gel. The filtrate was washed with water (10 mL × 2), brine (10 mL) and dried over Na_2SO_4. After solvent was removed under reduced pressure, the crude residue was analyzed by ^1H and ^{19}F NMR with CH_2Br_2 as internal standard.

7. References

[1] S. Ding, L. Xu, P. Li, *ACS Catal.,* 2016, **6**, 1329.

[2] N. Ortega, A. Feher-Voelger, M. Brovetto, J. I. Padron, V. S. Martin, T. Martin, *Adv. Synth. Catal.,* 2011, **353**, 963.

[3] C. S. Thomoson, H. Martinez, W. R. Dolbier, *J. Fluorine Chem.,* 2013, **150**, 53.

[4] T. M. Gøgsig, L. S. Søbjerg, A. T. Lindhardt, K. L. Jensen, T. Skrydstrup, *J. Org. Chem.,* 2008, **73**, 3404.

[5] X. Lei, G. Dutheuil, X. Pannecoucke, J.-C. Quirion, *Org. Lett.,* 2004, **6**, 2101.

[6] S. G. Newman, C. S. Bryan, D. Prez, M. Lautens, *Synthesis,* 2011, **43**, 342.

[7] X. Ma, S. B. Herzon, *J. Org. Chem.,* 2016, **81**, 8673.

[8] G. K. S. Prakash, A. Shakhmin, Z. Milhail, L. Istvan, S. Chacko, G. A. Olah, *J. Fluorine Chem.,* 2010, **131**, 1192.

[9] Marth, C. F. *e-EROS Encyclopedia of Reagents for Organic Synthesis,* 2001, **33**, 1.

[10] D. Chang, Y. Gu, Q. Shen, *Chem. - Eur. J.,* 2015, **21**, 6074.

[11] M. J. Maclean, S. Walker, T.-F. Wang, P. C. H. Eichinger, P. J. Sherman, J. H. Bowie, *Org. Biomol. Chem.,* 2010, **8**, 371.

8. NMR spectra

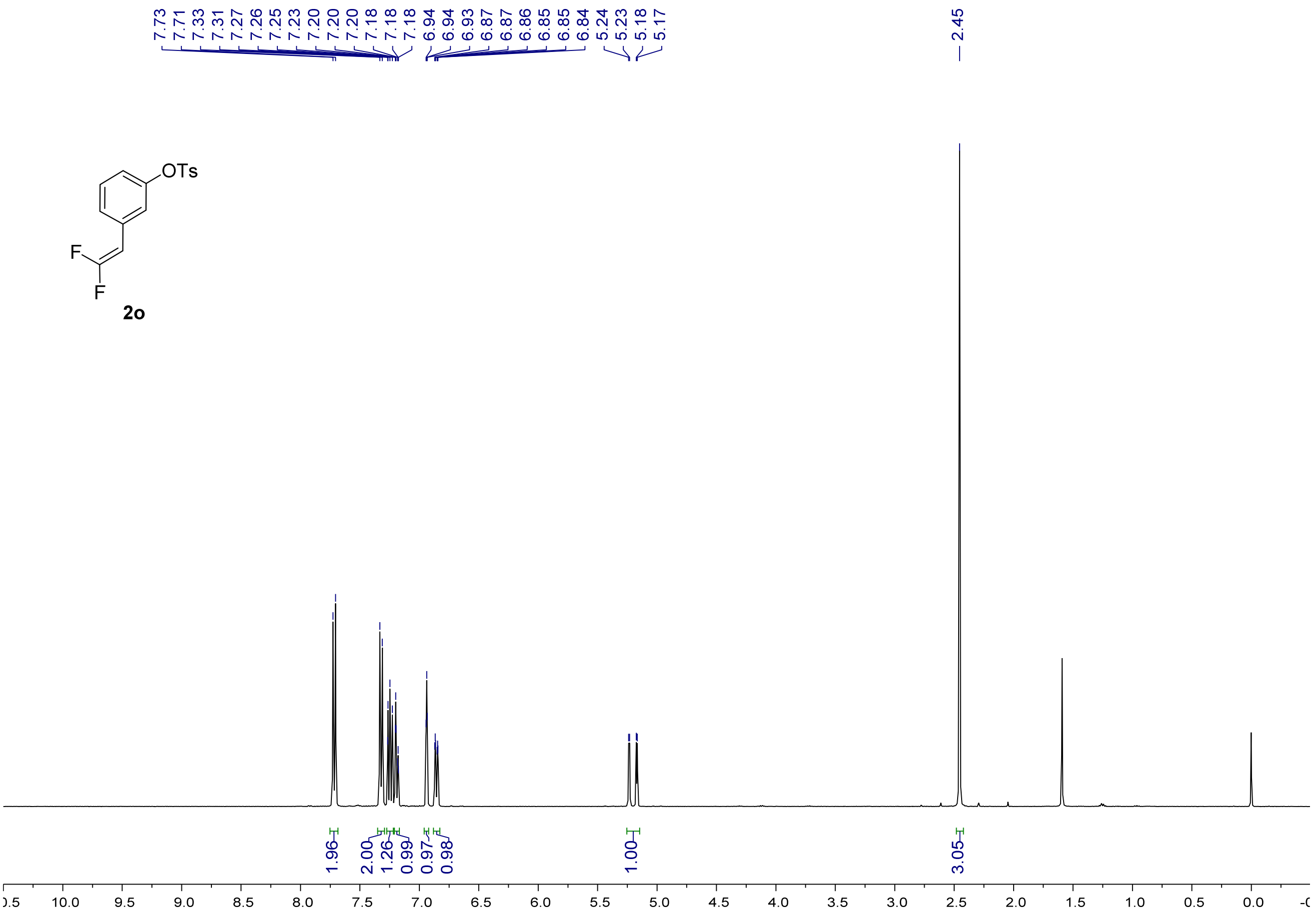

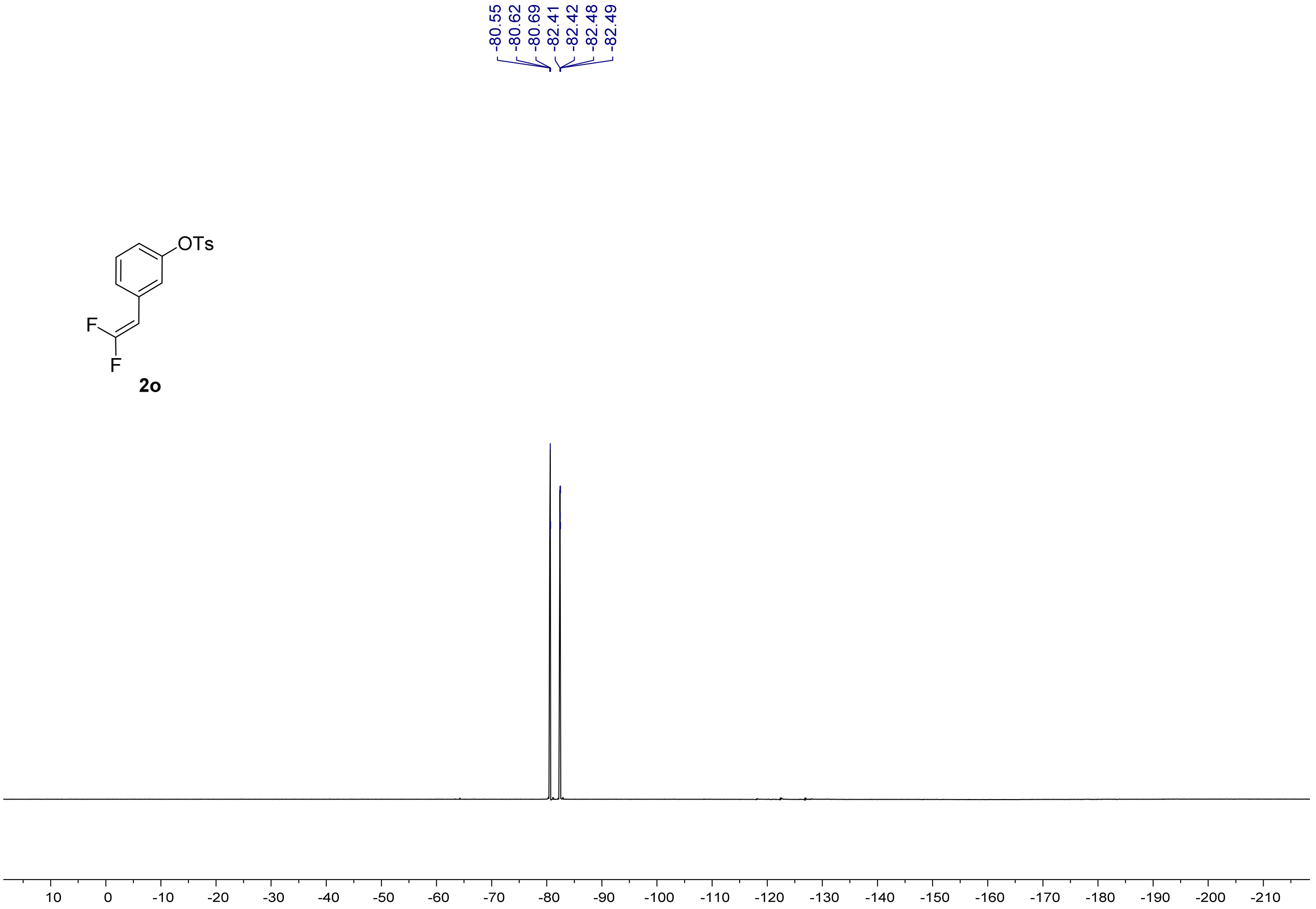

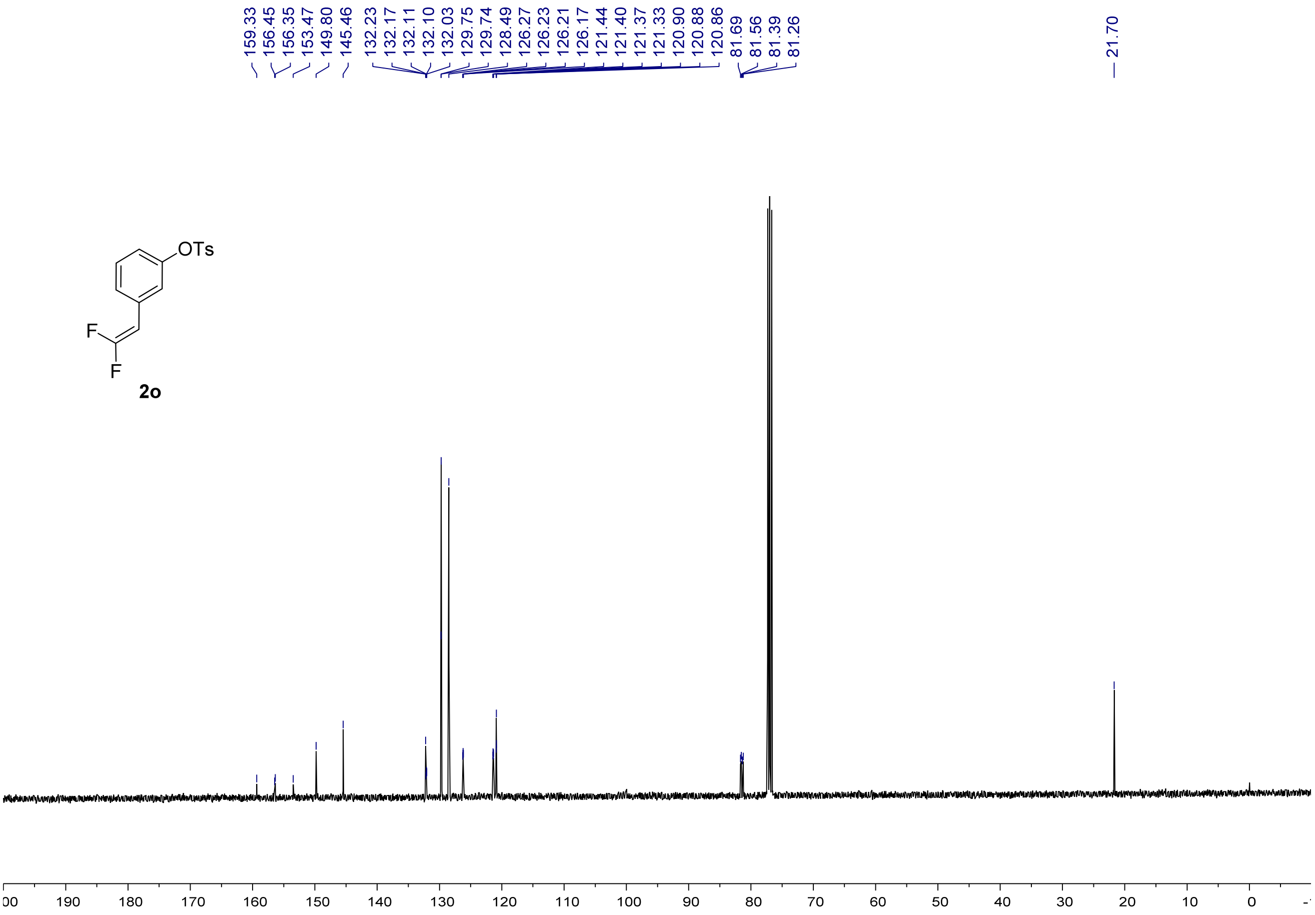
159.33
156.45
156.35
153.47
149.80
145.46
132.23
132.17
132.11
132.10
132.03
129.75
129.74
128.49
126.27
126.23
126.21
126.17
121.44
121.40
121.37
121.33
120.90
120.88
120.86
81.69
81.56
81.39
81.26
21.70
OTs
F
F
190
180
170
160
150
140
130
120
110
100
90
80
70
60
50
40
30
20
10
0

2o

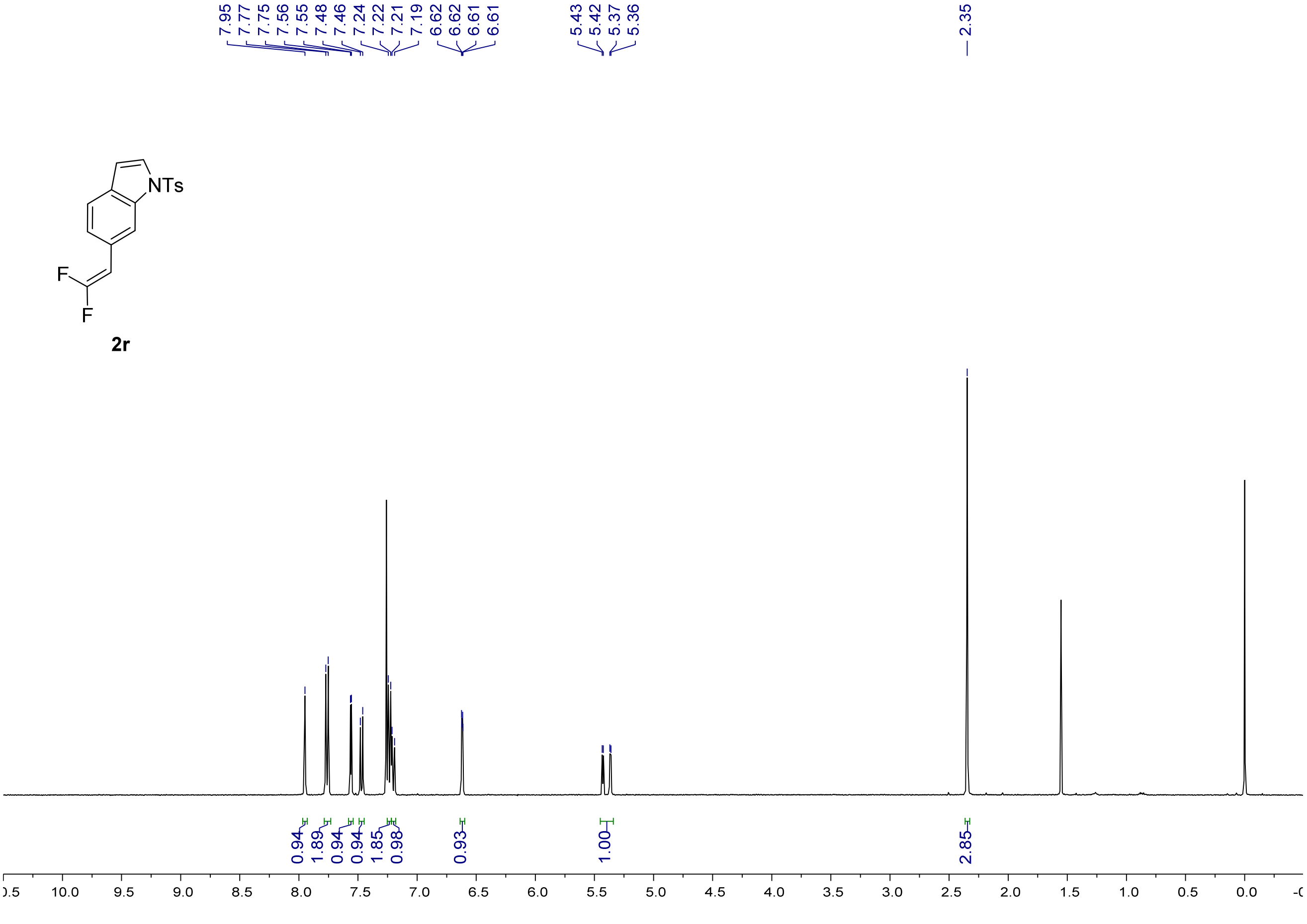
7.95
7.77
7.75
7.56
7.55
7.48
7.46
7.24
7.22
7.21
7.19
6.62
6.62
6.61
6.61
5.43
5.42
5.37
5.36
2.35
NTs
F
F
0.94
1.89
0.94
0.94
1.85
0.98
0.93
1.00
2.85
10.0
9.5
9.0
8.5
8.0
7.5
7.0
6.5
6.0
5.5
5.0
4.5
4.0
3.5
3.0
2.5
2.0
1.5
1.0
0.5
0.0

2r

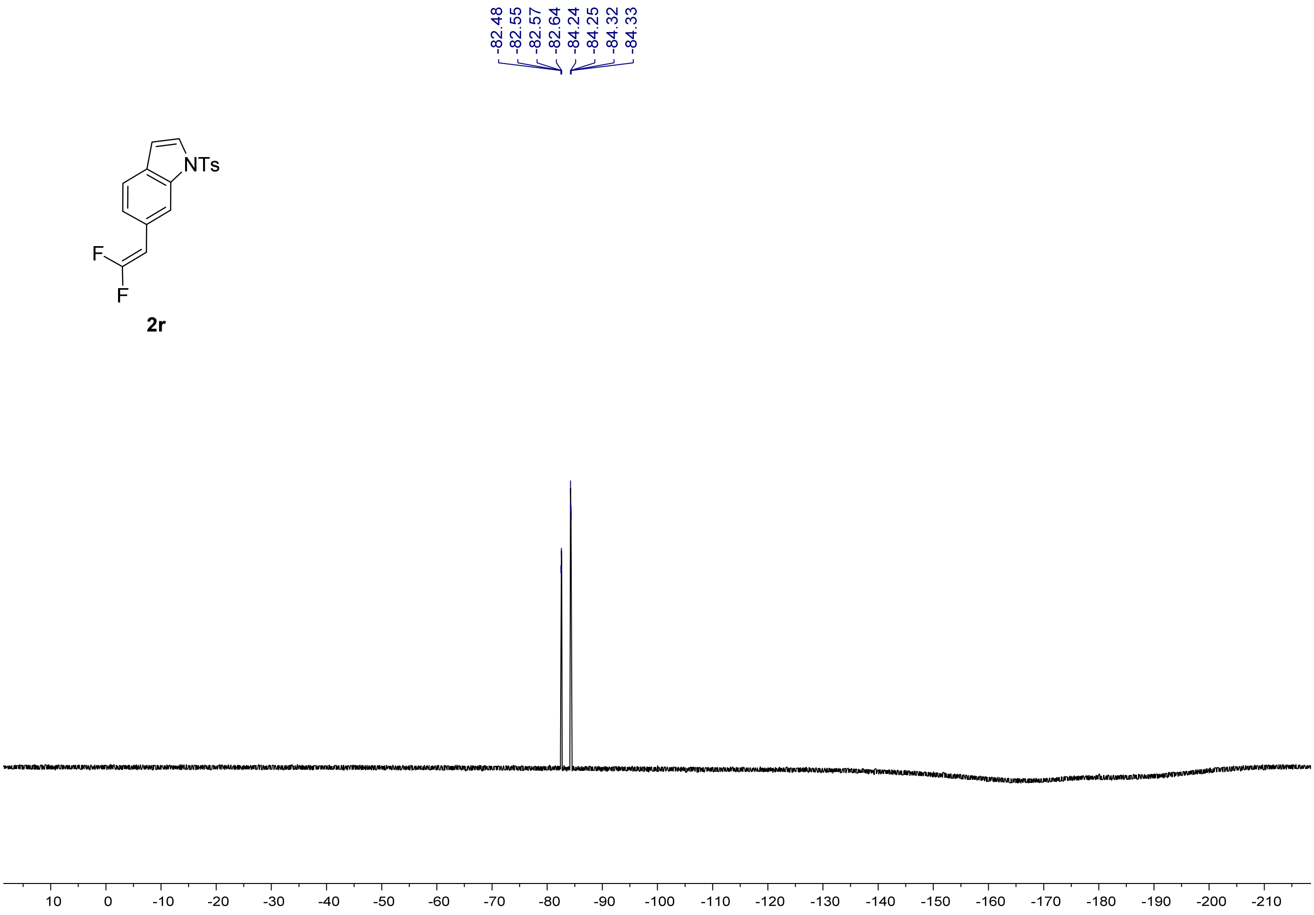

-82.48
-82.55
-82.57
-82.64
-84.24
-84.25
-84.32
-84.33
NTs
F
F
10 0 -10 -20 -30 -40 -50 -60 -70 -80 -90 -100 -110 -120 -130 -140 -150 -160 -170 -180 -190 -200 -210

2r

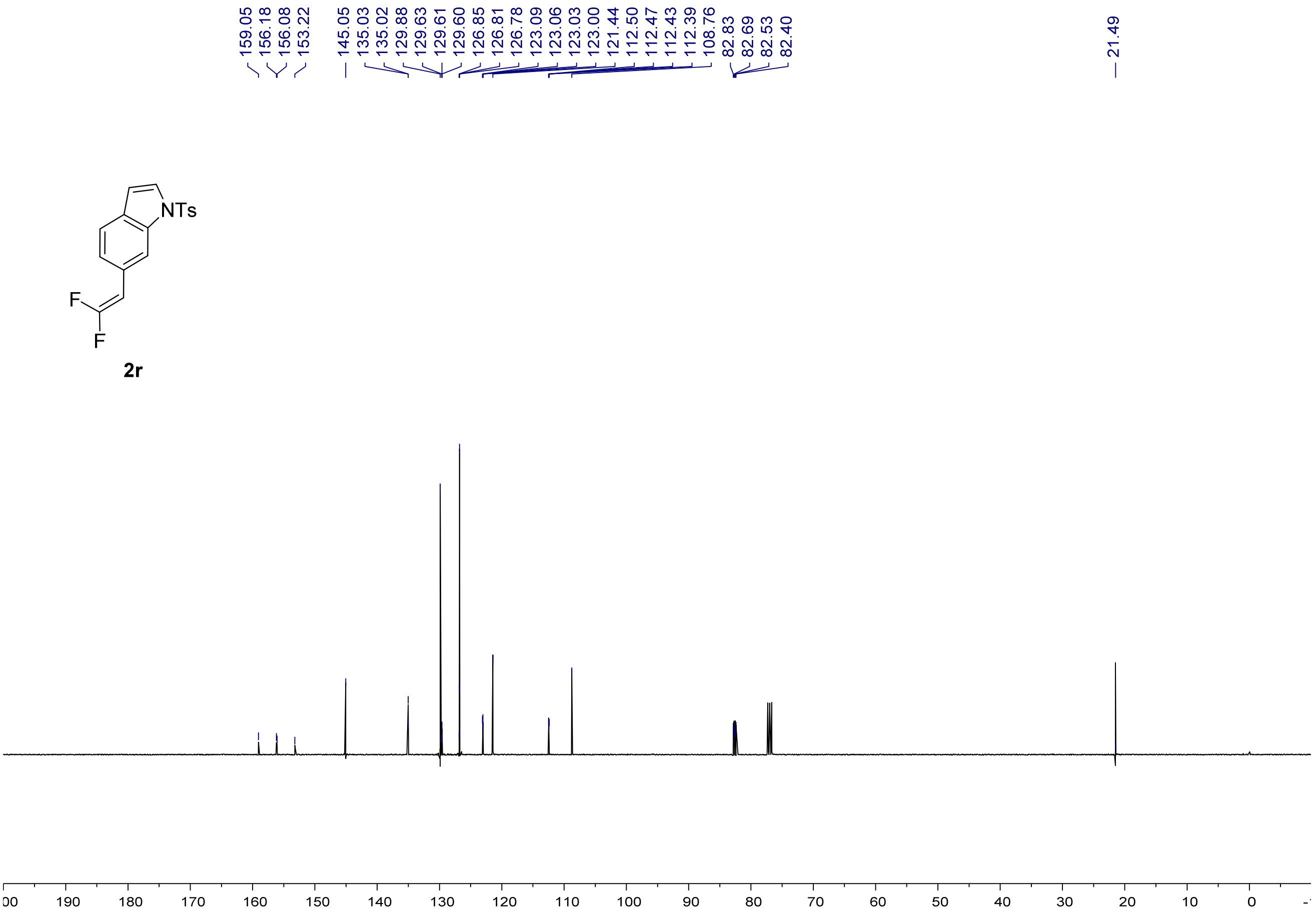

159.05
156.18
156.08
153.22
145.05
135.03
135.02
129.88
129.63
129.61
129.60
126.85
126.81
126.78
123.09
123.06
123.03
123.00
121.44
112.50
112.47
112.43
112.39
108.76
82.83
82.69
82.53
82.40
21.49
NTs
F
F
190 180 170 160 150 140 130 120 110 100 90 80 70 60 50 40 30 20 10 0

2r

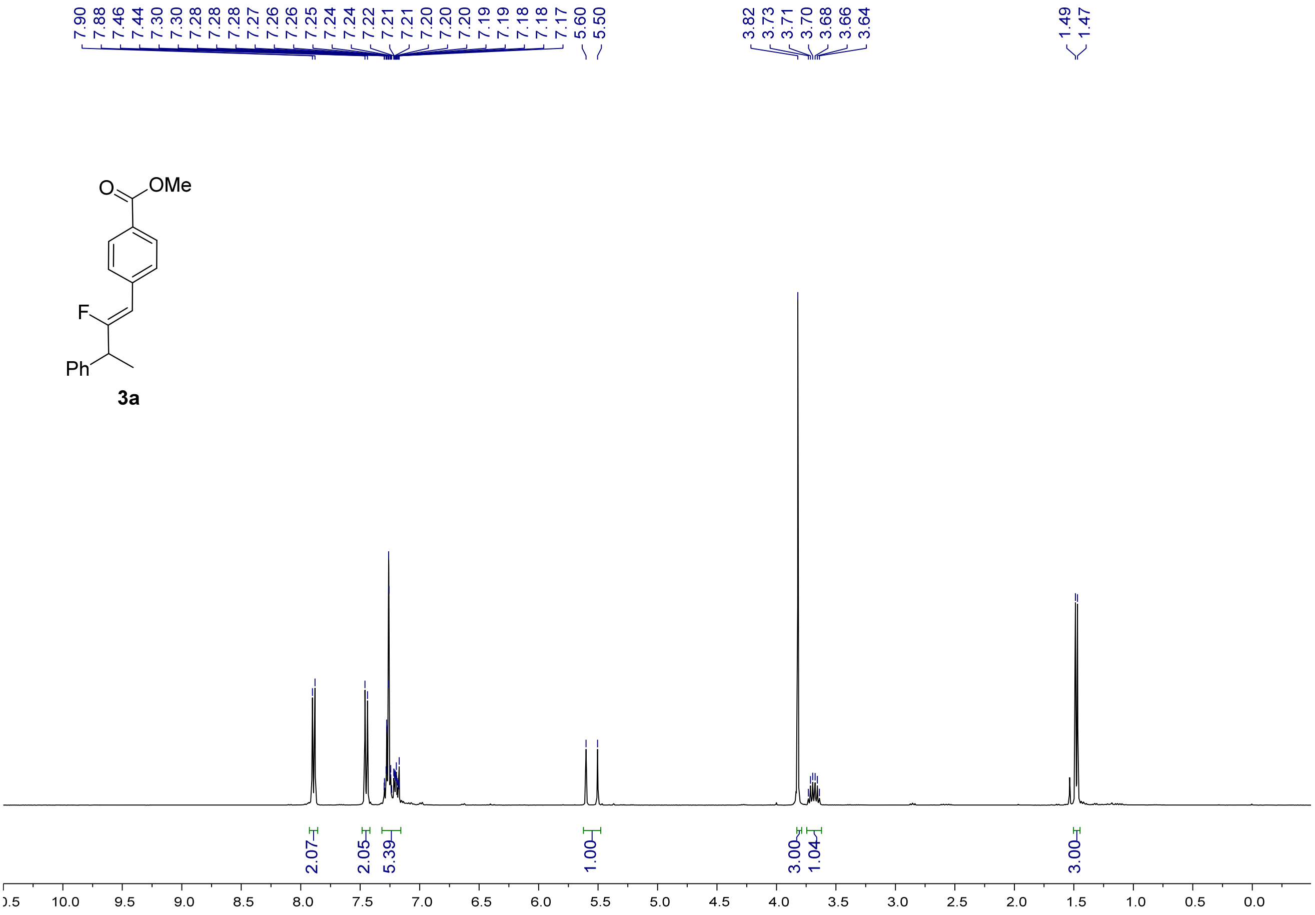
7.90
7.88
7.46
7.44
7.30
7.28
7.28
7.28
7.27
7.26
7.26
7.25
7.24
7.24
7.22
7.21
7.21
7.20
7.20
7.20
7.19
7.19
7.18
7.18
7.17
5.60
5.50
3.82
3.73
3.71
3.70
3.68
3.66
3.64
1.49
1.47
OMe
O
F
Ph
3a
2.07
2.05
5.39
1.00
3.00
1.04
3.00
10.0
9.5
9.0
8.5
8.0
7.5
7.0
6.5
6.0
5.5
5.0
4.5
4.0
3.5
3.0
2.5
2.0
1.5
1.0
0.5
0.0

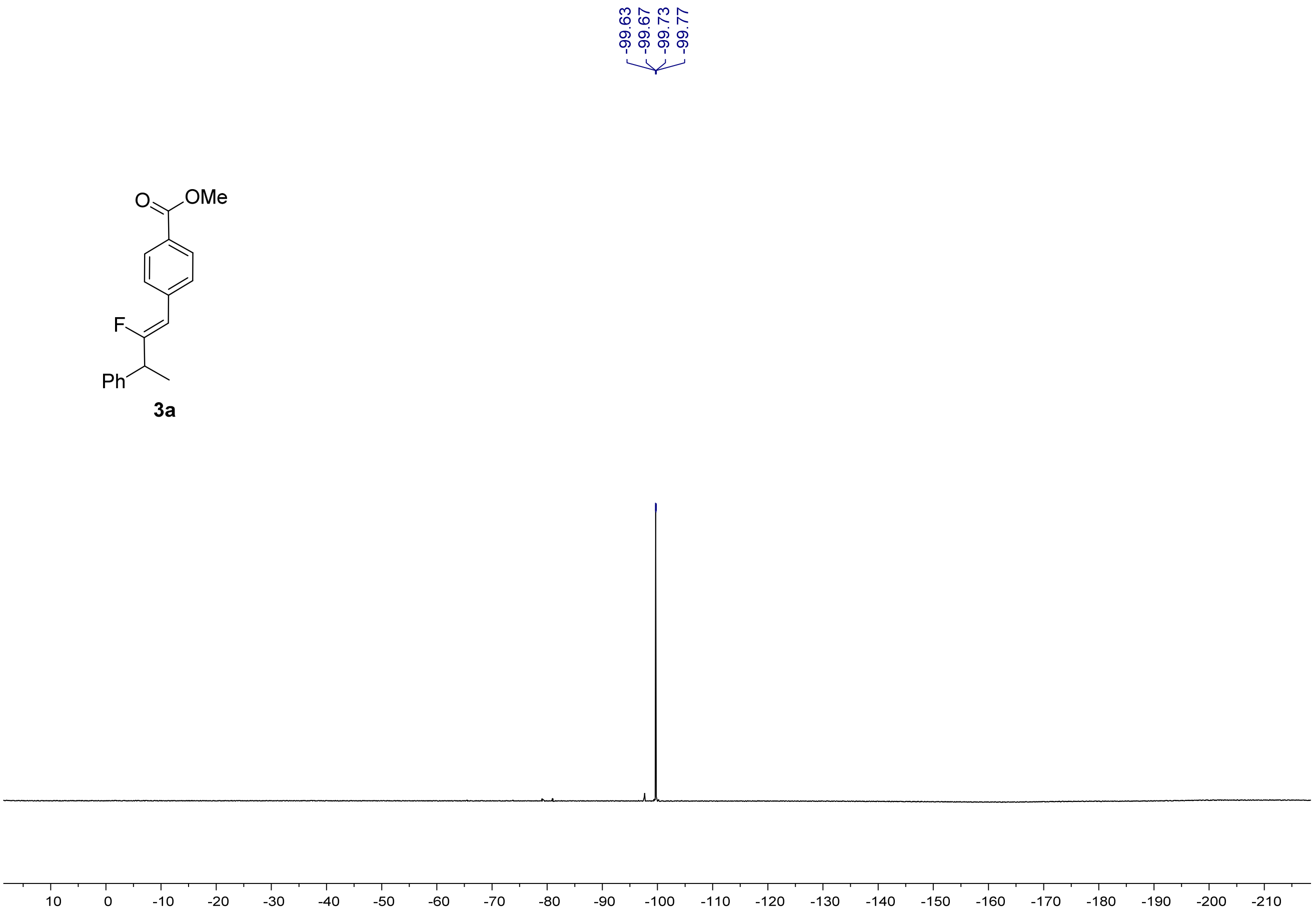
-99.63
-99.67
-99.73
-99.77
OMe
O
F
Ph
3a
10
0
-10
-20
-30
-40
-50
-60
-70
-80
-90
-100
-110
-120
-130
-140
-150
-160
-170
-180
-190
-200
-210

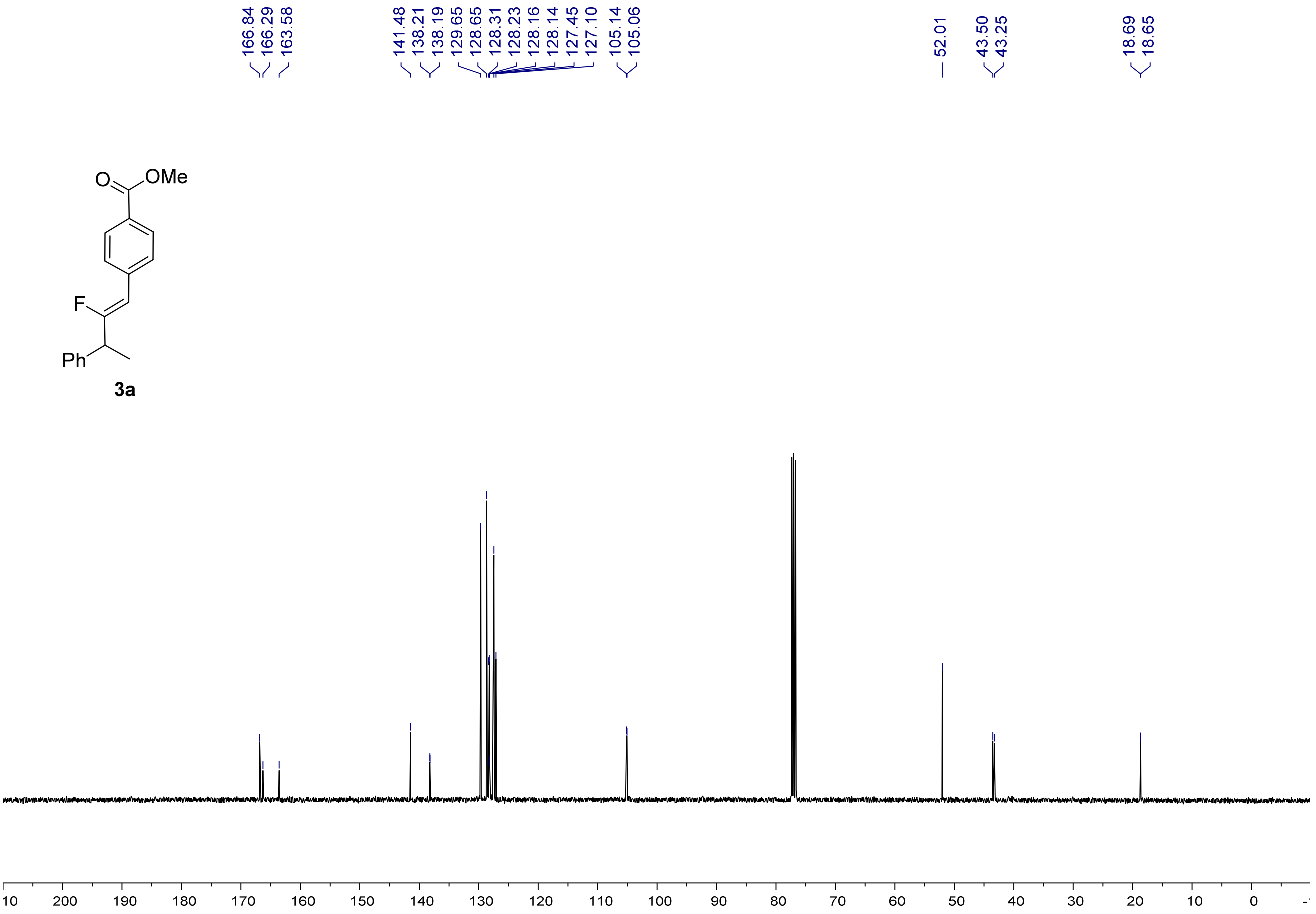
166.84
166.29
163.58
141.48
138.21
138.19
129.65
128.65
128.31
128.23
128.16
128.14
127.45
127.10
105.14
105.06
52.01
43.50
43.25
18.69
18.65
O
OMe
F
Ph
3a
10
200
190
180
170
160
150
140
130
120
110
100
90
80
70
60
50
40
30
20
10
0

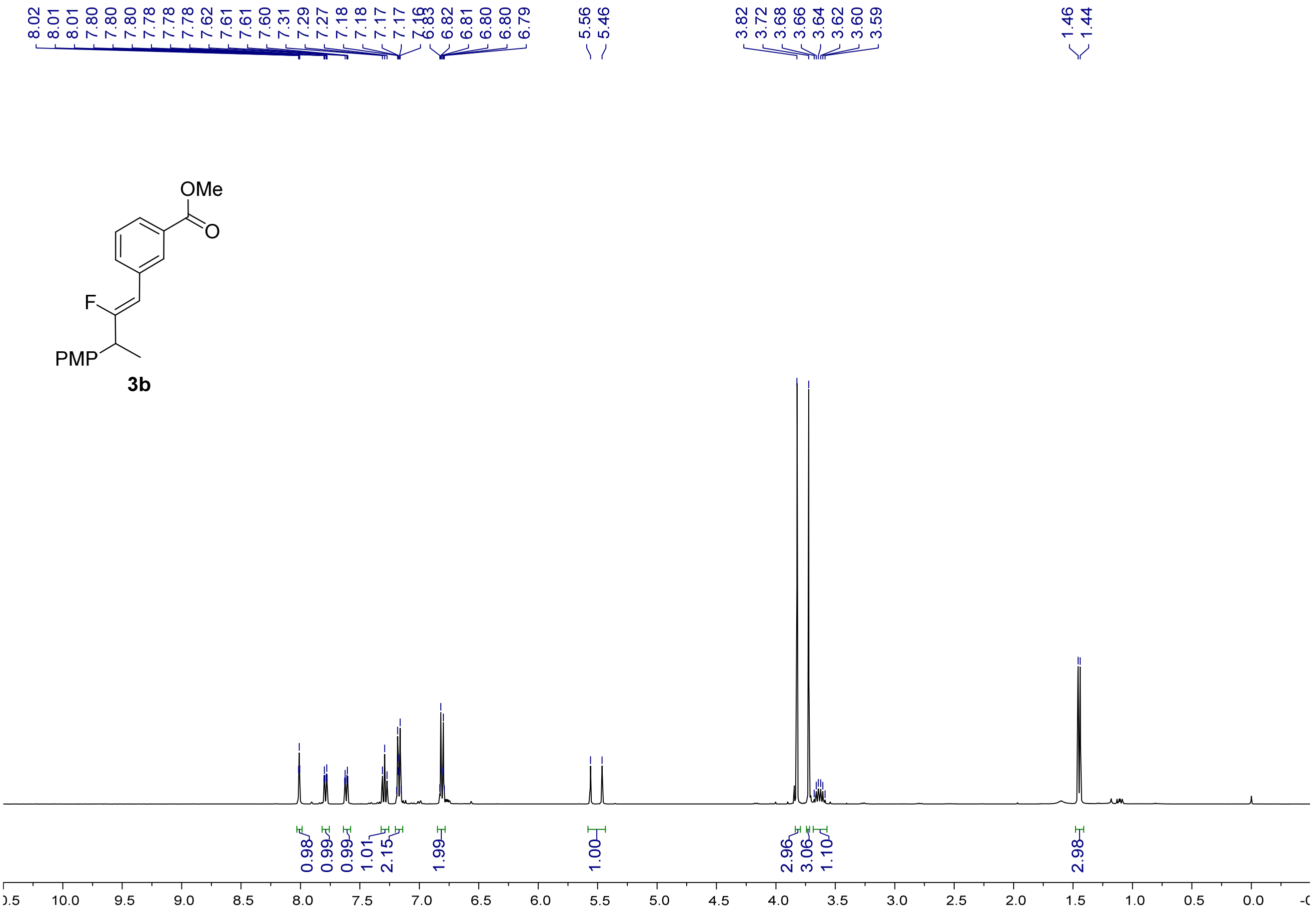
8.02
8.01
8.01
7.80
7.80
7.80
7.78
7.78
7.78
7.62
7.61
7.61
7.60
7.31
7.29
7.27
7.18
7.18
7.17
7.17
7.16
6.83
6.82
6.81
6.80
6.80
6.79
5.56
5.46
3.82
3.72
3.68
3.66
3.64
3.62
3.60
3.59
1.46
1.44
OMe
O
F
PMP
3b
0.98
0.99
0.99
1.01
2.15
1.99
1.00
2.96
3.06
1.10
2.98
10.0
9.5
9.0
8.5
8.0
7.5
7.0
6.5
6.0
5.5
5.0
4.5
4.0
3.5
3.0
2.5
2.0
1.5
1.0
0.5
0.0

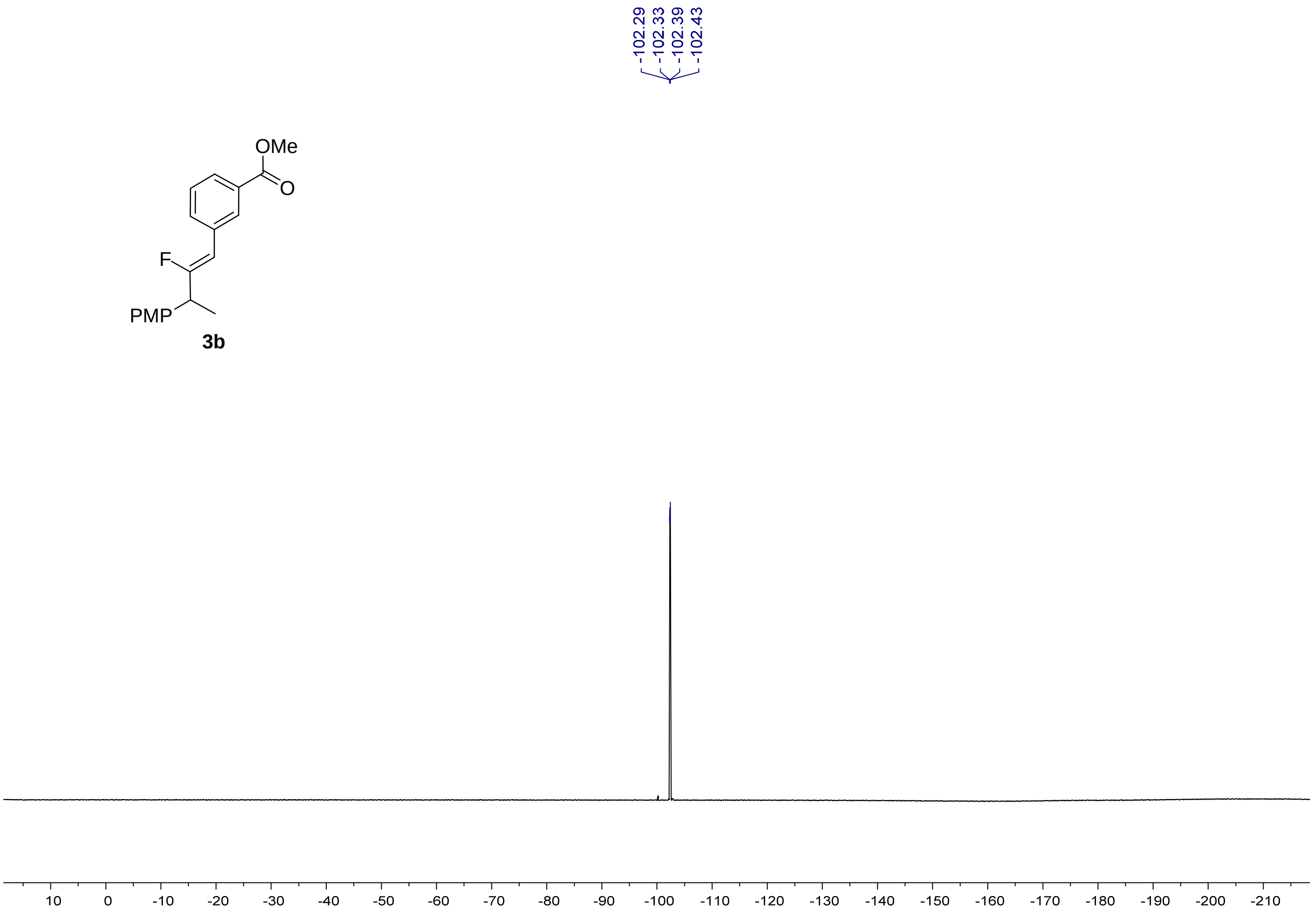
-102.29
-102.33
-102.39
-102.43
OMe
O
F
PMP
3b
10 0 -10 -20 -30 -40 -50 -60 -70 -80 -90 -100 -110 -120 -130 -140 -150 -160 -170 -180 -190 -200 -210

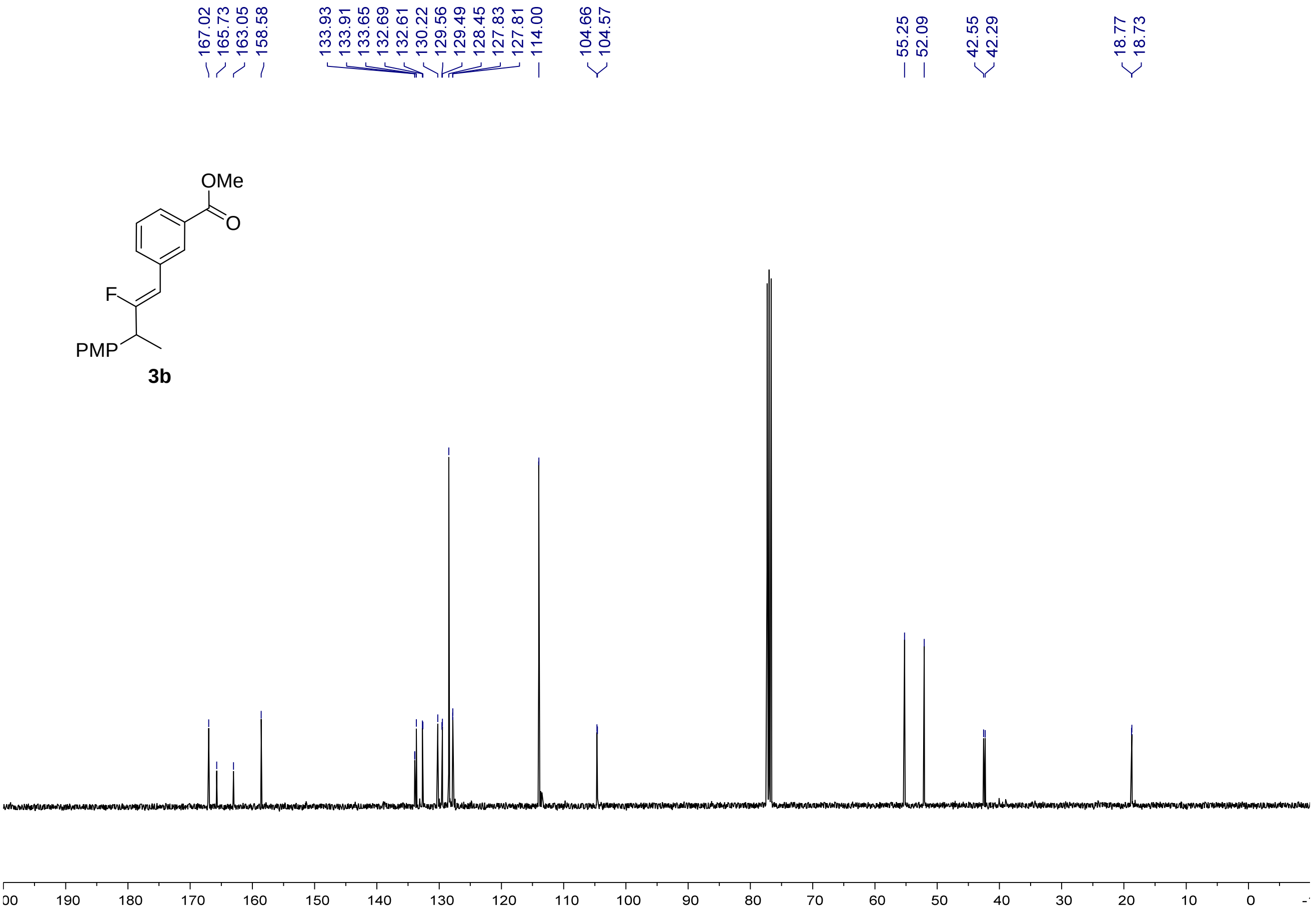
167.02
165.73
163.05
158.58
133.93
133.91
133.65
132.69
132.61
130.22
129.56
129.49
128.45
127.83
127.81
114.00
104.66
104.57
55.25
52.09
42.55
42.29
18.77
18.73
OMe
O
F
PMP
3b
190 180 170 160 150 140 130 120 110 100 90 80 70 60 50 40 30 20 10 0

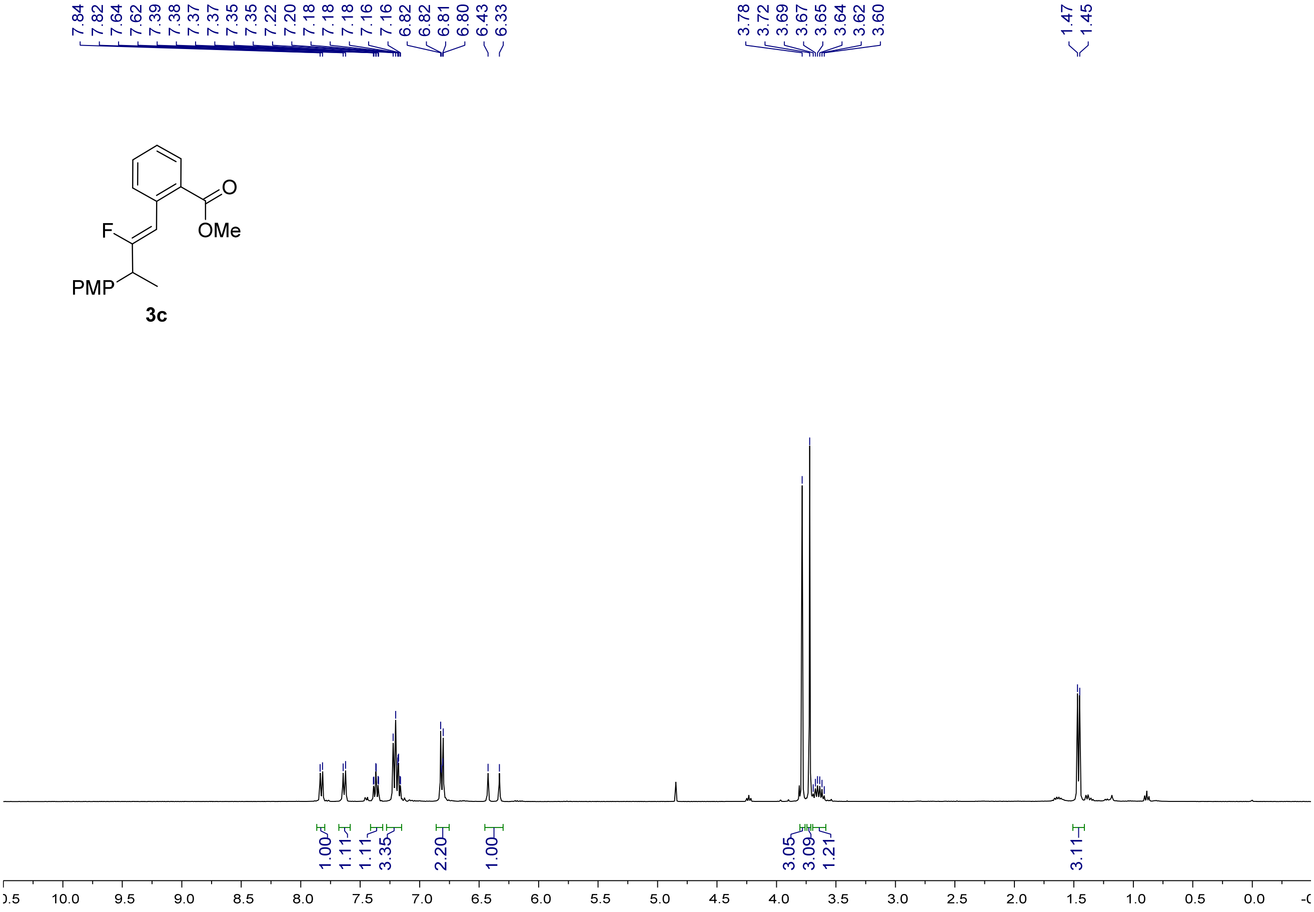
7.84
7.82
7.64
7.62
7.39
7.38
7.37
7.37
7.35
7.35
7.22
7.20
7.18
7.18
7.18
7.16
7.16
6.82
6.82
6.81
6.80
6.43
6.33
3.78
3.72
3.69
3.67
3.65
3.64
3.62
3.60
1.47
1.45
O
OMe
F
PMP
1.00
1.11
1.11
3.35
2.20
1.00
3.05
3.09
1.21
3.11

3c

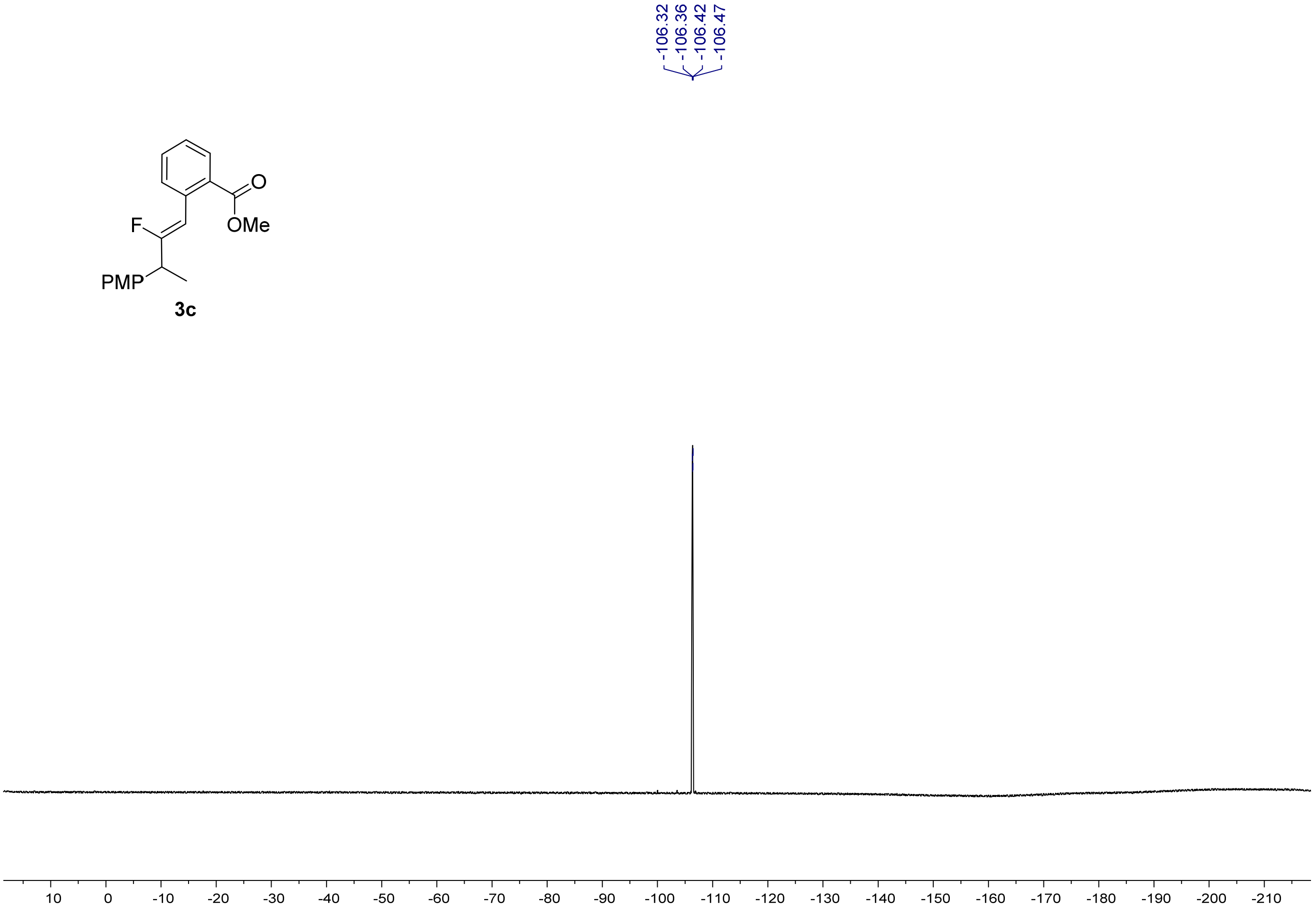
-106.32
-106.36
-106.42
-106.47
O
OMe
F
PMP

3c

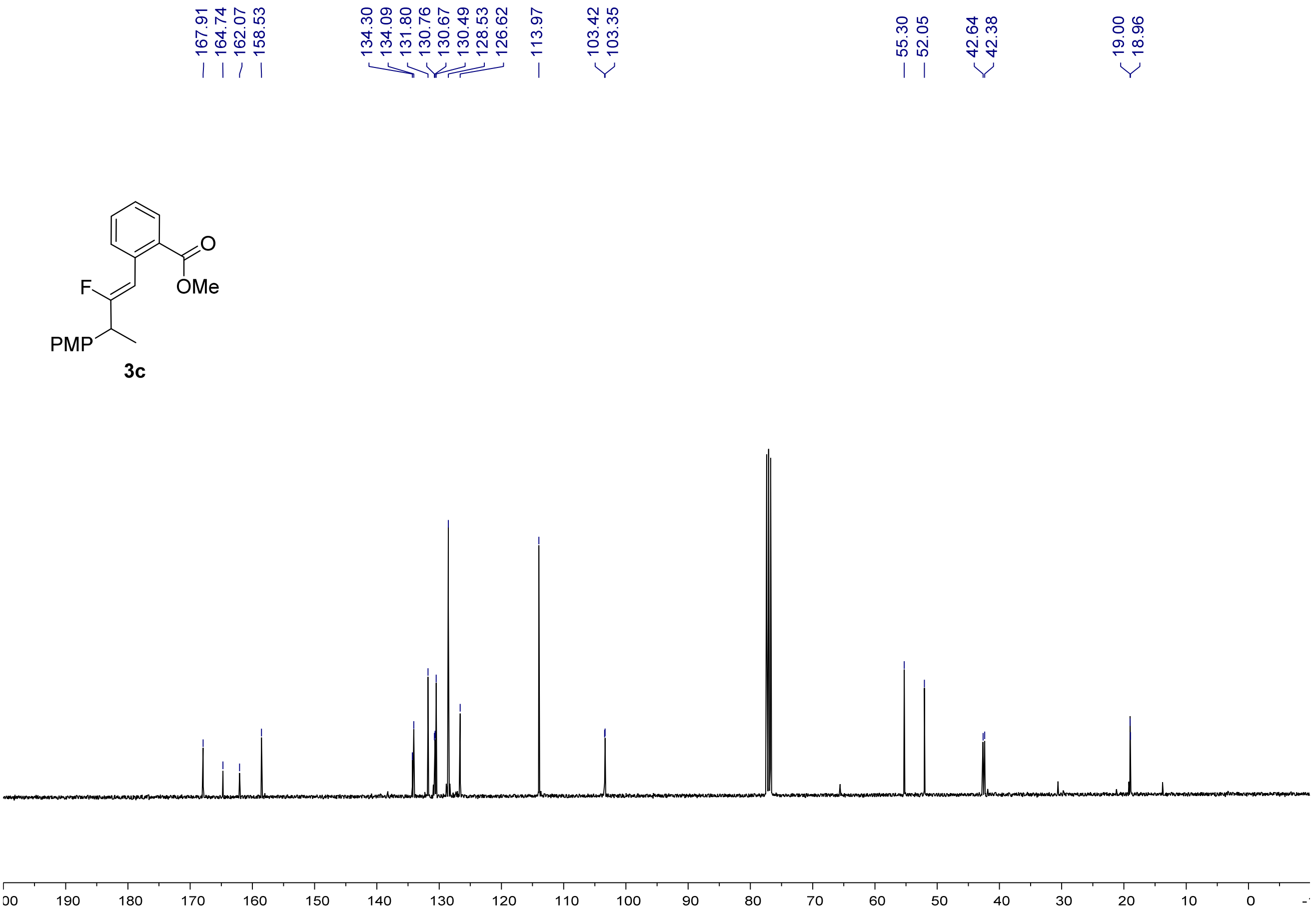

167.91
164.74
162.07
158.53
134.30
134.09
131.80
130.76
130.67
130.49
128.53
126.62
113.97
103.42
103.35
55.30
52.05
42.64
42.38
19.00
18.96
F
OMe
O
PMP
3c

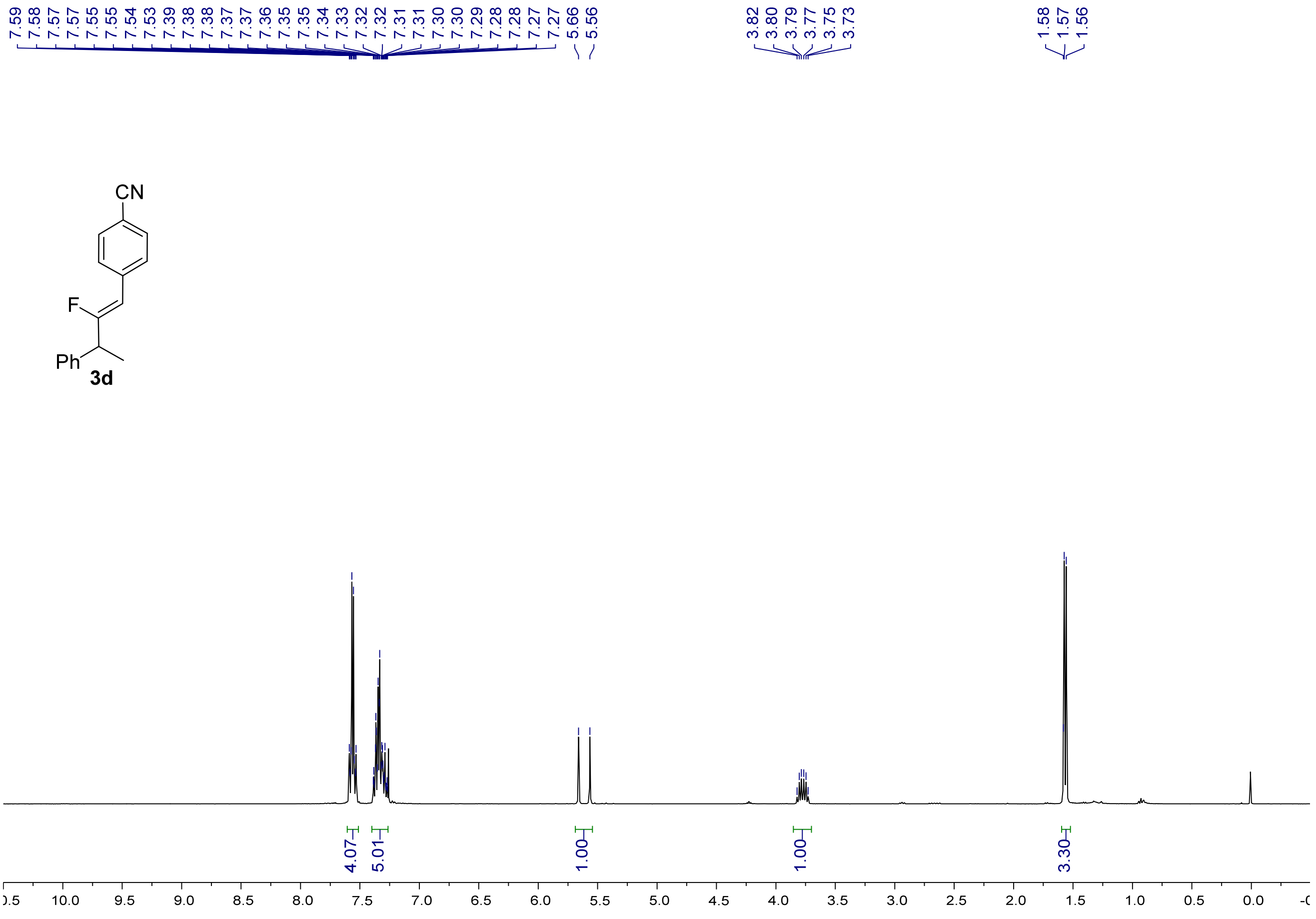

7.59
7.58
7.57
7.57
7.55
7.55
7.54
7.53
7.39
7.38
7.38
7.37
7.37
7.36
7.35
7.35
7.34
7.33
7.32
7.32
7.31
7.30
7.30
7.29
7.28
7.28
7.27
7.27
5.66
5.56
3.82
3.80
3.79
3.77
3.75
3.73
1.58
1.57
1.56
CN
F
Ph
3d
4.07
5.01
1.00
1.00
3.30

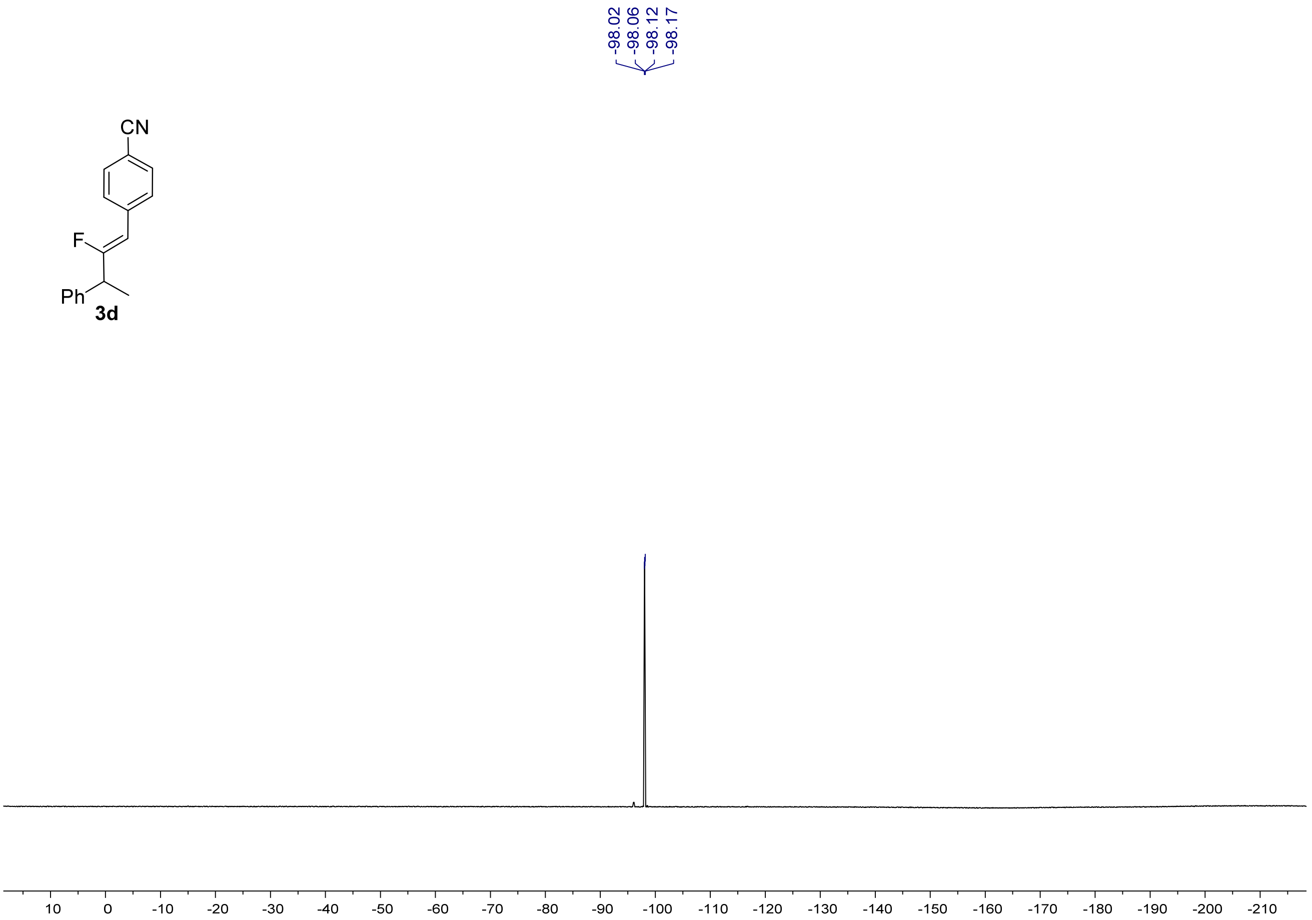

-98.02
-98.06
-98.12
-98.17
CN
F
Ph
3d
10 0 -10 -20 -30 -40 -50 -60 -70 -80 -90 -100 -110 -120 -130 -140 -150 -160 -170 -180 -190 -200 -210

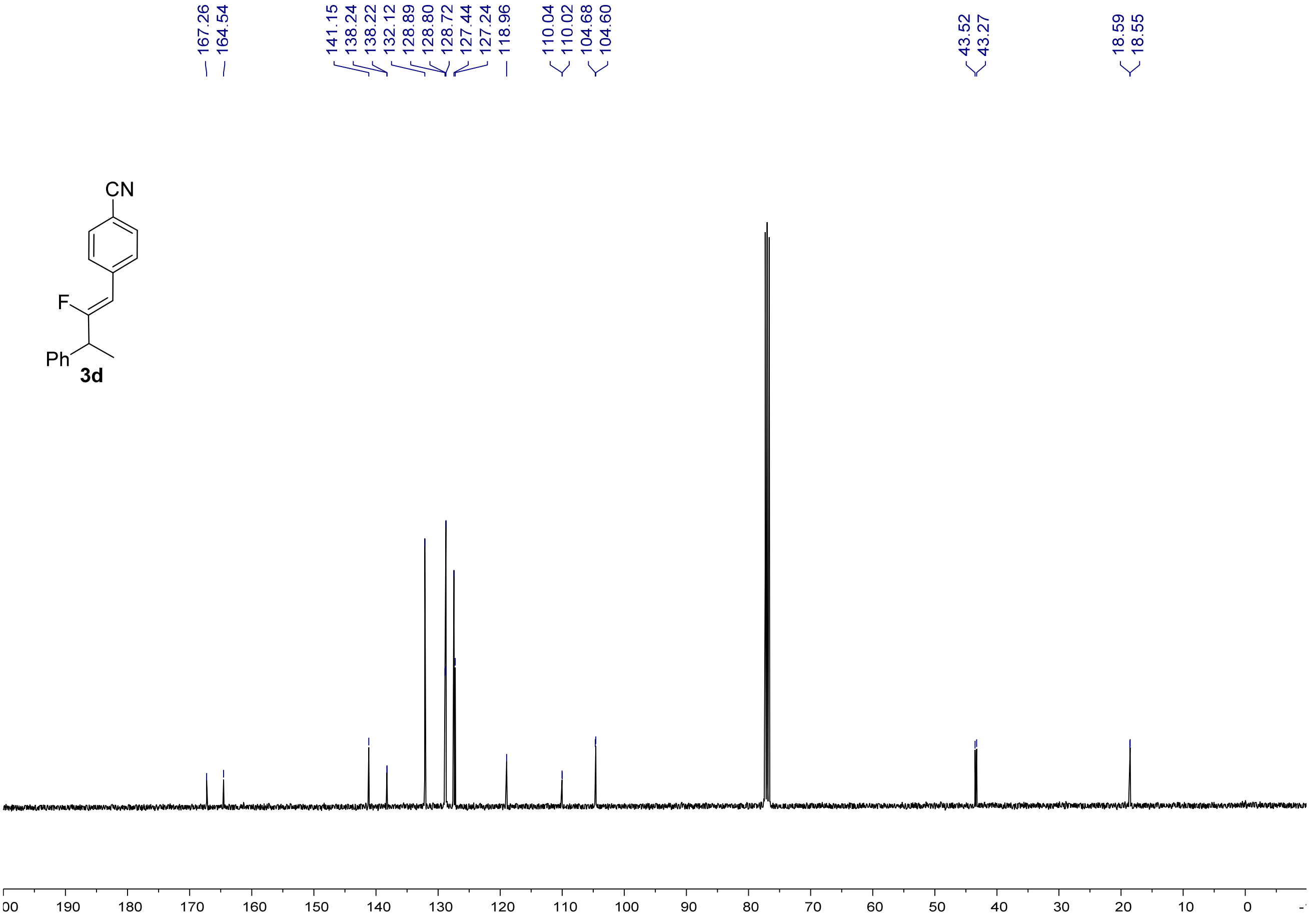

167.26
164.54
141.15
138.24
138.22
132.12
128.89
128.80
128.72
127.44
127.24
118.96
110.04
110.02
104.68
104.60
43.52
43.27
18.59
18.55
CN
F
Ph
3d
190 180 170 160 150 140 130 120 110 100 90 80 70 60 50 40 30 20 10 0

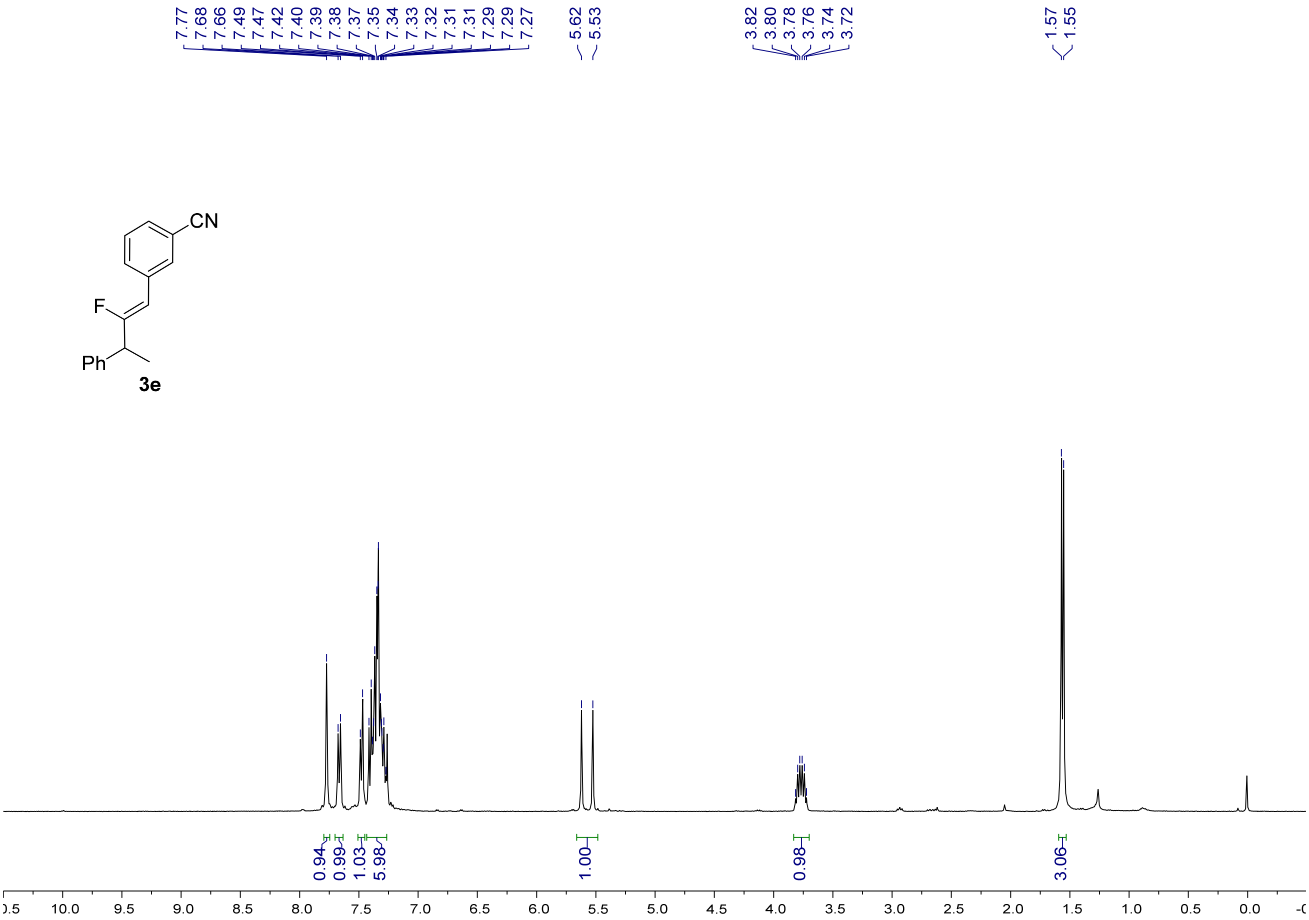
7.77 7.68 7.66 7.49 7.47 7.42 7.40 7.39 7.38 7.37 7.35 7.34 7.33 7.32 7.31 7.31 7.29 7.29 7.27
5.62 5.53
3.82 3.80 3.78 3.76 3.74 3.72
1.57 1.55
CN
F
Ph
3e
0.94 0.99 1.03 5.98
1.00
0.98
3.06
10.0 9.5 9.0 8.5 8.0 7.5 7.0 6.5 6.0 5.5 5.0 4.5 4.0 3.5 3.0 2.5 2.0 1.5 1.0 0.5 0.0

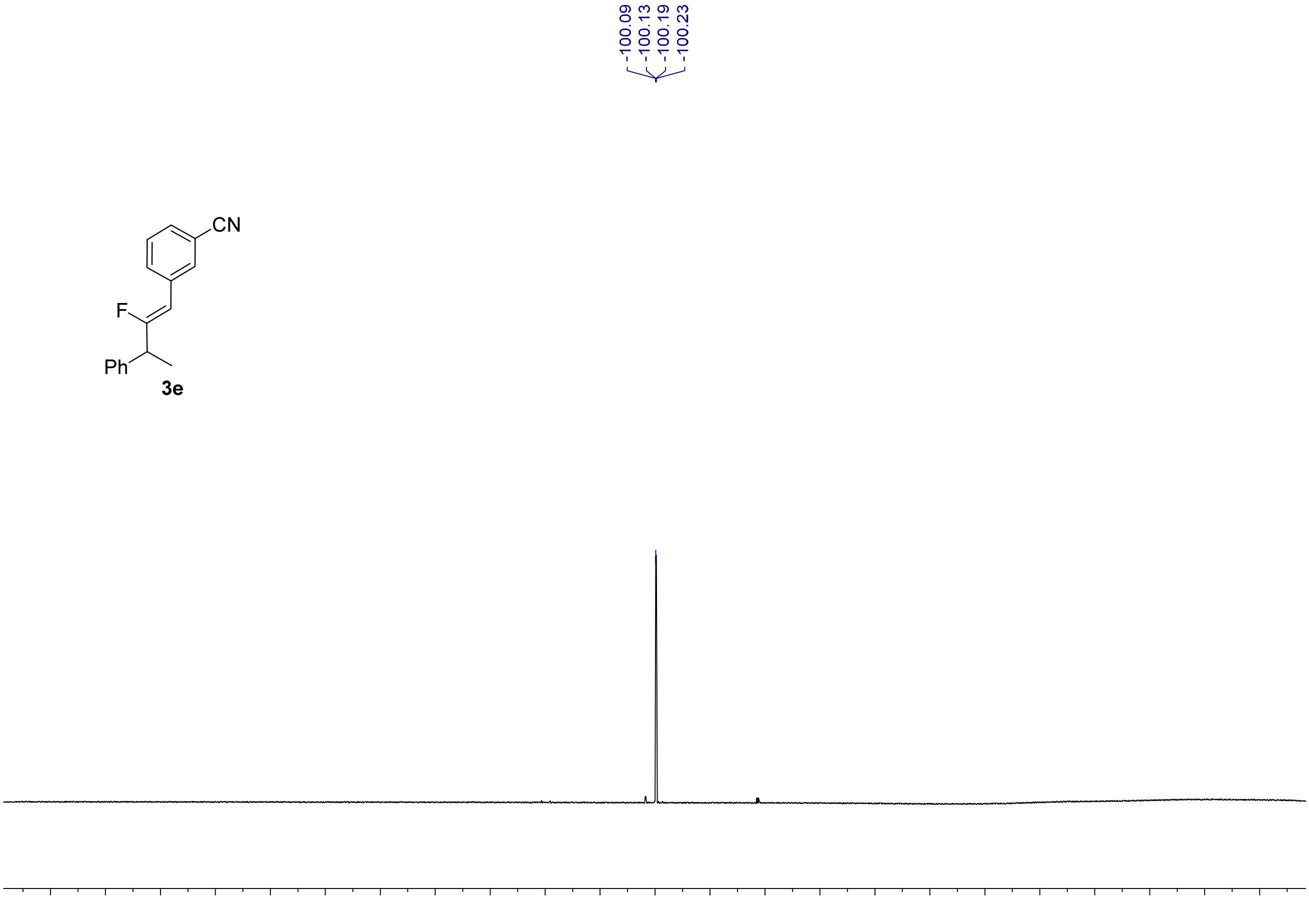
-100.09 -100.13 -100.19 -100.23
CN
F
Ph
3e
10 0 -10 -20 -30 -40 -50 -60 -70 -80 -90 -100 -110 -120 -130 -140 -150 -160 -170 -180 -190 -200 -210

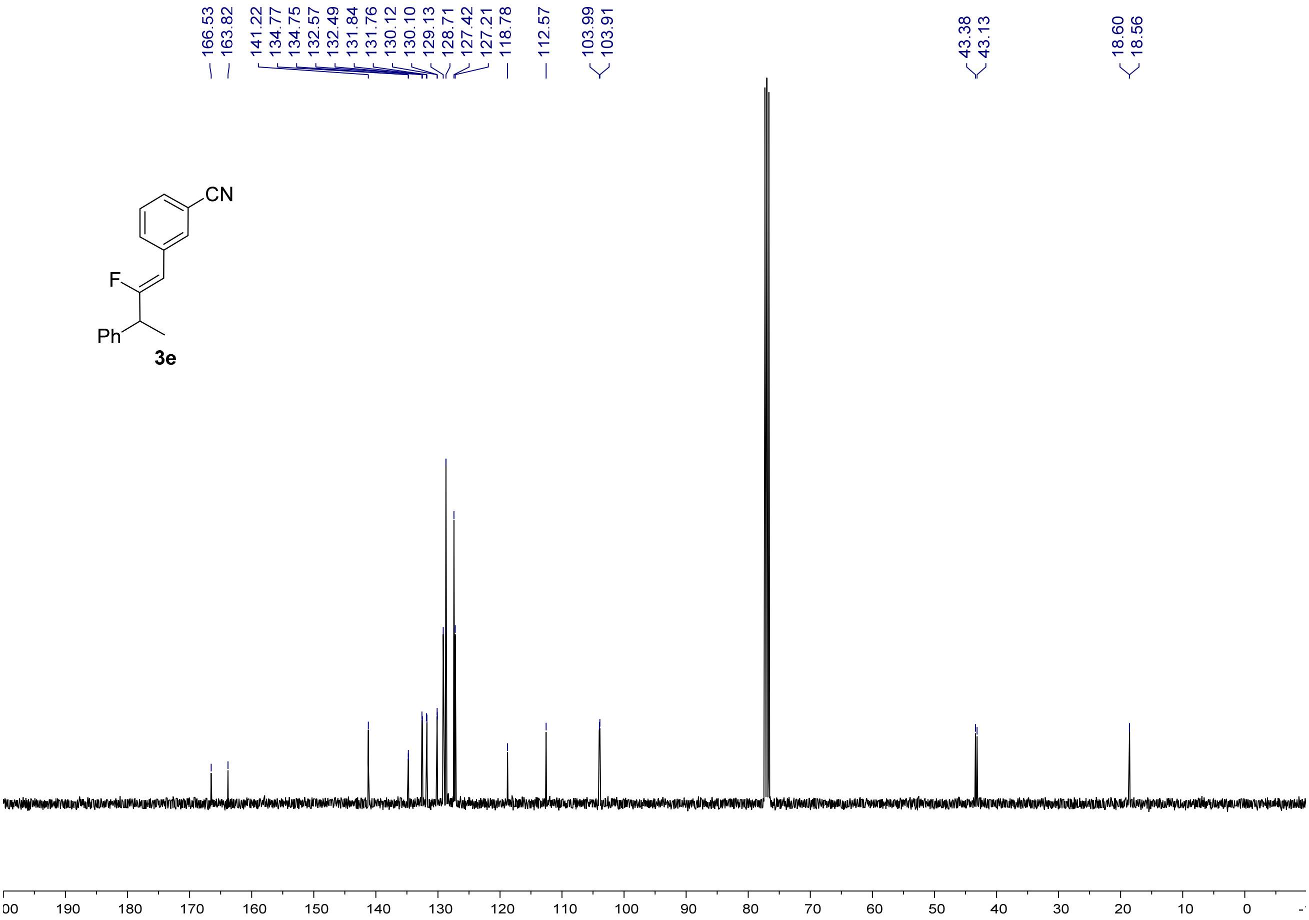

166.53
163.82
141.22
134.77
134.75
132.57
132.49
131.84
131.76
130.12
130.10
129.13
128.71
127.42
127.21
118.78
112.57
103.99
103.91
43.38
43.13
18.60
18.56
CN
F
Ph
3e

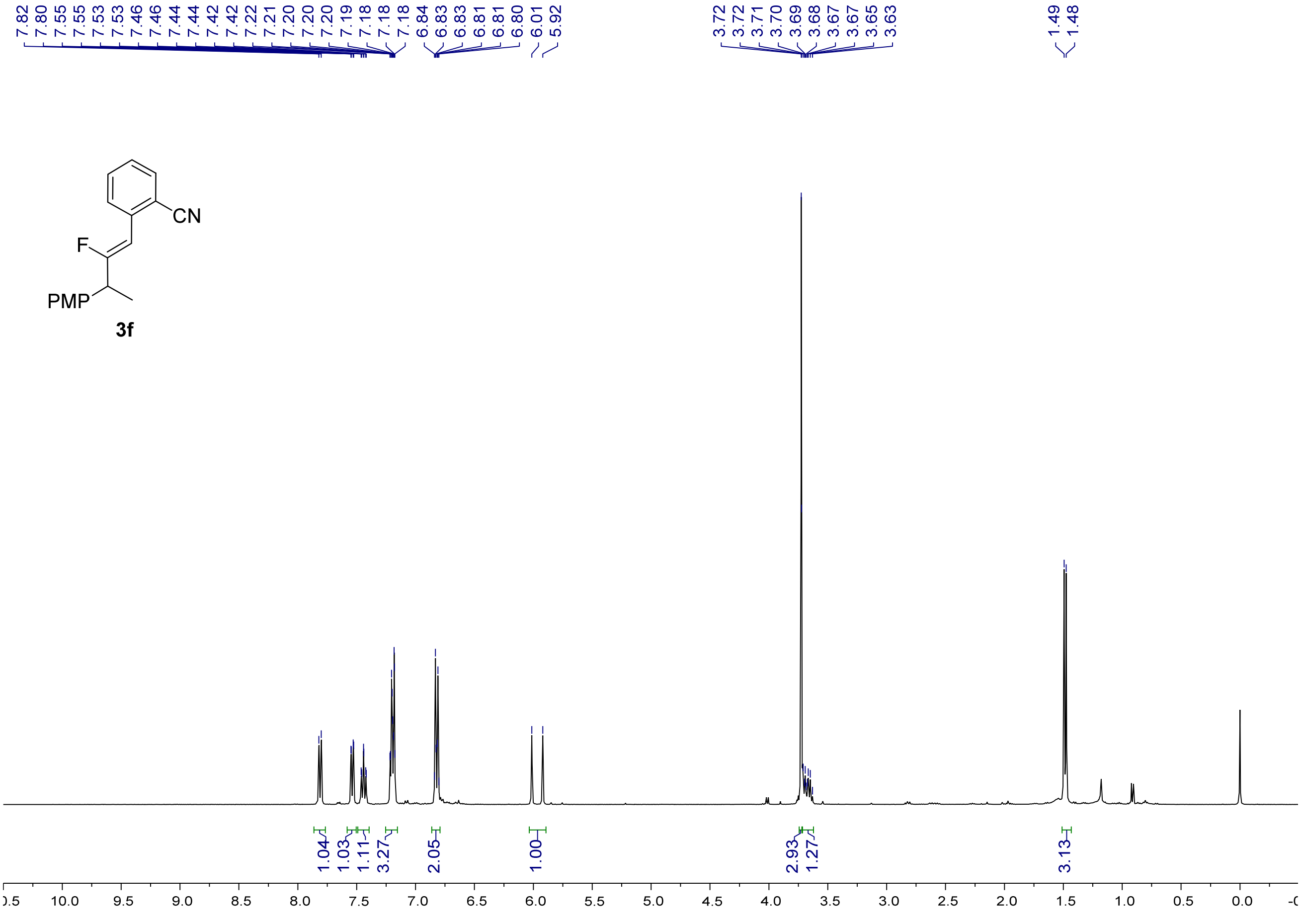

7.82
7.80
7.55
7.55
7.53
7.46
7.46
7.44
7.44
7.42
7.42
7.22
7.21
7.20
7.20
7.19
7.18
7.18
7.18
6.84
6.83
6.83
6.81
6.81
6.80
6.01
5.92
3.72
3.72
3.71
3.70
3.69
3.68
3.67
3.65
3.63
1.49
1.48
CN
F
PMP
3f
1.04
1.03
1.11
3.27
2.05
1.00
2.93
1.27
3.13

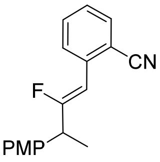

3f

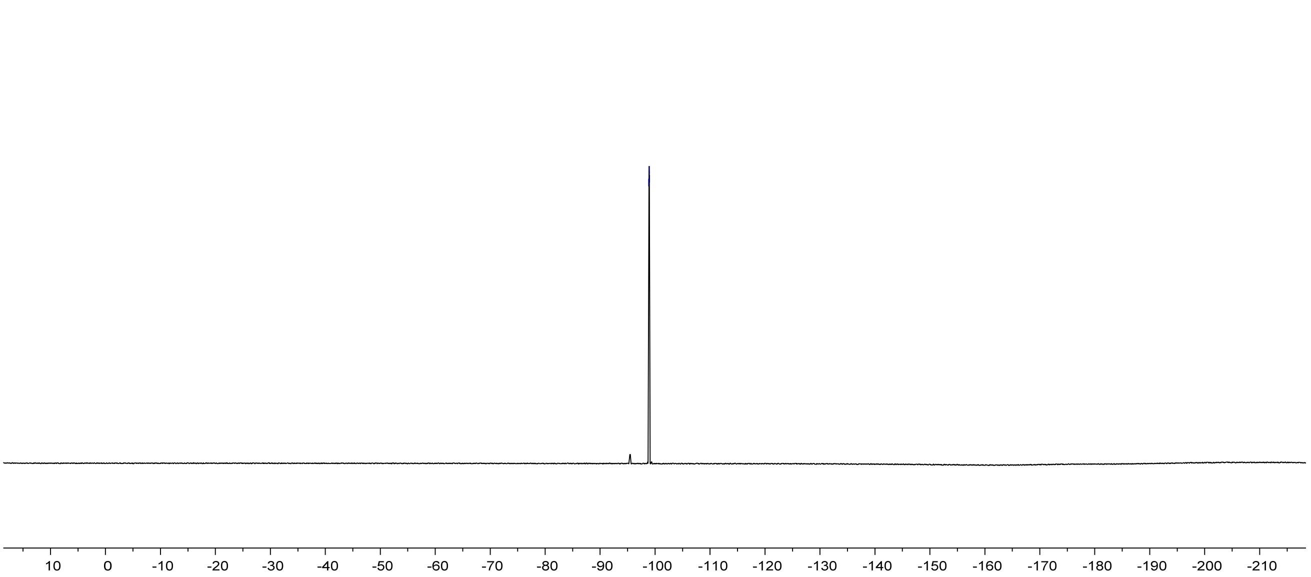
-98.84
-98.89
-98.94
-98.99
10 0 -10 -20 -30 -40 -50 -60 -70 -80 -90 -100 -110 -120 -130 -140 -150 -160 -170 -180 -190 -200 -210

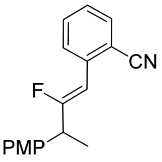

3f

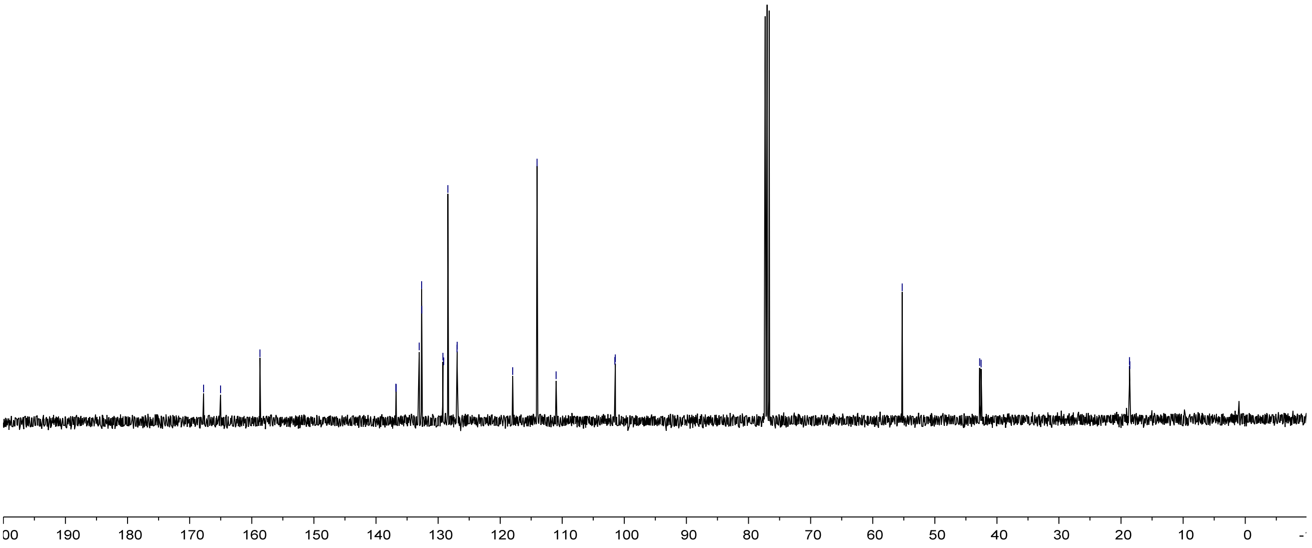
167.77
165.04
158.68
136.79
136.75
133.04
132.65
132.62
129.22
129.08
128.41
126.94
126.92
117.98
114.05
110.99
101.54
101.46
55.24
42.78
42.53
18.63
18.59
190 180 170 160 150 140 130 120 110 100 90 80 70 60 50 40 30 20 10 0

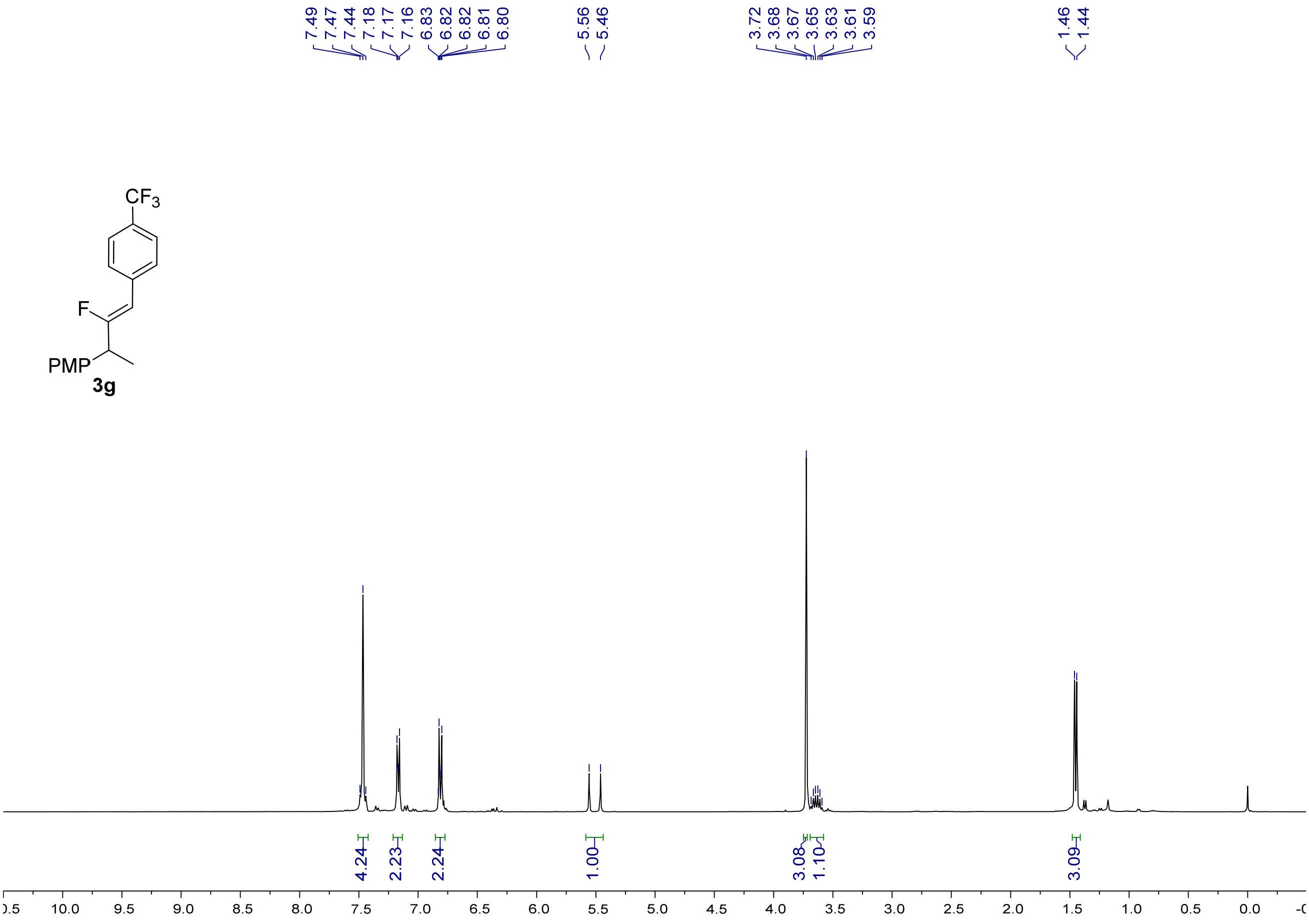
7.49
7.47
7.44
7.18
7.17
7.16
6.83
6.82
6.82
6.81
6.80
5.56
5.46
3.72
3.68
3.67
3.65
3.63
3.61
3.59
1.46
1.44
CF3
F
PMP
3g
4.24
2.23
2.24
1.00
3.08
1.10
3.09

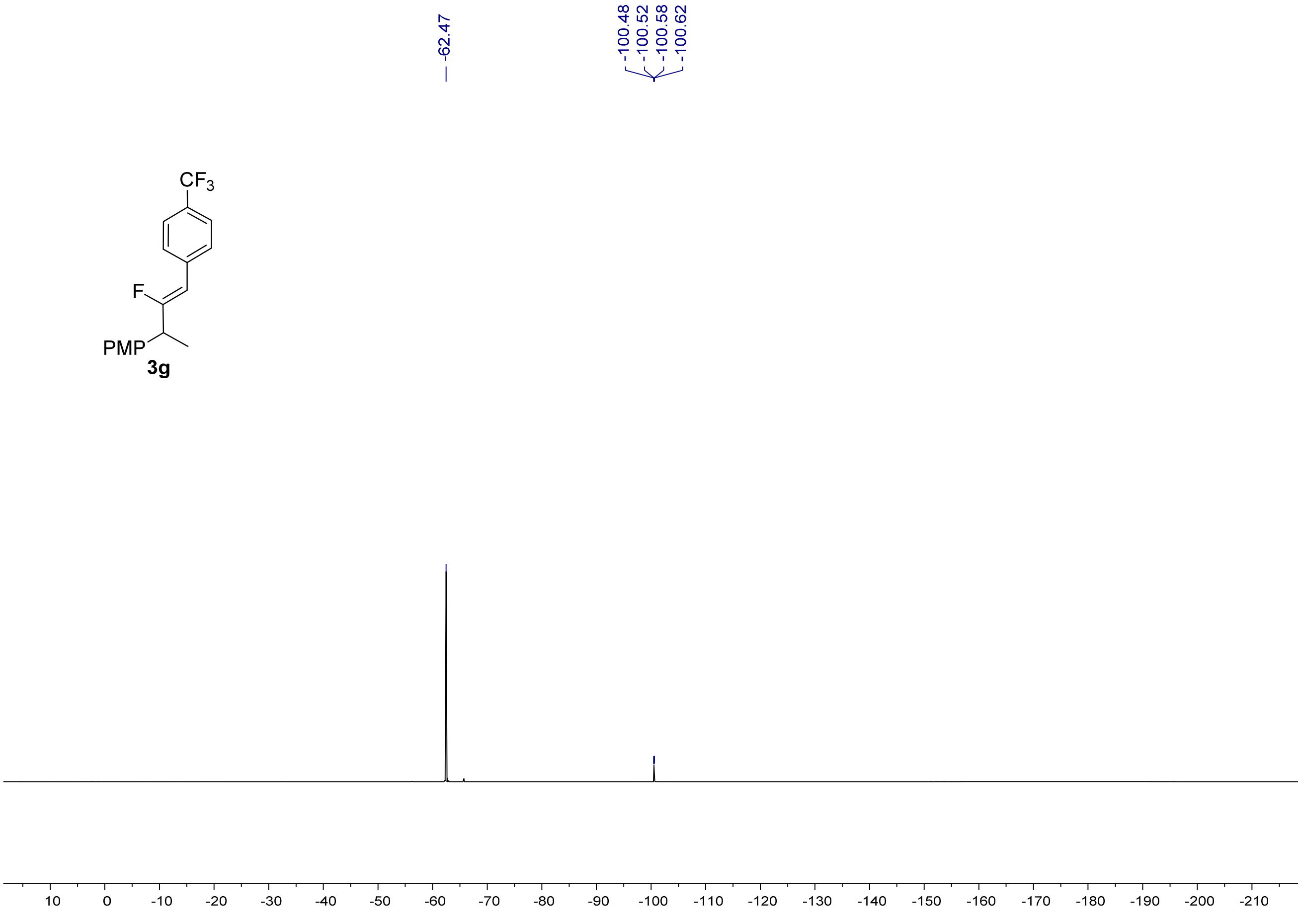
-62.47
-100.48
-100.52
-100.58
-100.62
CF3
F
PMP
3g

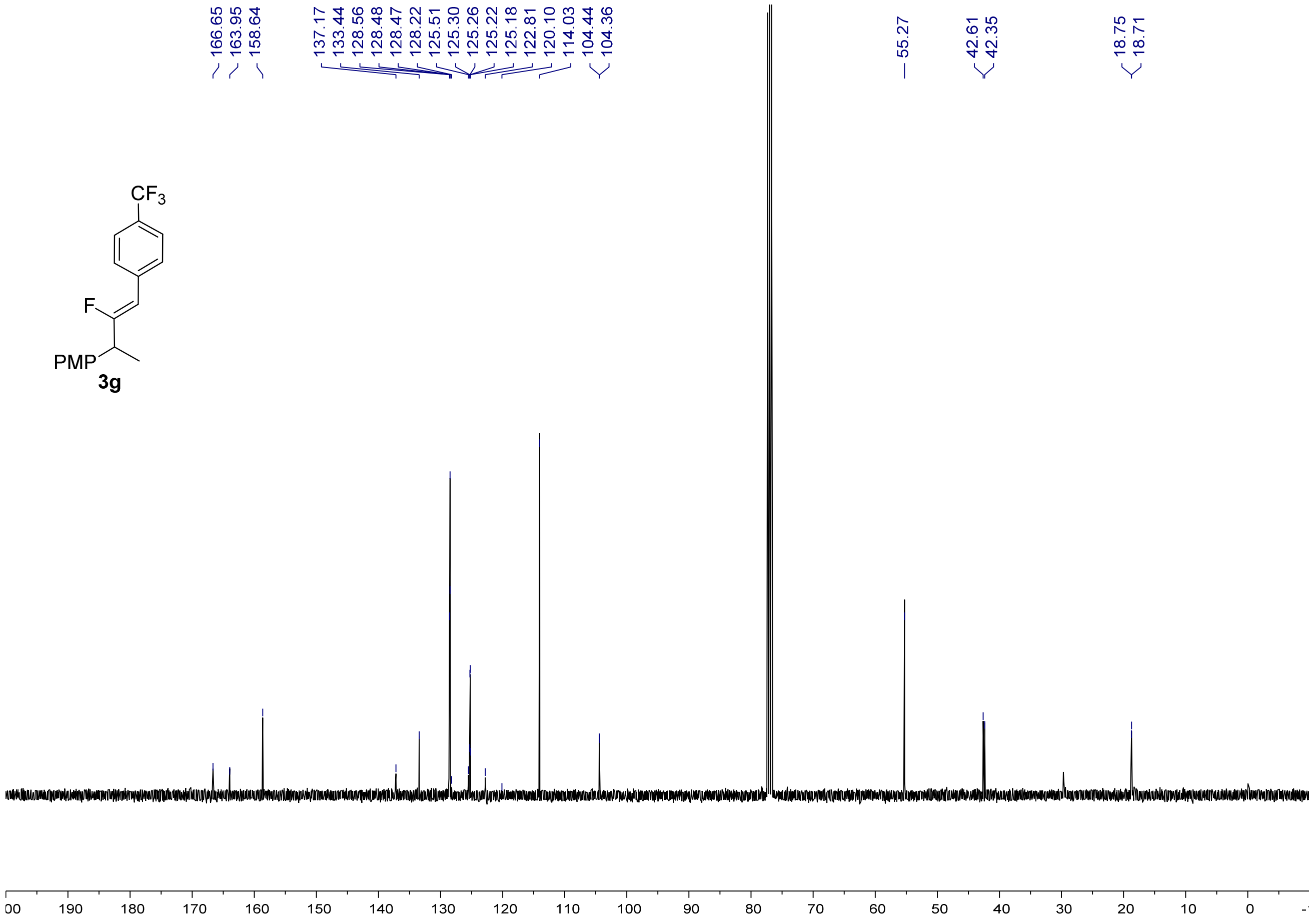
166.65
163.95
158.64
137.17
133.44
128.56
128.48
128.47
128.22
125.51
125.30
125.26
125.22
125.18
122.81
120.10
114.03
104.44
104.36
55.27
42.61
42.35
18.75
18.71
CF3
F
PMP
3g

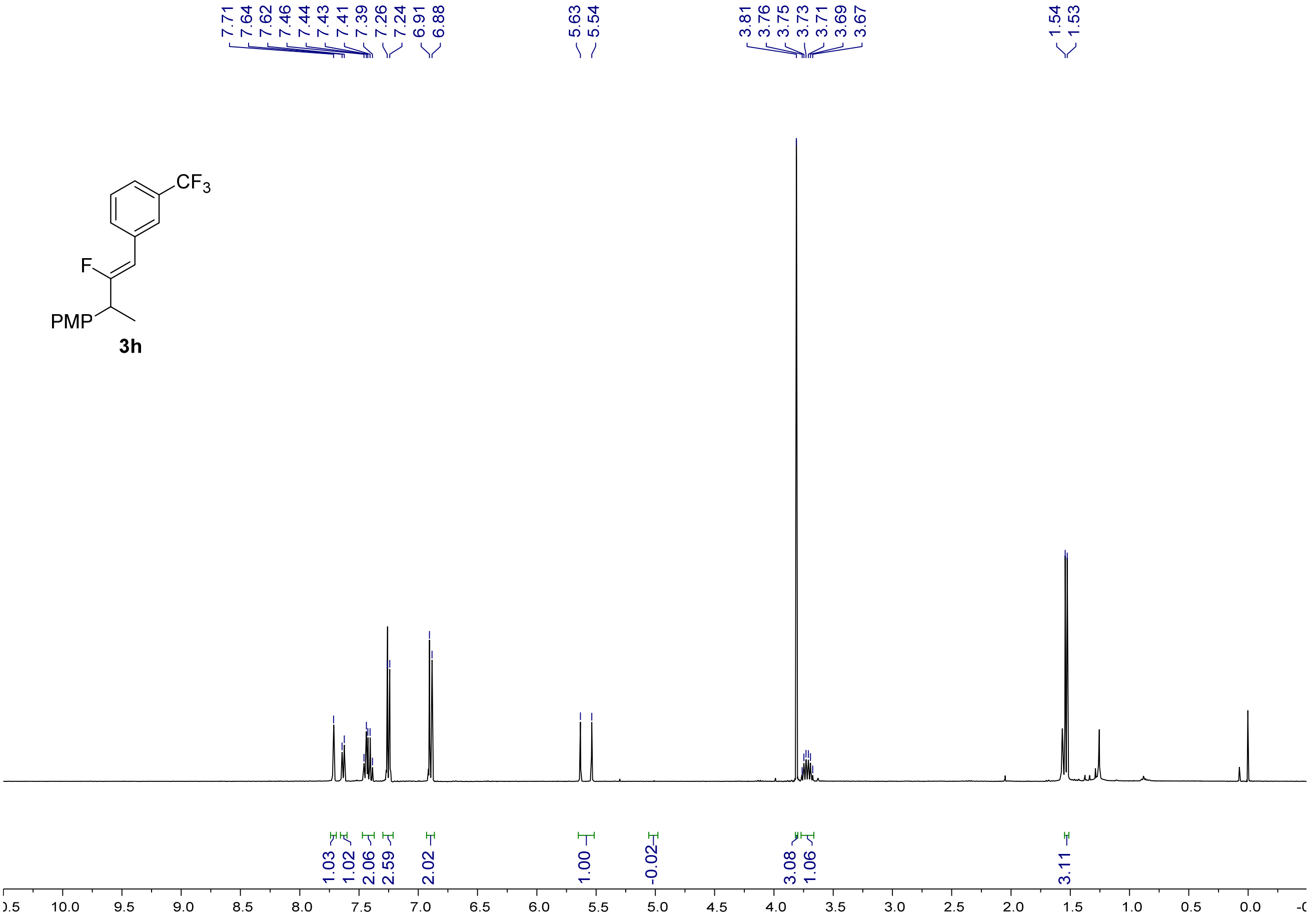
7.71
7.64
7.62
7.46
7.44
7.43
7.41
7.39
7.26
7.24
6.91
6.88
5.63
5.54
3.81
3.76
3.75
3.73
3.71
3.69
3.67
1.54
1.53
CF3
F
PMP
3h
1.03
1.02
2.06
2.59
2.02
1.00
-0.02
3.08
1.06
3.11

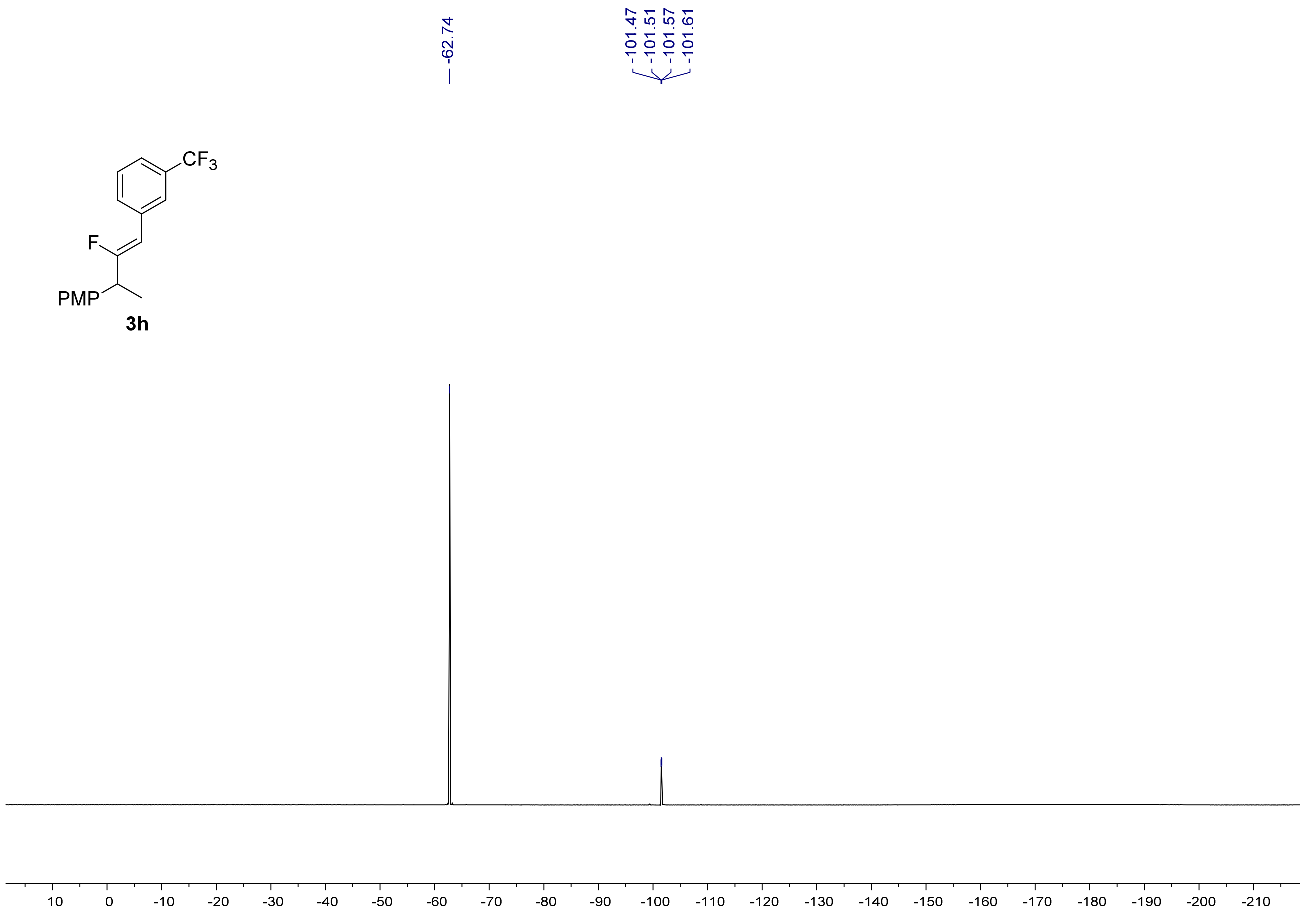
-62.74
-101.47
-101.51
-101.57
-101.61
CF3
F
PMP
3h
10 0 -10 -20 -30 -40 -50 -60 -70 -80 -90 -100 -110 -120 -130 -140 -150 -160 -170 -180 -190 -200 -210

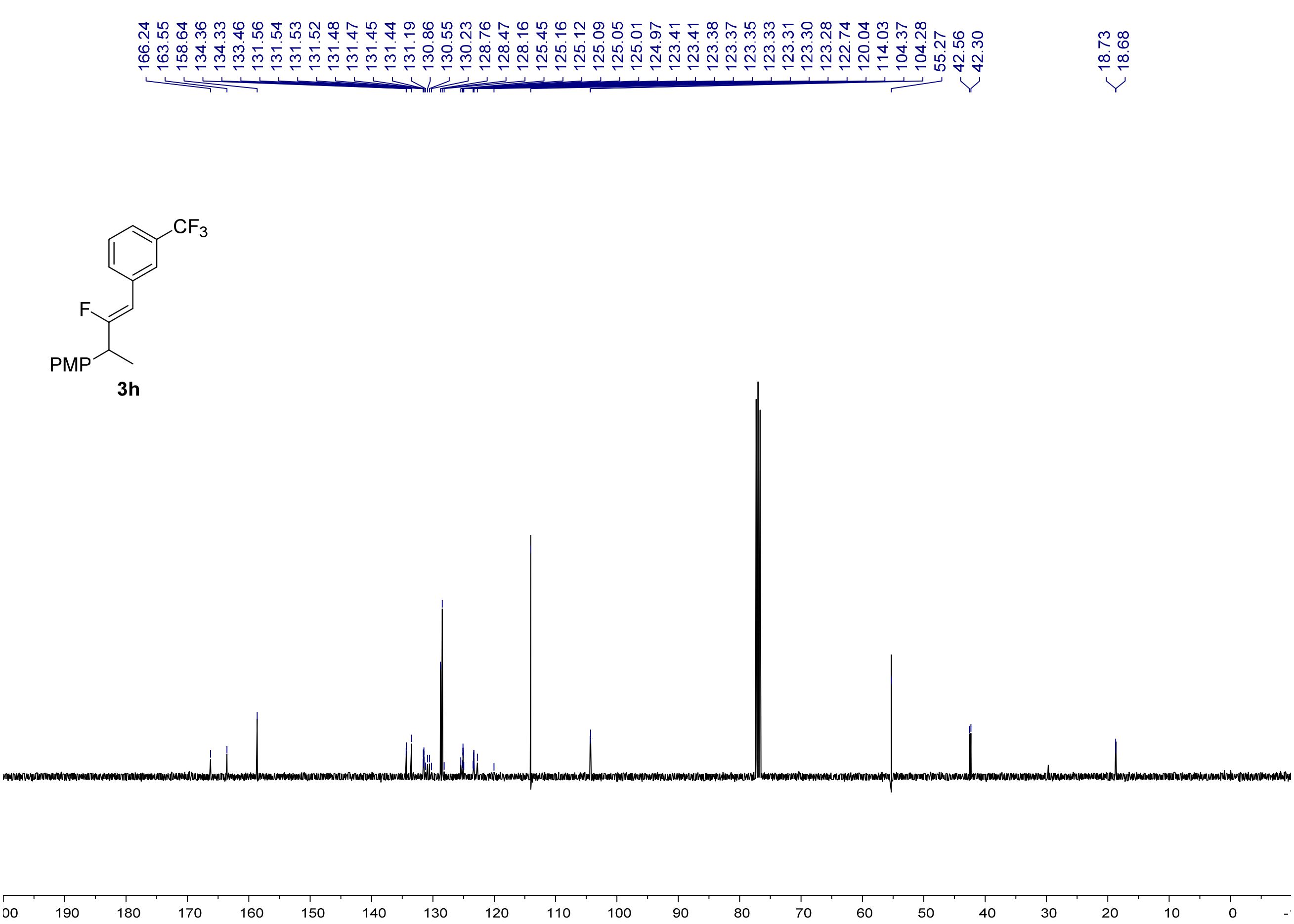
166.24
163.55
158.64
134.36
134.33
133.46
131.56
131.54
131.53
131.52
131.48
131.47
131.45
131.44
131.19
130.86
130.55
130.23
128.76
128.47
128.16
125.45
125.16
125.12
125.09
125.05
125.01
124.97
123.41
123.41
123.38
123.37
123.35
123.33
123.31
123.30
123.28
122.74
120.04
114.03
104.37
104.28
55.27
42.56
42.30
18.73
18.68
CF3
F
PMP
3h
190 180 170 160 150 140 130 120 110 100 90 80 70 60 50 40 30 20 10 0

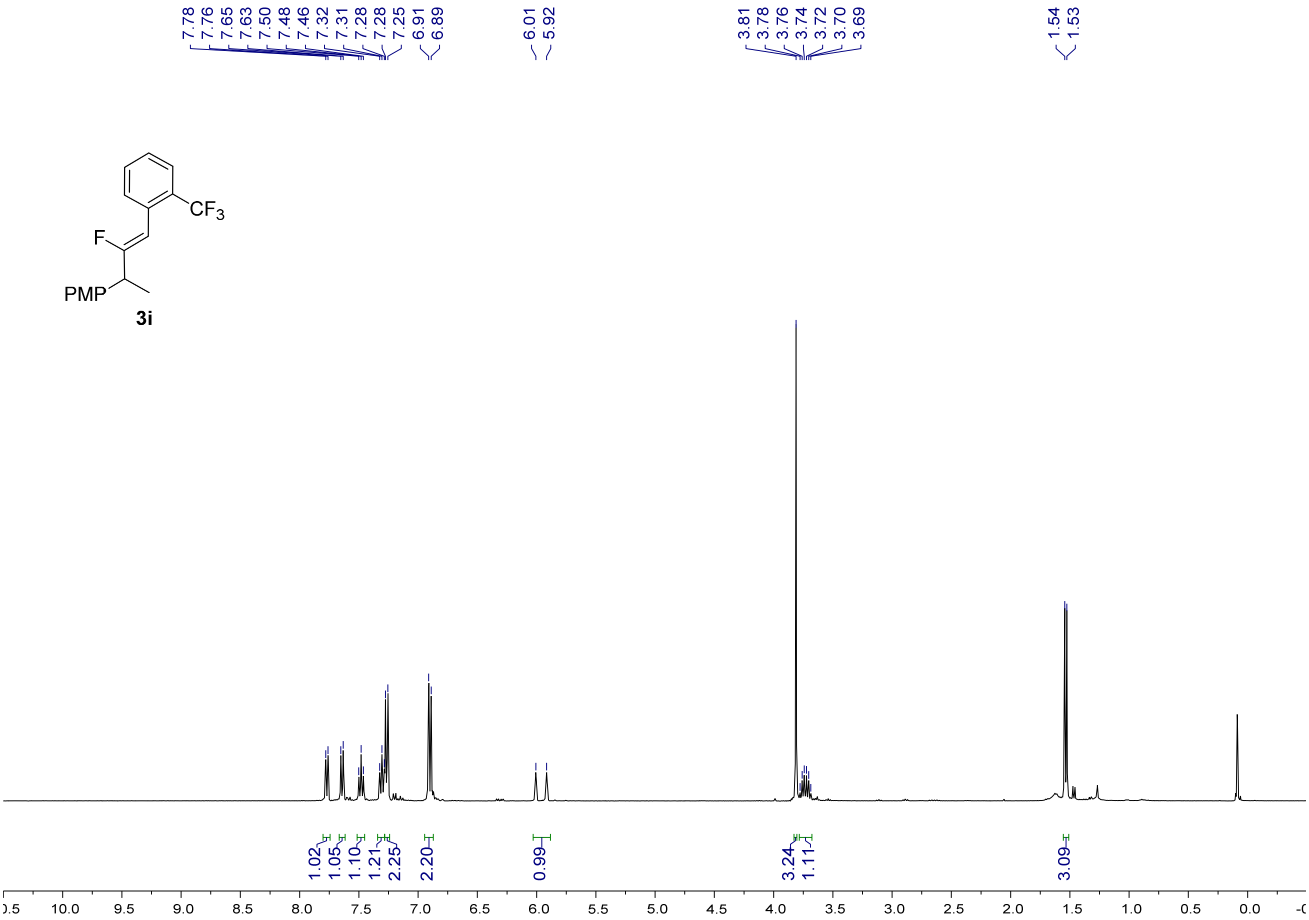
7.78
7.76
7.65
7.63
7.50
7.48
7.46
7.32
7.31
7.28
7.28
7.25
6.91
6.89
6.01
5.92
3.81
3.78
3.76
3.74
3.72
3.70
3.69
1.54
1.53
CF3
F
PMP
3i
1.02
1.05
1.10
1.21
2.25
2.20
0.99
3.24
1.11
3.09

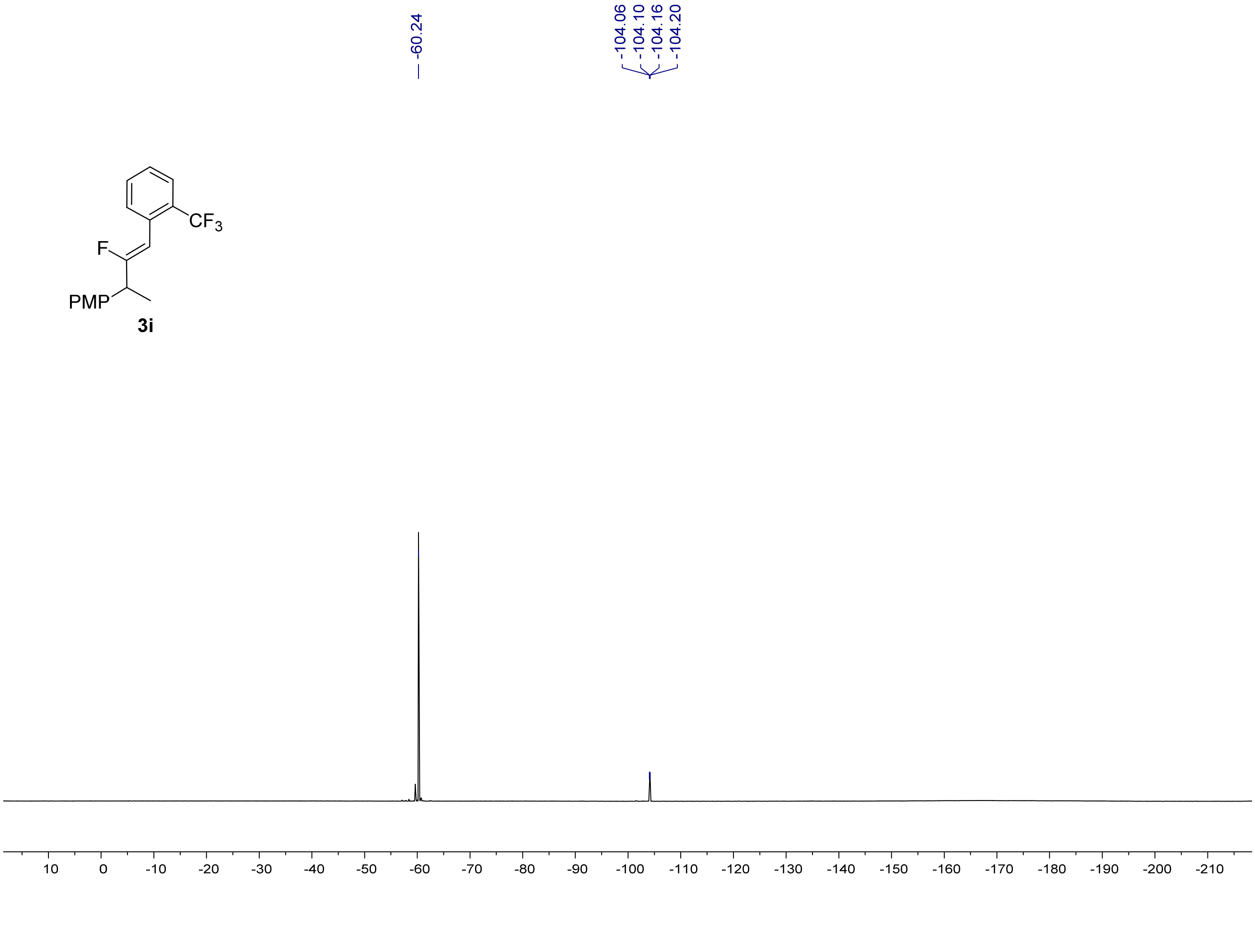
-60.24
-104.06
-104.10
-104.16
-104.20
CF3
F
PMP
3i

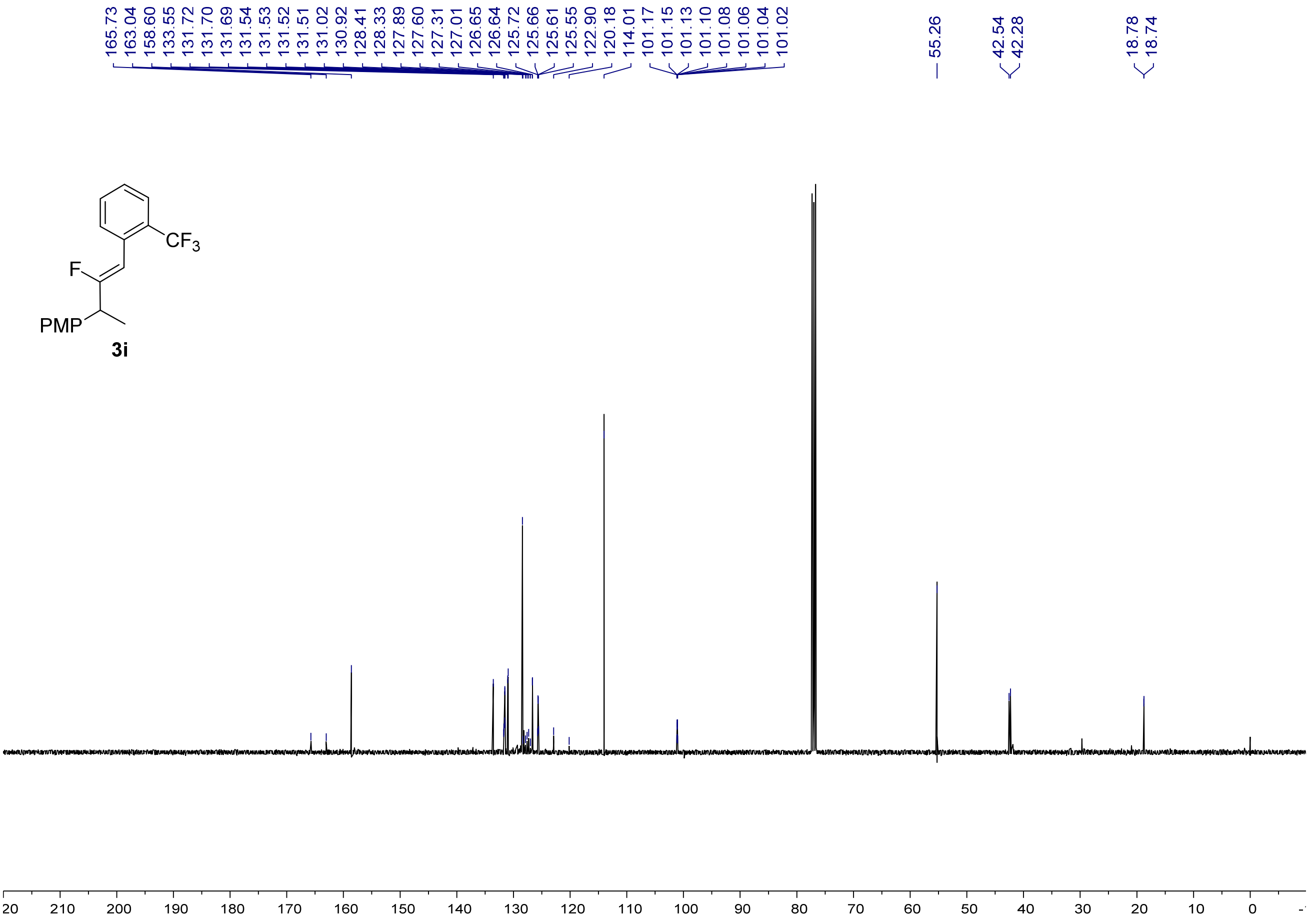
165.73 163.04 158.60 133.55 131.72 131.70 131.69 131.54 131.53 131.52 131.51 131.02 130.92 128.41 128.33 127.89 127.60 127.31 127.01 126.65 126.64 125.72 125.66 125.61 125.55 122.90 120.18 114.01 101.17 101.15 101.13 101.10 101.08 101.06 101.04 101.02
55.26
42.54 42.28
18.78 18.74
CF3
F
PMP
3i
20 210 200 190 180 170 160 150 140 130 120 110 100 90 80 70 60 50 40 30 20 10 0

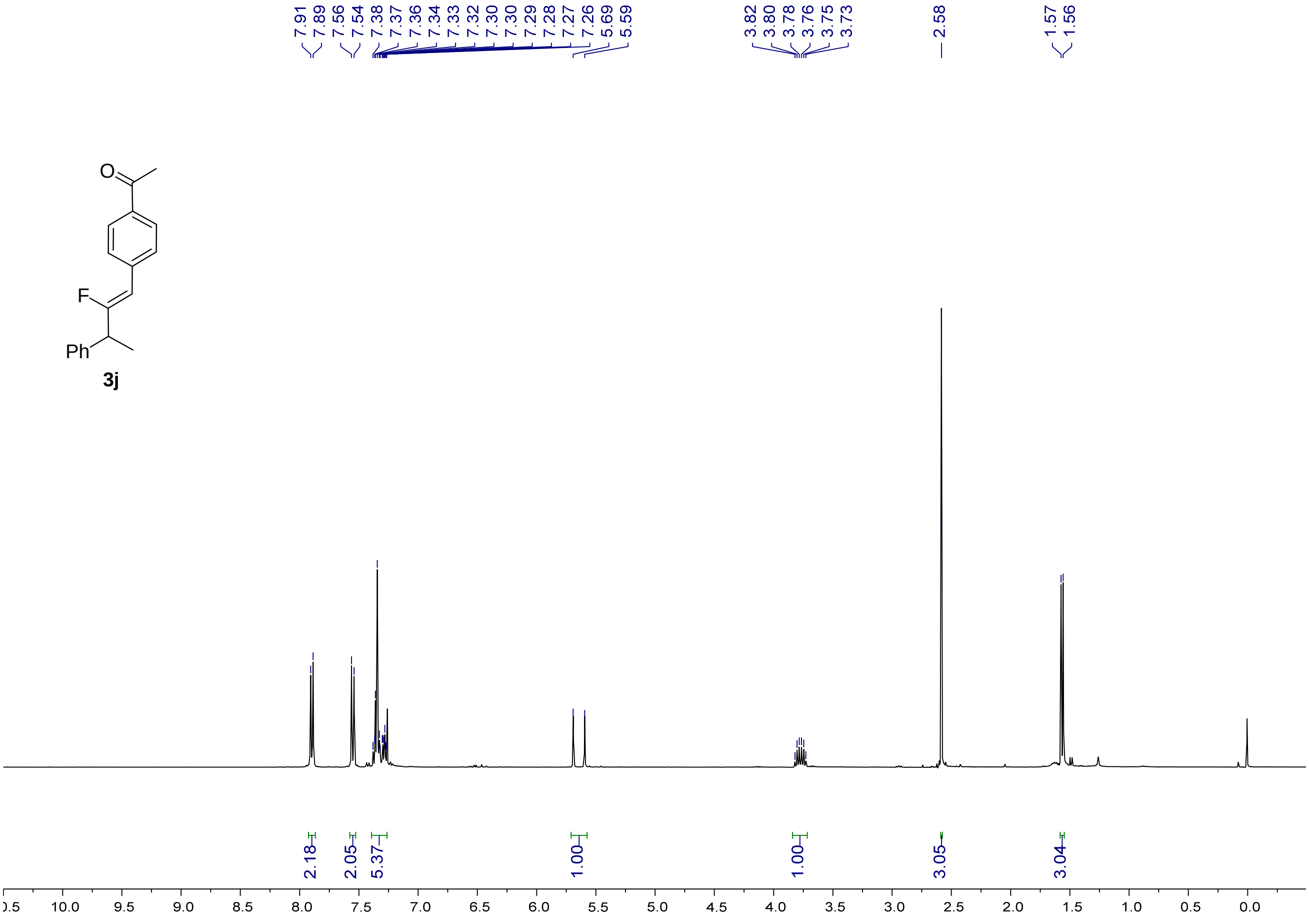
7.91 7.89 7.56 7.54 7.38 7.37 7.36 7.34 7.33 7.32 7.30 7.30 7.29 7.28 7.27 7.26 5.69 5.59
3.82 3.80 3.78 3.76 3.75 3.73
2.58
1.57 1.56
O
F
Ph
3j
2.18 2.05 5.37 1.00 1.00 3.05 3.04
10.0 9.5 9.0 8.5 8.0 7.5 7.0 6.5 6.0 5.5 5.0 4.5 4.0 3.5 3.0 2.5 2.0 1.5 1.0 0.5 0.0

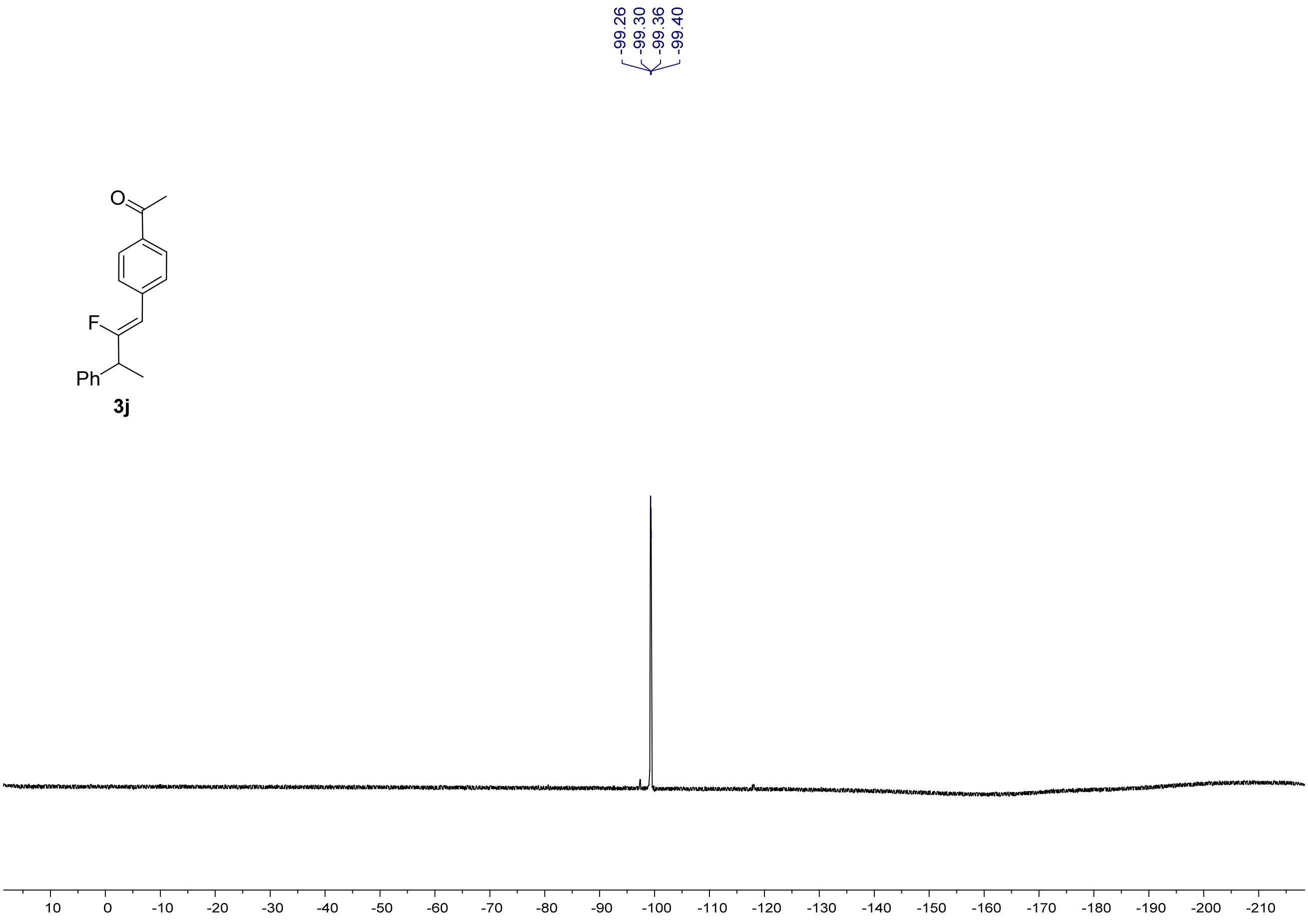
-99.26
-99.30
-99.36
-99.40
O
F
Ph
3j

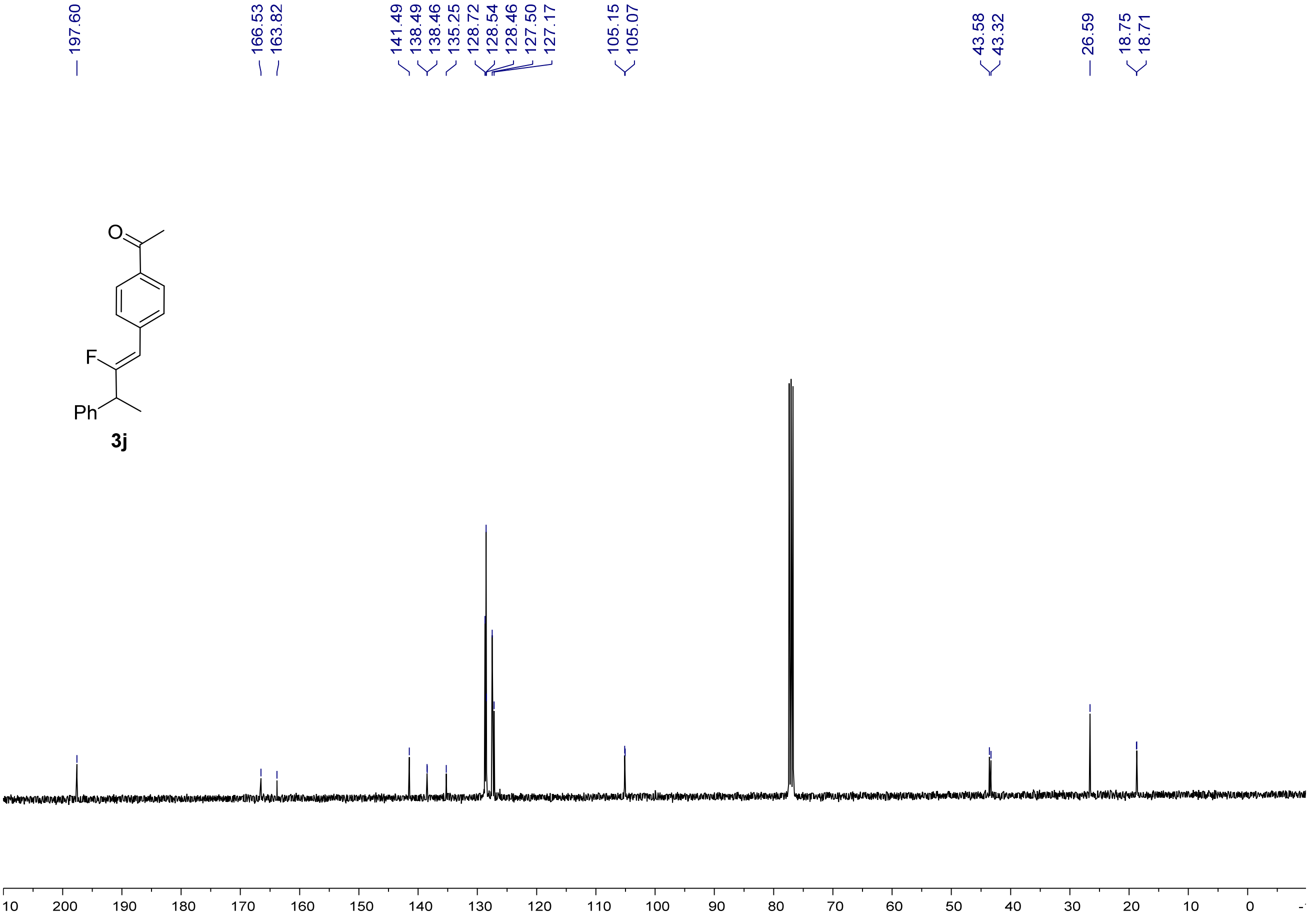
197.60
166.53
163.82
141.49
138.49
138.46
135.25
128.72
128.54
128.46
127.50
127.17
105.15
105.07
43.58
43.32
26.59
18.75
18.71
O
F
Ph
3j

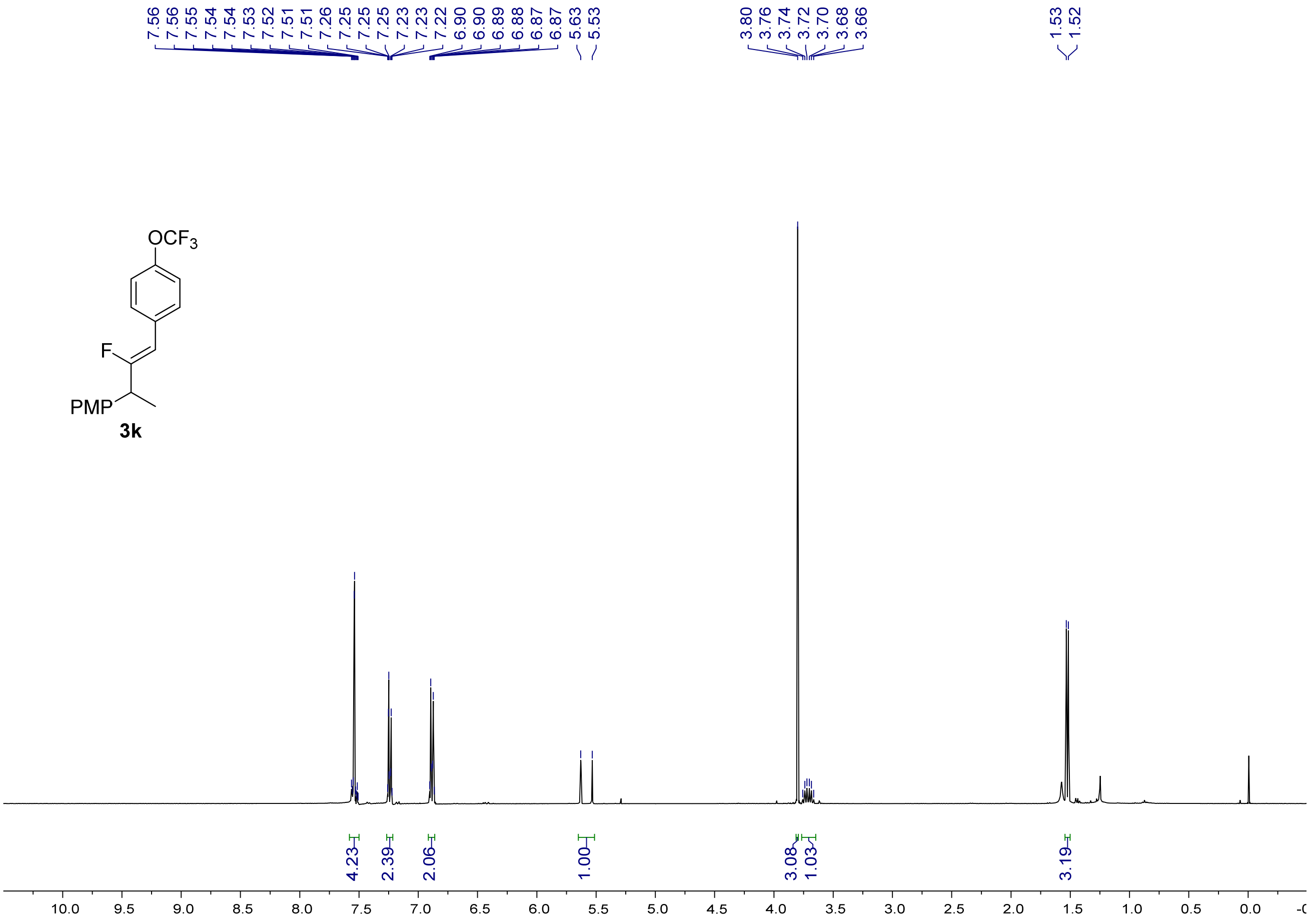
OCF3
F
PMP
3k

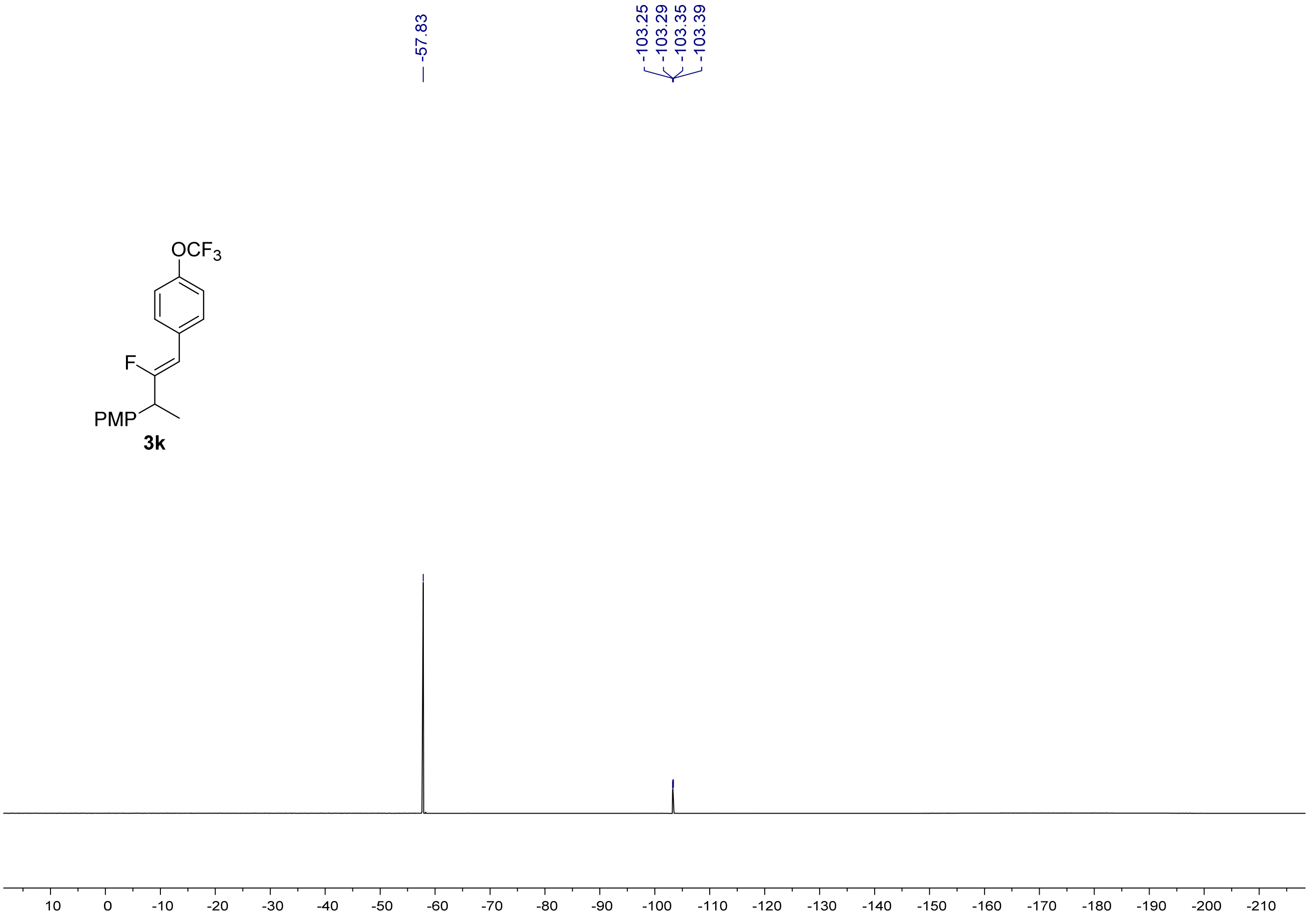
OCF3
F
PMP
3k

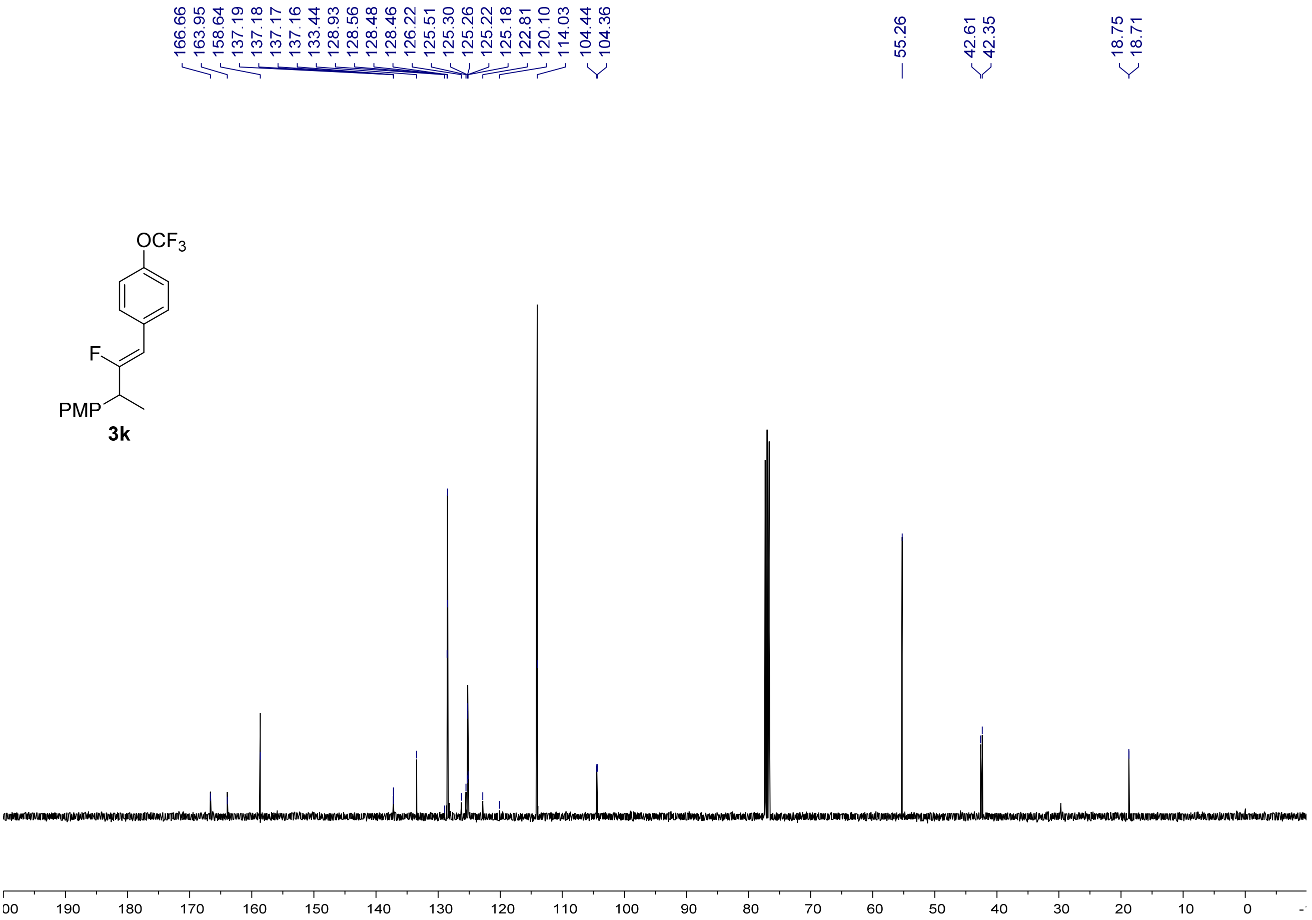
166.66 163.95 158.64 137.19 137.18 137.17 137.16 133.44 128.93 128.56 128.48 128.46 126.22 125.51 125.30 125.26 125.22 125.18 122.81 120.10 114.03 104.44 104.36
55.26
42.61 42.35
18.75 18.71
OCF3
F
PMP
3k
00 190 180 170 160 150 140 130 120 110 100 90 80 70 60 50 40 30 20 10 0

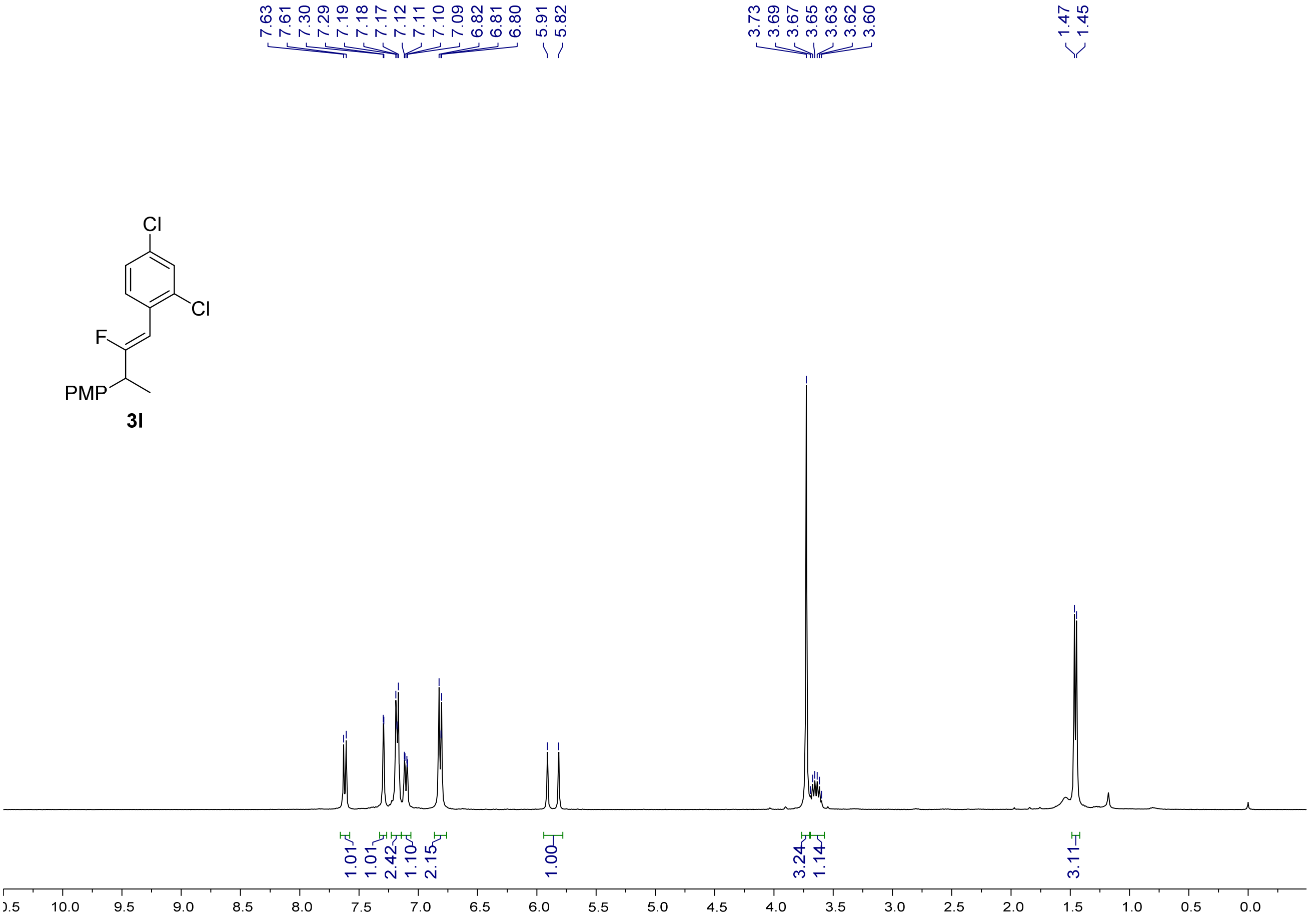
7.63 7.61 7.30 7.29 7.19 7.18 7.17 7.12 7.11 7.10 7.09 6.82 6.81 6.80 5.91 5.82
3.73 3.69 3.67 3.65 3.63 3.62 3.60
1.47 1.45
Cl
Cl
F
PMP
3l
1.01 1.01 2.42 1.10 2.15 1.00 3.24 1.14 3.11
10.0 9.5 9.0 8.5 8.0 7.5 7.0 6.5 6.0 5.5 5.0 4.5 4.0 3.5 3.0 2.5 2.0 1.5 1.0 0.5 0.0

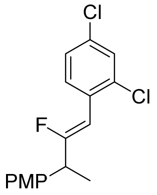
Cl
Cl
F
PMP

3l

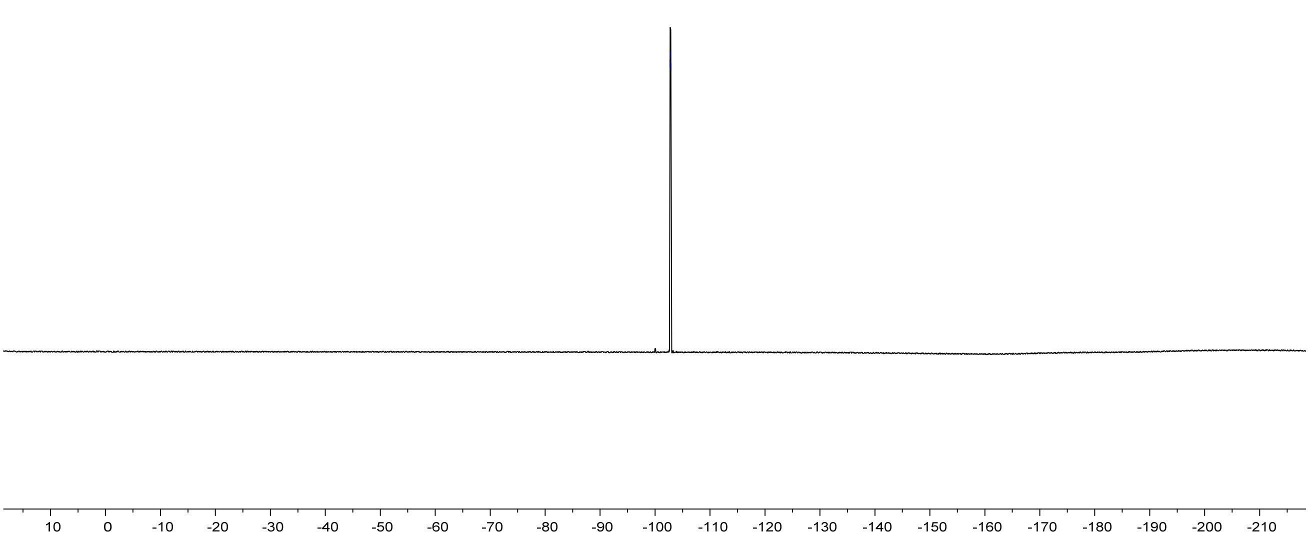
-102.71
-102.75
-102.81
-102.85
10 0 -10 -20 -30 -40 -50 -60 -70 -80 -90 -100 -110 -120 -130 -140 -150 -160 -170 -180 -190 -200 -210

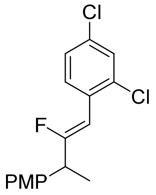
Cl
Cl
F
PMP

3l

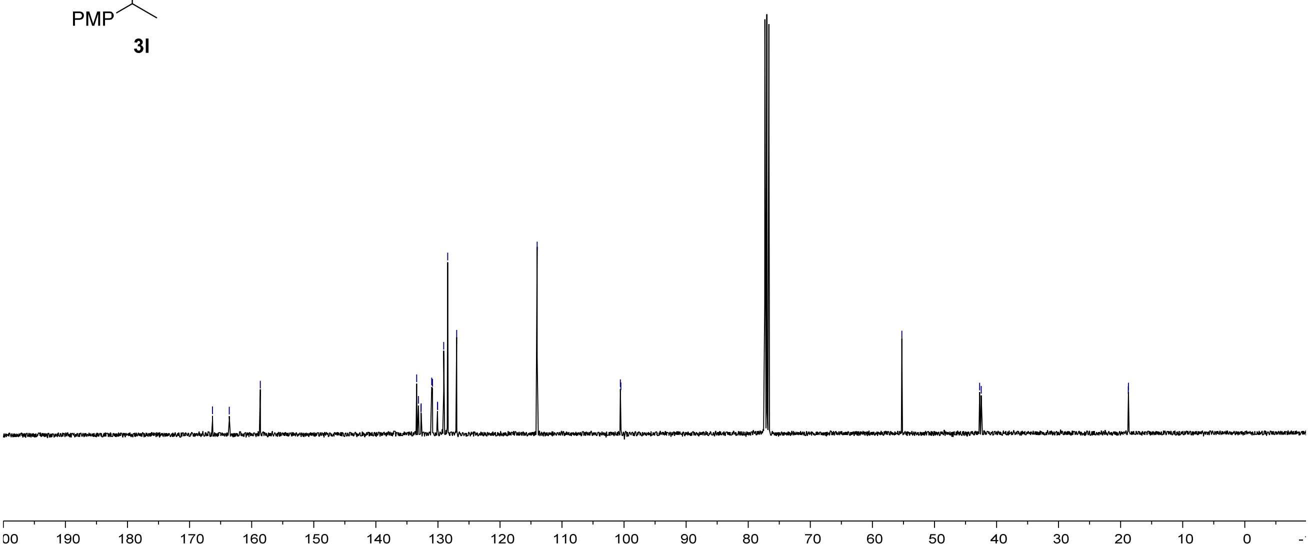
166.31
163.61
158.61
133.43
133.13
132.72
132.70
131.00
130.87
130.06
130.04
129.05
128.42
126.97
114.01
100.59
100.52
55.26
42.73
42.47
18.78
18.74
00 190 180 170 160 150 140 130 120 110 100 90 80 70 60 50 40 30 20 10 0

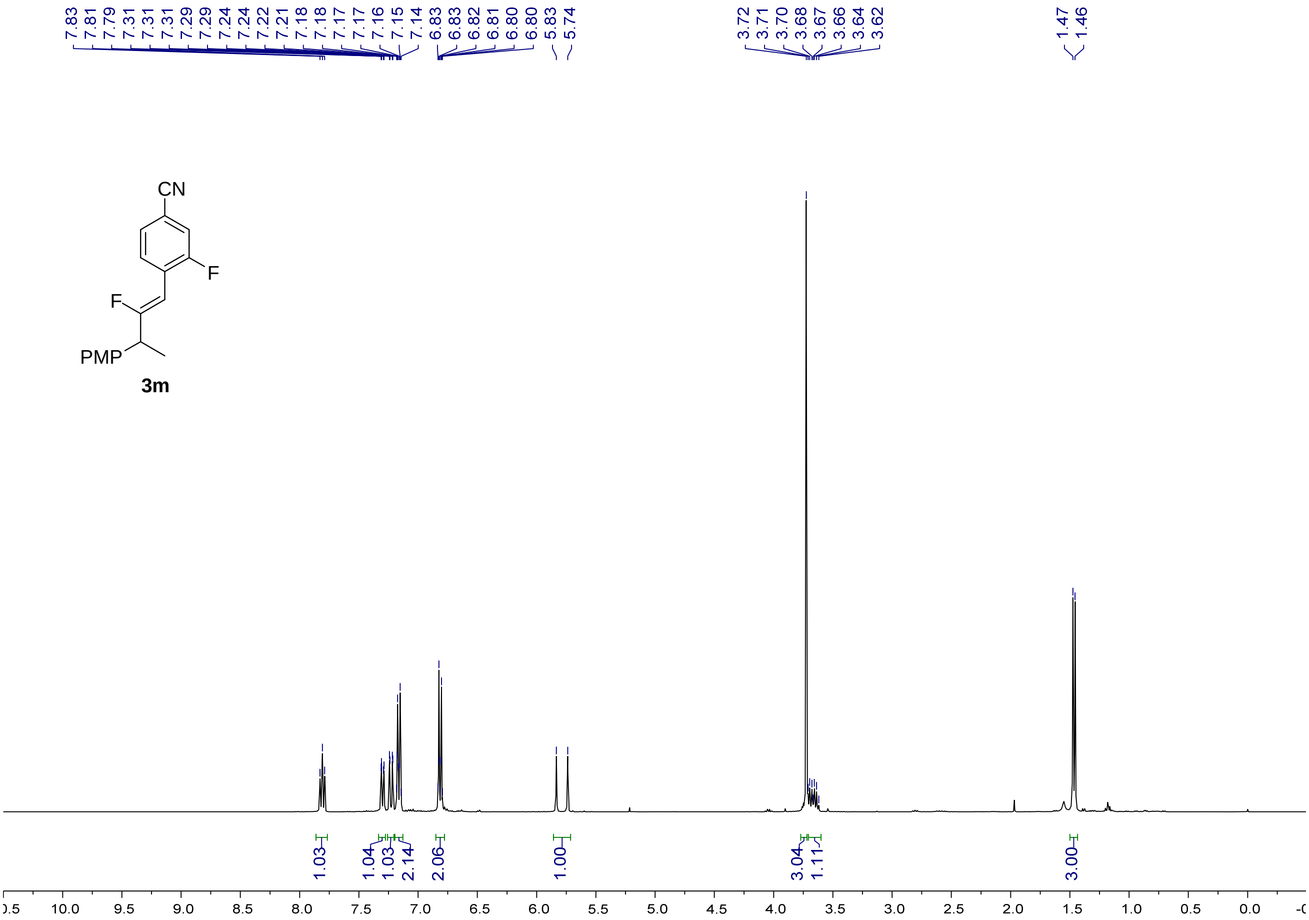
CN
F
F
PMP
3m

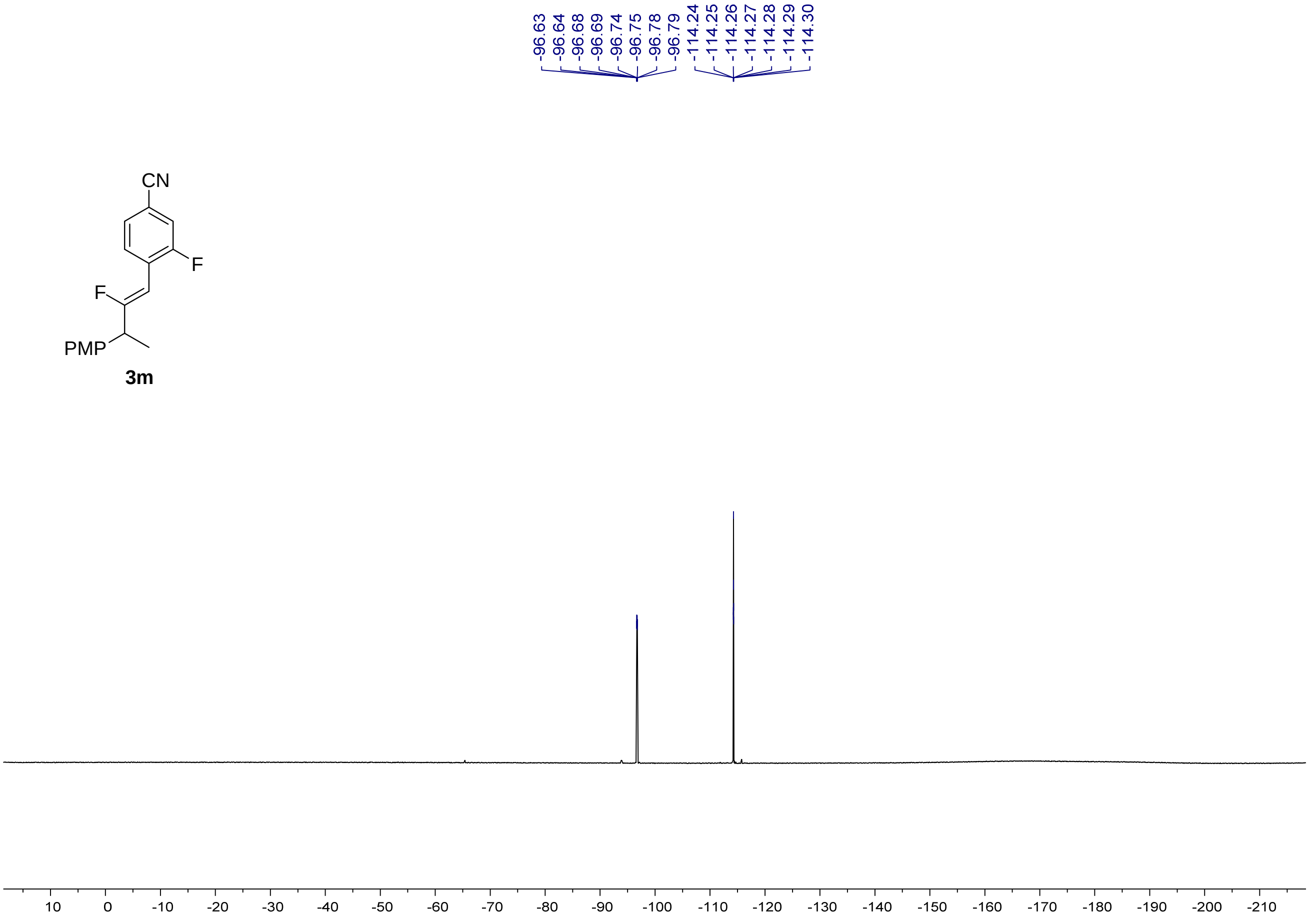
CN
F
F
PMP
3m

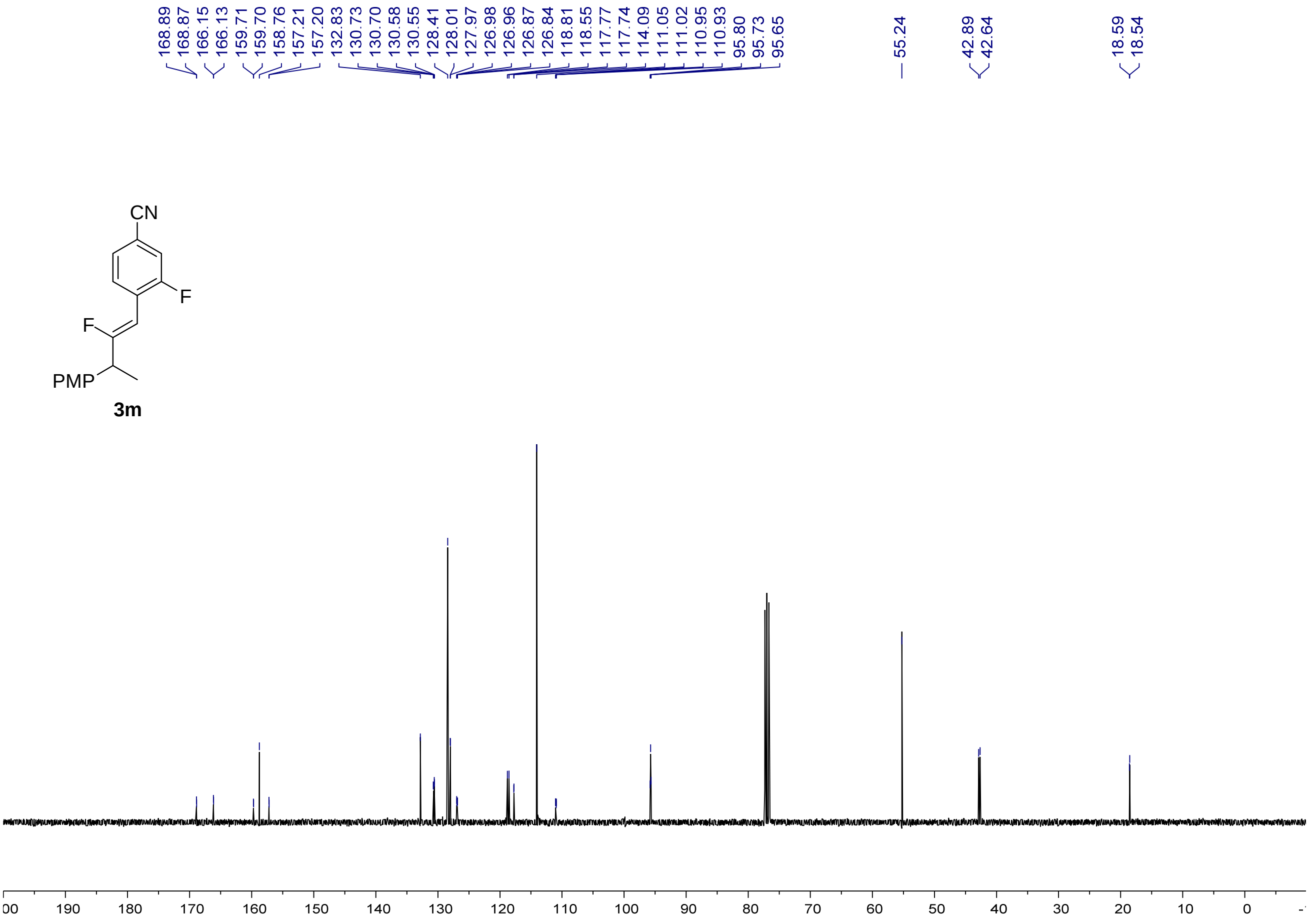
168.89
168.87
166.15
166.13
159.71
159.70
158.76
157.21
157.20
132.83
130.73
130.70
130.58
130.55
128.41
128.01
127.97
126.98
126.96
126.87
126.84
118.81
118.55
117.77
117.74
114.09
111.05
111.02
110.95
110.93
95.80
95.73
95.65
55.24
42.89
42.64
18.59
18.54
CN
F
F
PMP
3m

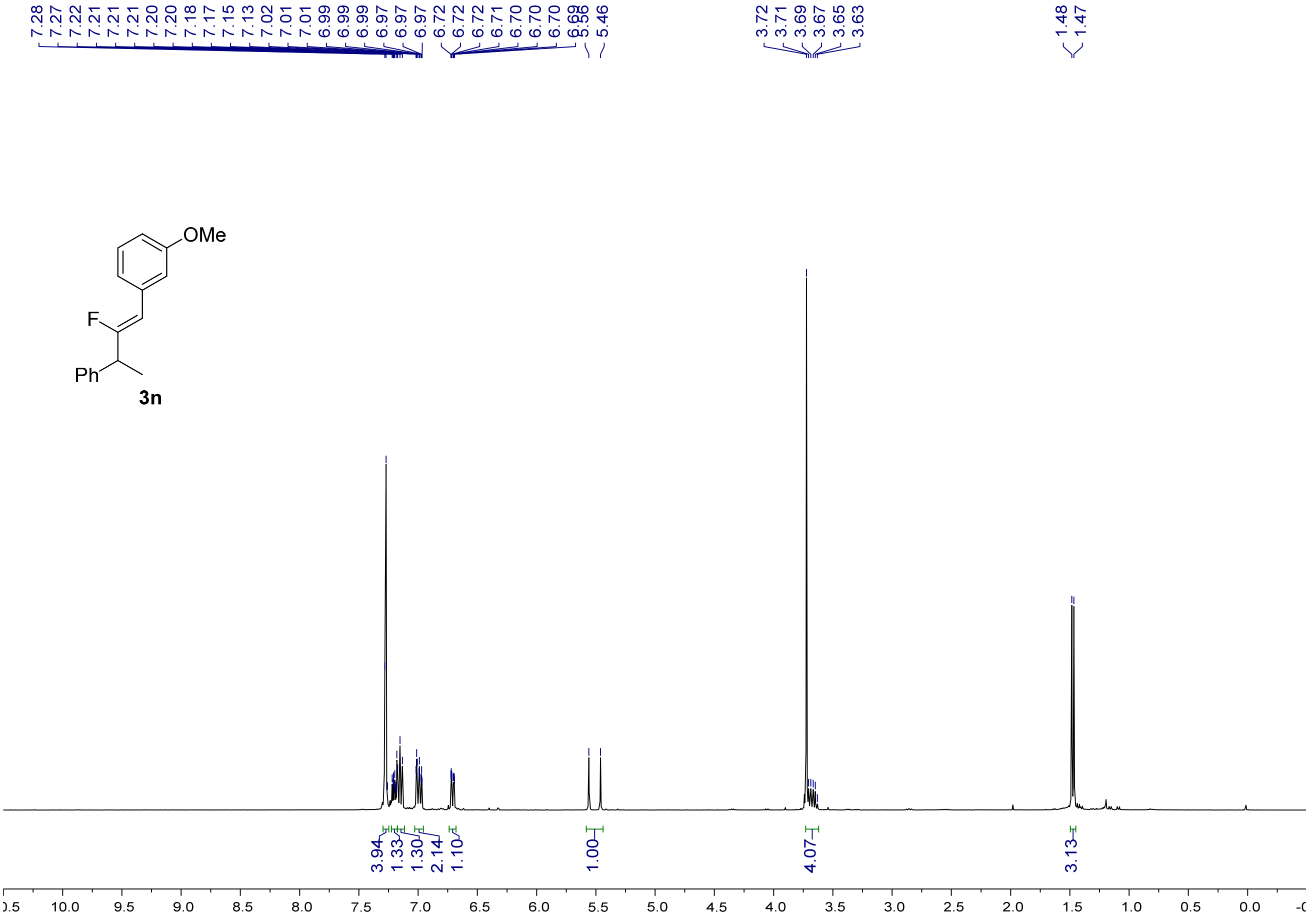
7.28
7.27
7.22
7.21
7.21
7.21
7.20
7.18
7.17
7.15
7.13
7.02
7.01
7.01
6.99
6.99
6.97
6.97
6.97
6.72
6.72
6.71
6.70
6.70
6.70
6.69
5.56
5.46
3.72
3.71
3.69
3.67
3.65
3.63
1.48
1.47
OMe
F
Ph
3n
3.94
1.33
1.30
2.14
1.10
1.00
4.07
3.13

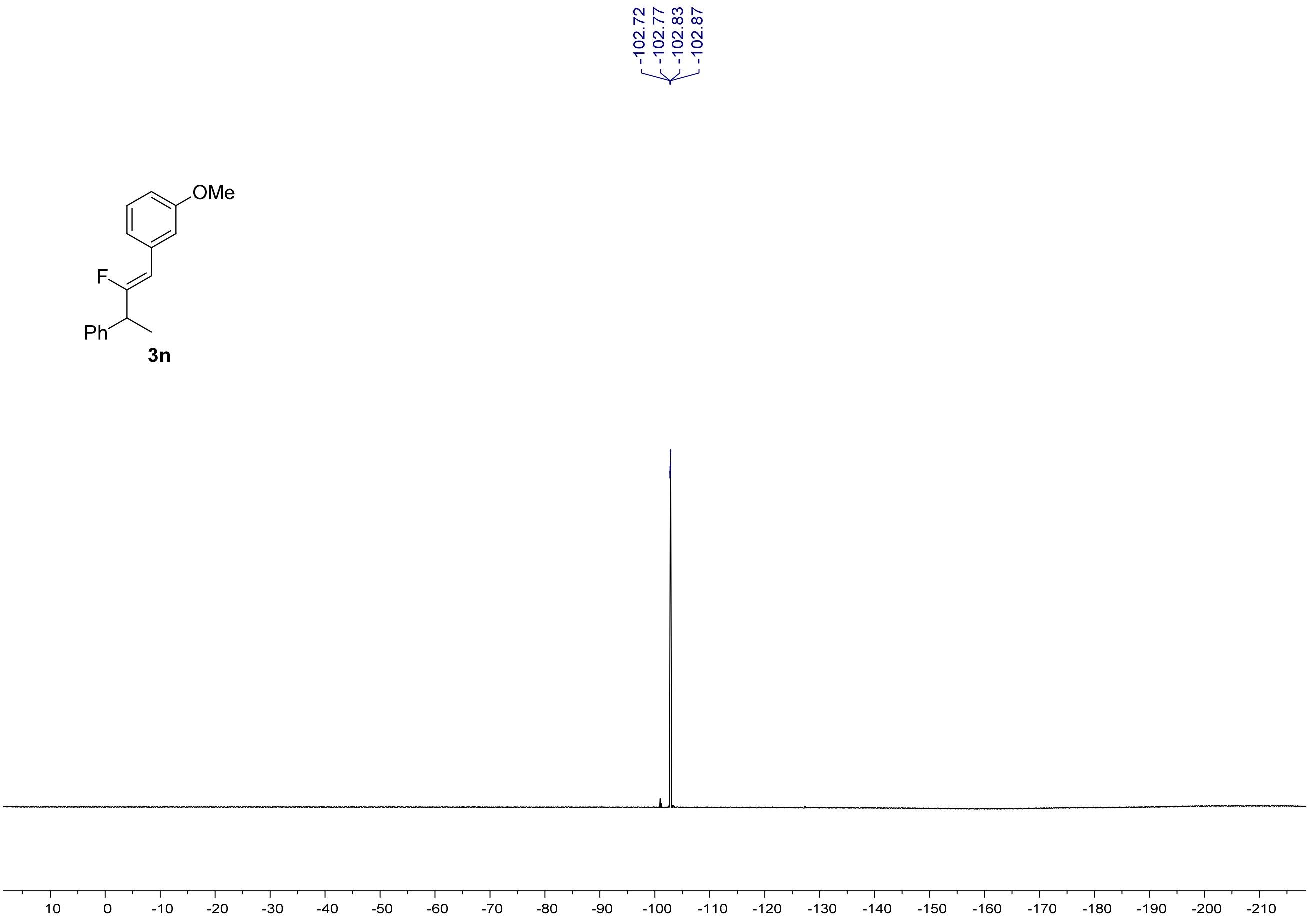
-102.72
-102.77
-102.83
-102.87
OMe
F
Ph
3n
10 0 -10 -20 -30 -40 -50 -60 -70 -80 -90 -100 -110 -120 -130 -140 -150 -160 -170 -180 -190 -200 -210

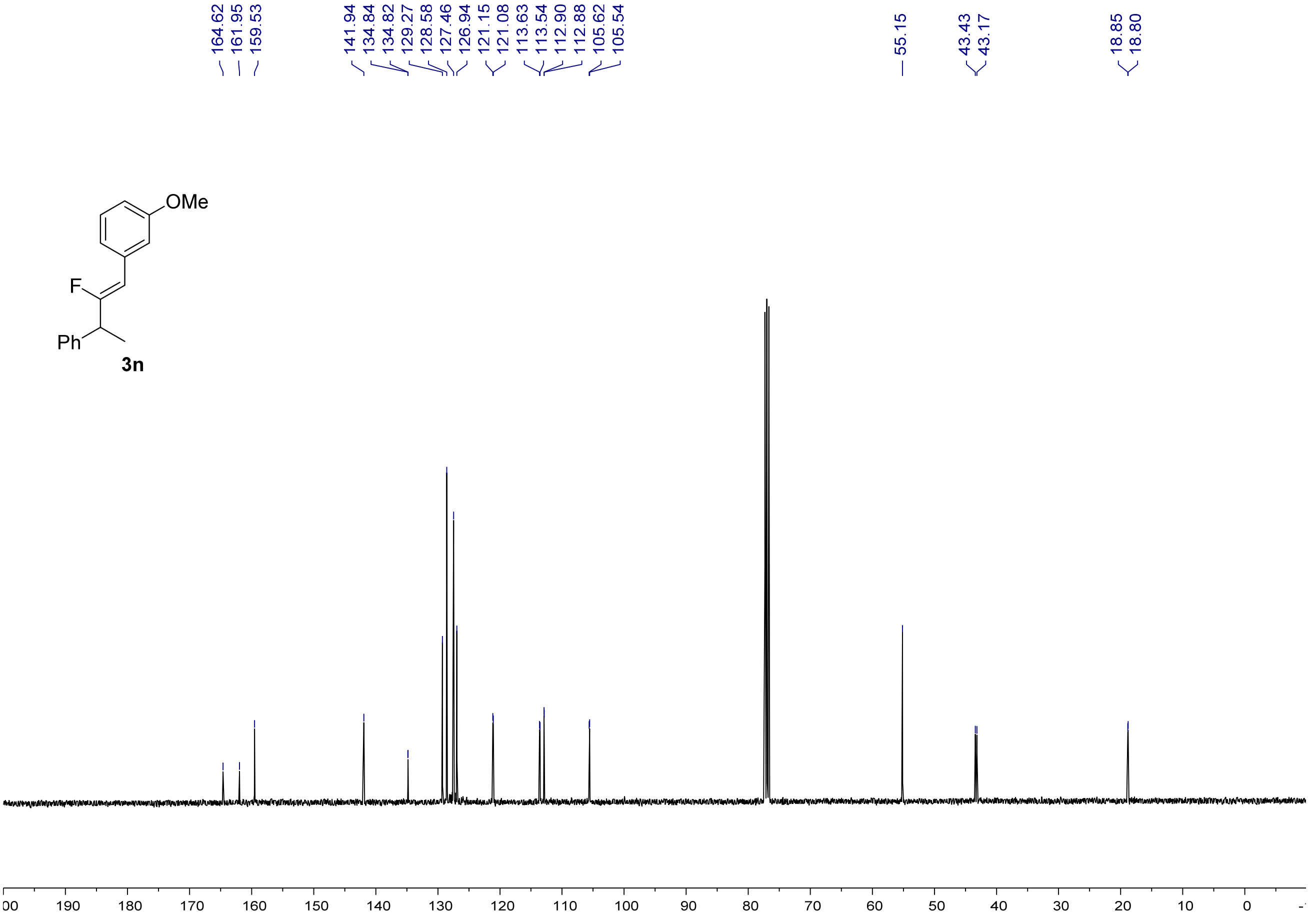
164.62
161.95
159.53
141.94
134.84
134.82
129.27
128.58
127.46
126.94
121.15
121.08
113.63
113.54
112.90
112.88
105.62
105.54
55.15
43.43
43.17
18.85
18.80
OMe
F
Ph
3n
190 180 170 160 150 140 130 120 110 100 90 80 70 60 50 40 30 20 10 0

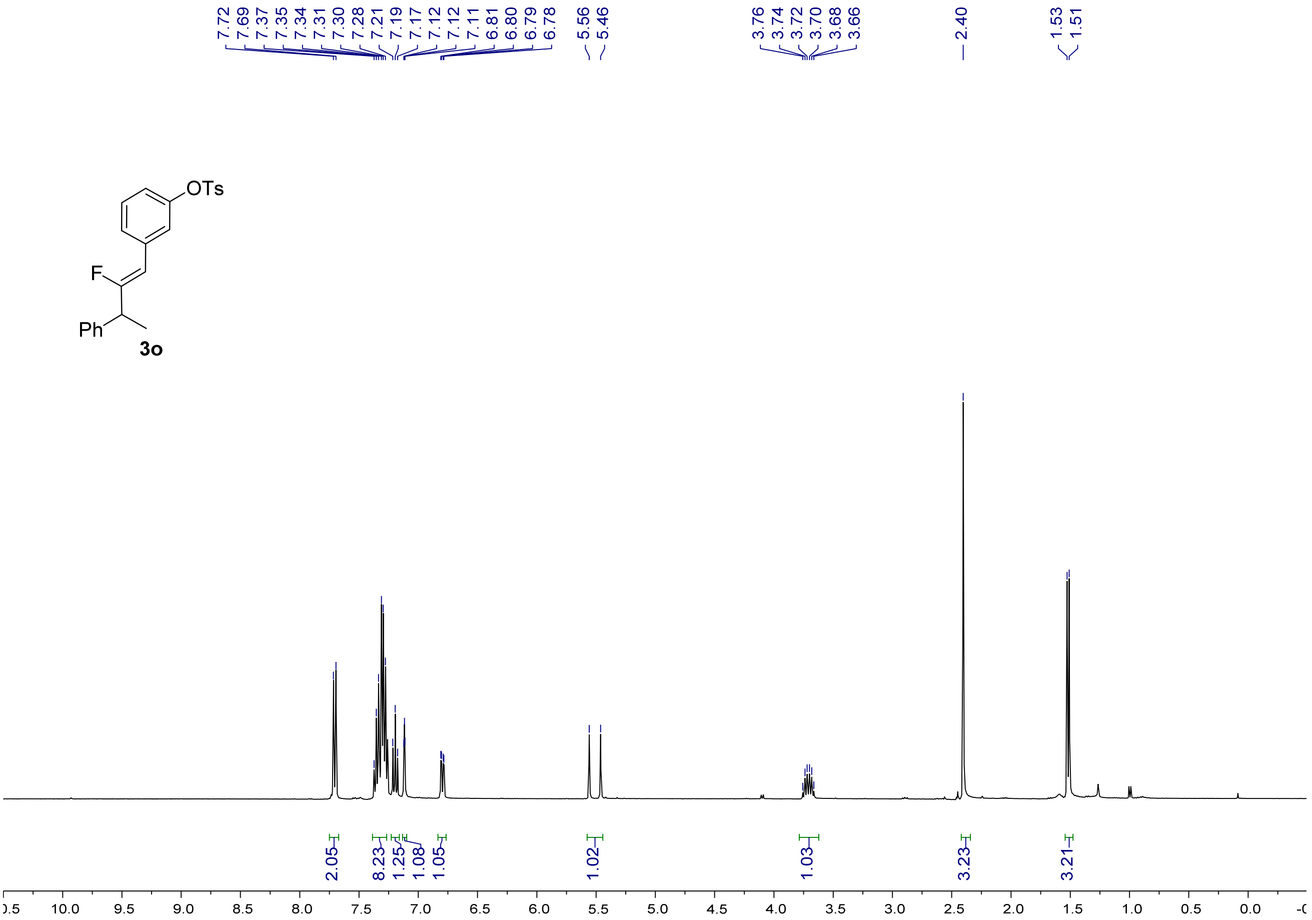
OTs
F
Ph
3o
7.72
7.69
7.37
7.35
7.34
7.31
7.30
7.28
7.21
7.19
7.17
7.12
7.12
7.11
6.81
6.80
6.79
6.78
5.56
5.46
3.76
3.74
3.72
3.70
3.68
3.66
2.40
1.53
1.51
2.05
8.23
1.25
1.08
1.05
1.02
1.03
3.23
3.21

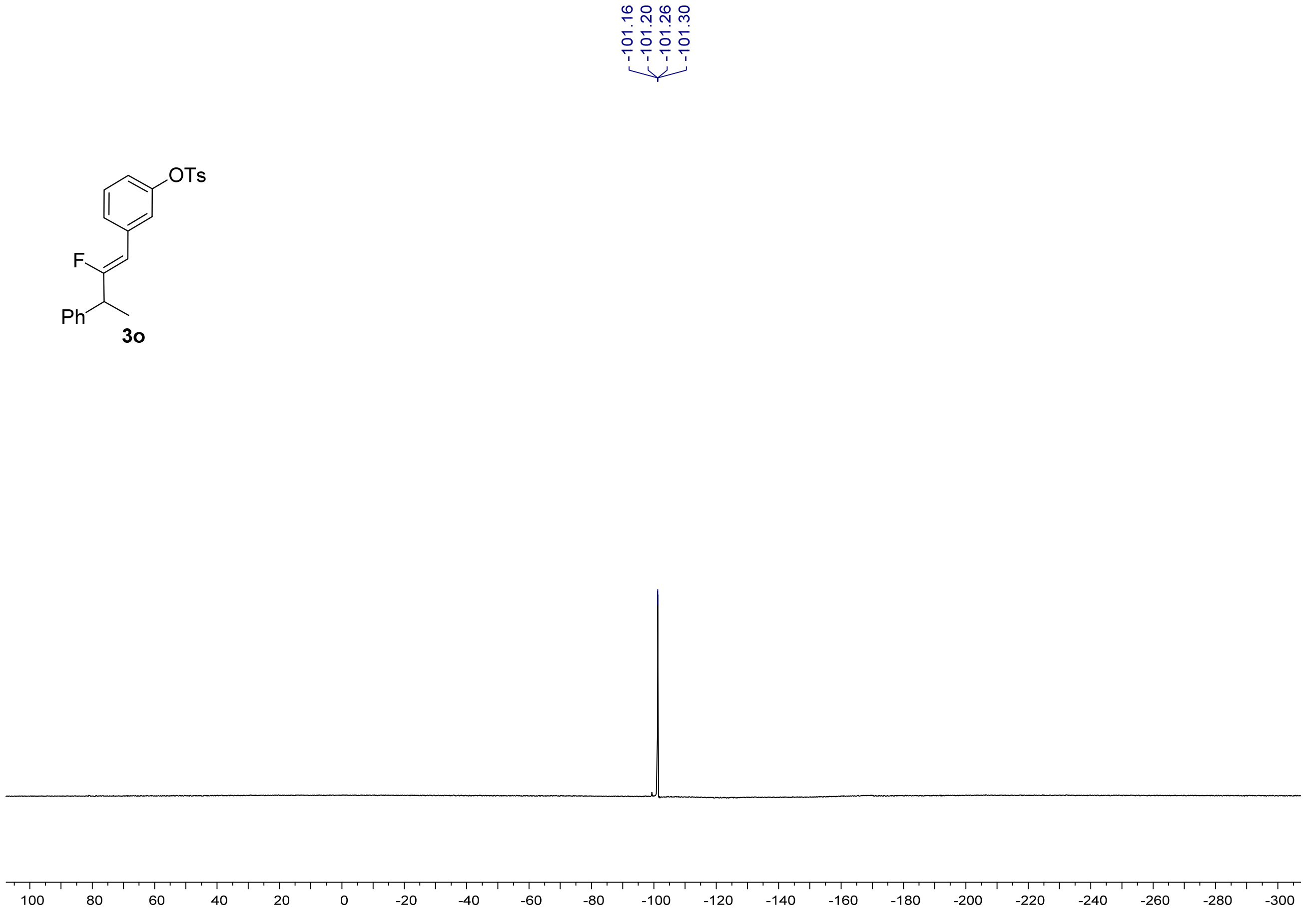
OTs
F
Ph
3o
-101.16
-101.20
-101.26
-101.30

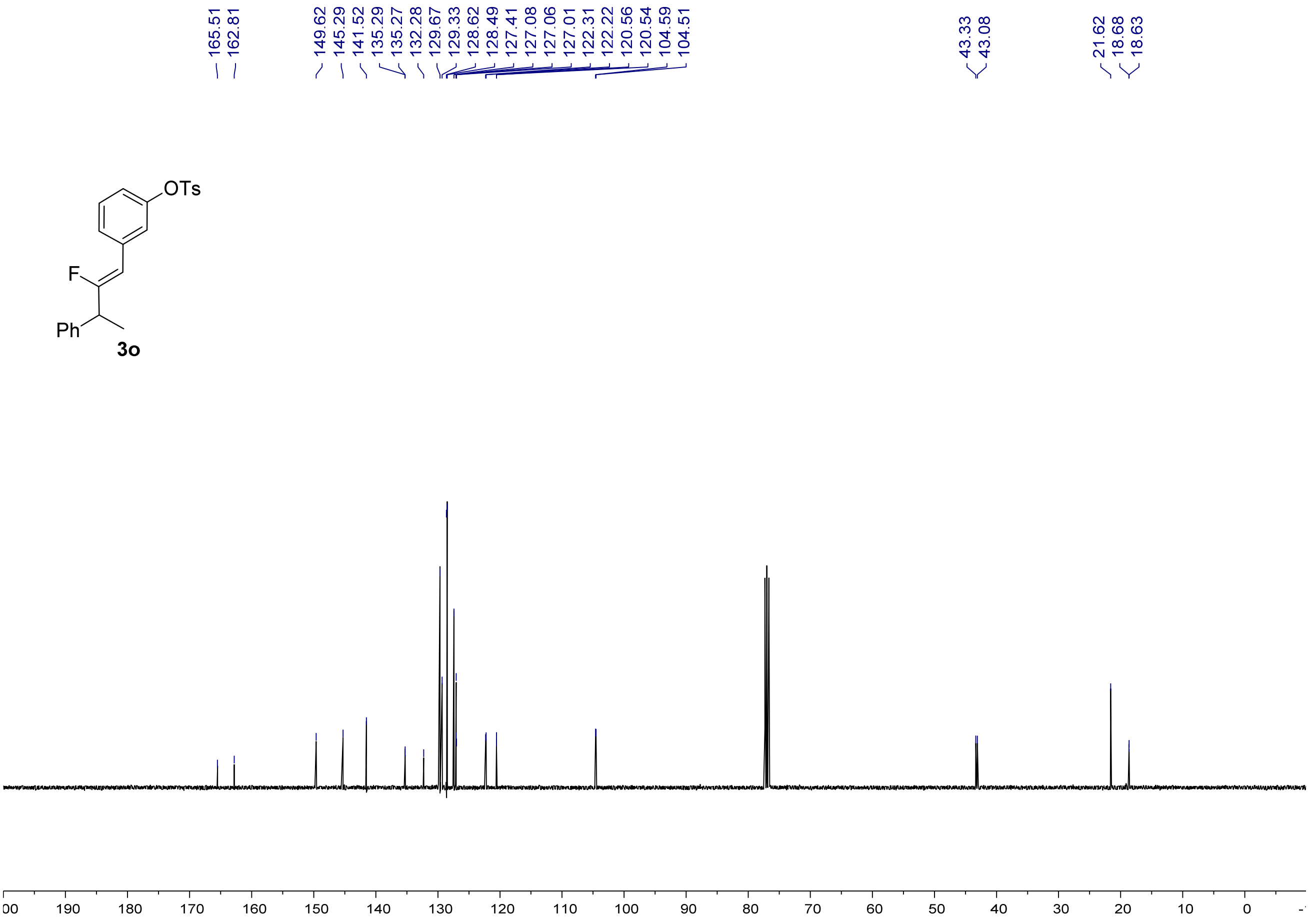
165.51
162.81
149.62
145.29
141.52
135.29
135.27
132.28
129.67
129.33
128.62
128.49
127.41
127.08
127.06
127.01
122.31
122.22
120.56
120.54
104.59
104.51
43.33
43.08
21.62
18.68
18.63
OTs
F
Ph
3o
190 180 170 160 150 140 130 120 110 100 90 80 70 60 50 40 30 20 10 0

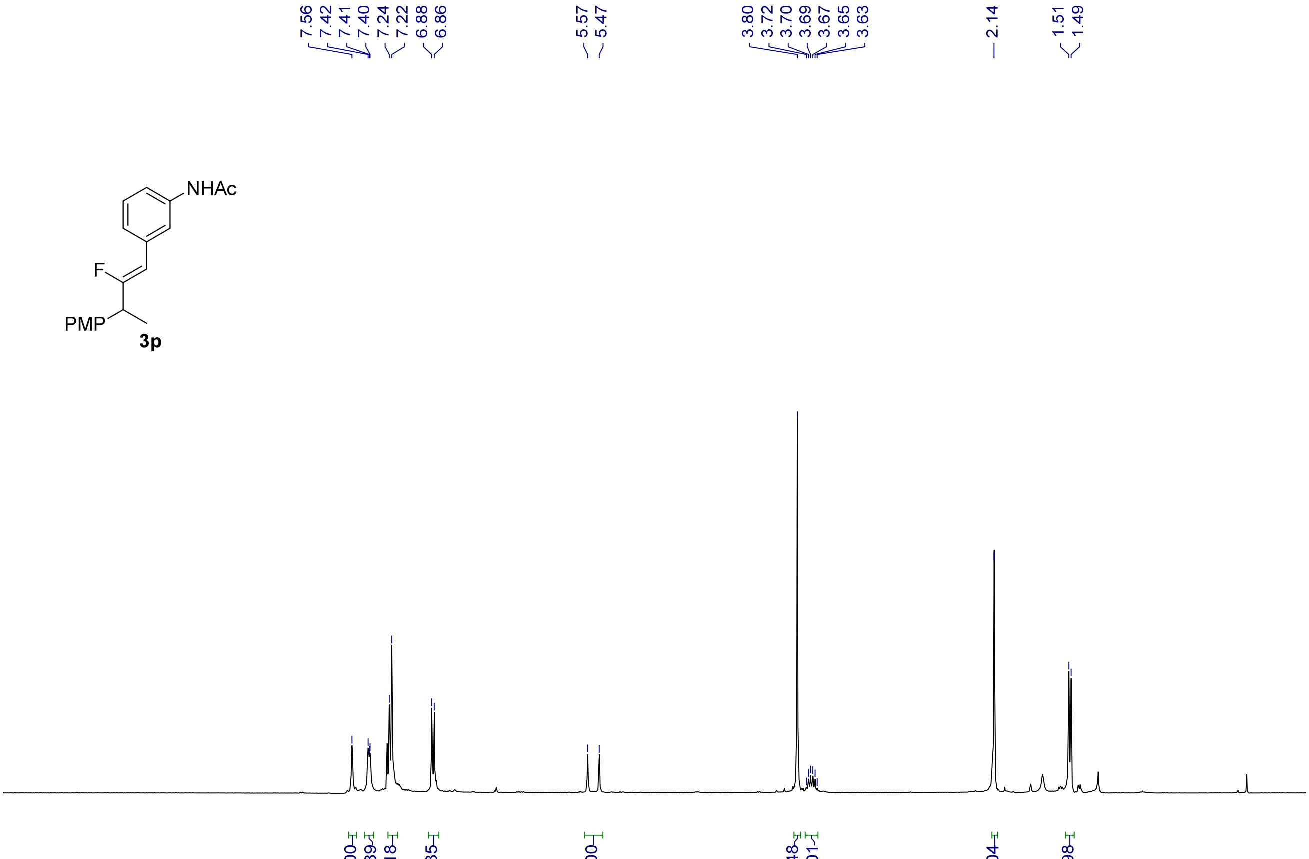
7.56
7.42
7.41
7.40
7.24
7.22
6.88
6.86
5.57
5.47
3.80
3.72
3.70
3.69
3.67
3.65
3.63
2.14
1.51
1.49
NHAc
F
PMP
3p
1.00
1.89
4.18
2.35
1.00
3.48
1.01
3.04
2.98
10.0 9.5 9.0 8.5 8.0 7.5 7.0 6.5 6.0 5.5 5.0 4.5 4.0 3.5 3.0 2.5 2.0 1.5 1.0 0.5 0.0

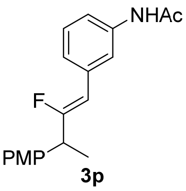
NHAc
F
PMP

3p

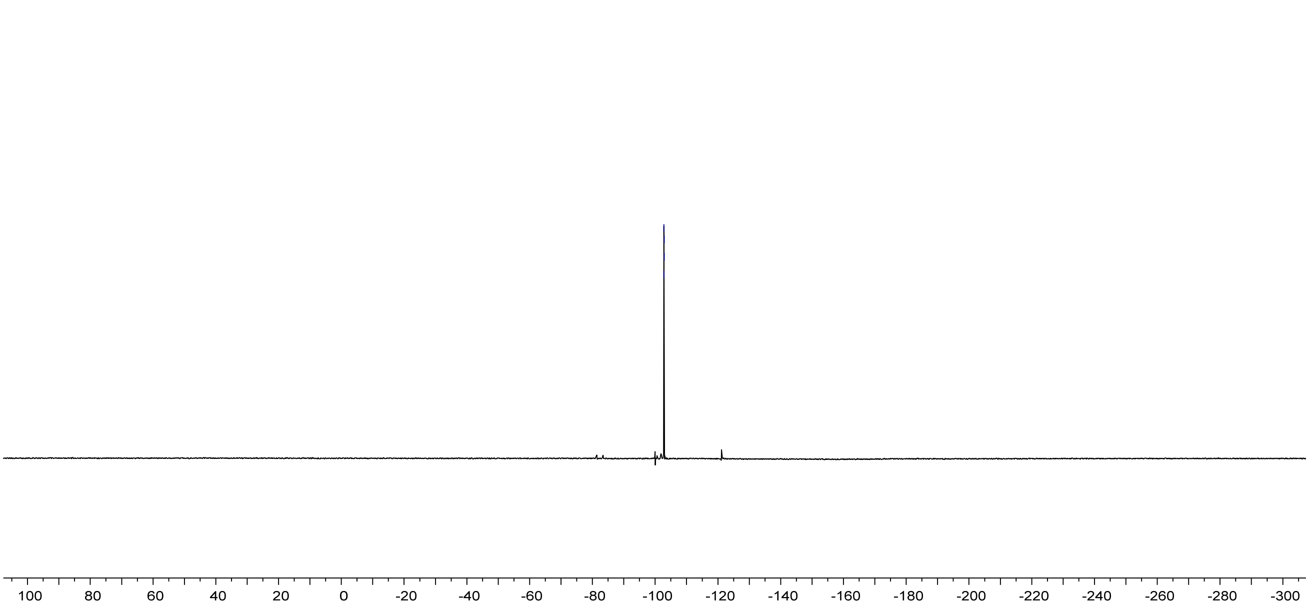
-102.77
-102.81
-102.87
-102.91
100 80 60 40 20 0 -20 -40 -60 -80 -100 -120 -140 -160 -180 -200 -220 -240 -260 -280 -300

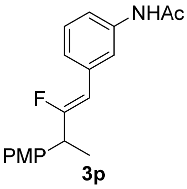
NHAc
F
PMP

3p

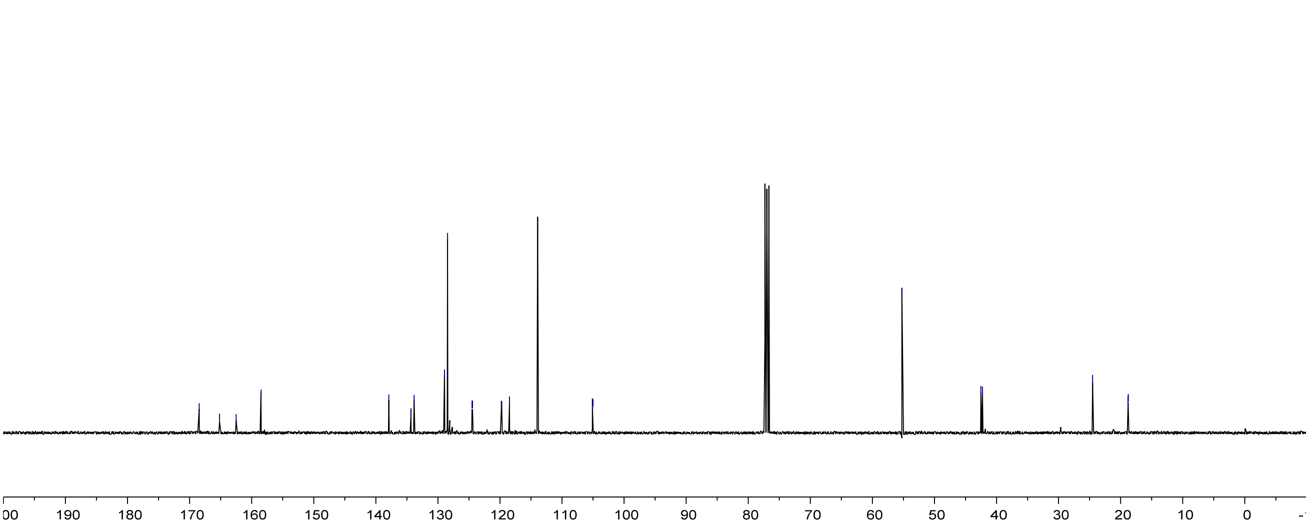
168.46
165.18
162.50
158.49
137.91
134.34
134.32
133.84
128.94
128.43
124.49
124.42
119.79
119.71
118.47
118.46
113.93
105.10
105.02
55.24
42.52
42.26
24.52
18.81
18.77
190 180 170 160 150 140 130 120 110 100 90 80 70 60 50 40 30 20 10 0

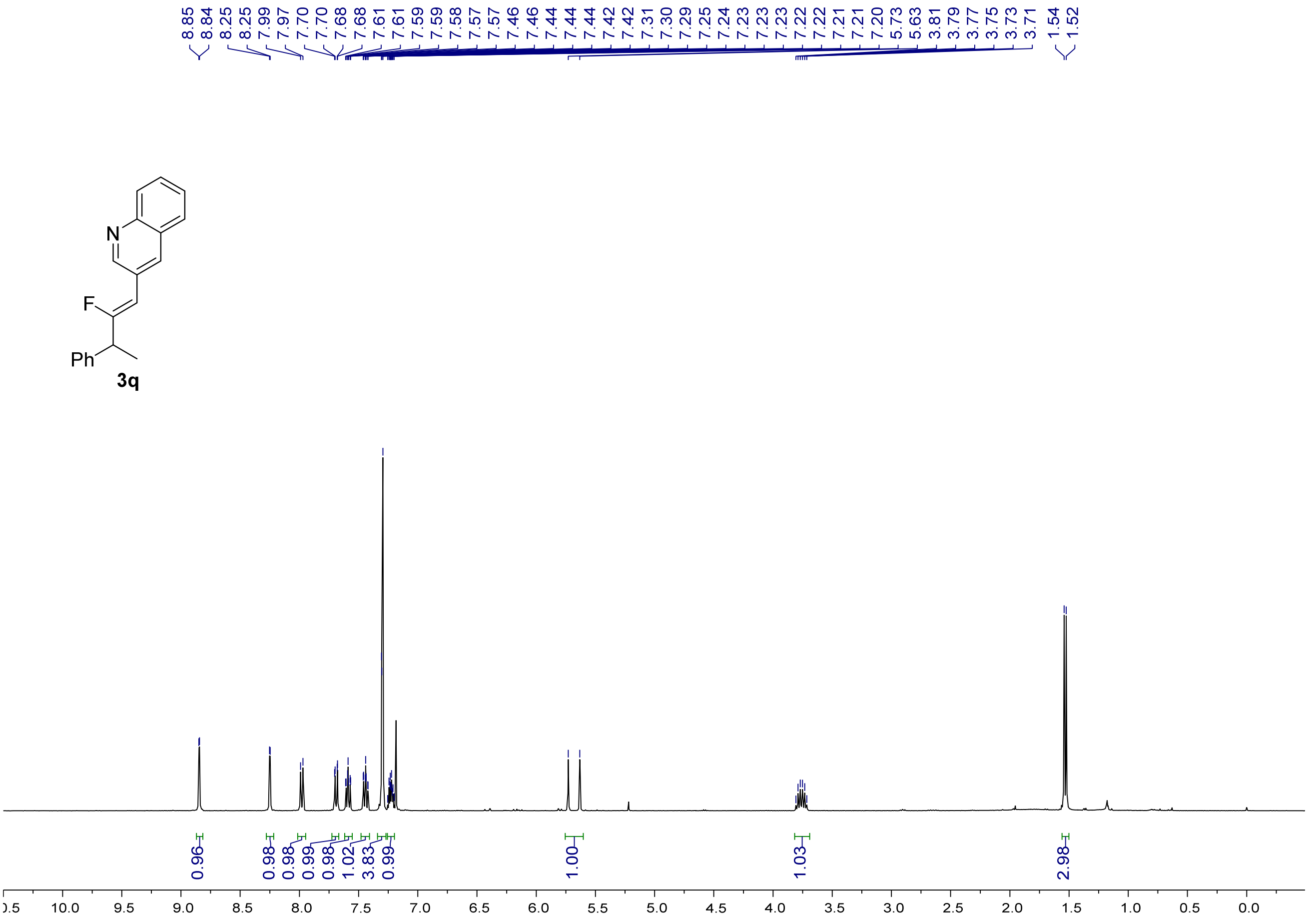
8.85
8.84
8.25
8.25
7.99
7.97
7.70
7.70
7.68
7.68
7.61
7.61
7.59
7.59
7.58
7.57
7.57
7.46
7.46
7.44
7.44
7.44
7.42
7.42
7.31
7.30
7.29
7.25
7.24
7.23
7.23
7.23
7.22
7.22
7.21
7.21
7.20
5.73
5.63
3.81
3.79
3.77
3.75
3.73
3.71
1.54
1.52
N
F
Ph
3q
0.96
0.98
0.98
0.99
0.98
1.02
3.83
0.99
1.00
1.03
2.98

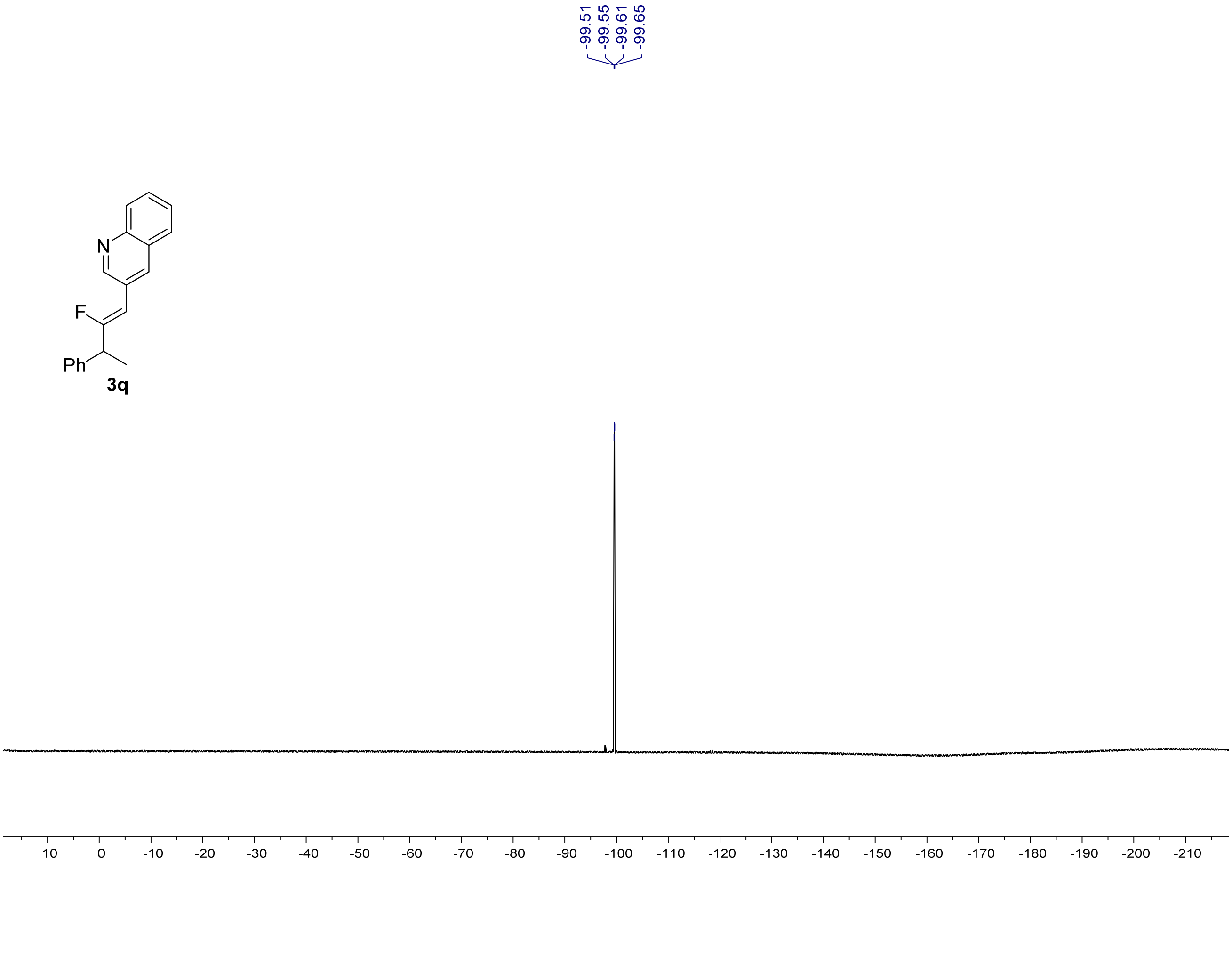
-99.51
-99.55
-99.61
-99.65
N
F
Ph
3q

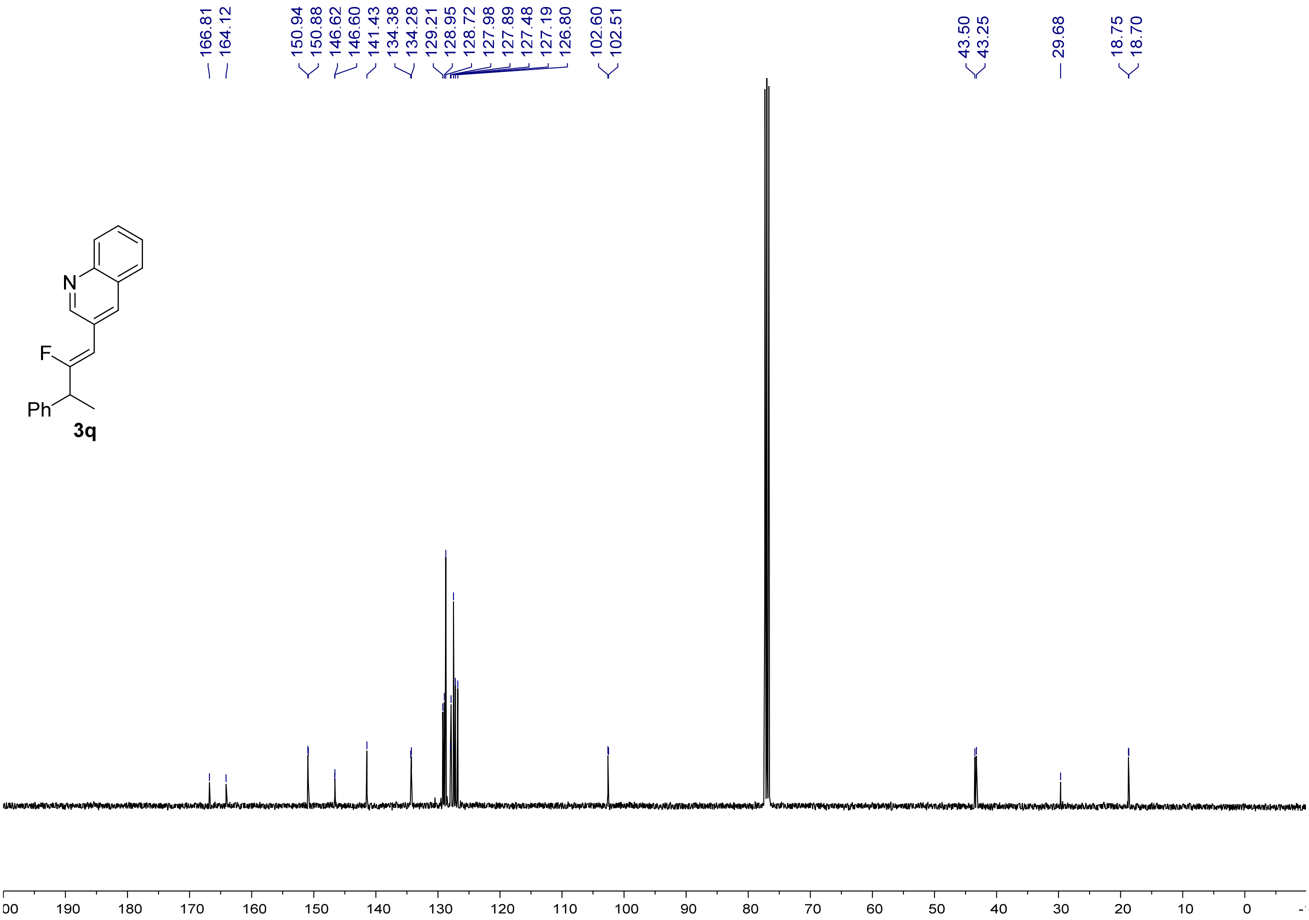
166.81
164.12
150.94
150.88
146.62
146.60
141.43
134.38
134.28
129.21
128.95
128.72
127.98
127.89
127.48
127.19
126.80
102.60
102.51
43.50
43.25
29.68
18.75
18.70
N
F
Ph
3q
00 190 180 170 160 150 140 130 120 110 100 90 80 70 60 50 40 30 20 10 0

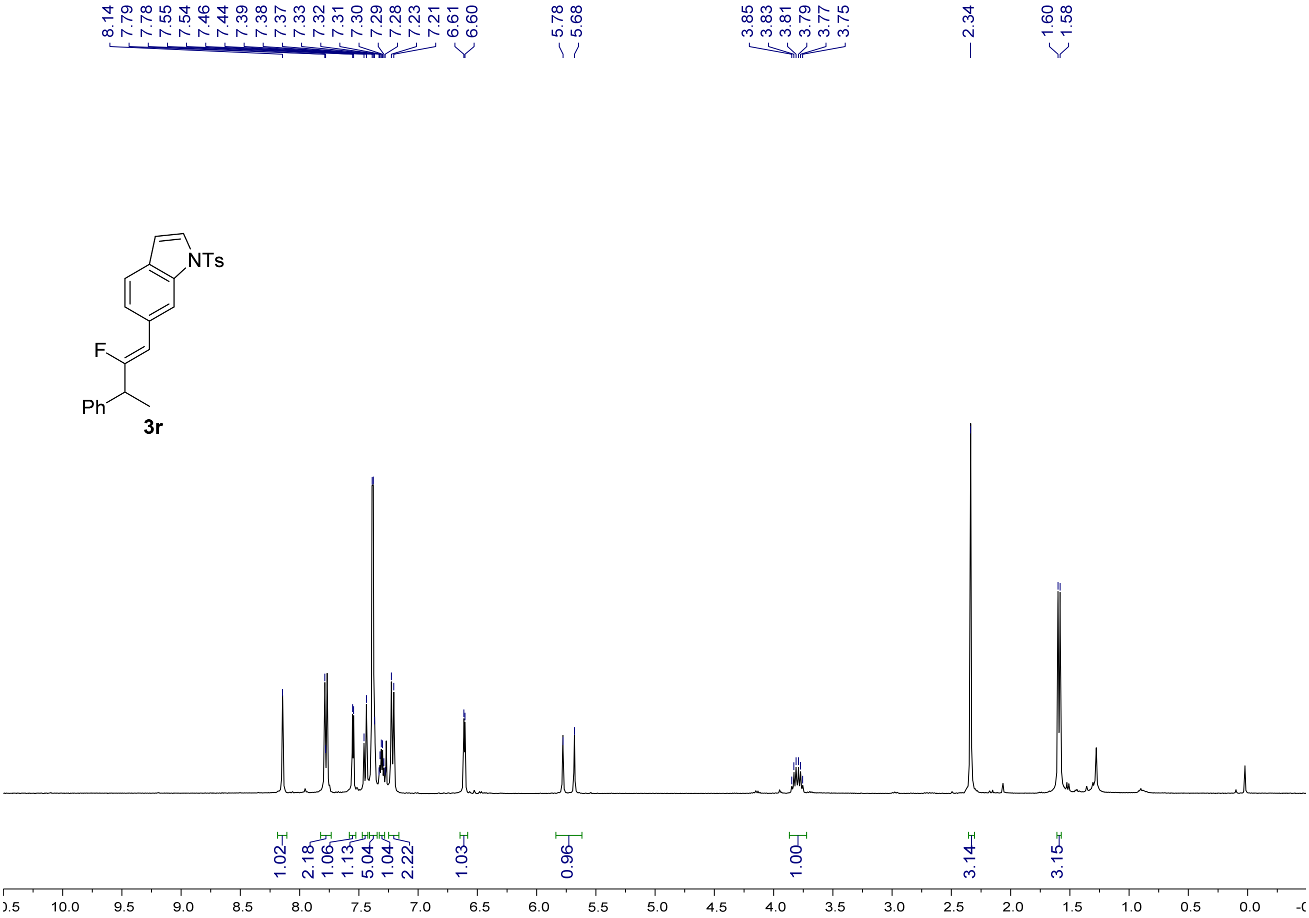
8.14
7.79
7.78
7.55
7.54
7.46
7.44
7.39
7.38
7.37
7.33
7.32
7.31
7.30
7.29
7.28
7.23
7.21
6.61
6.60
5.78
5.68
3.85
3.83
3.81
3.79
3.77
3.75
2.34
1.60
1.58
NTs
F
Ph
3r
1.02
2.18
1.06
1.13
5.04
1.04
2.22
1.03
0.96
1.00
3.14
3.15
10.0 9.5 9.0 8.5 8.0 7.5 7.0 6.5 6.0 5.5 5.0 4.5 4.0 3.5 3.0 2.5 2.0 1.5 1.0 0.5 0.0

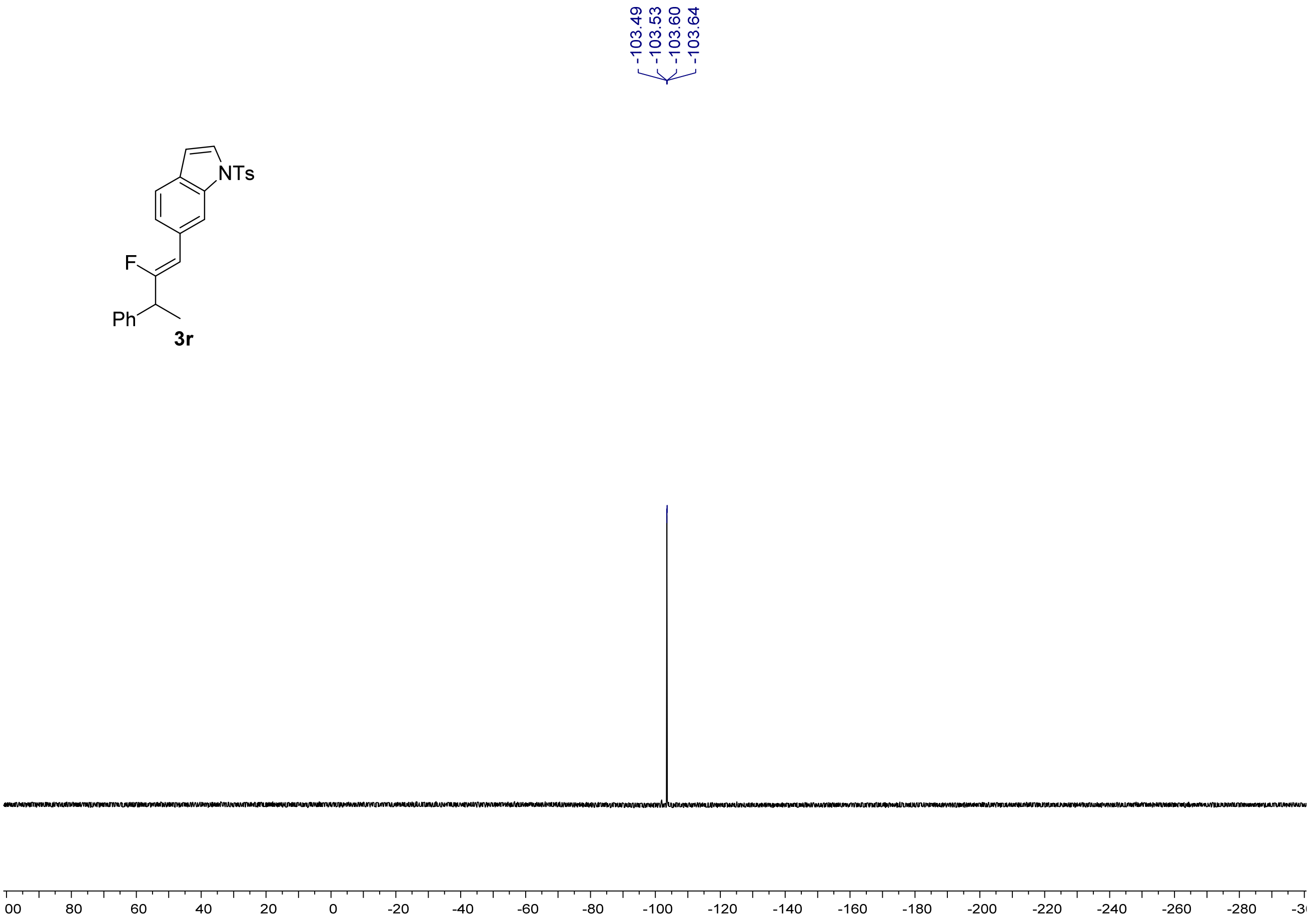
-103.49
-103.53
-103.60
-103.64
NTs
F
Ph
3r
00 80 60 40 20 0 -20 -40 -60 -80 -100 -120 -140 -160 -180 -200 -220 -240 -260 -280

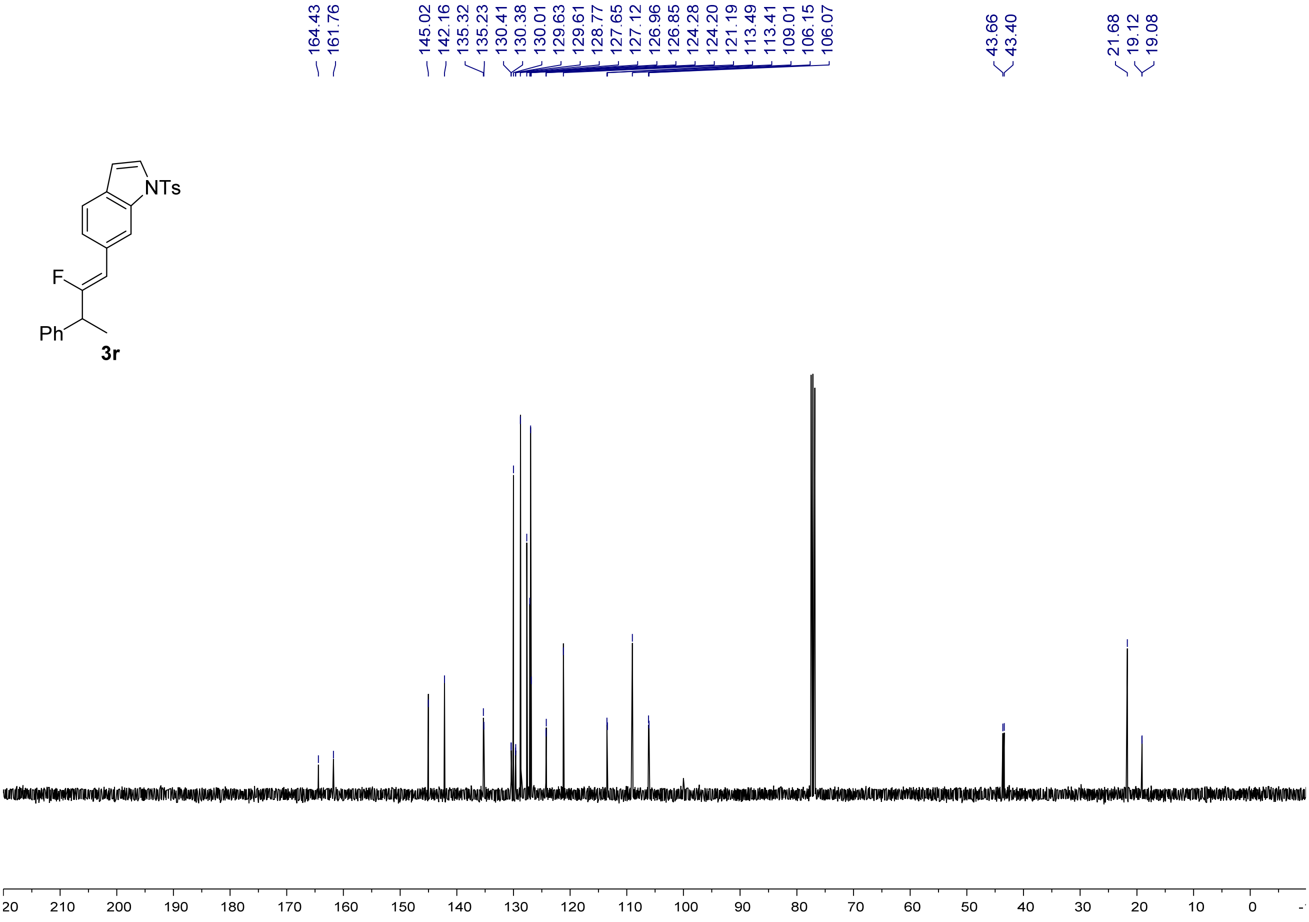
164.43
161.76
145.02
142.16
135.32
135.23
130.41
130.38
130.01
129.63
129.61
128.77
127.65
127.12
126.96
126.85
124.28
124.20
121.19
113.49
113.41
109.01
106.15
106.07
43.66
43.40
21.68
19.12
19.08
NTs
F
Ph
3r
20 210 200 190 180 170 160 150 140 130 120 110 100 90 80 70 60 50 40 30 20 10 0

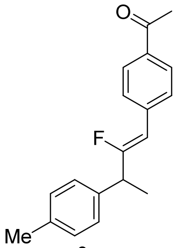

3s

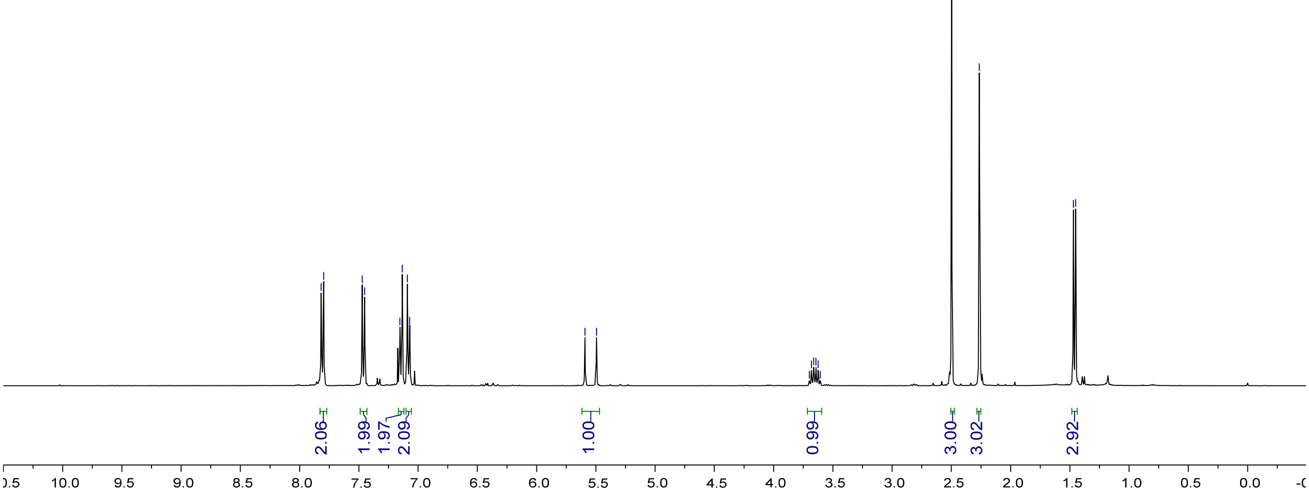
7.82 7.80 7.47 7.45 7.15 7.13 7.09 7.07 7.07
5.59 5.49
3.70 3.68 3.66 3.64 3.62 3.61
2.50 2.26
1.47 1.45
2.06 1.99 1.97 2.09 1.00 0.99 3.00 3.02 2.92
10.0 9.5 9.0 8.5 8.0 7.5 7.0 6.5 6.0 5.5 5.0 4.5 4.0 3.5 3.0 2.5 2.0 1.5 1.0 0.5 0.0

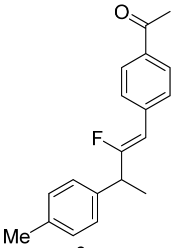

3s

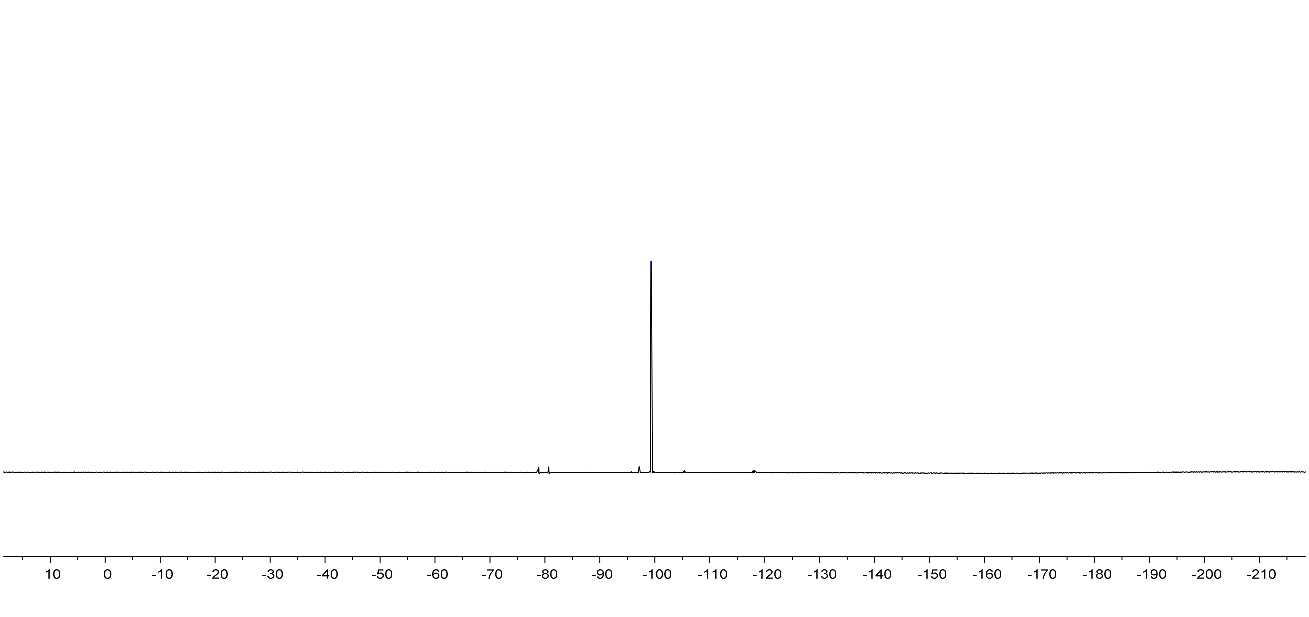
-99.28 -99.32 -99.38 -99.42
10 0 -10 -20 -30 -40 -50 -60 -70 -80 -90 -100 -110 -120 -130 -140 -150 -160 -170 -180 -190 -200 -210

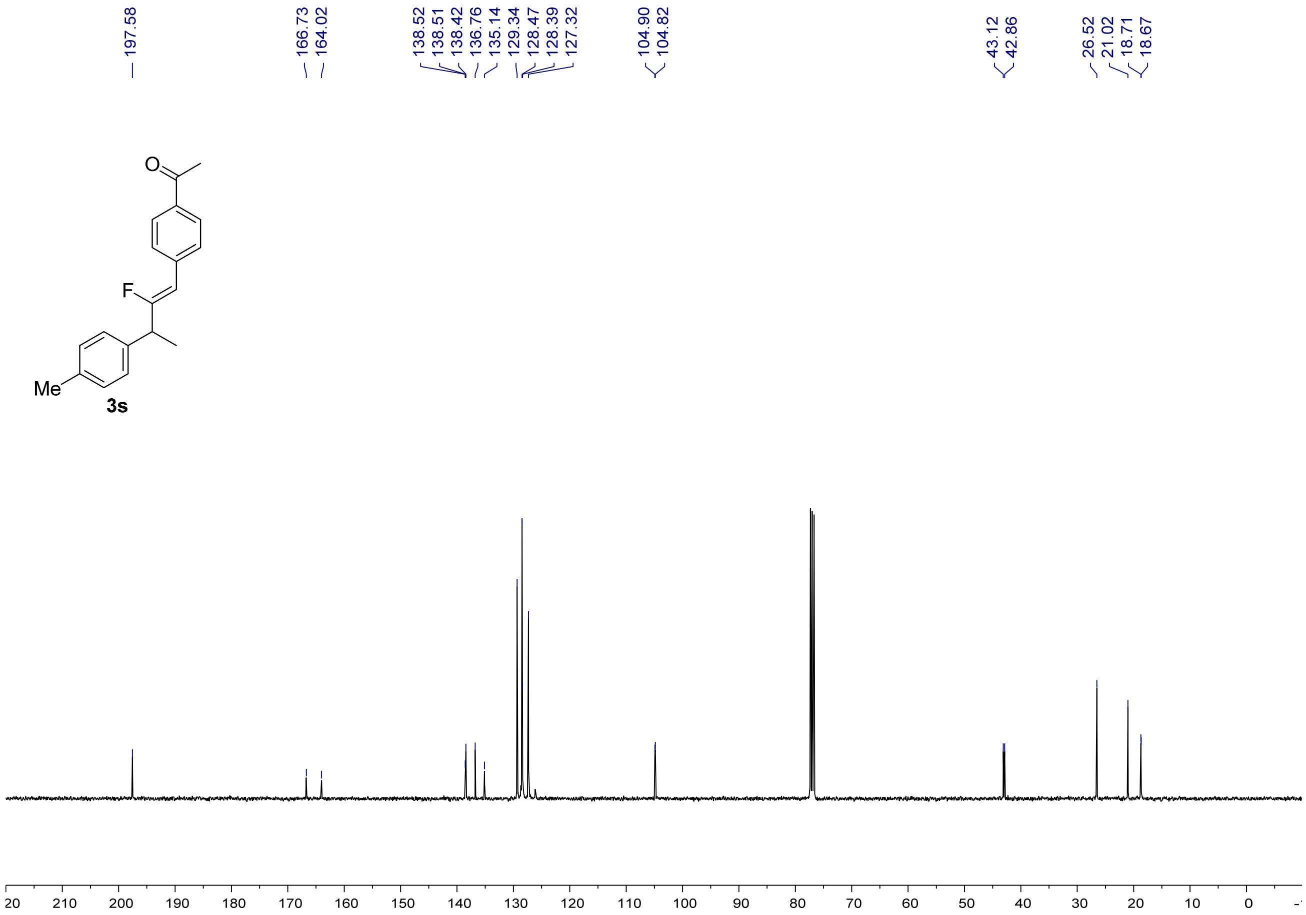
197.58
166.73
164.02
138.52
138.51
138.42
136.76
135.14
129.34
128.47
128.39
127.32
104.90
104.82
43.12
42.86
26.52
21.02
18.71
18.67
O
F
Me
3s

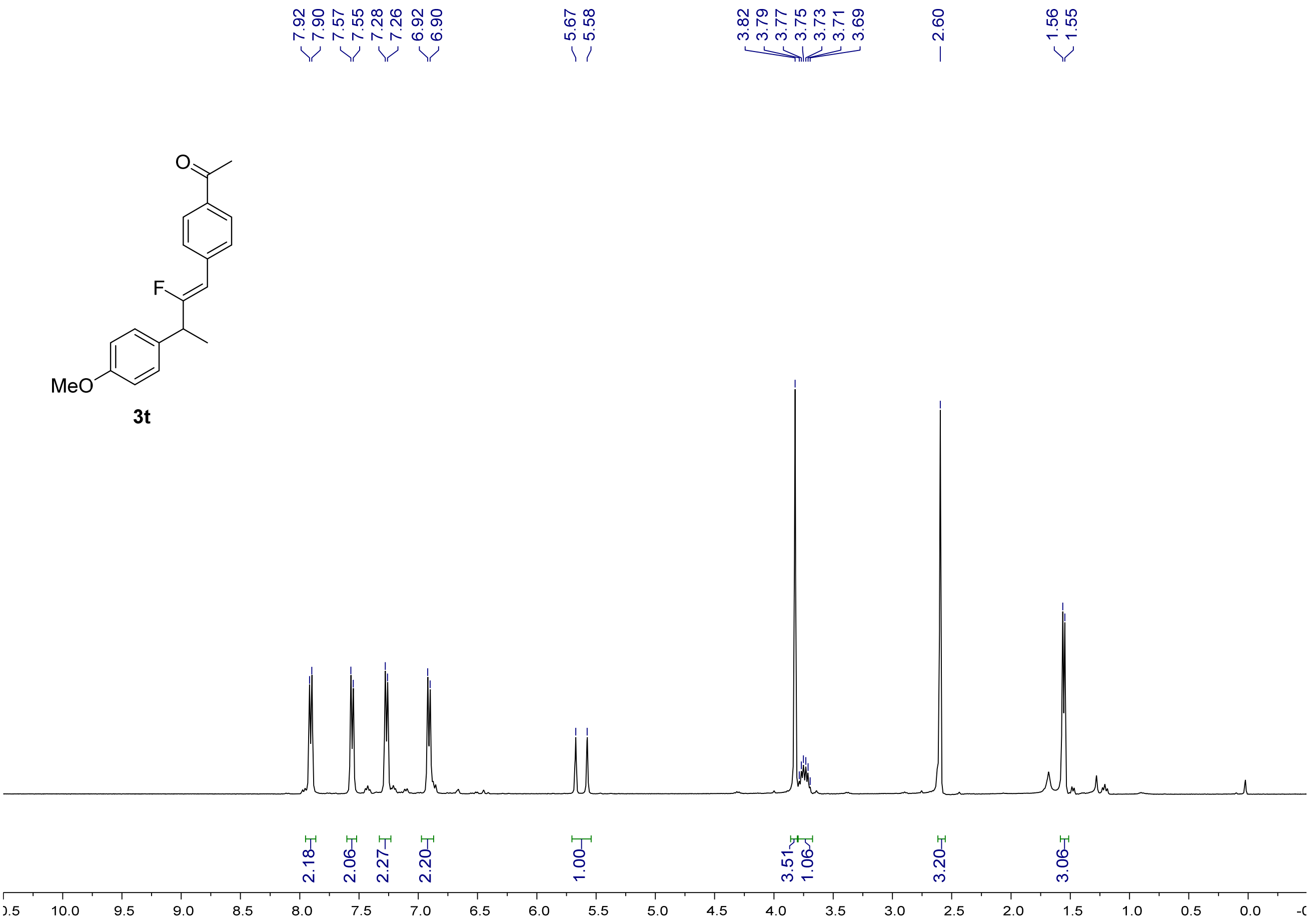
7.92
7.90
7.57
7.55
7.28
7.26
6.92
6.90
5.67
5.58
3.82
3.79
3.77
3.75
3.73
3.71
3.69
2.60
1.56
1.55
O
F
MeO
3t
2.18
2.06
2.27
2.20
1.00
3.51
1.06
3.20
3.06

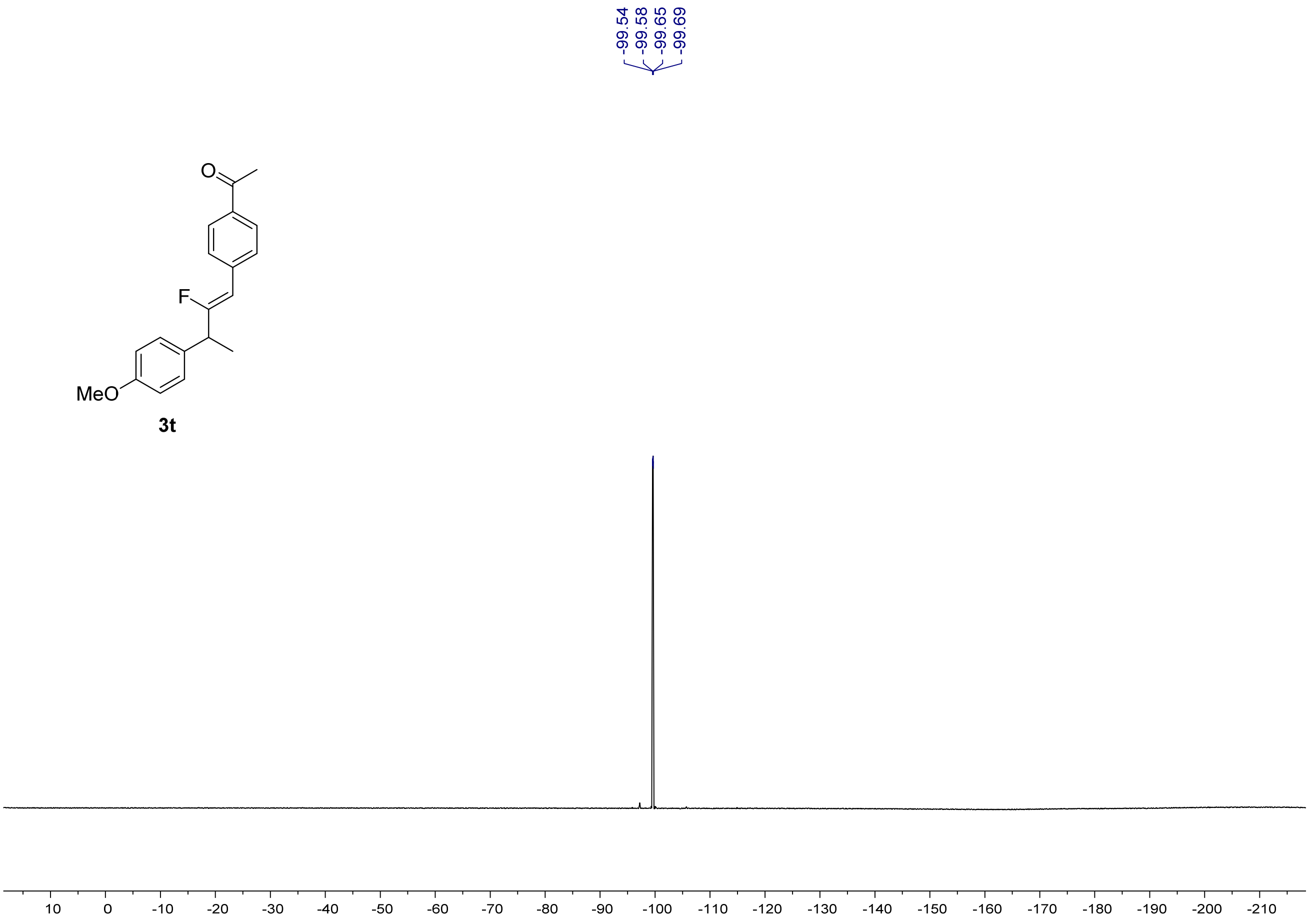
-99.54
-99.58
-99.65
-99.69
O
F
MeO
3t
10 0 -10 -20 -30 -40 -50 -60 -70 -80 -90 -100 -110 -120 -130 -140 -150 -160 -170 -180 -190 -200 -210

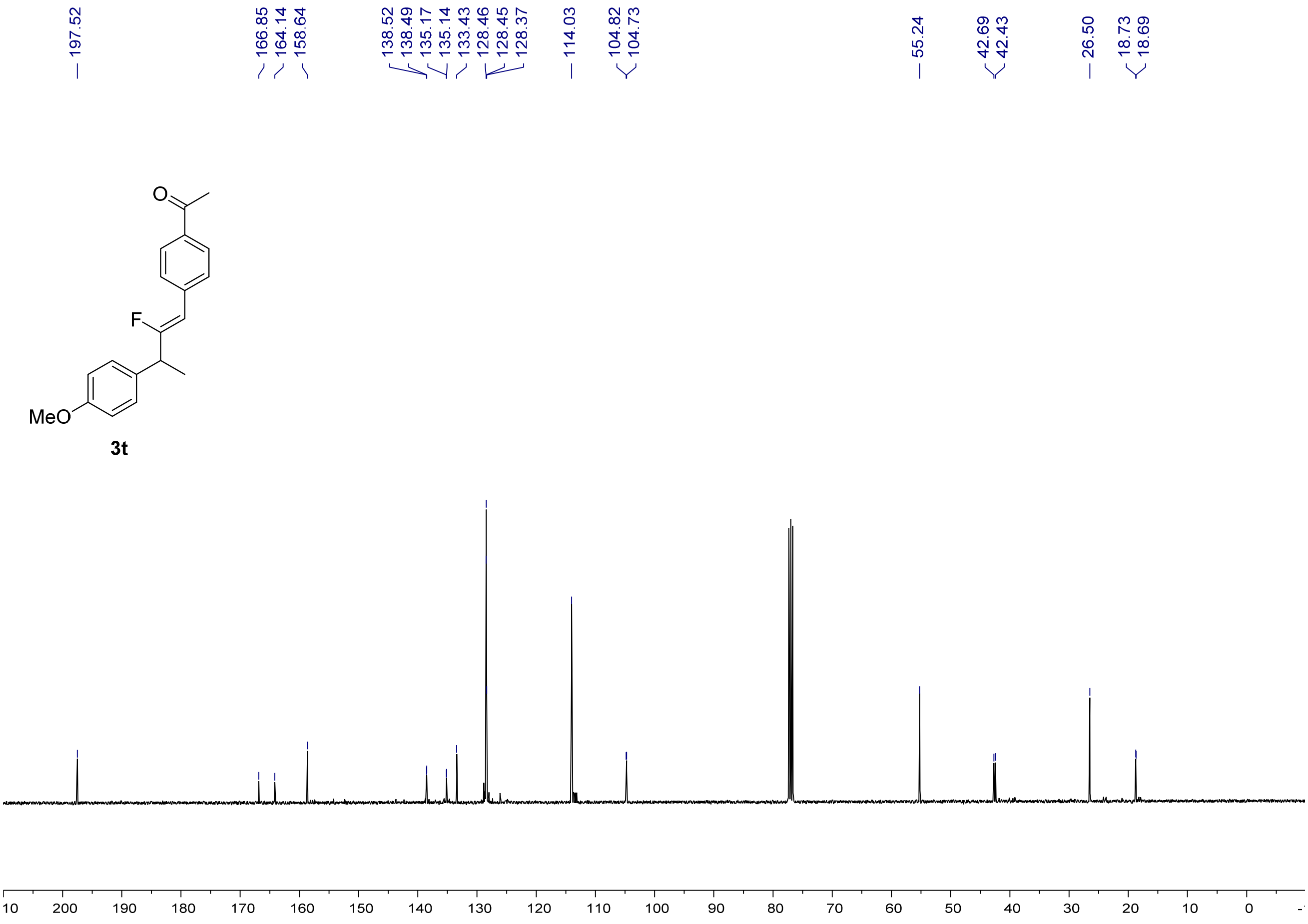
197.52
166.85
164.14
158.64
138.52
138.49
135.17
135.14
133.43
128.46
128.45
128.37
114.03
104.82
104.73
55.24
42.69
42.43
26.50
18.73
18.69
O
F
MeO
3t
10 200 190 180 170 160 150 140 130 120 110 100 90 80 70 60 50 40 30 20 10 0

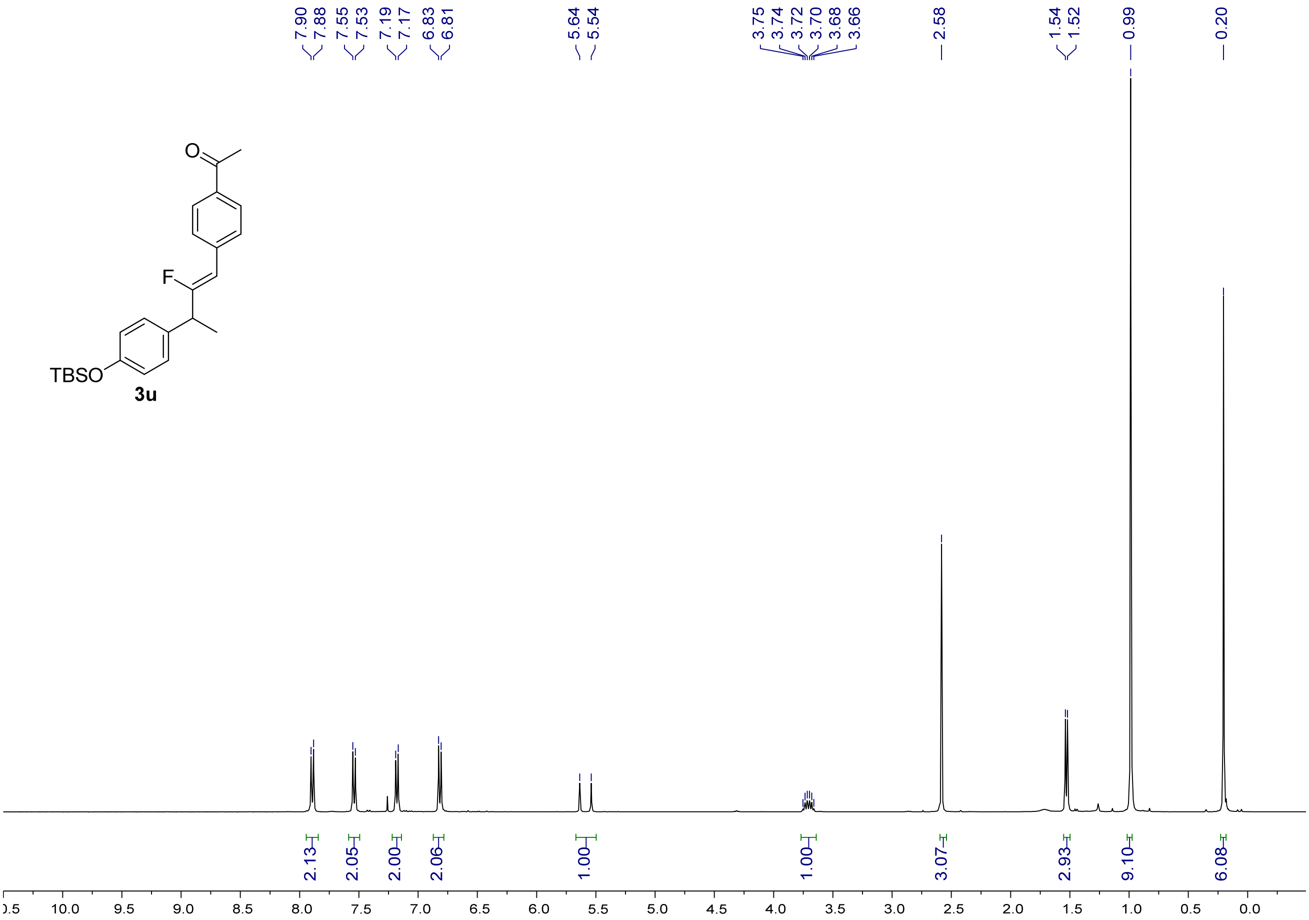
7.90
7.88
7.55
7.53
7.19
7.17
6.83
6.81
5.64
5.54
3.75
3.74
3.72
3.70
3.68
3.66
2.58
1.54
1.52
0.99
0.20
O
F
TBSO
3u
2.13
2.05
2.00
2.06
1.00
1.00
3.07
2.93
9.10
6.08
10.0 9.5 9.0 8.5 8.0 7.5 7.0 6.5 6.0 5.5 5.0 4.5 4.0 3.5 3.0 2.5 2.0 1.5 1.0 0.5 0.0

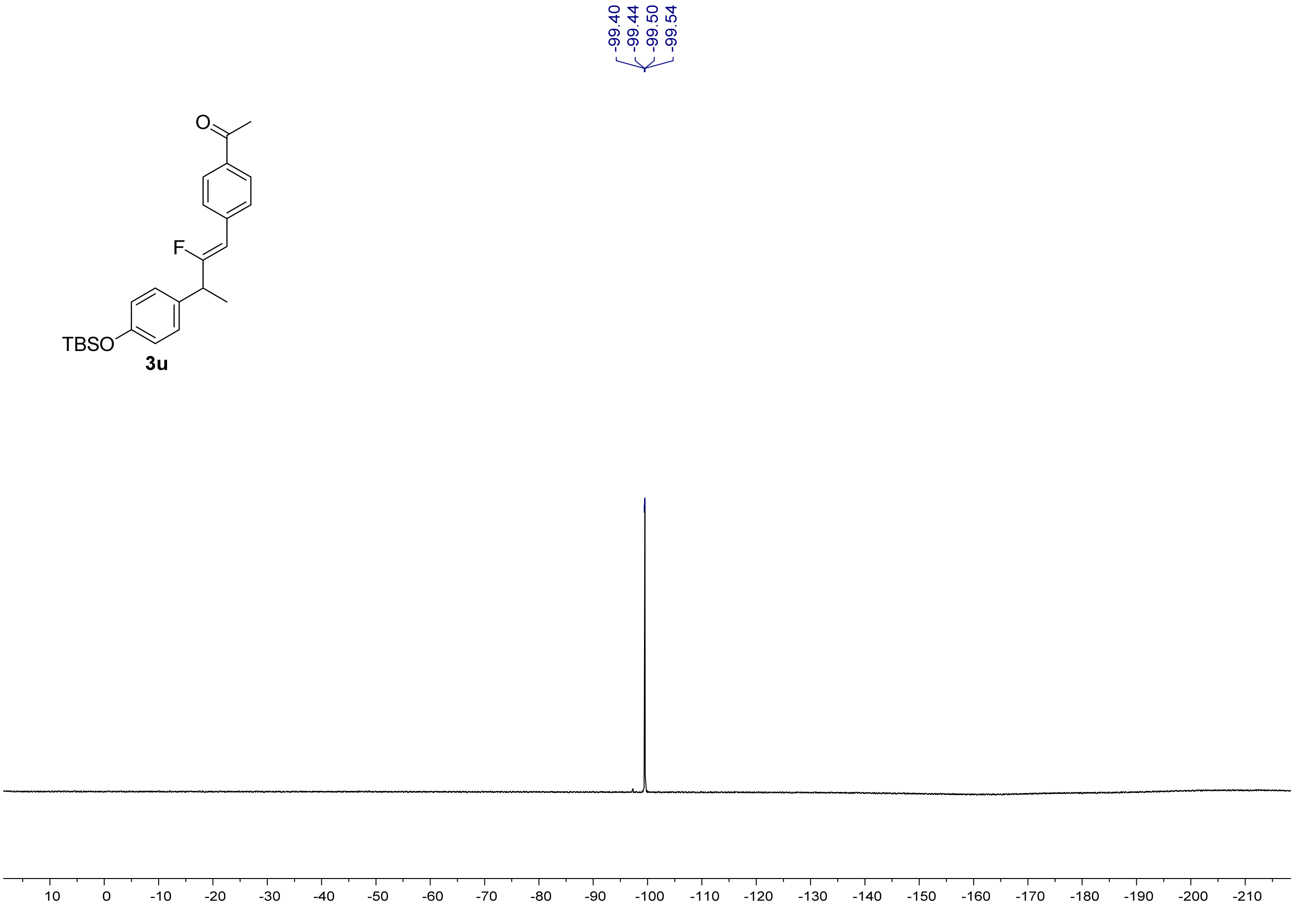
-99.40
-99.44
-99.50
-99.54
O
F
TBSO
3u
10 0 -10 -20 -30 -40 -50 -60 -70 -80 -90 -100 -110 -120 -130 -140 -150 -160 -170 -180 -190 -200 -210

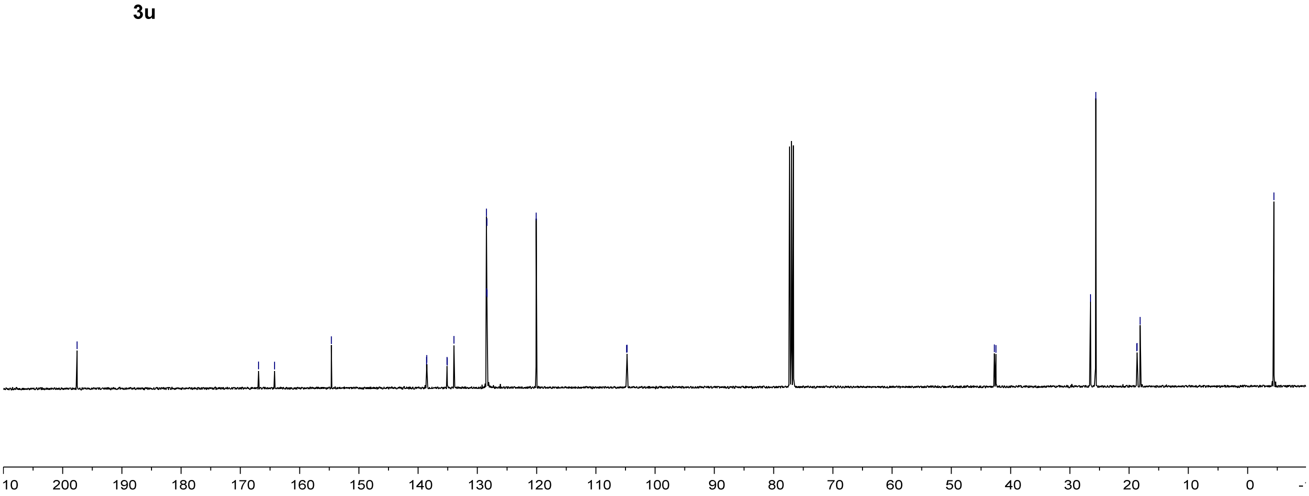
197.57
166.94
164.23
154.64
138.57
138.54
135.11
135.08
133.95
128.48
128.44
128.39
128.36
120.07
104.81
104.73
42.73
42.48
26.53
25.63
18.70
18.67
18.15
-4.44

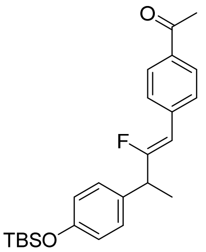
TBSO
F
O

3u

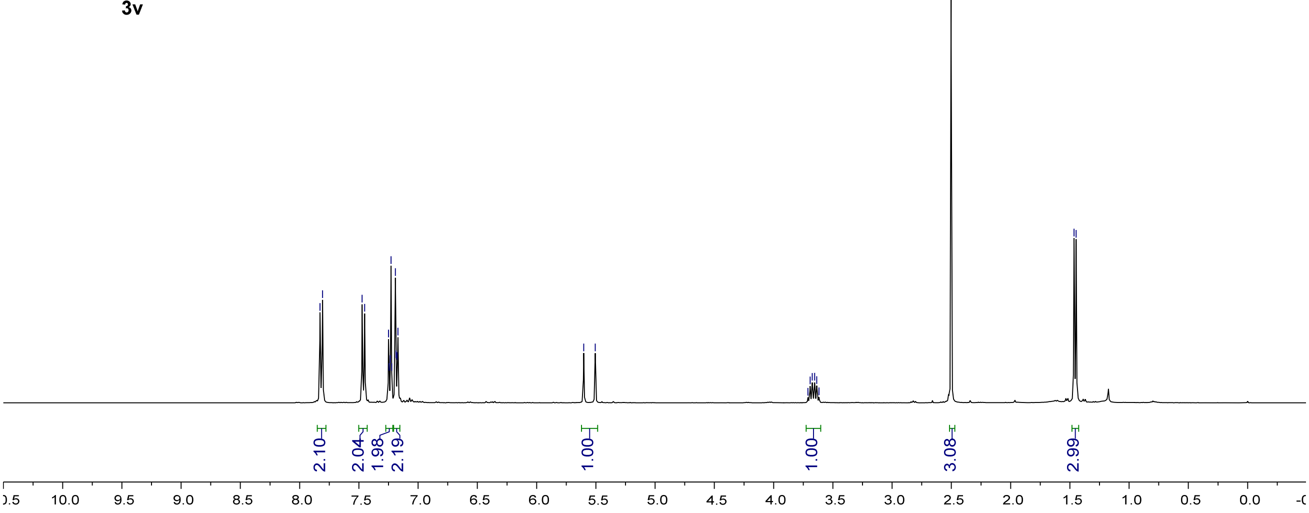
7.83
7.81
7.47
7.45
7.25
7.25
7.23
7.23
7.22
7.19
7.19
7.18
7.17
5.60
5.51
3.71
3.69
3.67
3.65
3.64
3.62
2.50
1.47
1.45
2.10
2.04
1.98
2.19
1.00
1.00
3.08
2.99

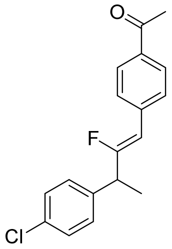
Cl
F
O

3v

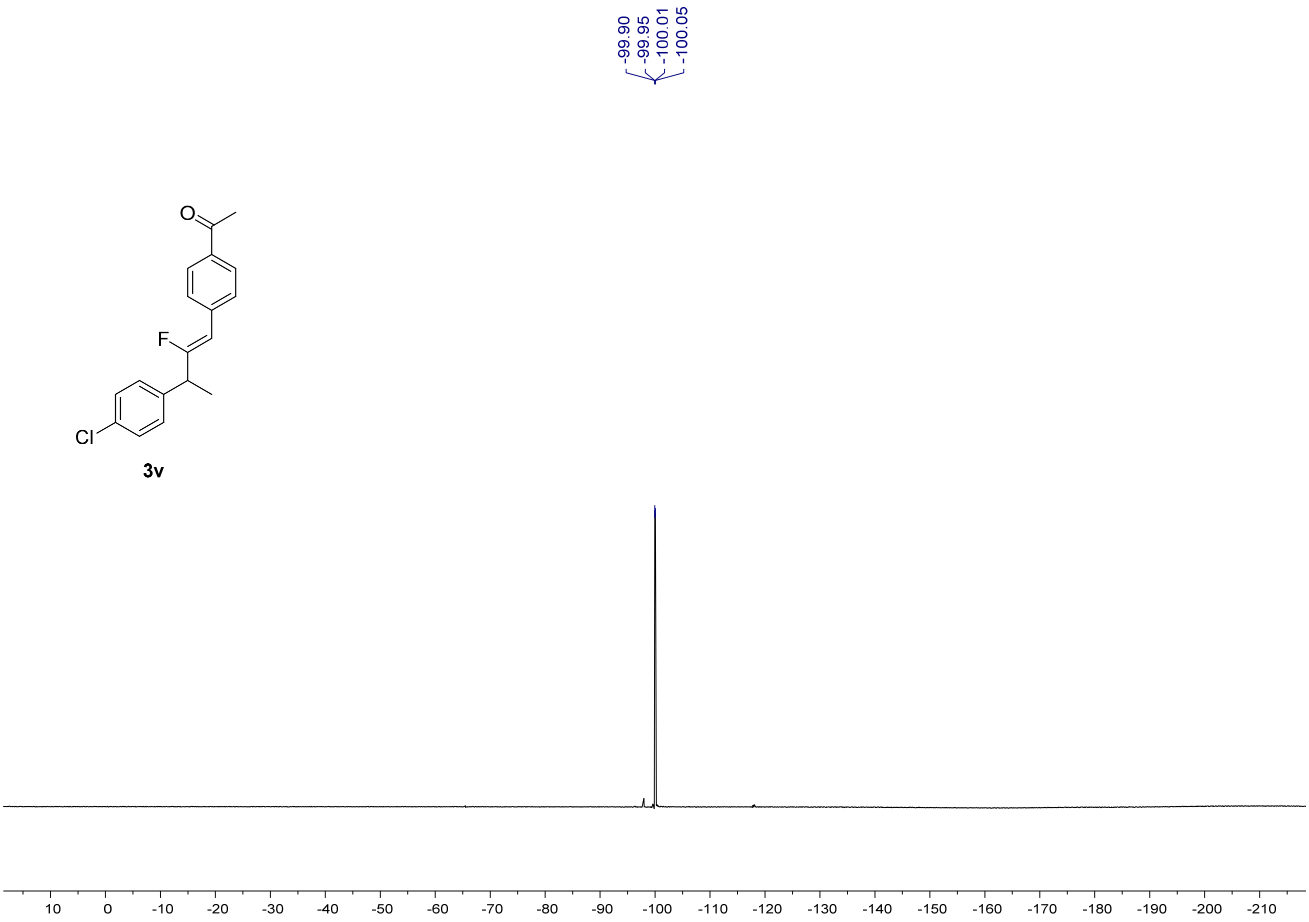

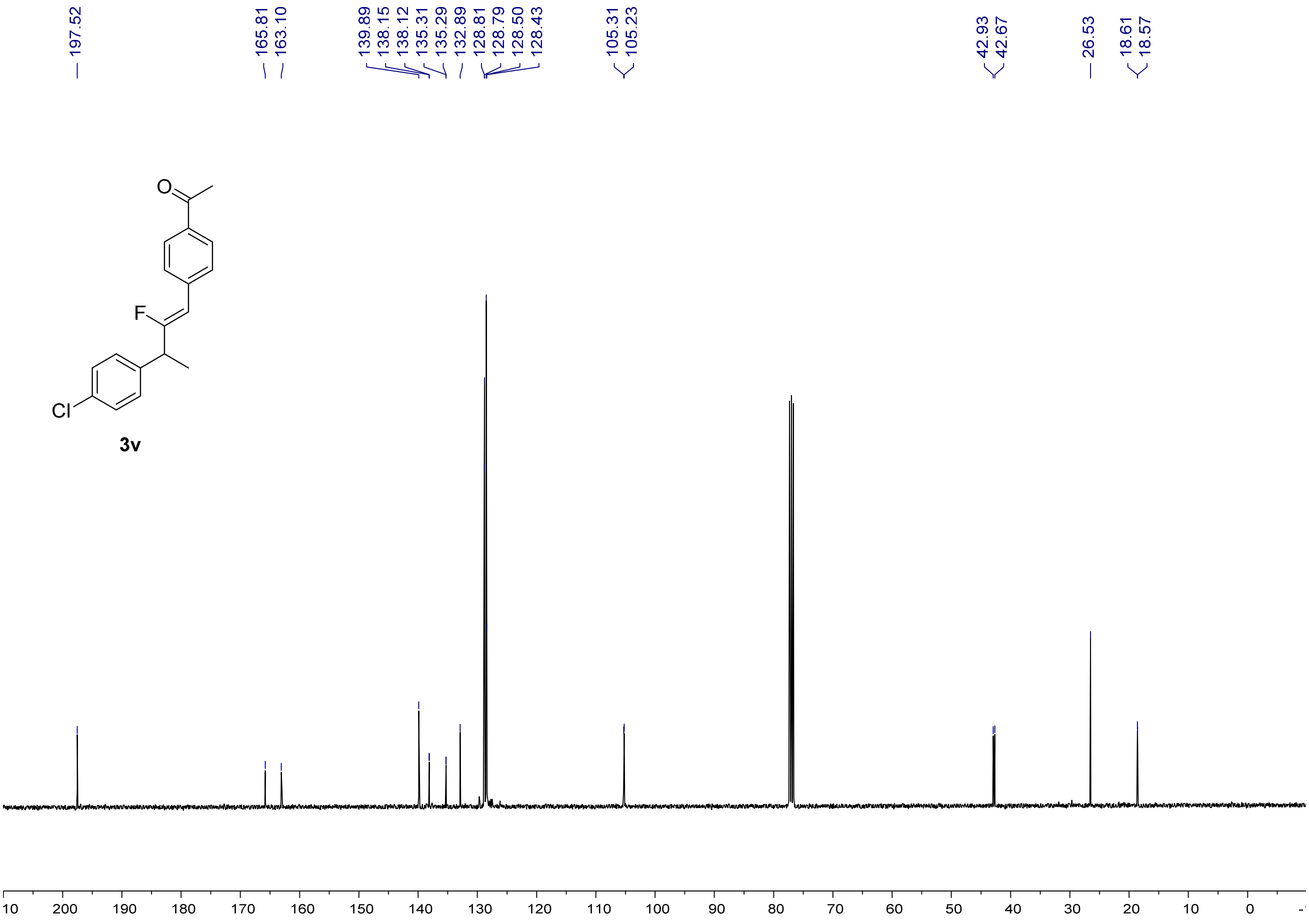

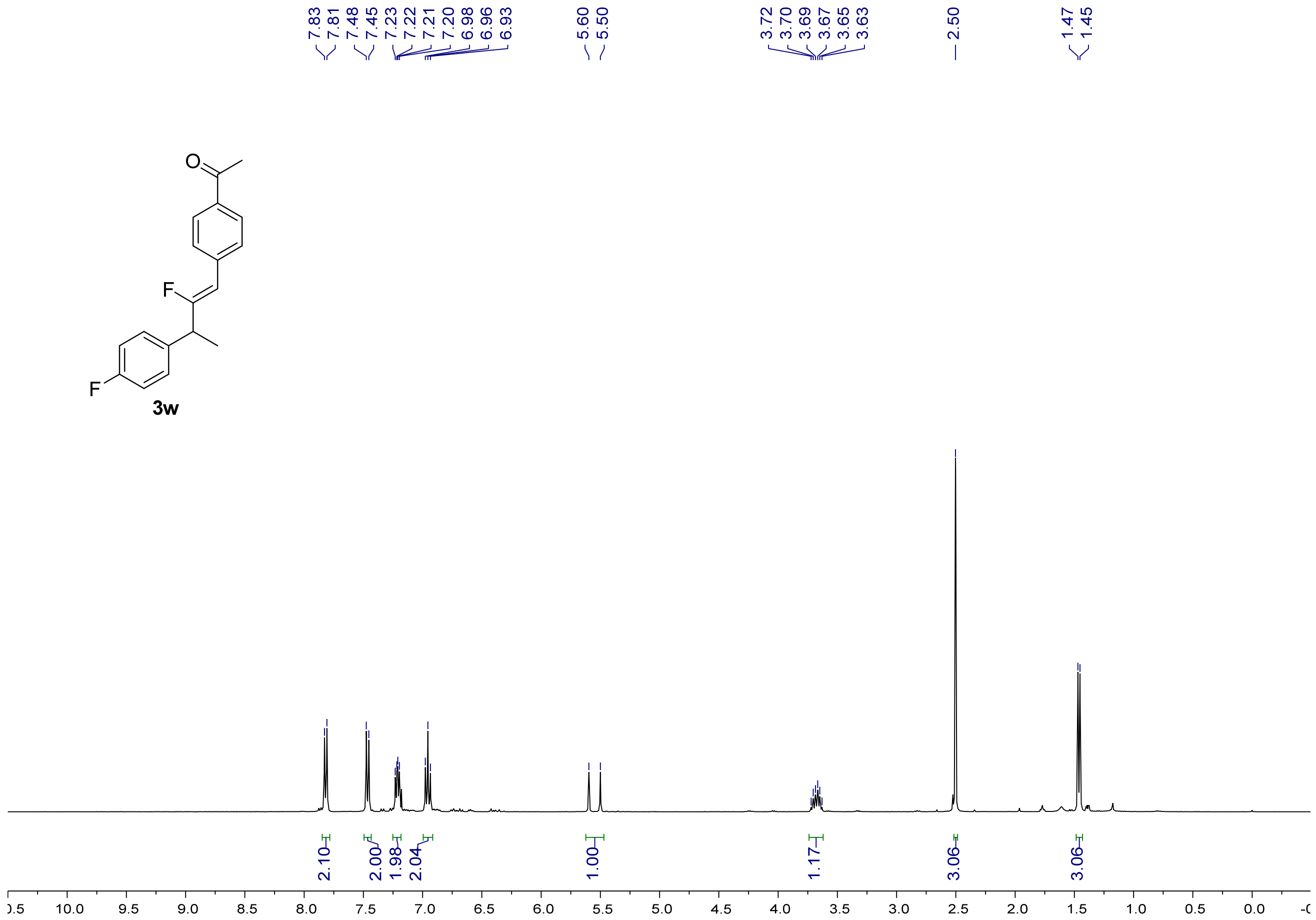
7.83
7.81
7.48
7.45
7.23
7.22
7.21
7.20
6.98
6.96
6.93
5.60
5.50
3.72
3.70
3.69
3.67
3.65
3.63
2.50
1.47
1.45
O
F
F
3w
2.10
2.00
1.98
2.04
1.00
1.17
3.06
3.06
10.0
9.5
9.0
8.5
8.0
7.5
7.0
6.5
6.0
5.5
5.0
4.5
4.0
3.5
3.0
2.5
2.0
1.5
1.0
0.5
0.0

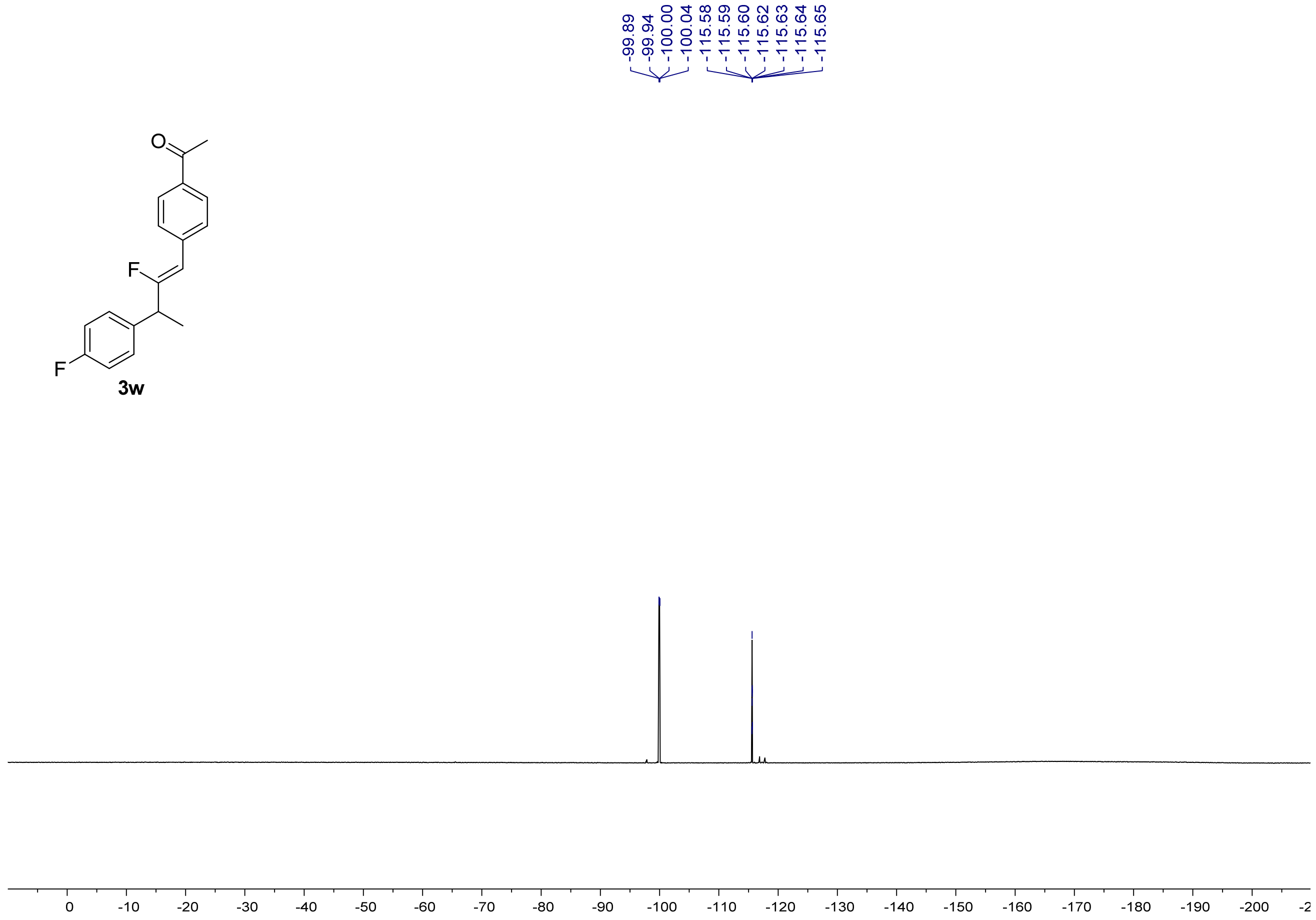
-99.89
-99.94
-100.00
-100.04
-115.58
-115.59
-115.60
-115.62
-115.63
-115.64
-115.65
O
F
F
3w
0
-10
-20
-30
-40
-50
-60
-70
-80
-90
-100
-110
-120
-130
-140
-150
-160
-170
-180
-190
-200

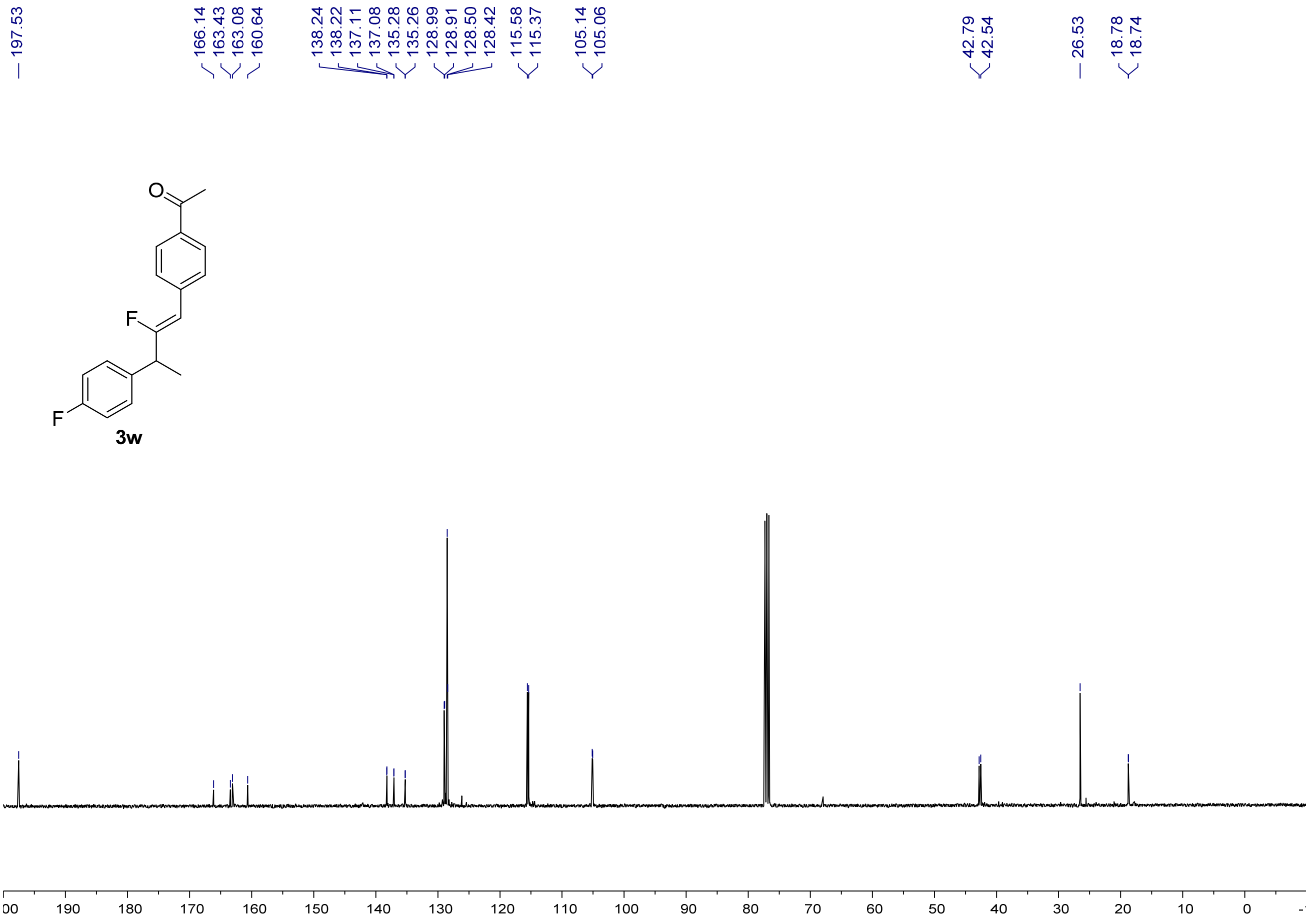

197.53
166.14
163.43
163.08
160.64
138.24
138.22
137.11
137.08
135.28
135.26
128.99
128.91
128.50
128.42
115.58
115.37
105.14
105.06
42.79
42.54
26.53
18.78
18.74
O
F
F

3w

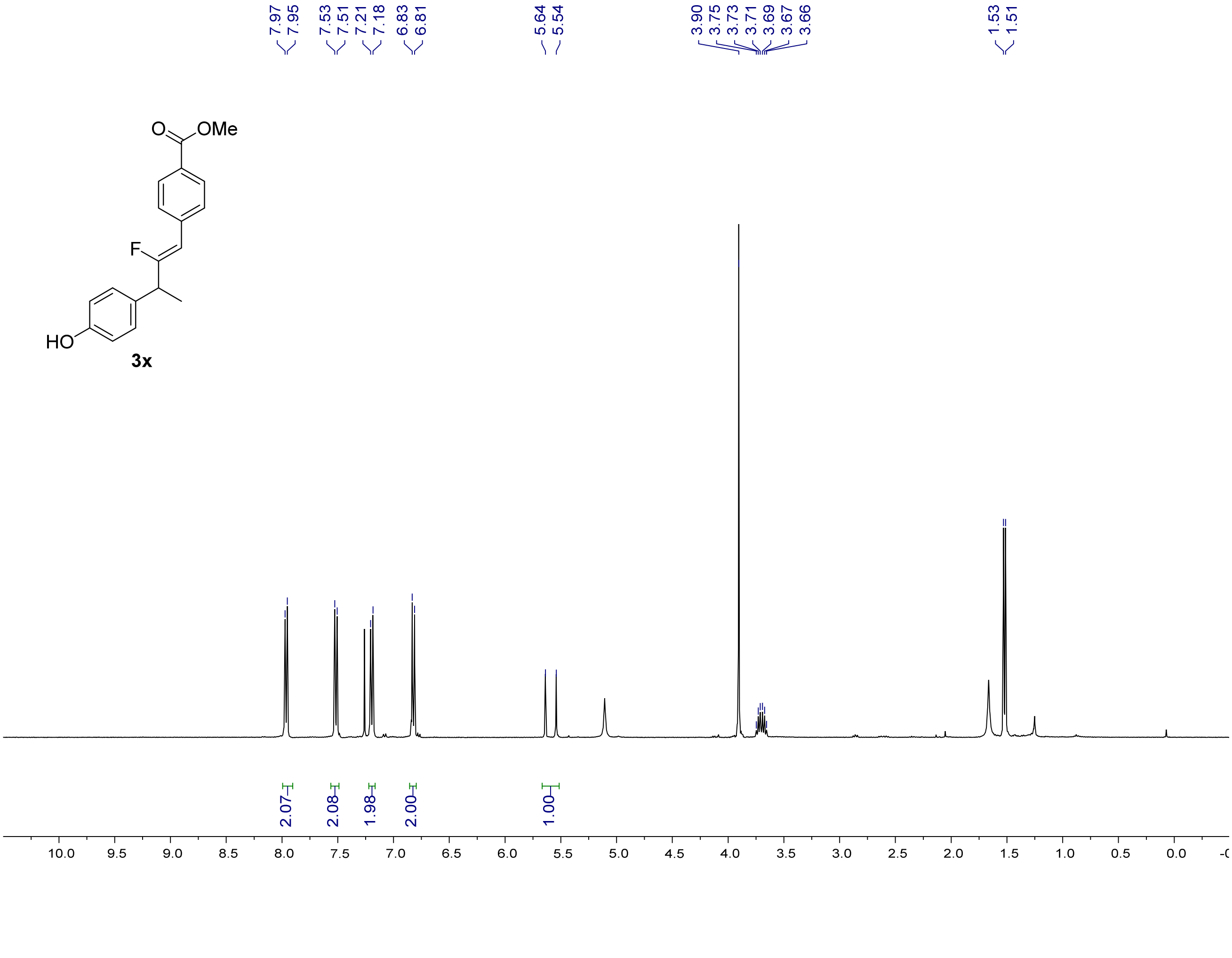

7.97
7.95
7.53
7.51
7.21
7.18
6.83
6.81
5.64
5.54
3.90
3.75
3.73
3.71
3.69
3.67
3.66
1.53
1.51
O
OMe
F
HO
2.07
2.08
1.98
2.00
1.00

3x

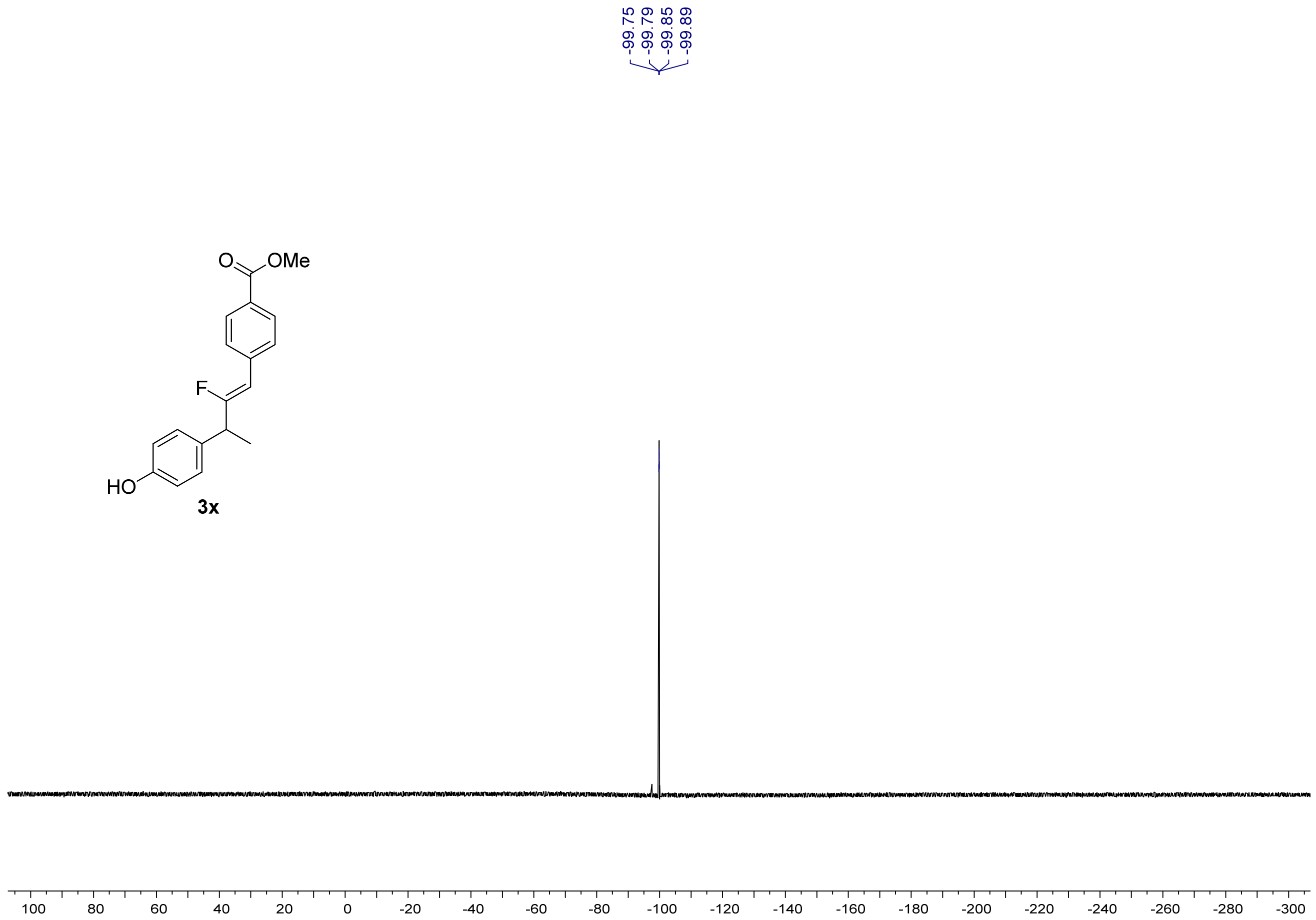
-99.75
-99.79
-99.85
-99.89
O
OMe
F
HO
3x

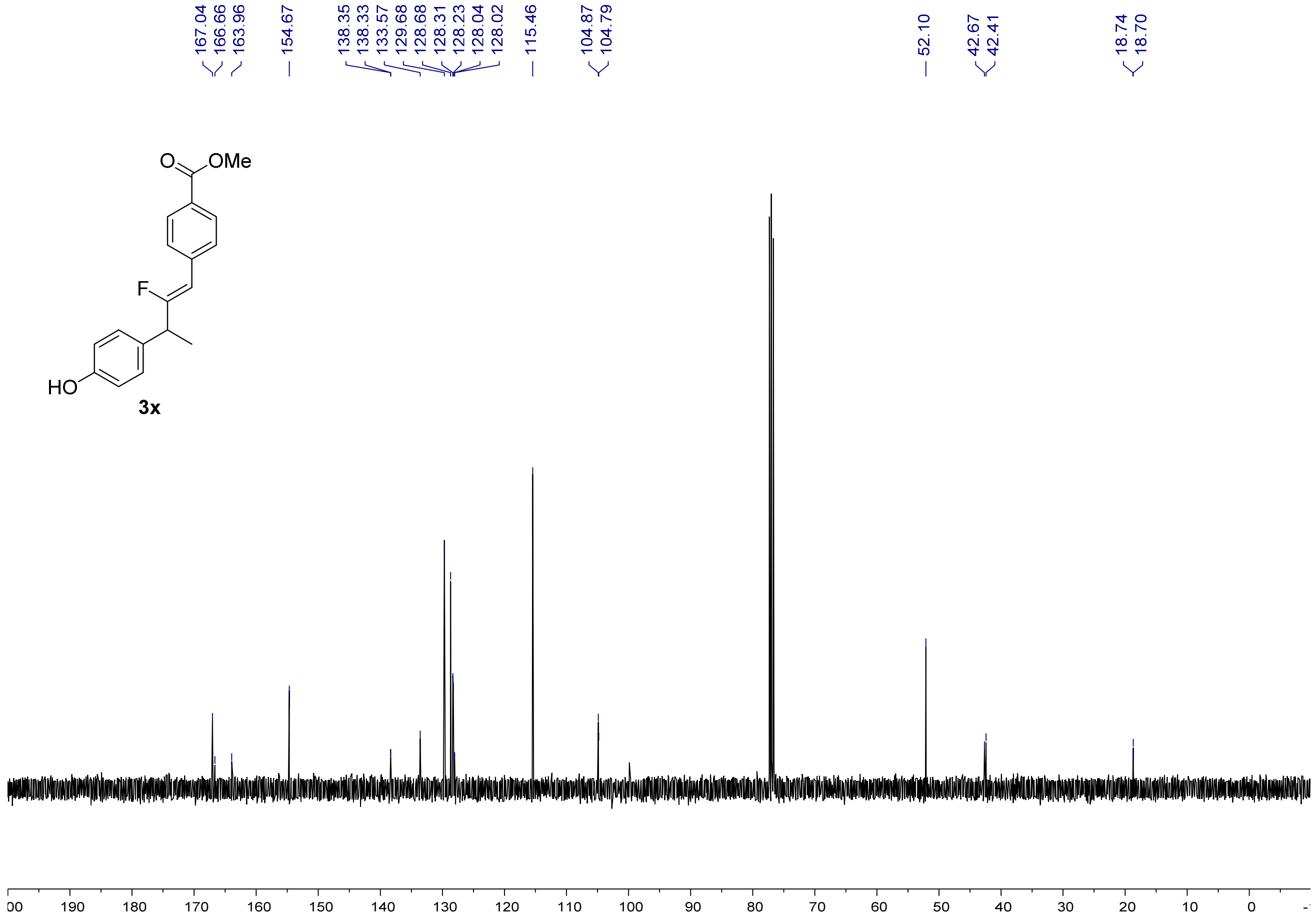
167.04
166.66
163.96
154.67
138.35
138.33
133.57
129.68
128.68
128.31
128.23
128.04
128.02
115.46
104.87
104.79
52.10
42.67
42.41
18.74
18.70
O
OMe
F
HO
3x

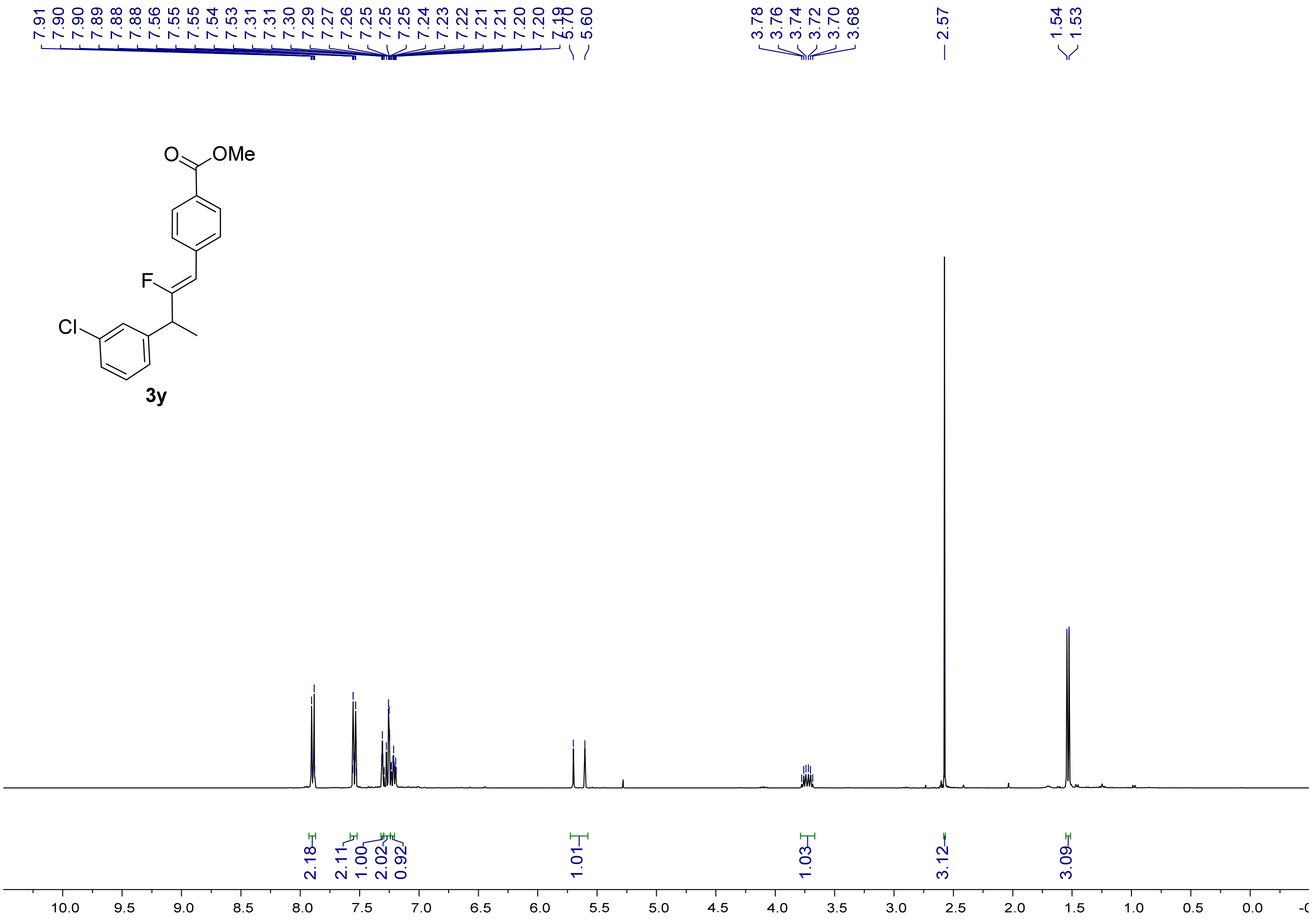
OMe
F
Cl
3y
7.91 7.90 7.90 7.89 7.88 7.88 7.56 7.55 7.55 7.54 7.53 7.31 7.31 7.30 7.29 7.27 7.26 7.25 7.25 7.25 7.24 7.23 7.22 7.21 7.21 7.20 7.20 [illegible] 5.70 5.60
3.78 3.76 3.74 3.72 3.70 3.68
2.57
1.54 1.53
2.18 2.11 1.00 2.02 0.92 1.01 1.03 3.12 3.09

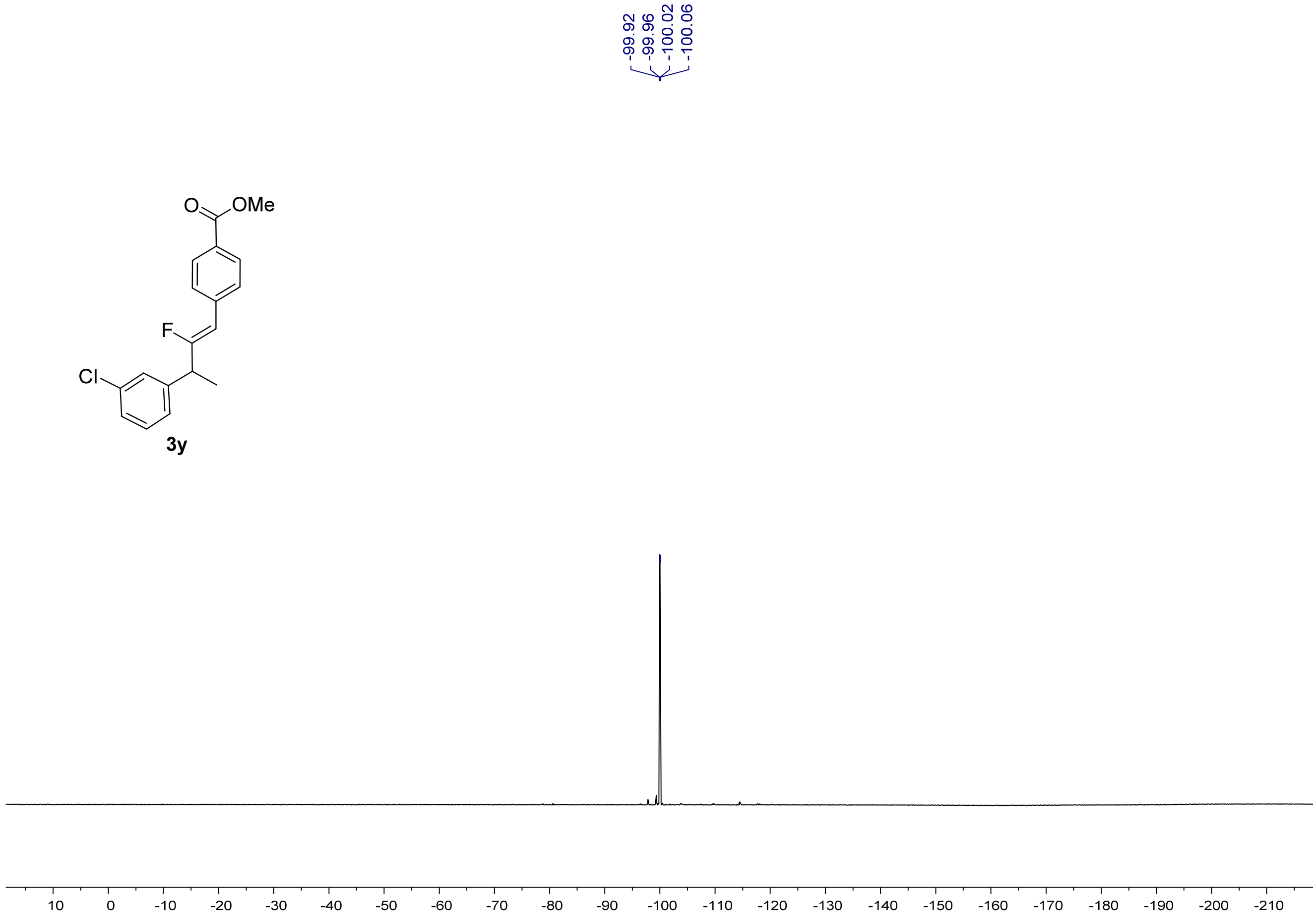
OMe
F
Cl
3y
-99.92 -99.96 -100.02 -100.06

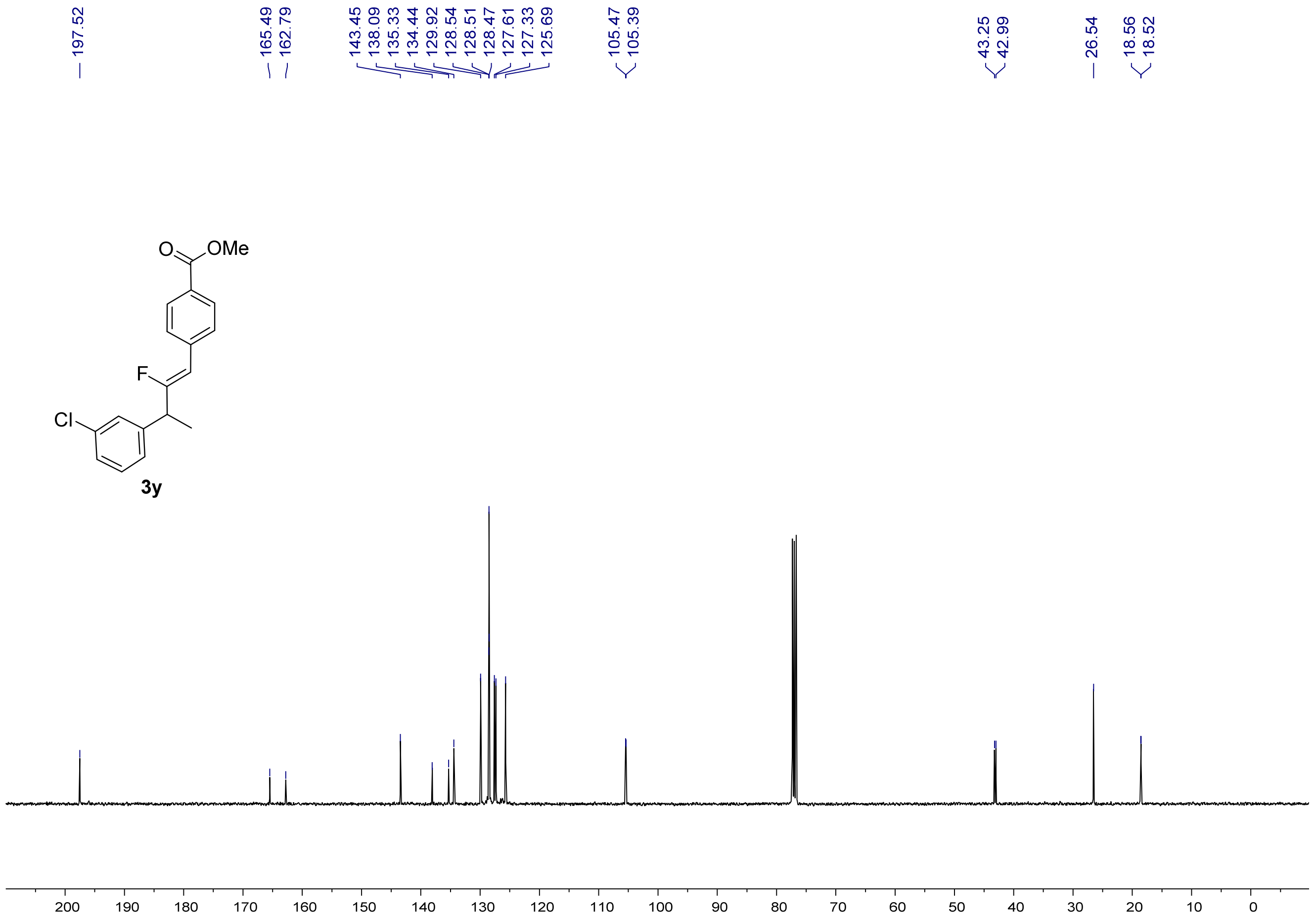
197.52
165.49
162.79
143.45
138.09
135.33
134.44
129.92
128.54
128.51
128.47
127.61
127.33
125.69
105.47
105.39
43.25
42.99
26.54
18.56
18.52
OMe
O
F
Cl
3y
200 190 180 170 160 150 140 130 120 110 100 90 80 70 60 50 40 30 20 10 0

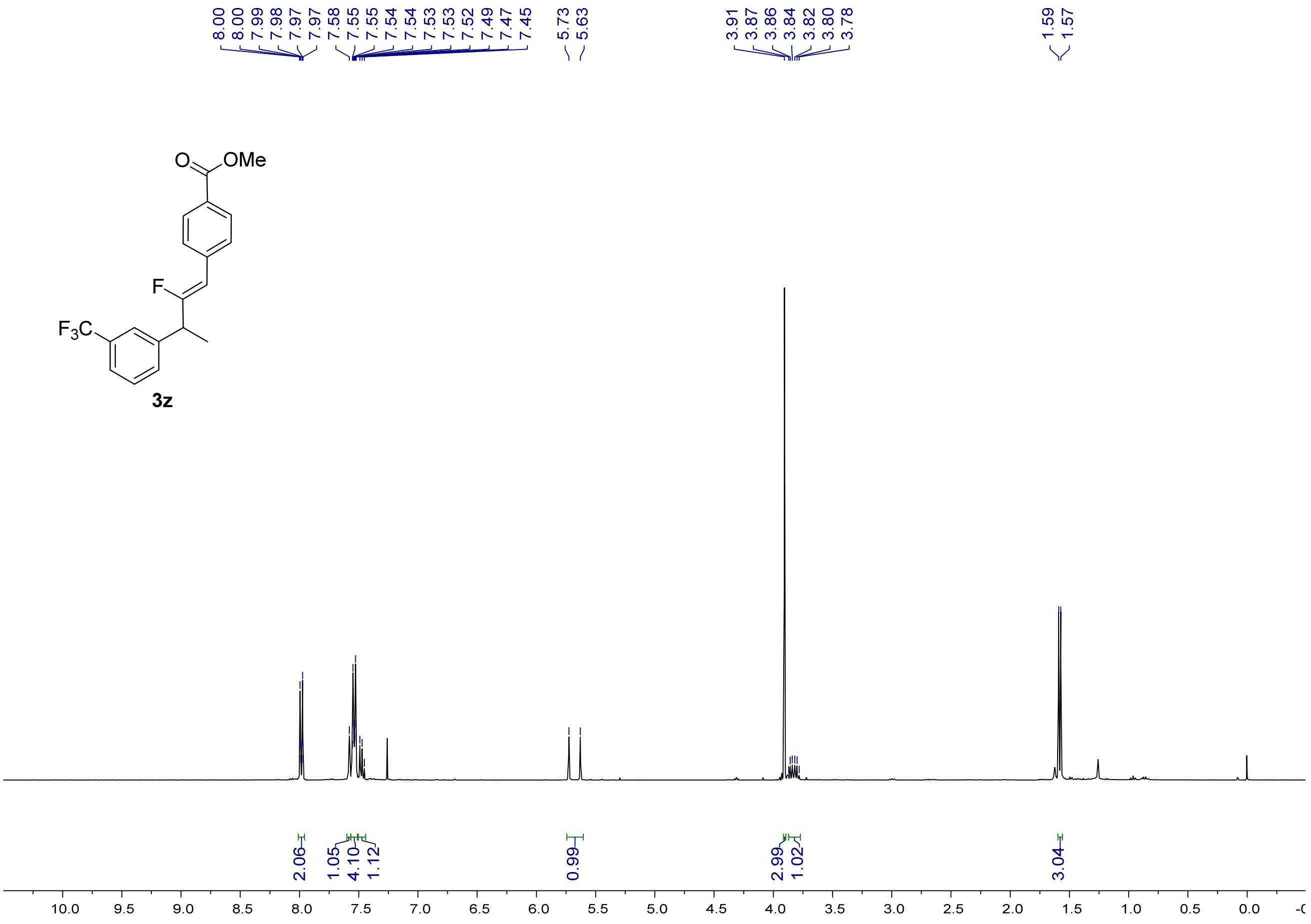
8.00
8.00
7.99
7.98
7.97
7.97
7.58
7.55
7.55
7.54
7.54
7.53
7.53
7.52
7.49
7.47
7.45
5.73
5.63
3.91
3.87
3.86
3.84
3.82
3.80
3.78
1.59
1.57
OMe
O
F
F_3C
3z
2.06
1.05
4.10
1.12
0.99
2.99
1.02
3.04
10.0 9.5 9.0 8.5 8.0 7.5 7.0 6.5 6.0 5.5 5.0 4.5 4.0 3.5 3.0 2.5 2.0 1.5 1.0 0.5 0.0

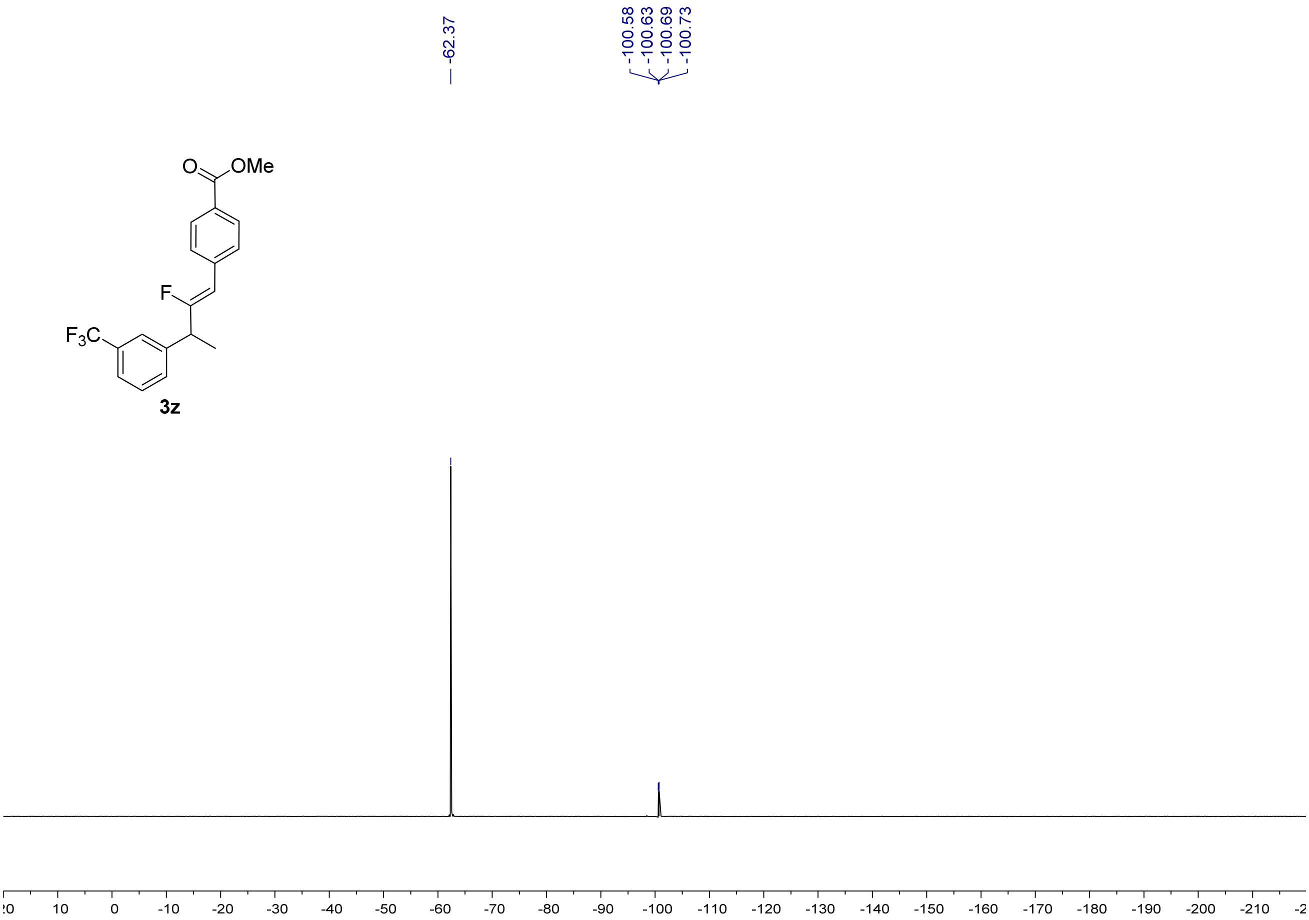
-62.37
-100.58
-100.63
-100.69
-100.73
O
OMe
F
F3C
3z
10 0 -10 -20 -30 -40 -50 -60 -70 -80 -90 -100 -110 -120 -130 -140 -150 -160 -170 -180 -190 -200 -210

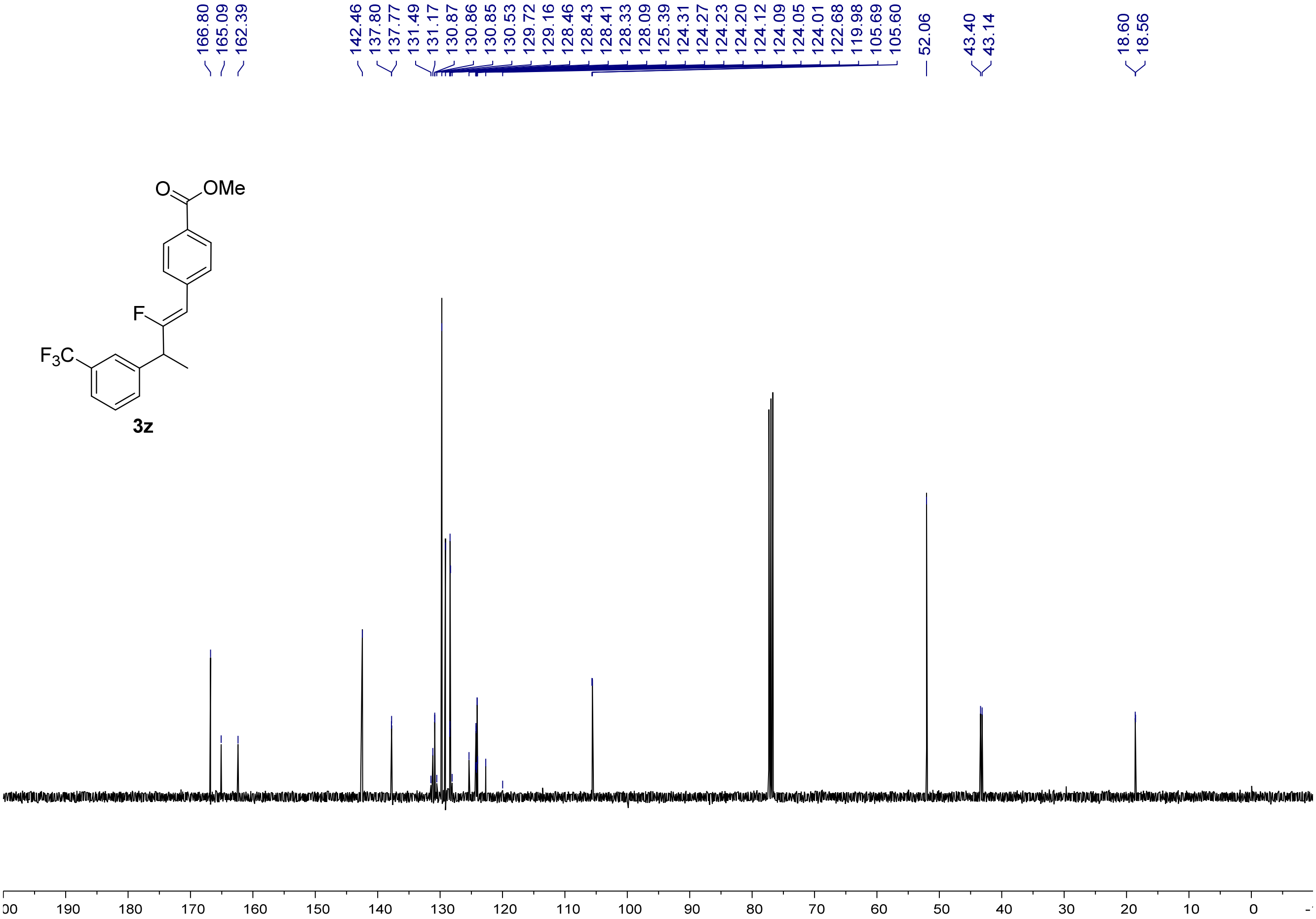
166.80
165.09
162.39
142.46
137.80
137.77
131.49
131.17
130.87
130.86
130.85
130.53
129.72
129.16
128.46
128.43
128.41
128.33
128.09
125.39
124.31
124.27
124.23
124.20
124.12
124.09
124.05
124.01
122.68
119.98
105.69
105.60
52.06
43.40
43.14
18.60
18.56
O
OMe
F
F3C
3z
190 180 170 160 150 140 130 120 110 100 90 80 70 60 50 40 30 20 10 0

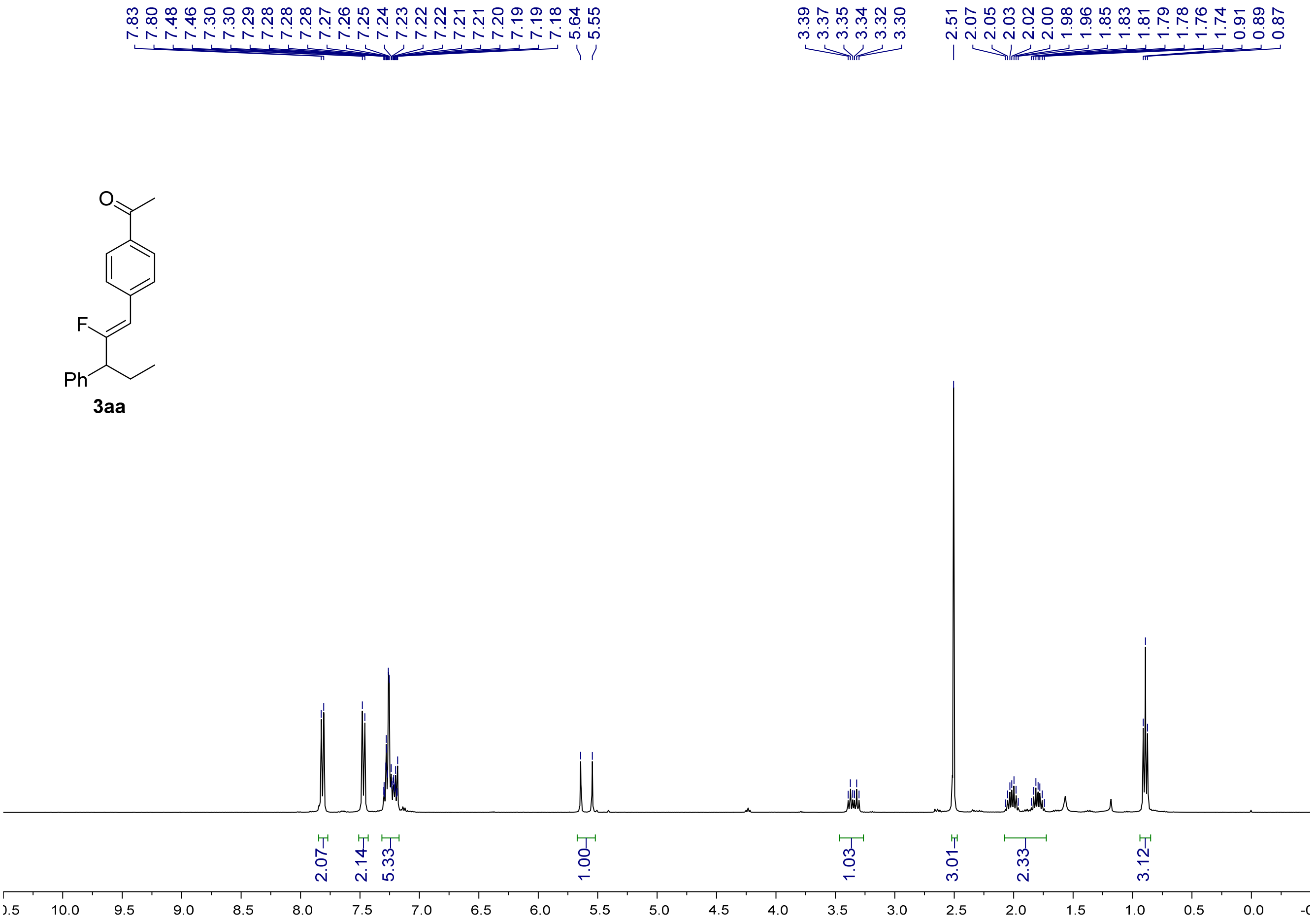
7.83 7.80 7.48 7.46 7.30 7.29 7.28 7.28 7.28 7.27 7.26 7.25 7.24 7.23 7.22 7.22 7.21 7.21 7.20 7.19 7.18 5.64 5.55
3.39 3.37 3.35 3.34 3.32 3.30
2.51 2.07 2.05 2.03 2.02 2.00 1.98 1.96 1.85 1.83 1.81 1.79 1.78 1.76 1.74 0.91 0.89 0.87
O
F
Ph
3aa
2.07 2.14 5.33 1.00 1.03 3.01 2.33 3.12
10.0 9.5 9.0 8.5 8.0 7.5 7.0 6.5 6.0 5.5 5.0 4.5 4.0 3.5 3.0 2.5 2.0 1.5 1.0 0.5 0.0

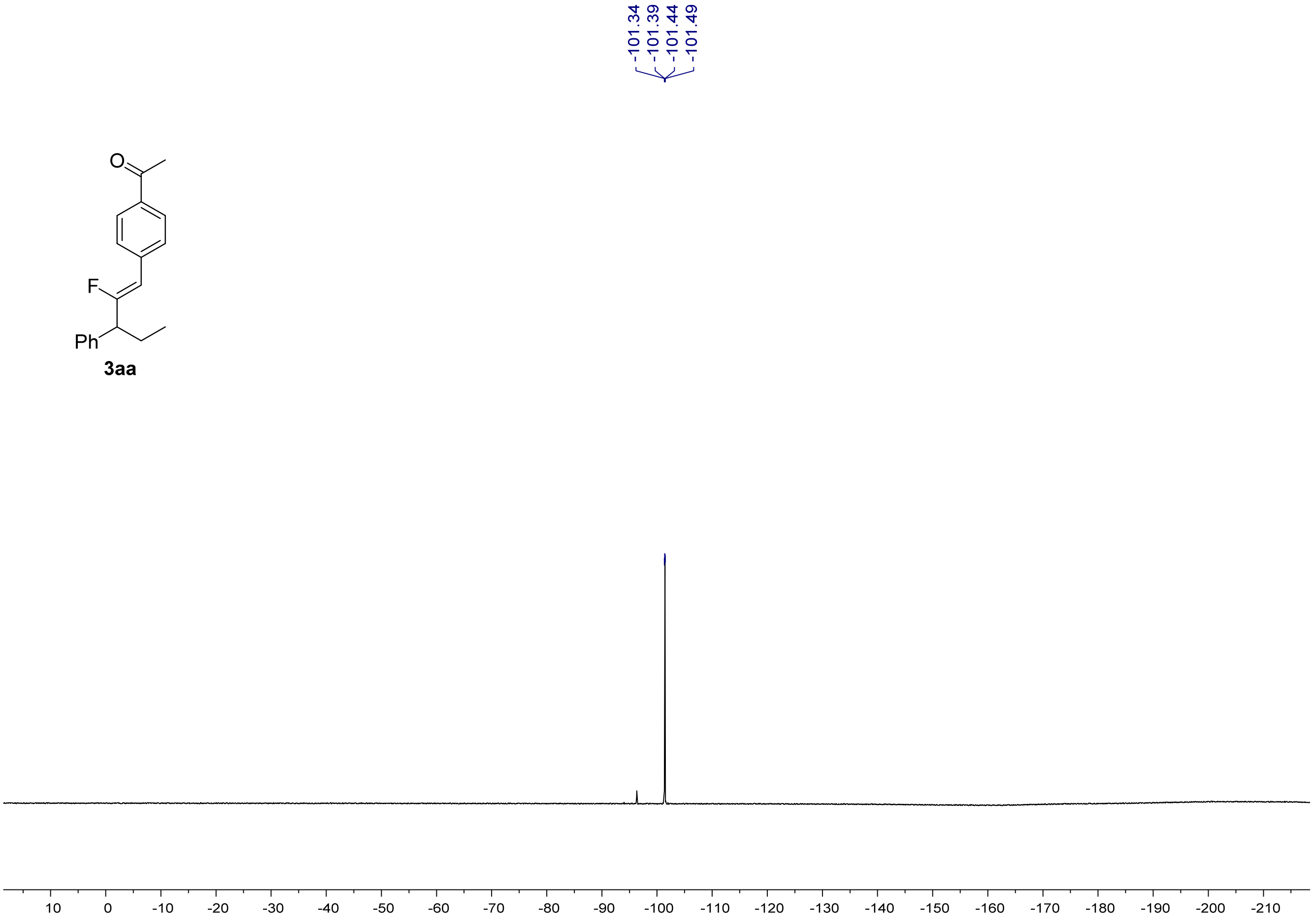
-101.34 -101.39 -101.44 -101.49
O
F
Ph
3aa
10 0 -10 -20 -30 -40 -50 -60 -70 -80 -90 -100 -110 -120 -130 -140 -150 -160 -170 -180 -190 -200 -210

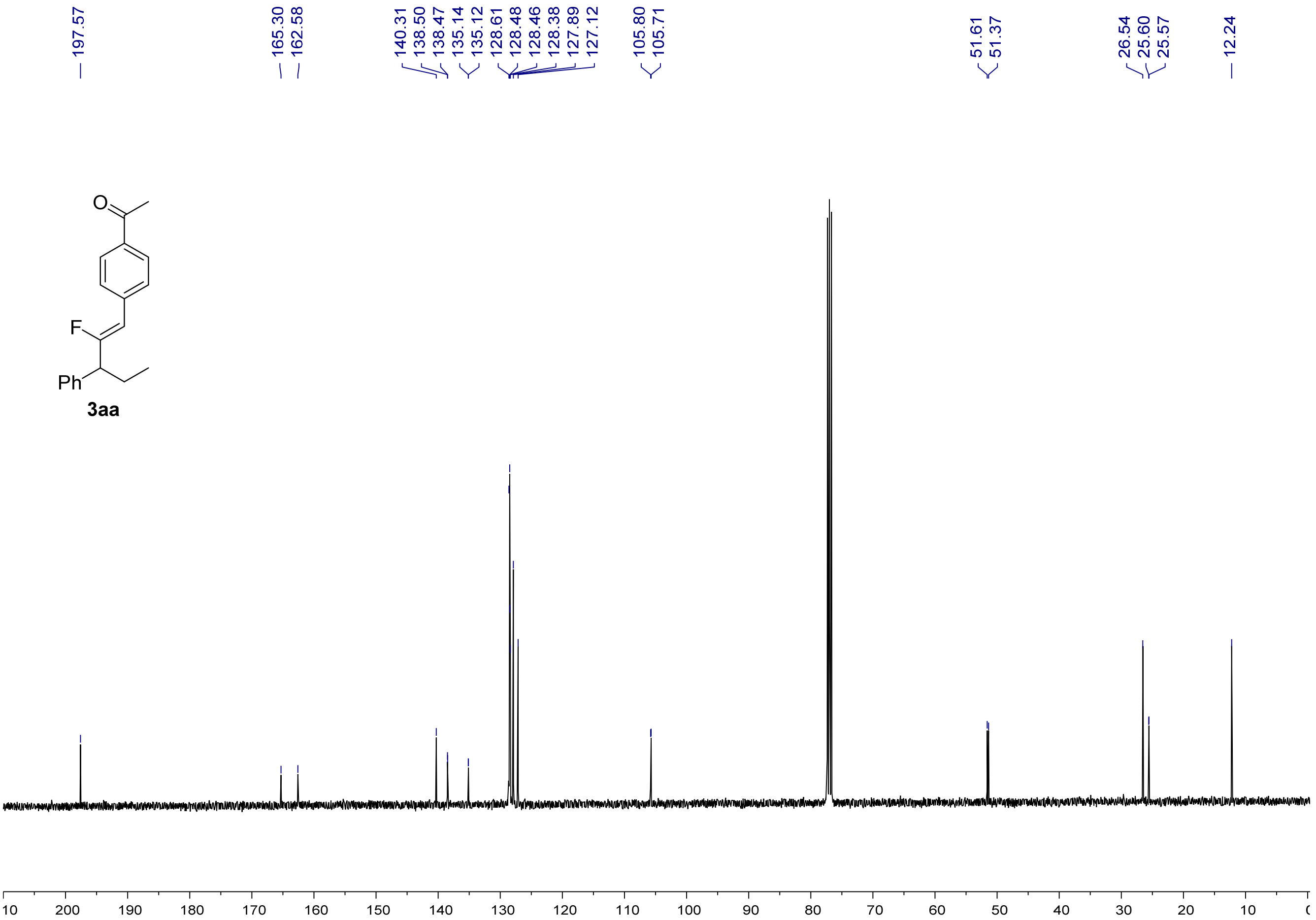
197.57
165.30
162.58
140.31
138.50
138.47
135.14
135.12
128.61
128.48
128.46
128.38
127.89
127.12
105.80
105.71
51.61
51.37
26.54
25.60
25.57
12.24
O
F
Ph
3aa
10
200
190
180
170
160
150
140
130
120
110
100
90
80
70
60
50
40
30
20
10

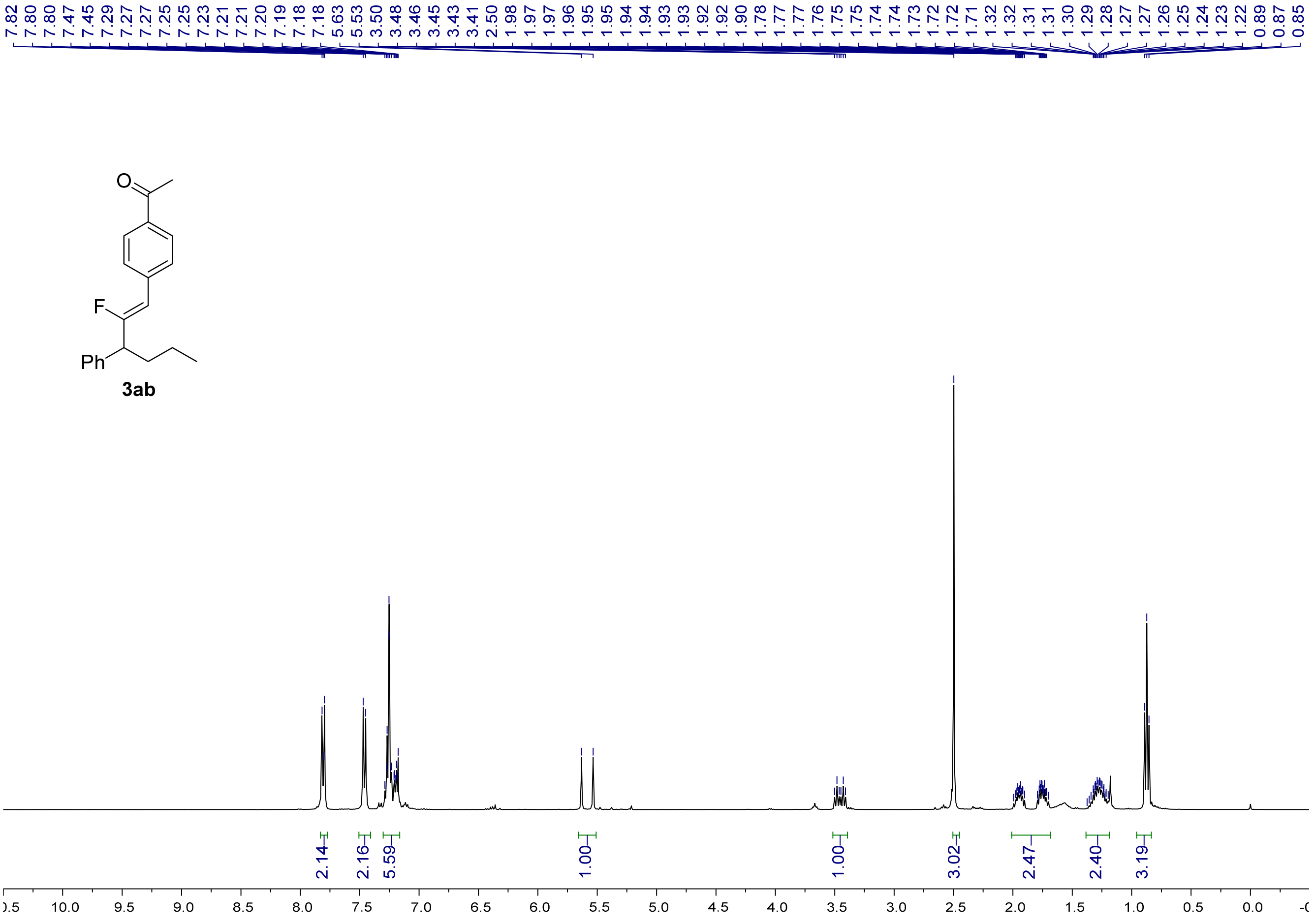
7.82
7.80
7.80
7.47
7.45
7.29
7.27
7.27
7.25
7.25
7.23
7.21
7.21
7.20
7.19
7.18
7.18
5.63
5.53
3.50
3.48
3.46
3.45
3.43
3.41
2.50
1.98
1.97
1.97
1.96
1.95
1.95
1.94
1.94
1.93
1.93
1.92
1.92
1.90
1.78
1.77
1.77
1.76
1.75
1.75
1.74
1.74
1.73
1.72
1.72
1.71
1.32
1.32
1.31
1.31
1.30
1.29
1.28
1.27
1.27
1.26
1.25
1.24
1.23
1.22
0.89
0.87
0.85
O
F
Ph
3ab
2.14
2.16
5.59
1.00
1.00
3.02
2.47
2.40
3.19
10.0
9.5
9.0
8.5
8.0
7.5
7.0
6.5
6.0
5.5
5.0
4.5
4.0
3.5
3.0
2.5
2.0
1.5
1.0
0.5
0.0

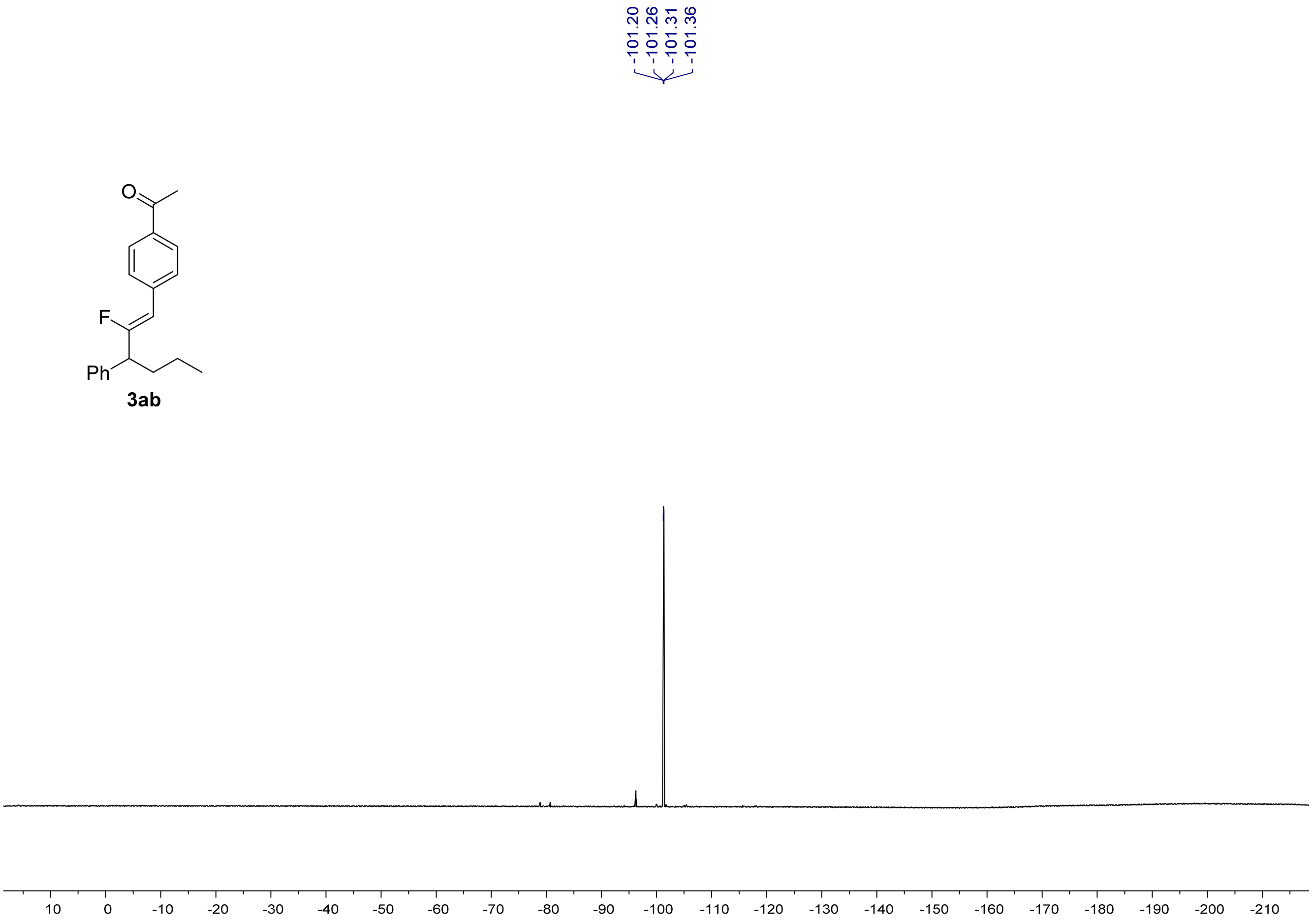
-101.20
-101.26
-101.31
-101.36
O
F
Ph
3ab
10 0 -10 -20 -30 -40 -50 -60 -70 -80 -90 -100 -110 -120 -130 -140 -150 -160 -170 -180 -190 -200 -210

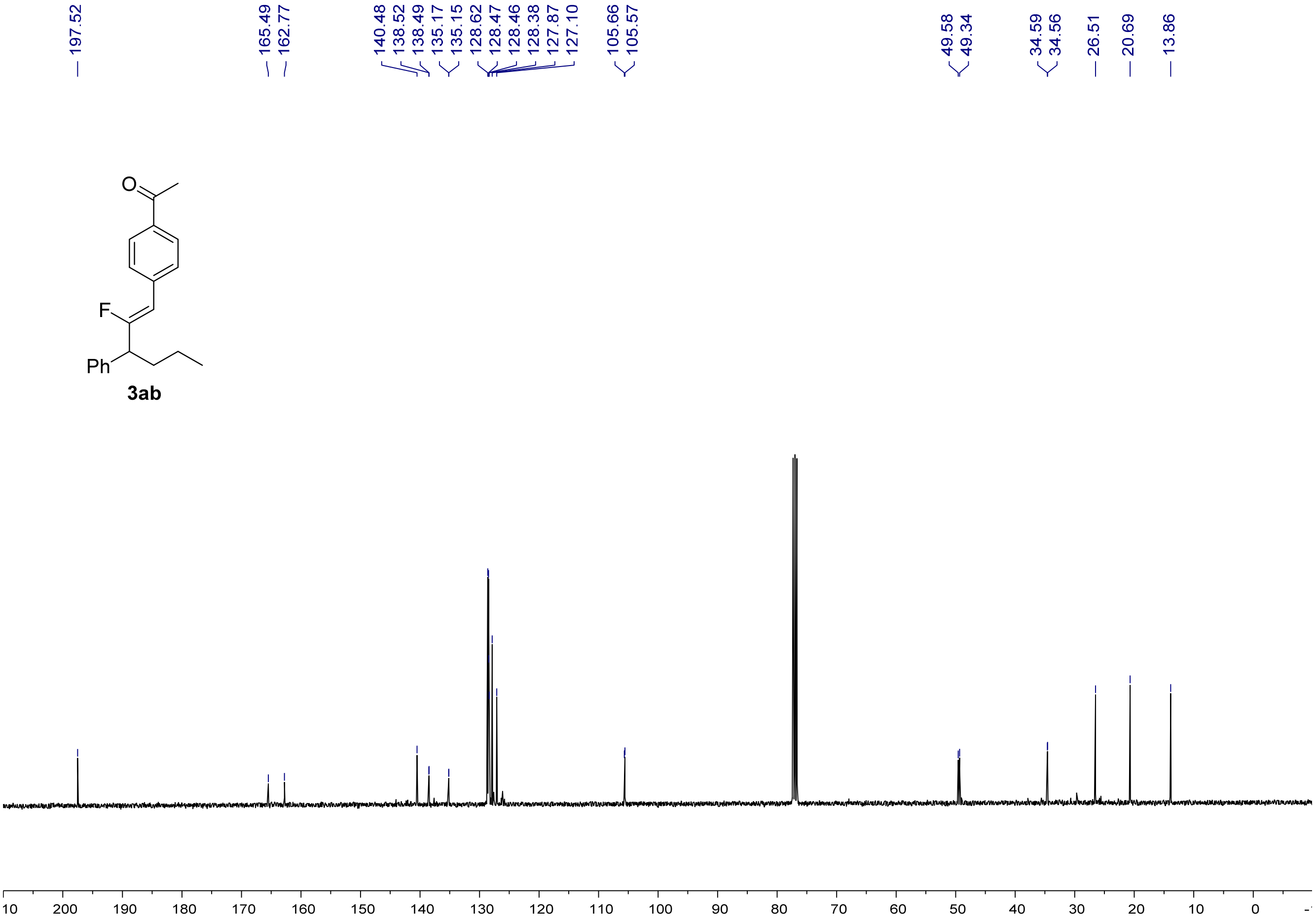
197.52
165.49
162.77
140.48
138.52
138.49
135.17
135.15
128.62
128.47
128.46
128.38
127.87
127.10
105.66
105.57
49.58
49.34
34.59
34.56
26.51
20.69
13.86
O
F
Ph
3ab
200 190 180 170 160 150 140 130 120 110 100 90 80 70 60 50 40 30 20 10 0

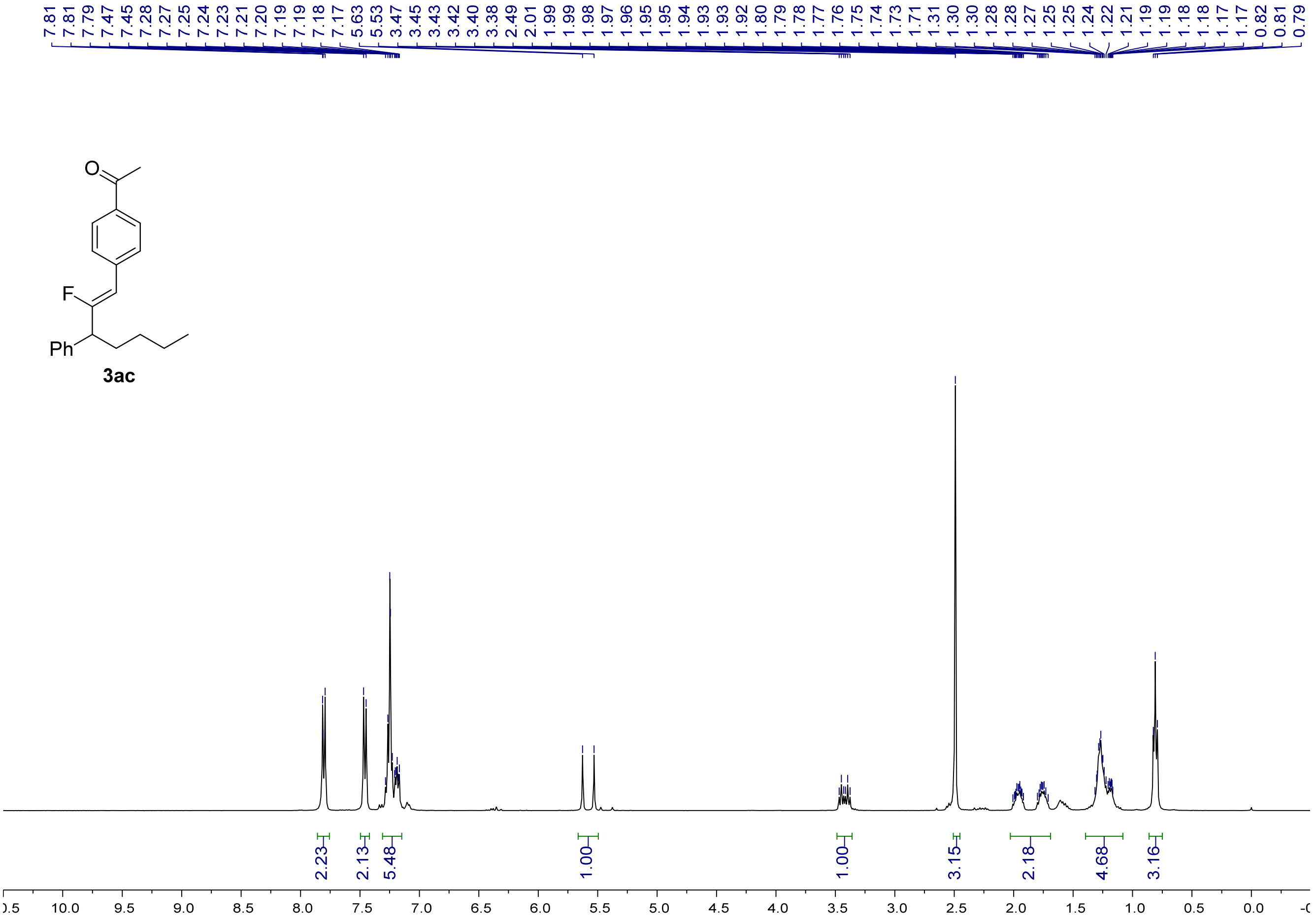
7.81
7.81
7.79
7.47
7.45
7.28
7.27
7.25
7.24
7.23
7.21
7.20
7.19
7.19
7.18
7.17
5.63
5.53
3.47
3.45
3.43
3.42
3.40
3.38
2.49
2.01
1.99
1.99
1.98
1.97
1.96
1.95
1.95
1.94
1.93
1.93
1.92
1.80
1.79
1.78
1.77
1.76
1.75
1.74
1.73
1.71
1.31
1.30
1.30
1.28
1.28
1.27
1.25
1.25
1.24
1.22
1.21
1.19
1.18
1.18
1.17
1.17
0.82
0.81
0.79
O
F
Ph
3ac
2.23
2.13
5.48
1.00
1.00
3.15
2.18
4.68
3.16
10.0
9.5
9.0
8.5
8.0
7.5
7.0
6.5
6.0
5.5
5.0
4.5
4.0
3.5
3.0
2.5
2.0
1.5
1.0
0.5
0.0

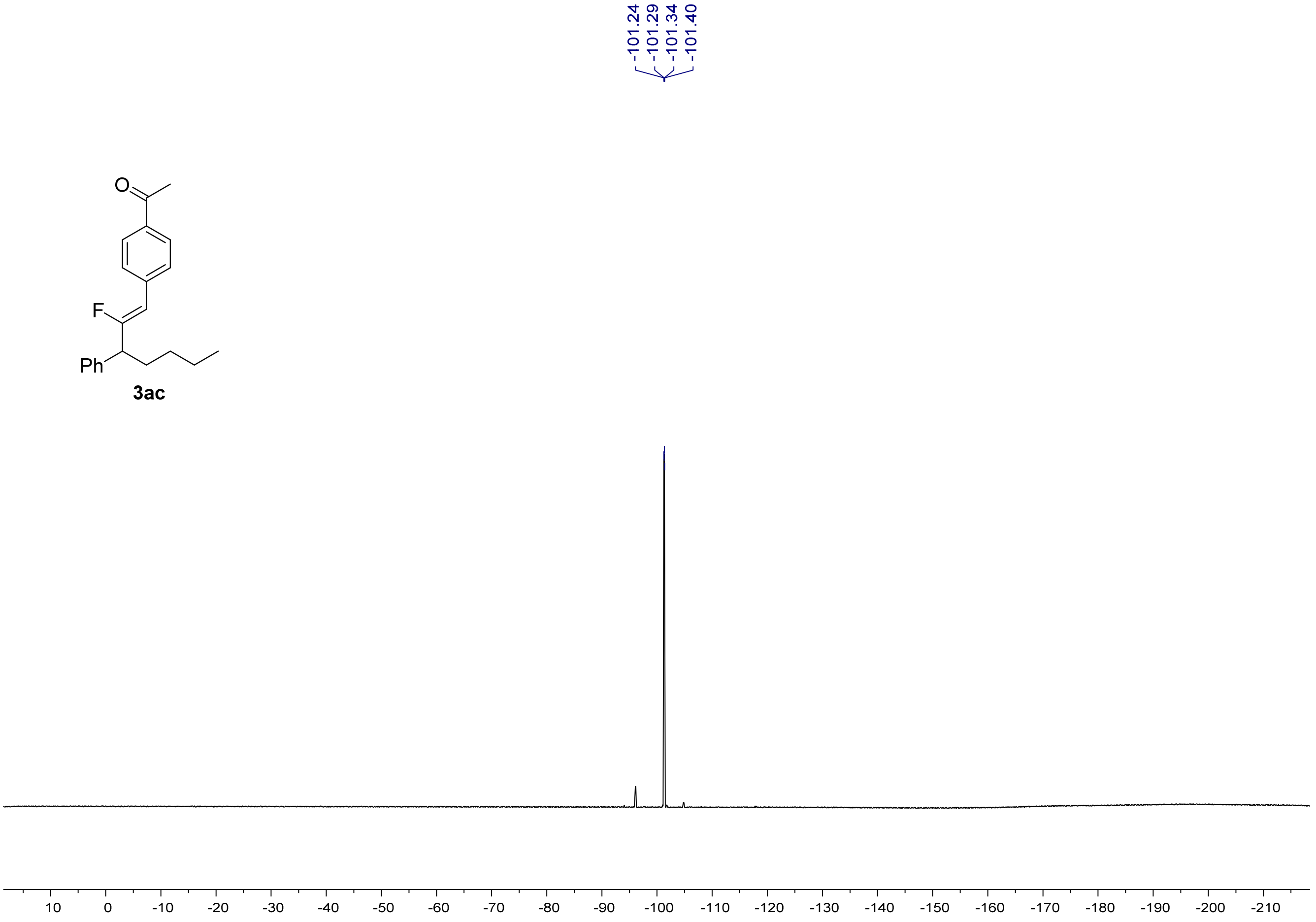
-101.24
-101.29
-101.34
-101.40
O
F
Ph
3ac
10
0
-10
-20
-30
-40
-50
-60
-70
-80
-90
-100
-110
-120
-130
-140
-150
-160
-170
-180
-190
-200
-210

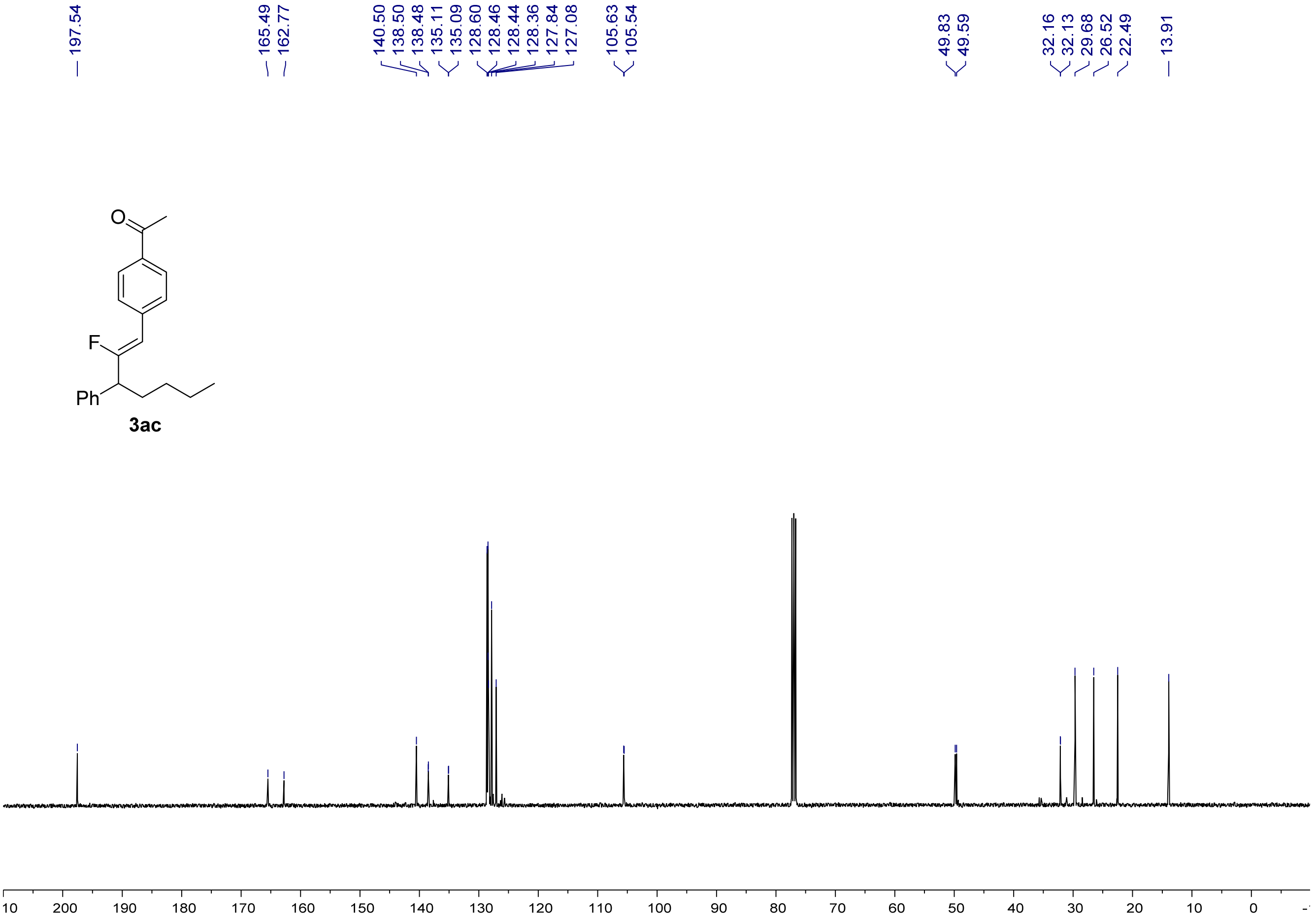
197.54
165.49
162.77
140.50
138.50
138.48
135.11
135.09
128.60
128.46
128.44
128.36
127.84
127.08
105.63
105.54
49.83
49.59
32.16
32.13
29.68
26.52
22.49
13.91
O
F
Ph
3ac

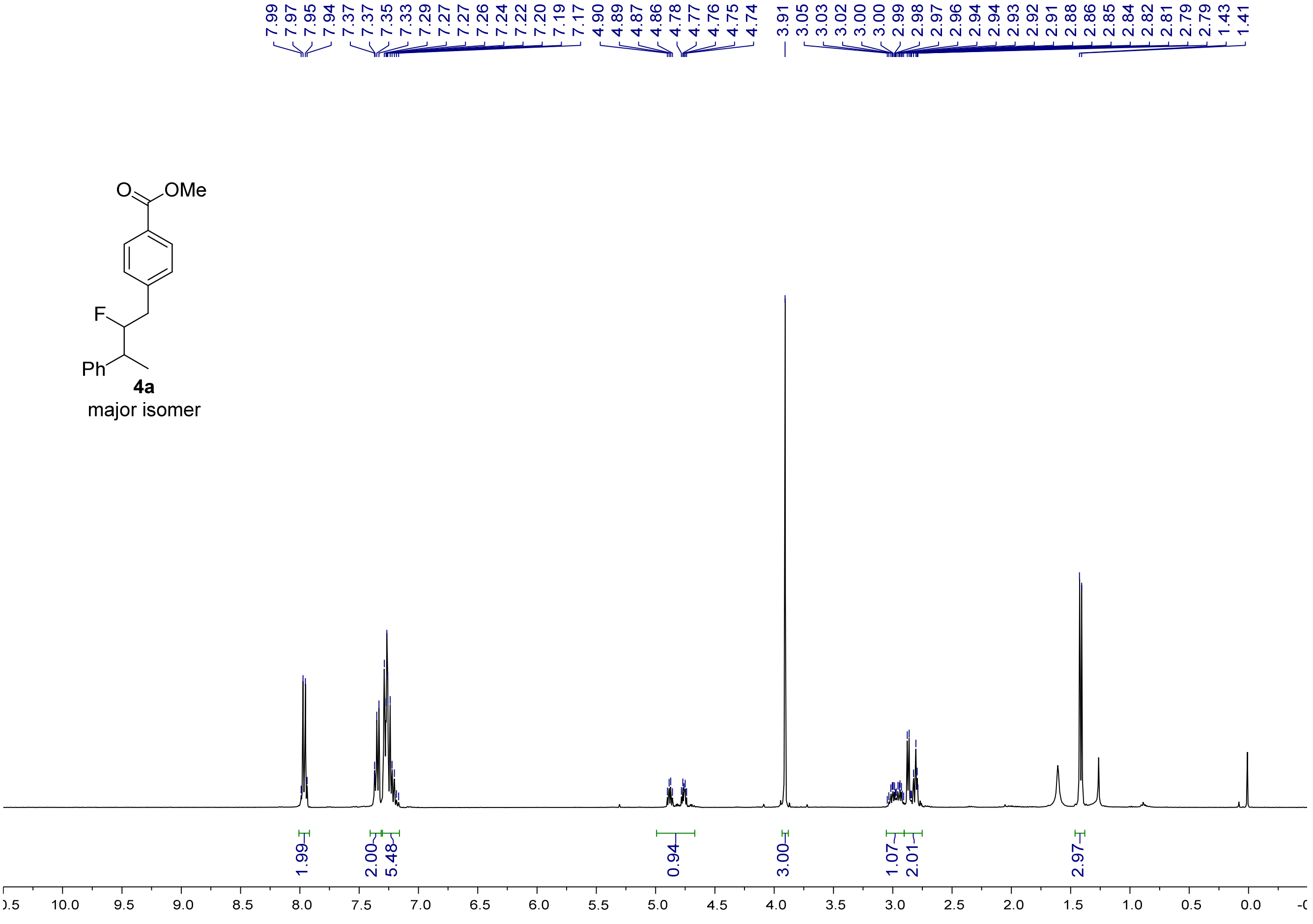
7.99
7.97
7.95
7.94
7.37
7.37
7.35
7.33
7.29
7.27
7.26
7.24
7.22
7.20
7.19
7.17
4.90
4.89
4.87
4.86
4.78
4.77
4.76
4.75
4.74
3.91
3.05
3.03
3.02
3.00
3.00
2.99
2.98
2.97
2.96
2.94
2.94
2.93
2.92
2.91
2.88
2.86
2.85
2.84
2.82
2.81
2.79
2.79
1.43
1.41
O
OMe
F
Ph
4a
major isomer
1.99
2.00
5.48
0.94
3.00
1.07
2.01
2.97

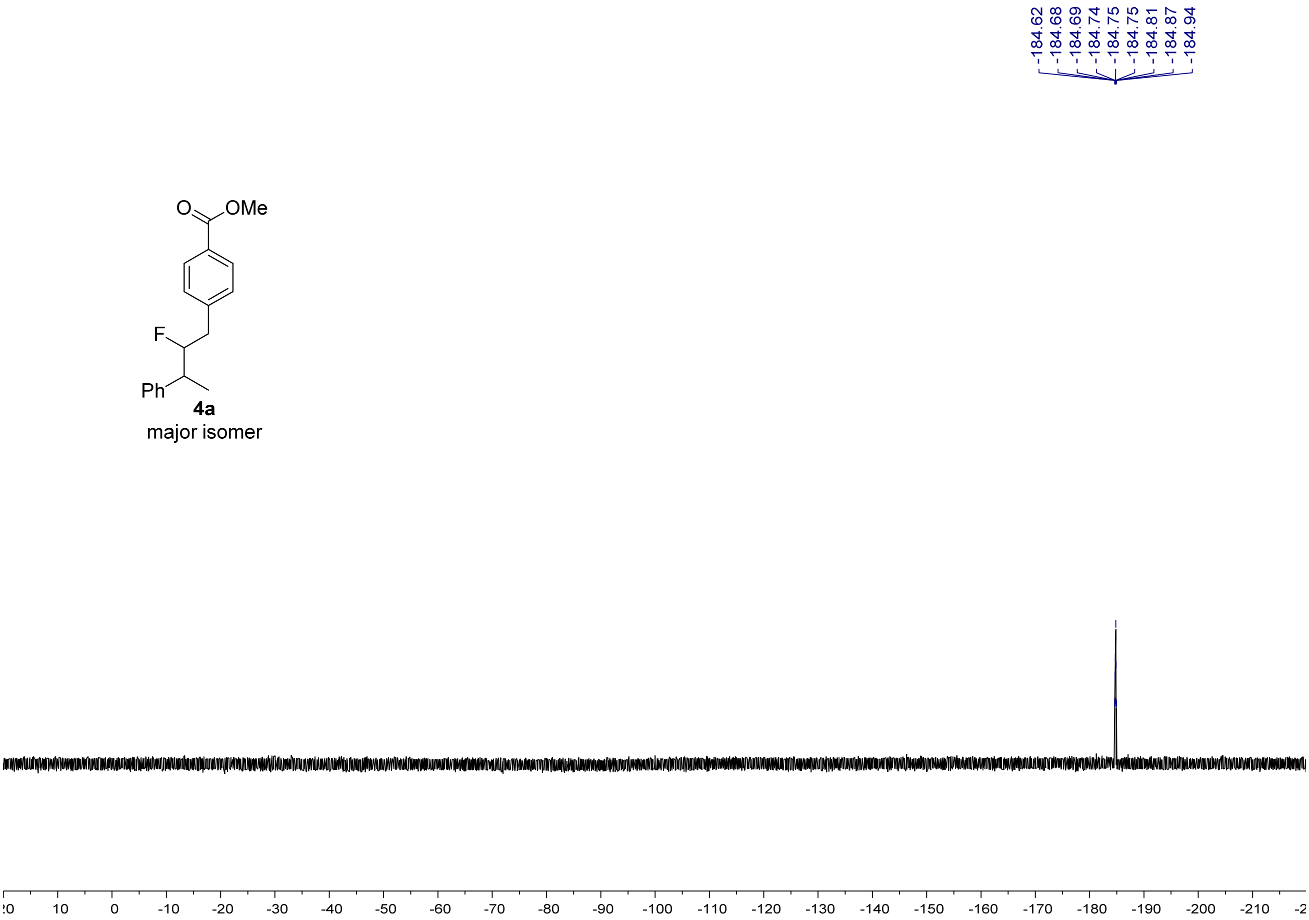
-184.62
-184.68
-184.69
-184.74
-184.75
-184.75
-184.81
-184.87
-184.94
O
OMe
F
Ph
4a
major isomer
10
0
-10
-20
-30
-40
-50
-60
-70
-80
-90
-100
-110
-120
-130
-140
-150
-160
-170
-180
-190
-200
-210

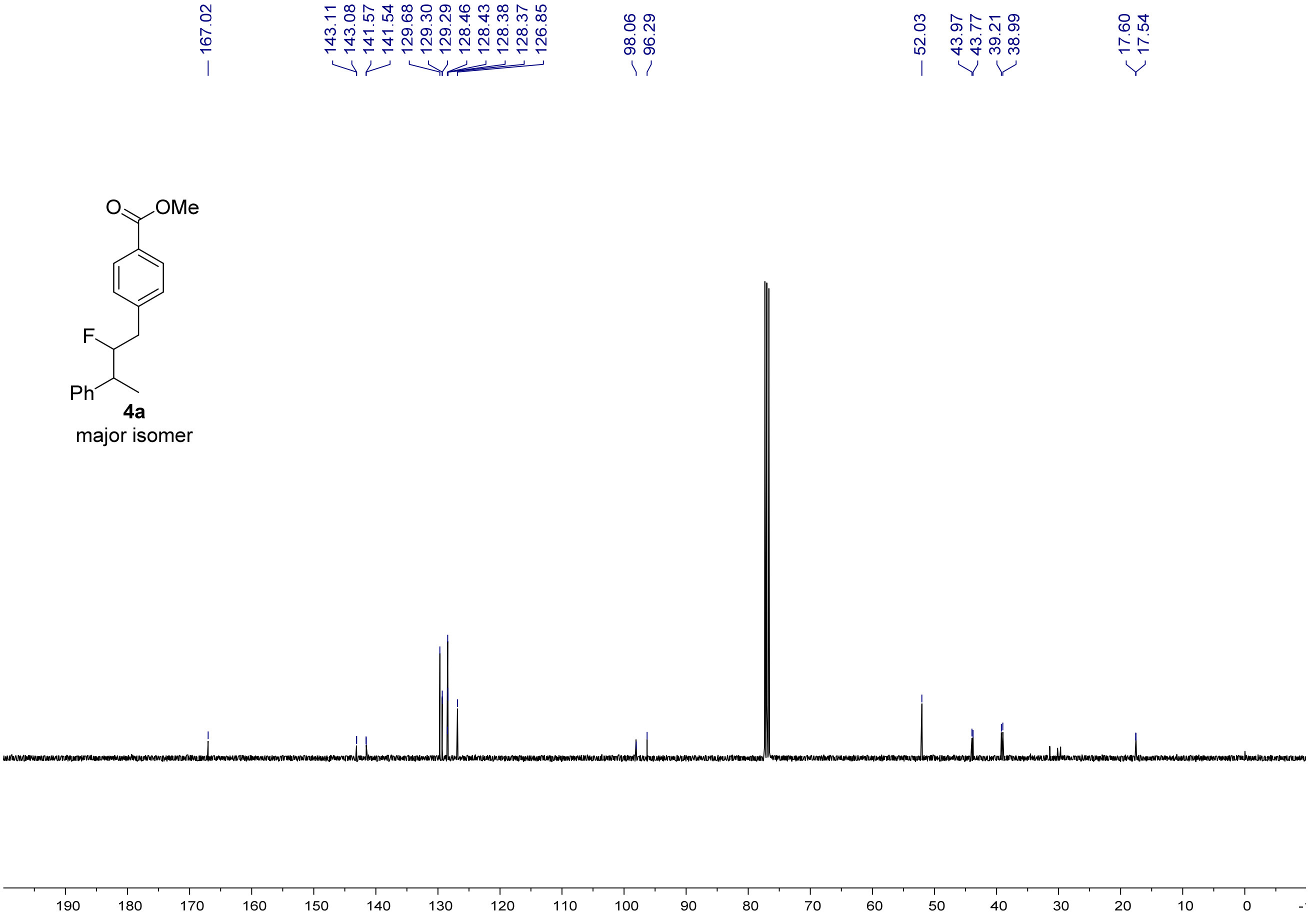
167.02
143.11
143.08
141.57
141.54
129.68
129.30
129.29
128.46
128.43
128.38
128.37
126.85
98.06
96.29
52.03
43.97
43.77
39.21
38.99
17.60
17.54
O
OMe
F
Ph
4a
major isomer
190
180
170
160
150
140
130
120
110
100
90
80
70
60
50
40
30
20
10
0

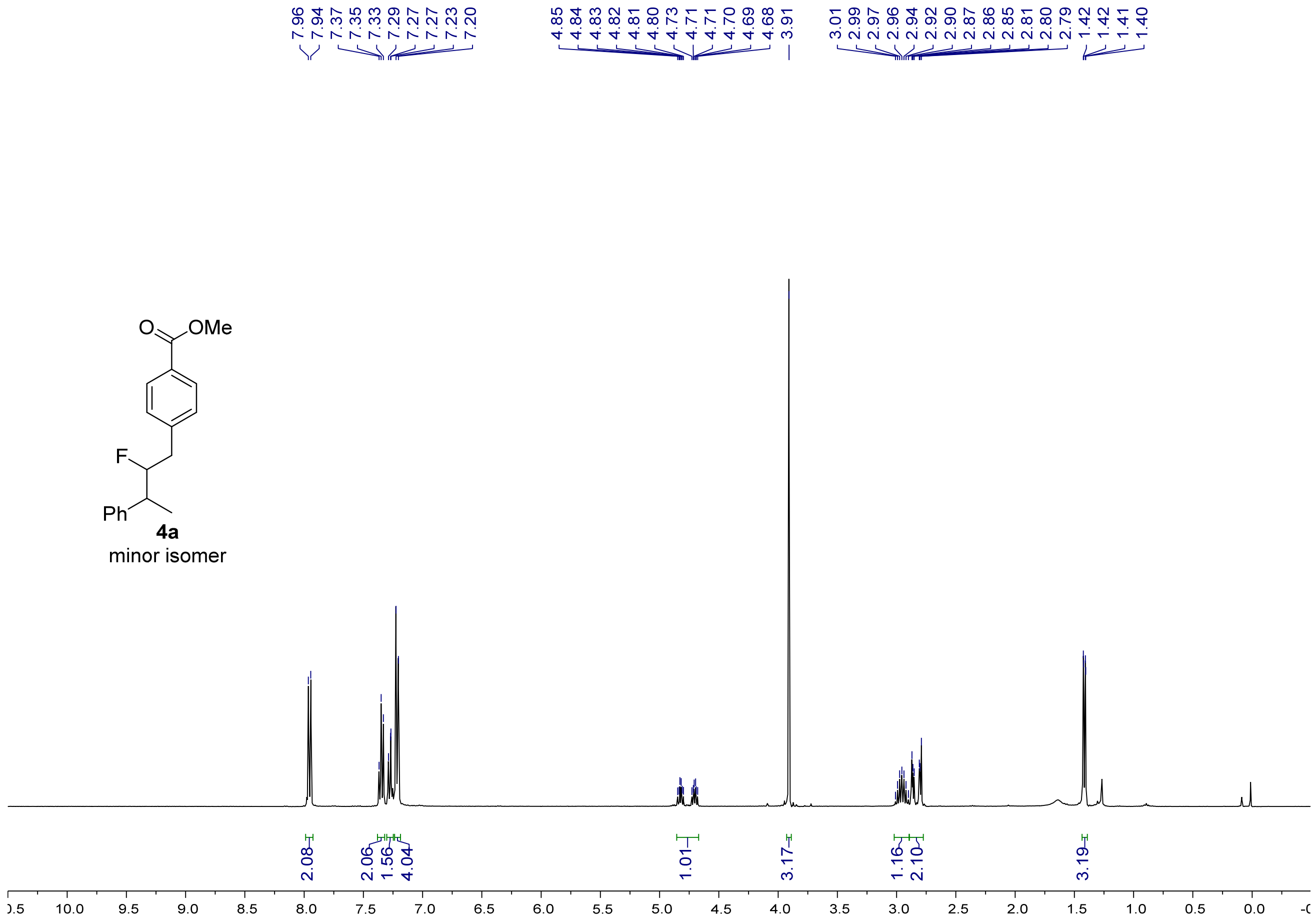
7.96
7.94
7.37
7.35
7.33
7.29
7.27
7.27
7.23
7.20
4.85
4.84
4.83
4.82
4.81
4.80
4.73
4.71
4.71
4.70
4.69
4.68
3.91
3.01
2.99
2.97
2.96
2.94
2.92
2.90
2.87
2.86
2.85
2.81
2.80
2.79
1.42
1.42
1.41
1.40
O
OMe
F
Ph
4a
minor isomer
2.08
2.06
1.56
4.04
1.01
3.17
1.16
2.10
3.19

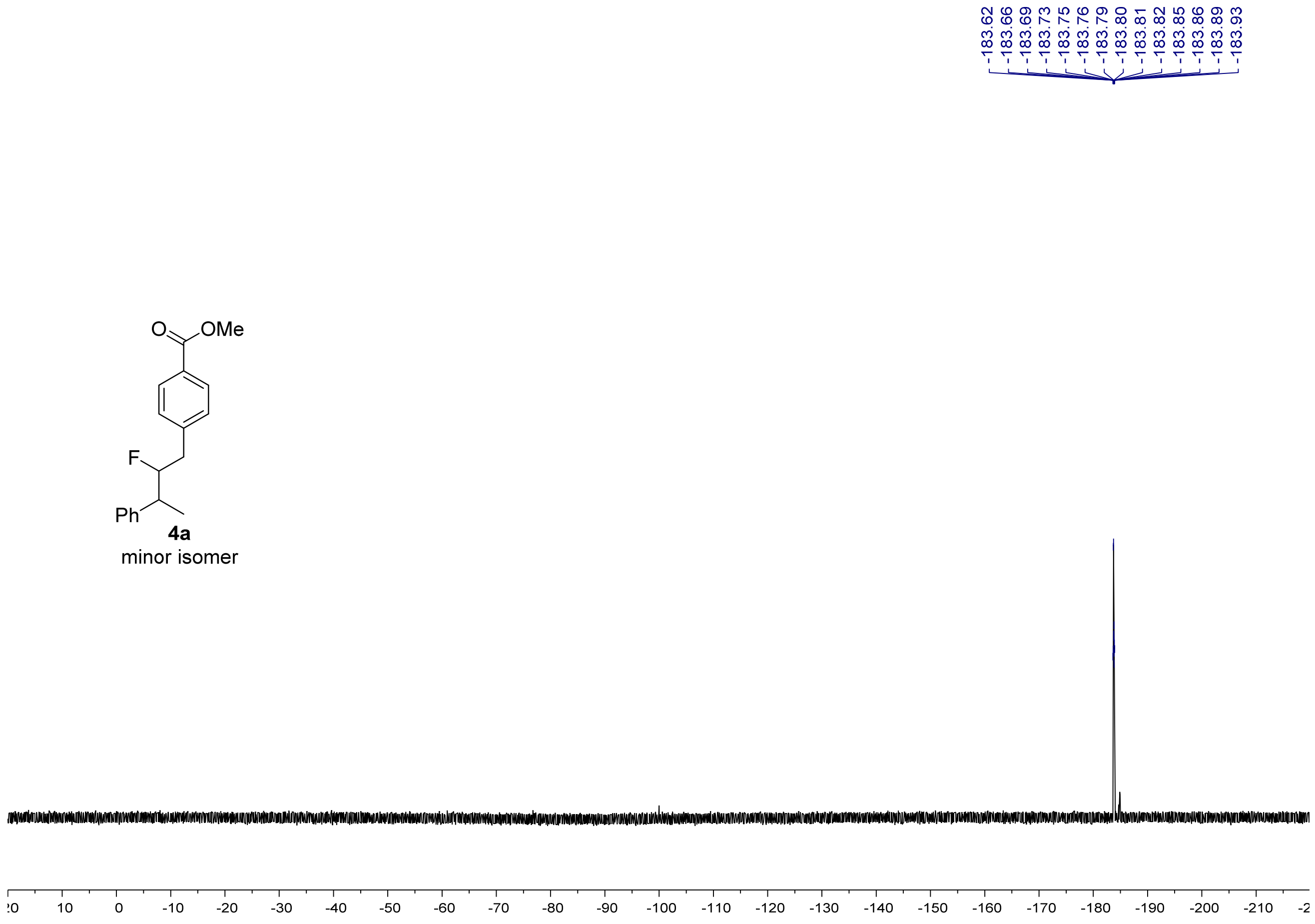
-183.62
-183.66
-183.69
-183.73
-183.75
-183.76
-183.79
-183.80
-183.81
-183.82
-183.85
-183.86
-183.89
-183.93
O
OMe
F
Ph
4a
minor isomer

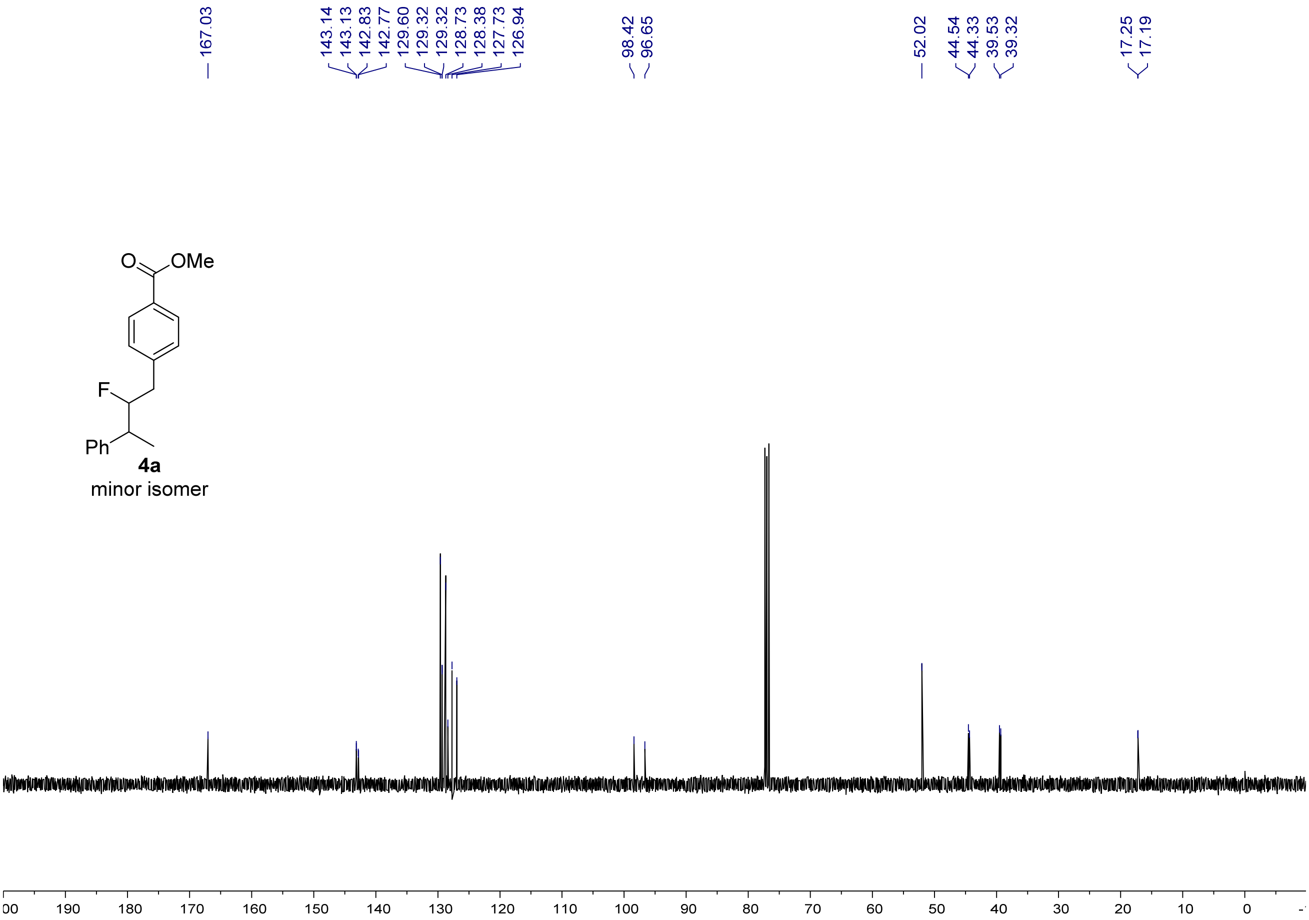
167.03
143.14
143.13
142.83
142.77
129.60
129.32
129.32
128.73
128.38
127.73
126.94
98.42
96.65
52.02
44.54
44.33
39.53
39.32
17.25
17.19
O
OMe
F
Ph
4a
minor isomer

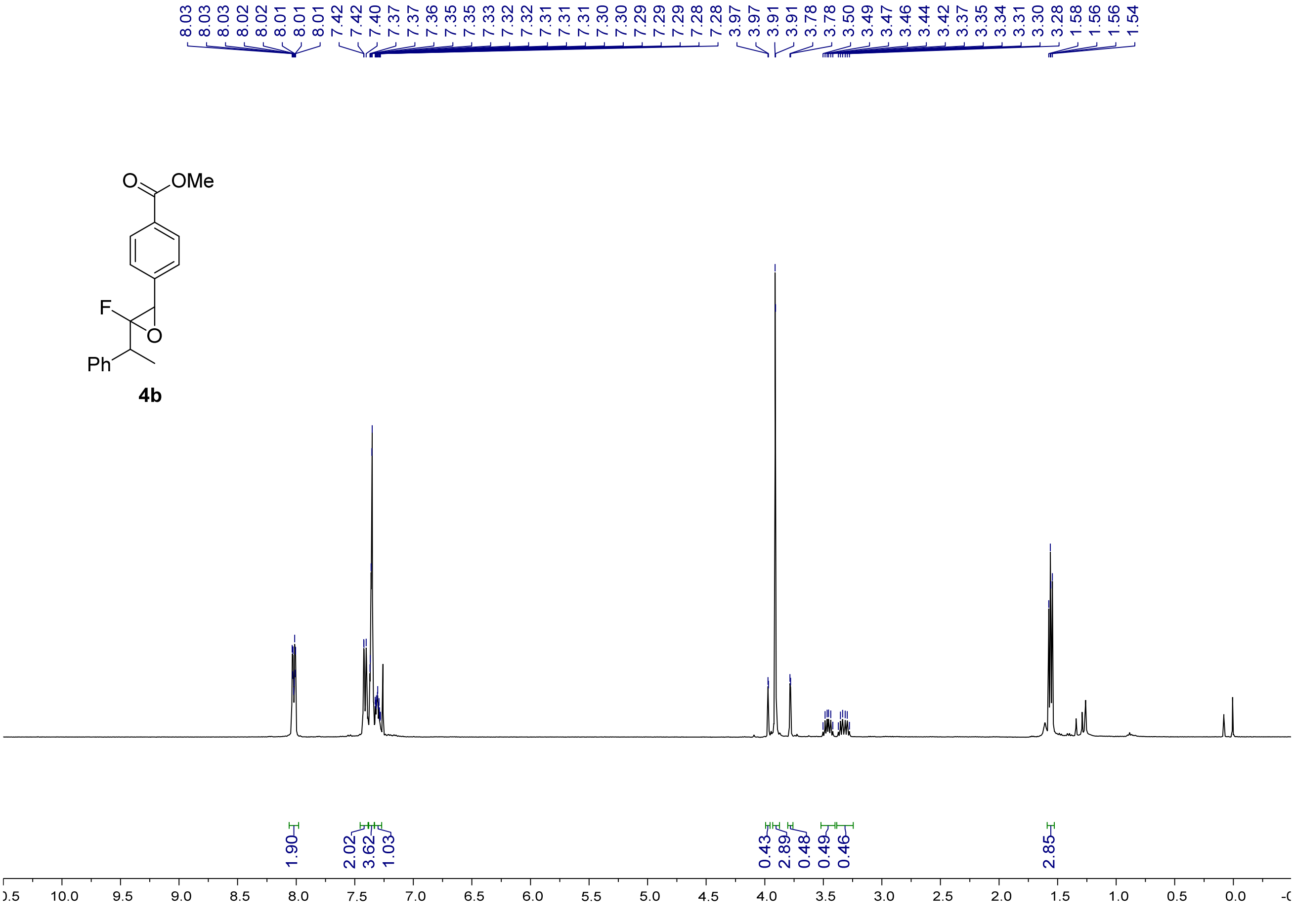
O
OMe
F
O
Ph
4b
1.90
2.02
3.62
1.03
0.43
2.89
0.48
0.49
0.46
2.85

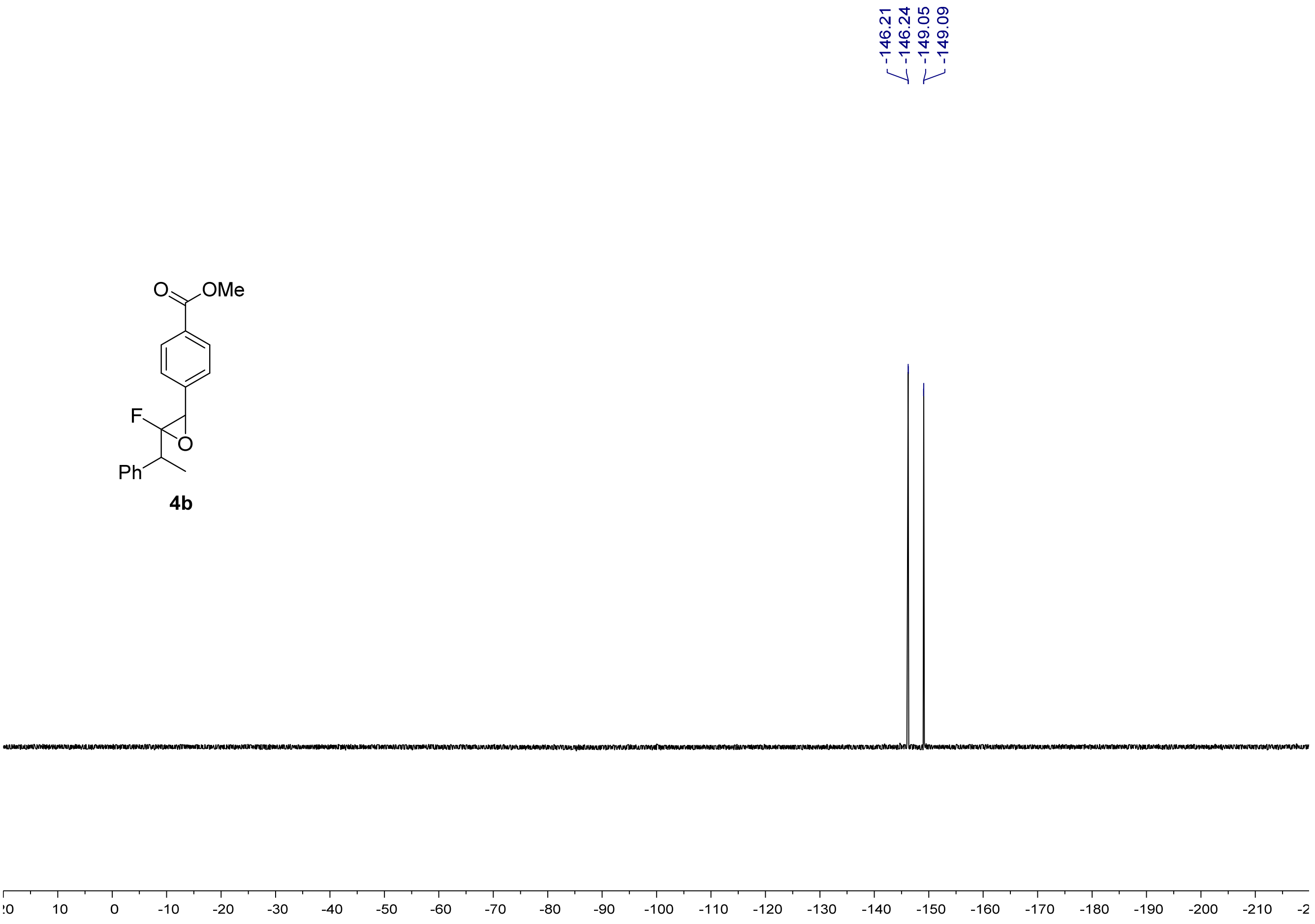
-146.21
-146.24
-149.05
-149.09
OMe
O
F
O
Ph
4b

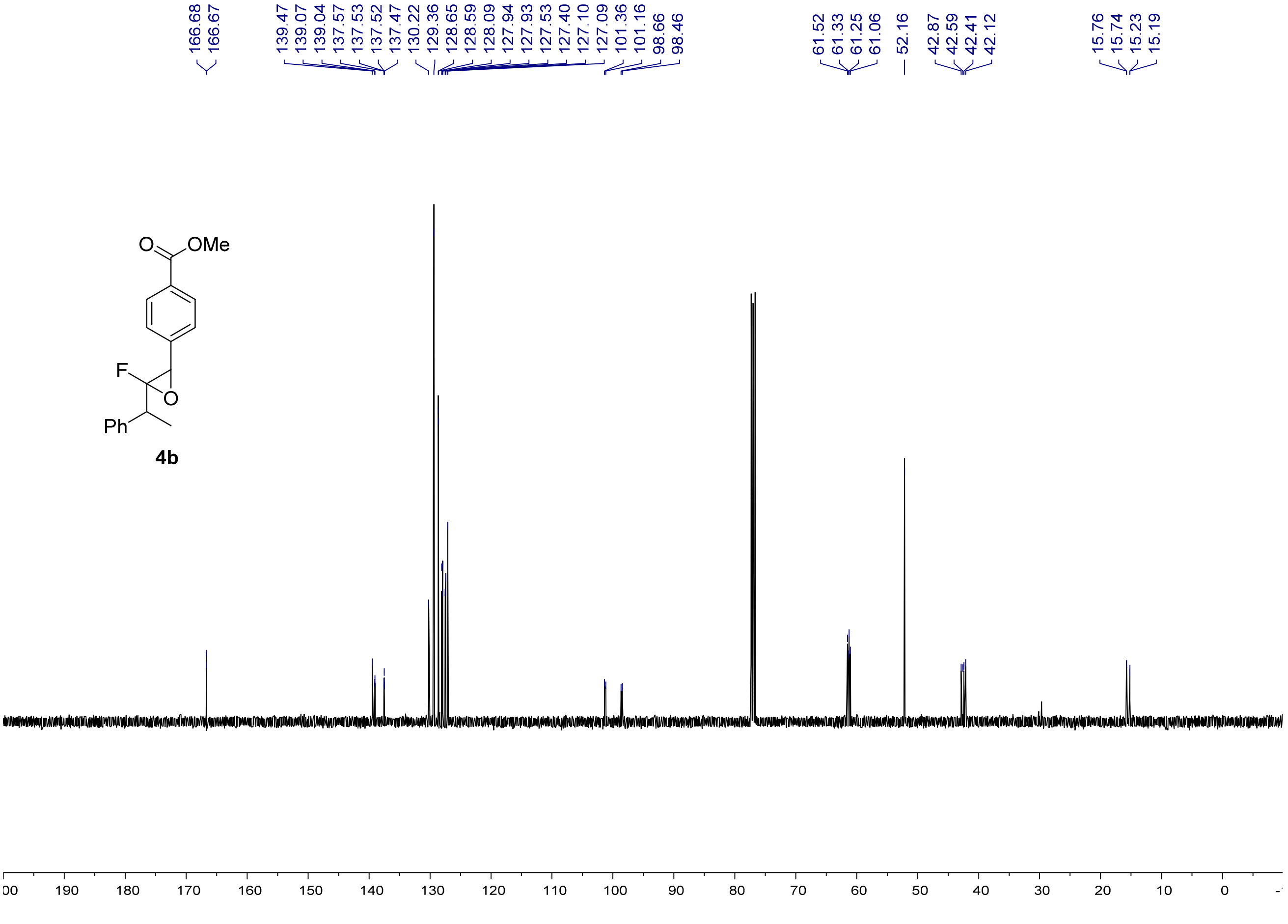
166.68
166.67
139.47
139.07
139.04
137.57
137.53
137.52
137.47
130.22
129.36
128.65
128.59
128.09
127.94
127.93
127.53
127.40
127.10
127.09
101.36
101.16
98.66
98.46
61.52
61.33
61.25
61.06
52.16
42.87
42.59
42.41
42.12
15.76
15.74
15.23
15.19
OMe
O
F
O
Ph
4b

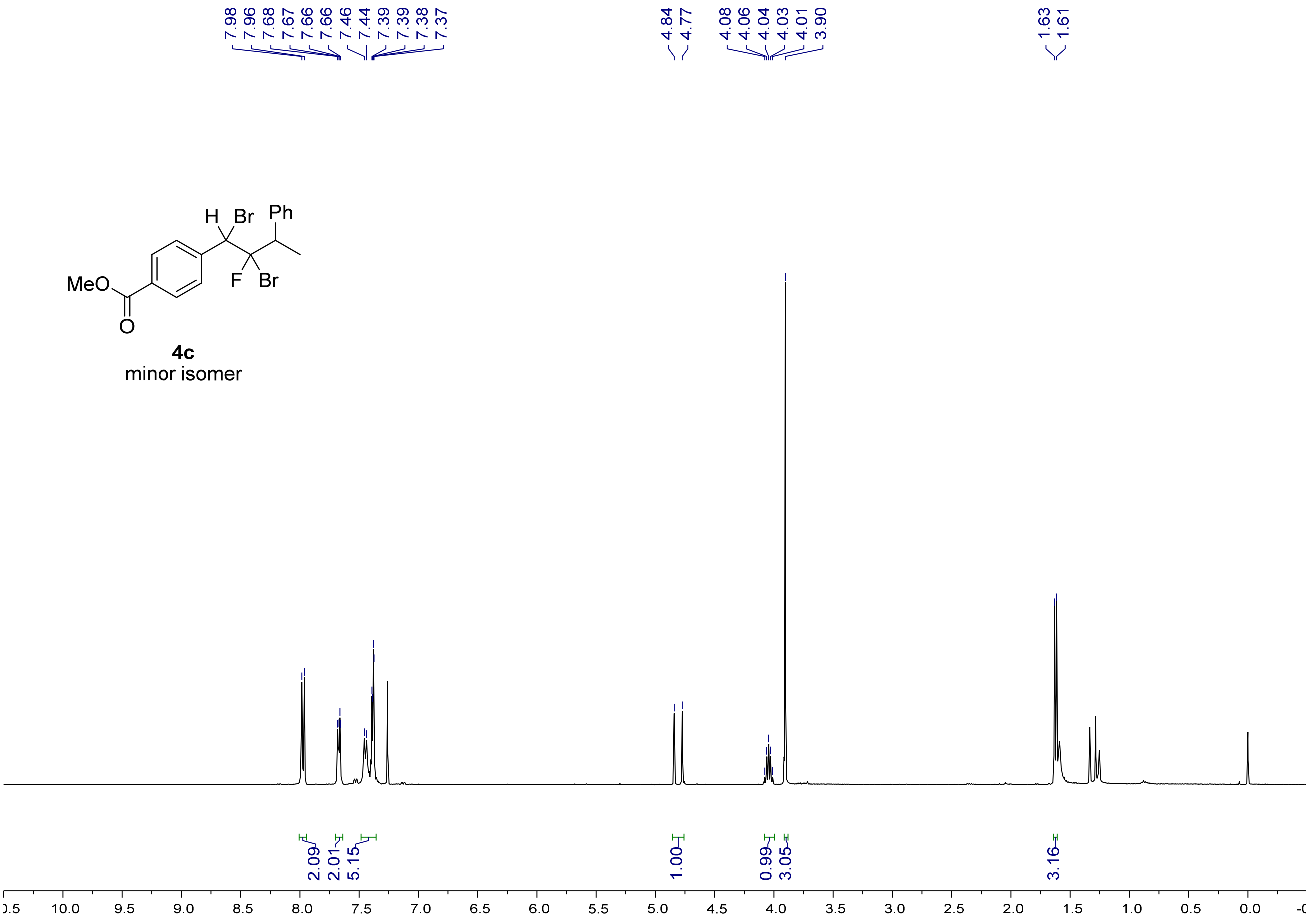

7.98
7.96
7.68
7.67
7.66
7.66
7.46
7.44
7.39
7.39
7.38
7.37
4.84
4.77
4.08
4.06
4.04
4.03
4.01
3.90
1.63
1.61
H Br Ph
MeO
F Br
O
4c
minor isomer
2.09
2.01
5.15
1.00
0.99
3.05
3.16
10.0
9.5
9.0
8.5
8.0
7.5
7.0
6.5
6.0
5.5
5.0
4.5
4.0
3.5
3.0
2.5
2.0
1.5
1.0
0.5
0.0

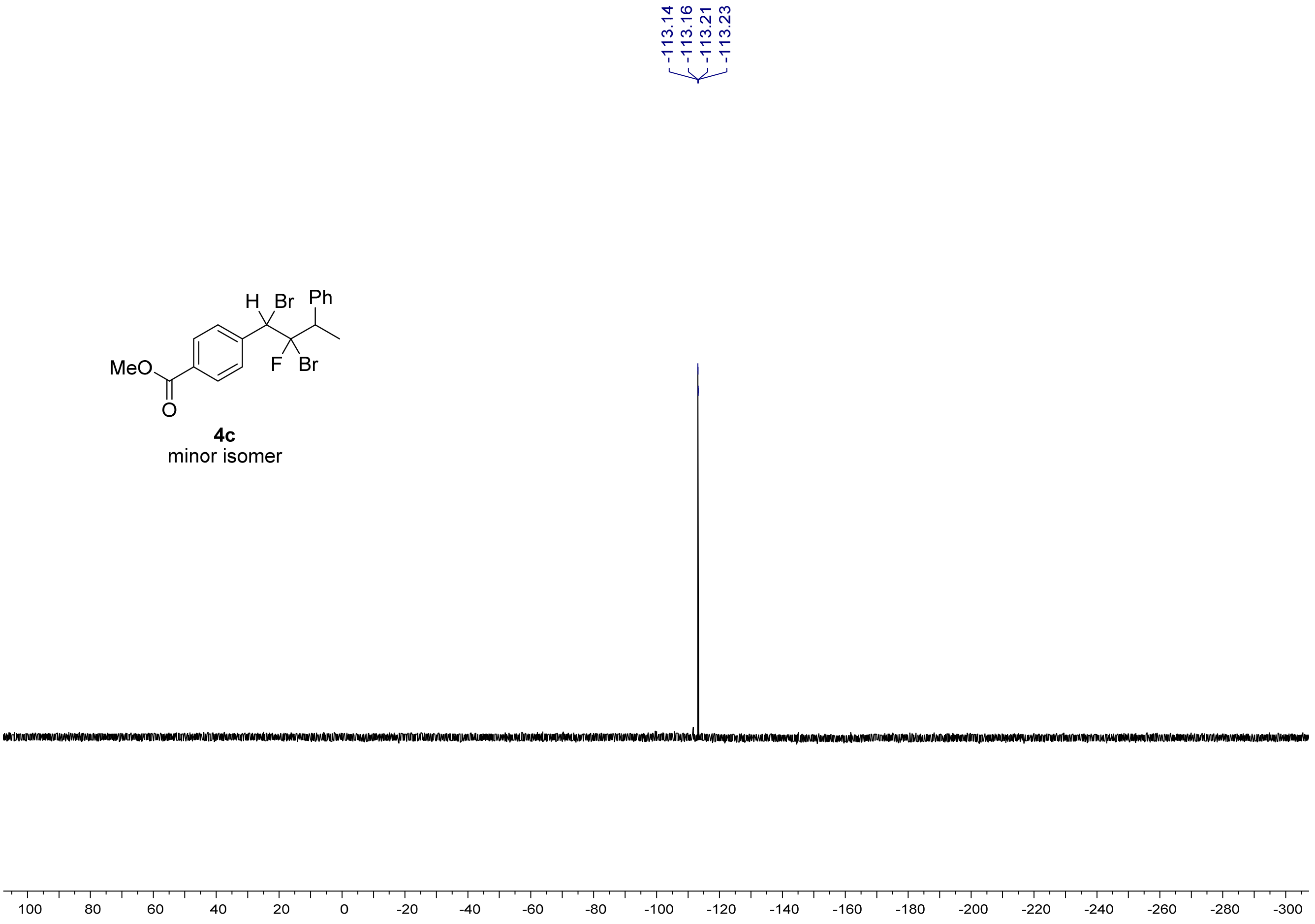

-113.14
-113.16
-113.21
-113.23
H Br Ph
MeO
F Br
O
4c
minor isomer
100
80
60
40
20
0
-20
-40
-60
-80
-100
-120
-140
-160
-180
-200
-220
-240
-260
-280
-300

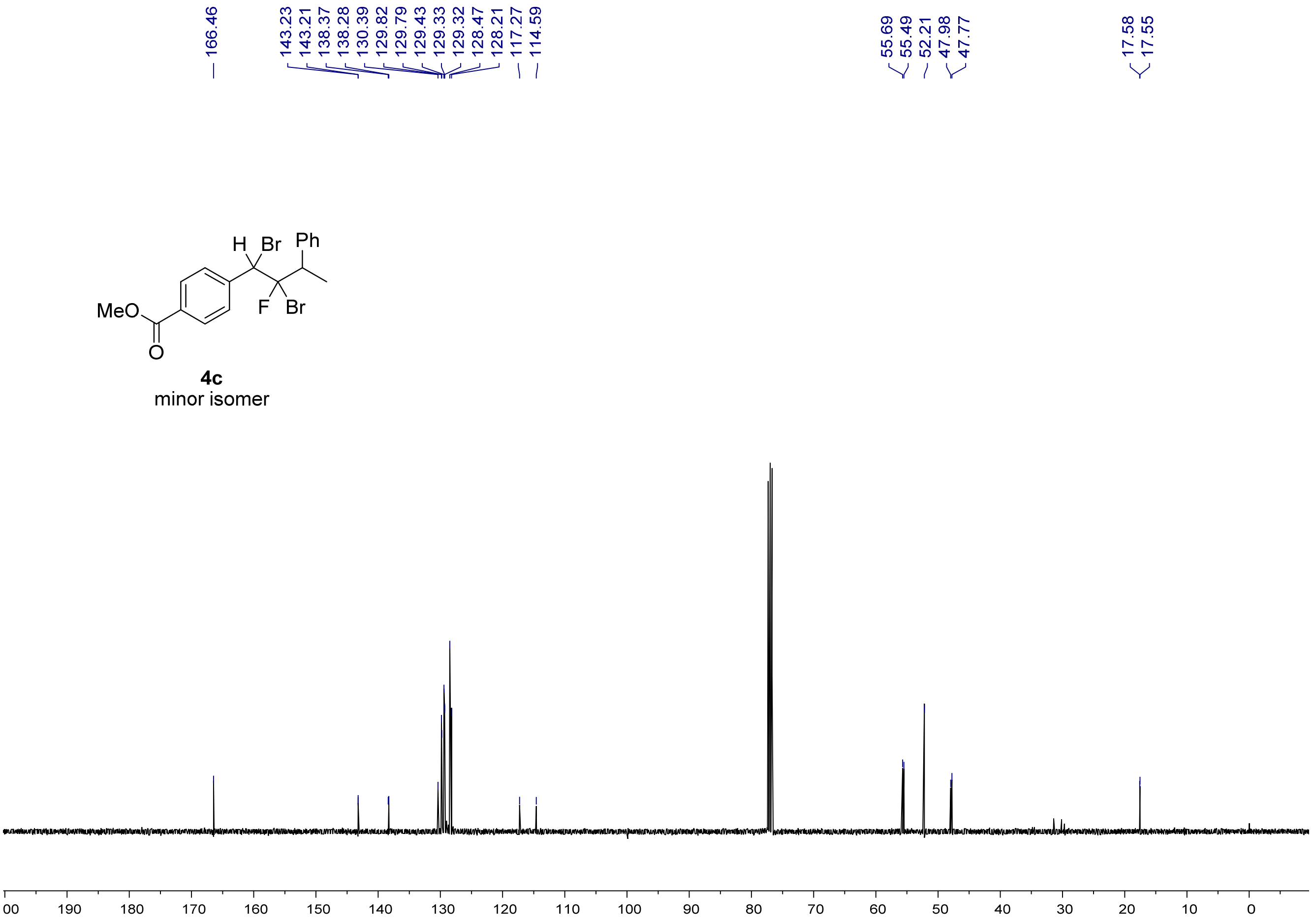
166.46
143.23
143.21
138.37
138.28
130.39
129.82
129.79
129.43
129.33
129.32
128.47
128.21
117.27
114.59
55.69
55.49
52.21
47.98
47.77
17.58
17.55
H Br Ph
MeO
F Br
O
4c
minor isomer
00 190 180 170 160 150 140 130 120 110 100 90 80 70 60 50 40 30 20 10 0

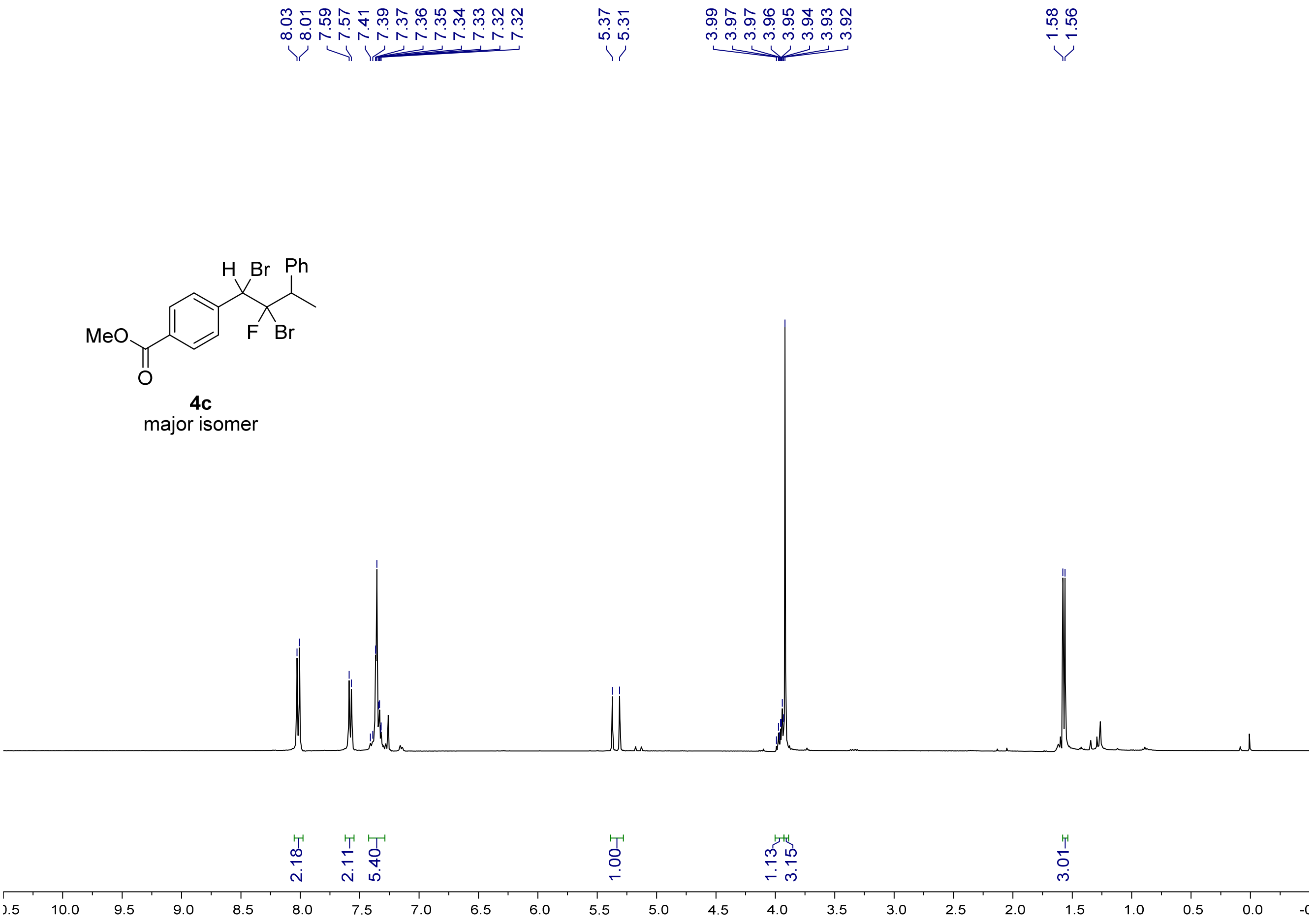
8.03
8.01
7.59
7.57
7.41
7.39
7.37
7.36
7.35
7.34
7.33
7.32
7.32
5.37
5.31
3.99
3.97
3.97
3.96
3.95
3.94
3.93
3.92
1.58
1.56
H Br Ph
MeO
F Br
O
4c
major isomer
2.18
2.11
5.40
1.00
1.13
3.15
3.01
10.0 9.5 9.0 8.5 8.0 7.5 7.0 6.5 6.0 5.5 5.0 4.5 4.0 3.5 3.0 2.5 2.0 1.5 1.0 0.5 0.0

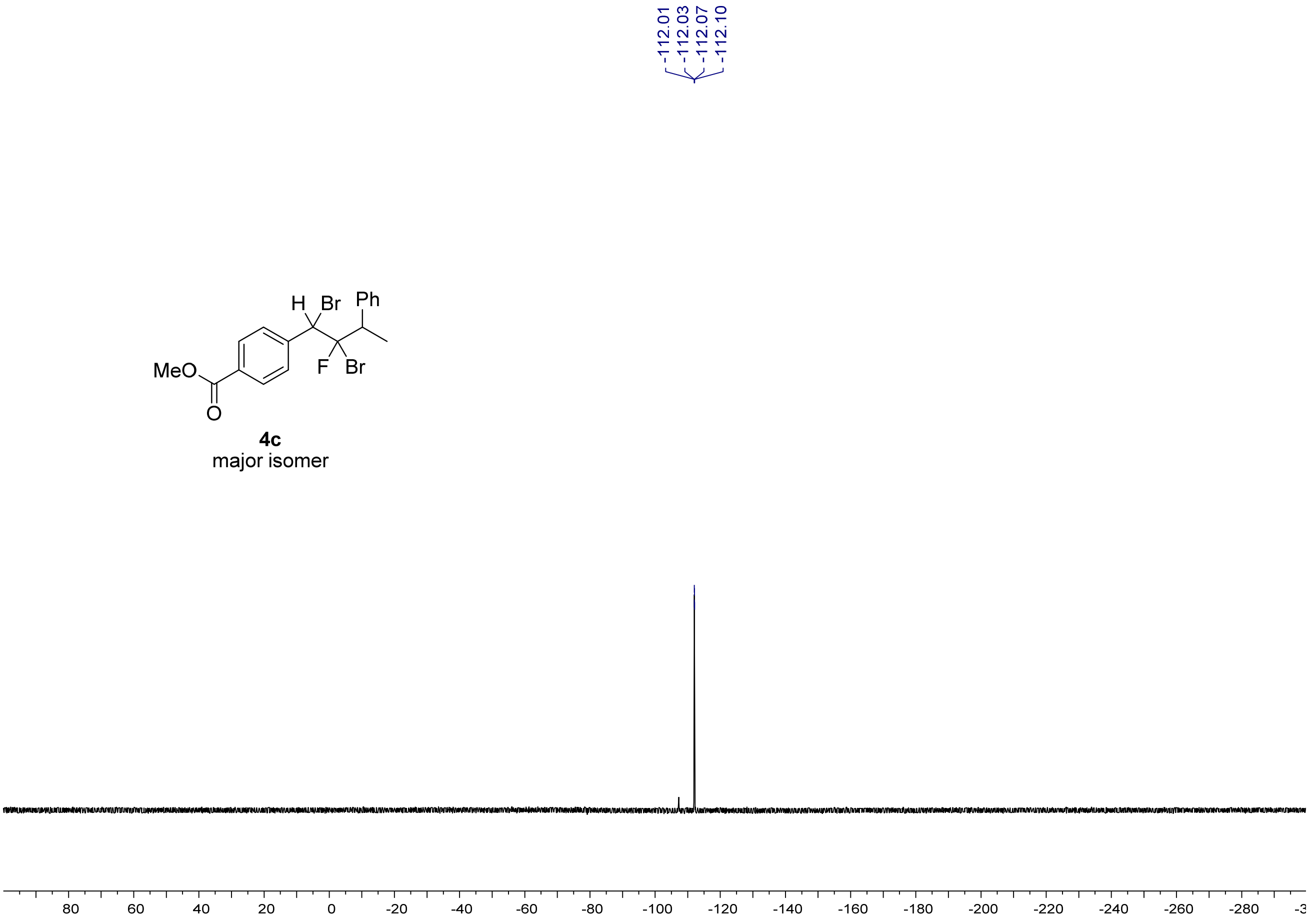
-112.01
-112.03
-112.07
-112.10
H Br Ph
MeO
O
F Br
4c
major isomer
80 60 40 20 0 -20 -40 -60 -80 -100 -120 -140 -160 -180 -200 -220 -240 -260 -280

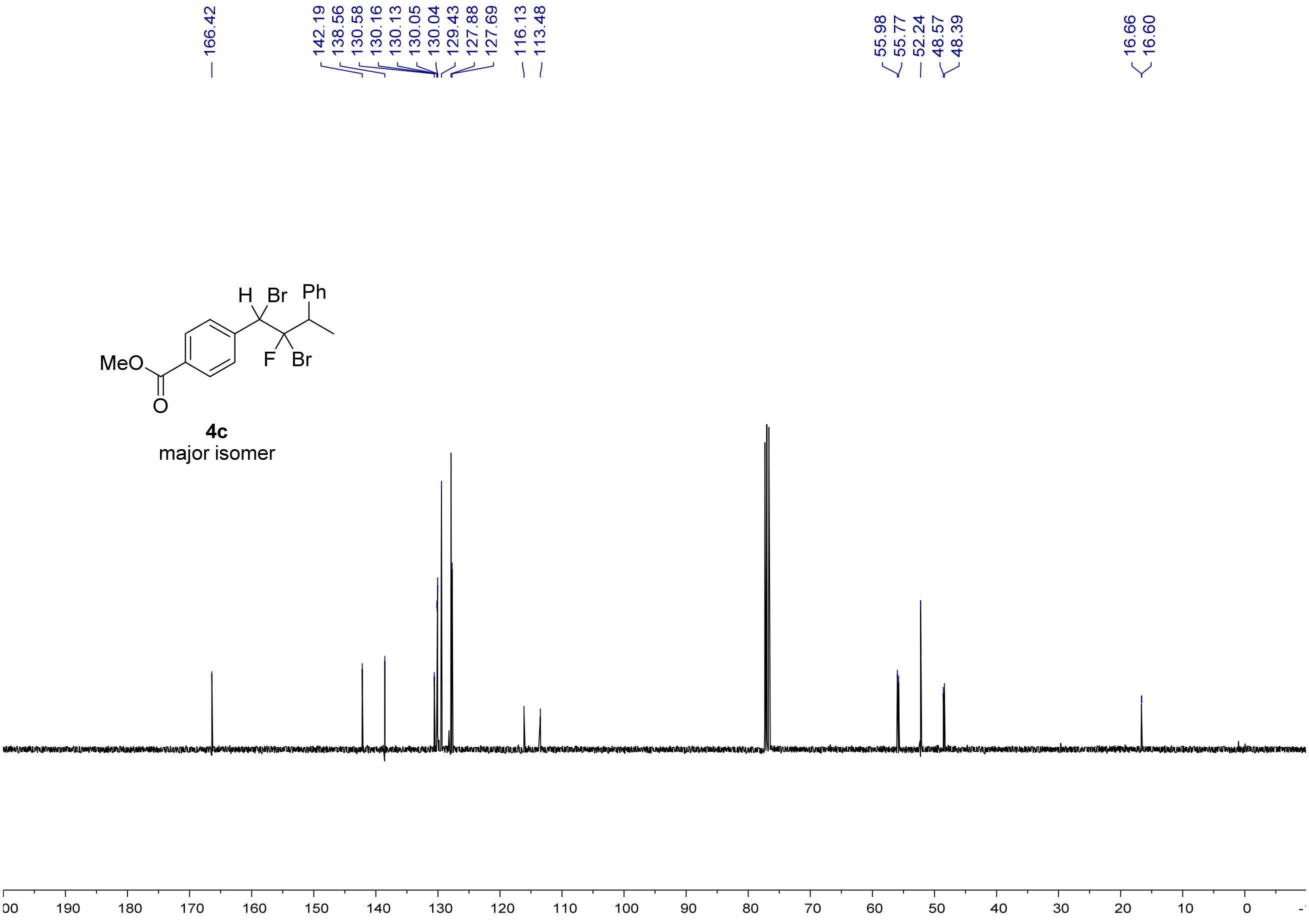
166.42
142.19
138.56
130.58
130.16
130.13
130.05
130.04
129.43
127.88
127.69
116.13
113.48
55.98
55.77
52.24
48.57
48.39
16.66
16.60
H Br Ph
MeO
O
F Br
4c
major isomer
190 180 170 160 150 140 130 120 110 100 90 80 70 60 50 40 30 20 10 0

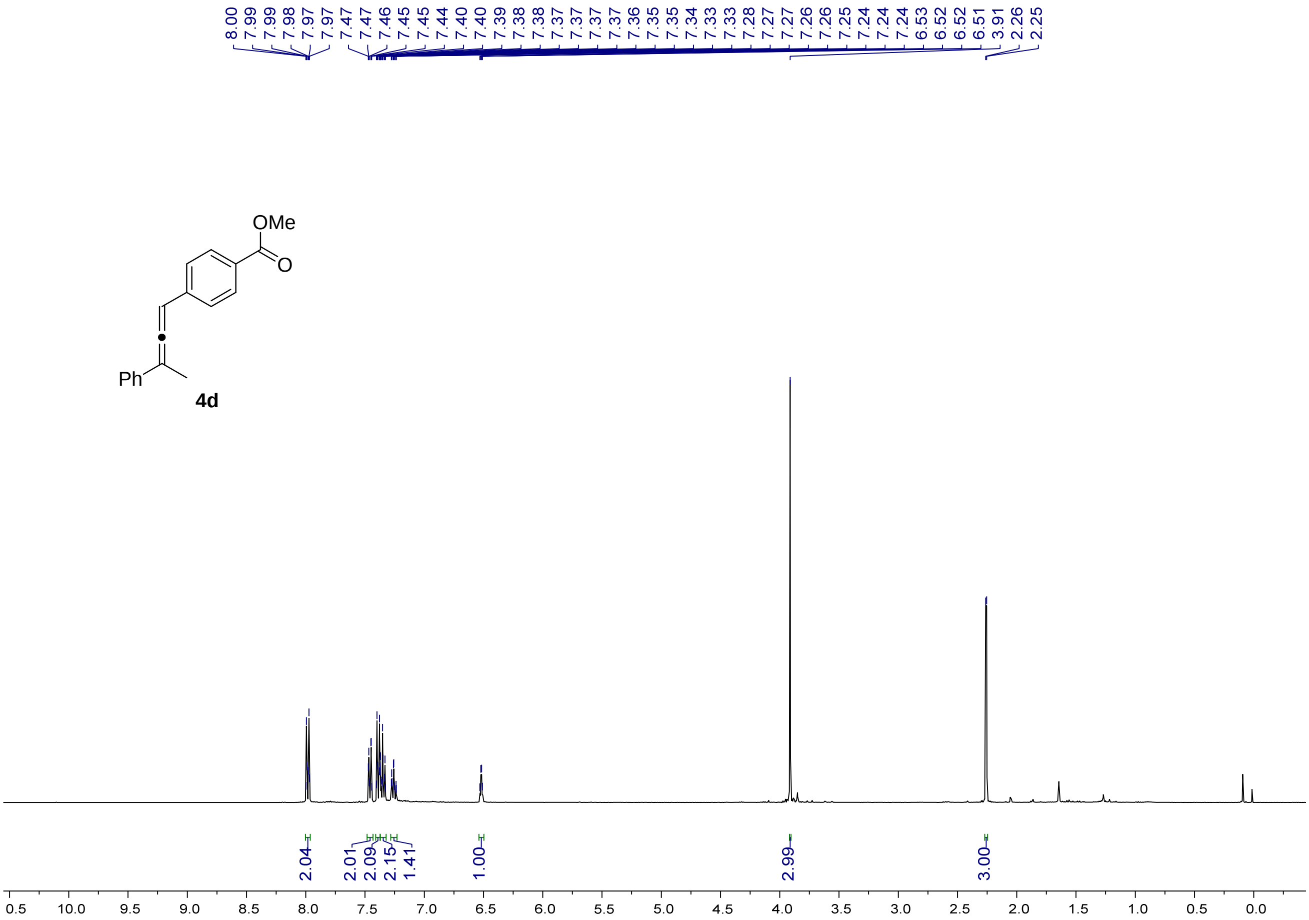
8.00 7.99 7.99 7.98 7.97 7.97 7.47 7.47 7.46 7.45 7.44 7.40 7.40 7.39 7.38 7.38 7.37 7.37 7.37 7.36 7.35 7.35 7.34 7.33 7.33 7.28 7.27 7.27 7.26 7.26 7.25 7.24 7.24 7.24 6.53 6.52 6.52 6.51 3.91 2.26 2.25
OMe
O
Ph
4d
2.04
2.01
2.09
2.15
1.41
1.00
2.99
3.00
0.5 10.0 9.5 9.0 8.5 8.0 7.5 7.0 6.5 6.0 5.5 5.0 4.5 4.0 3.5 3.0 2.5 2.0 1.5 1.0 0.5 0.0

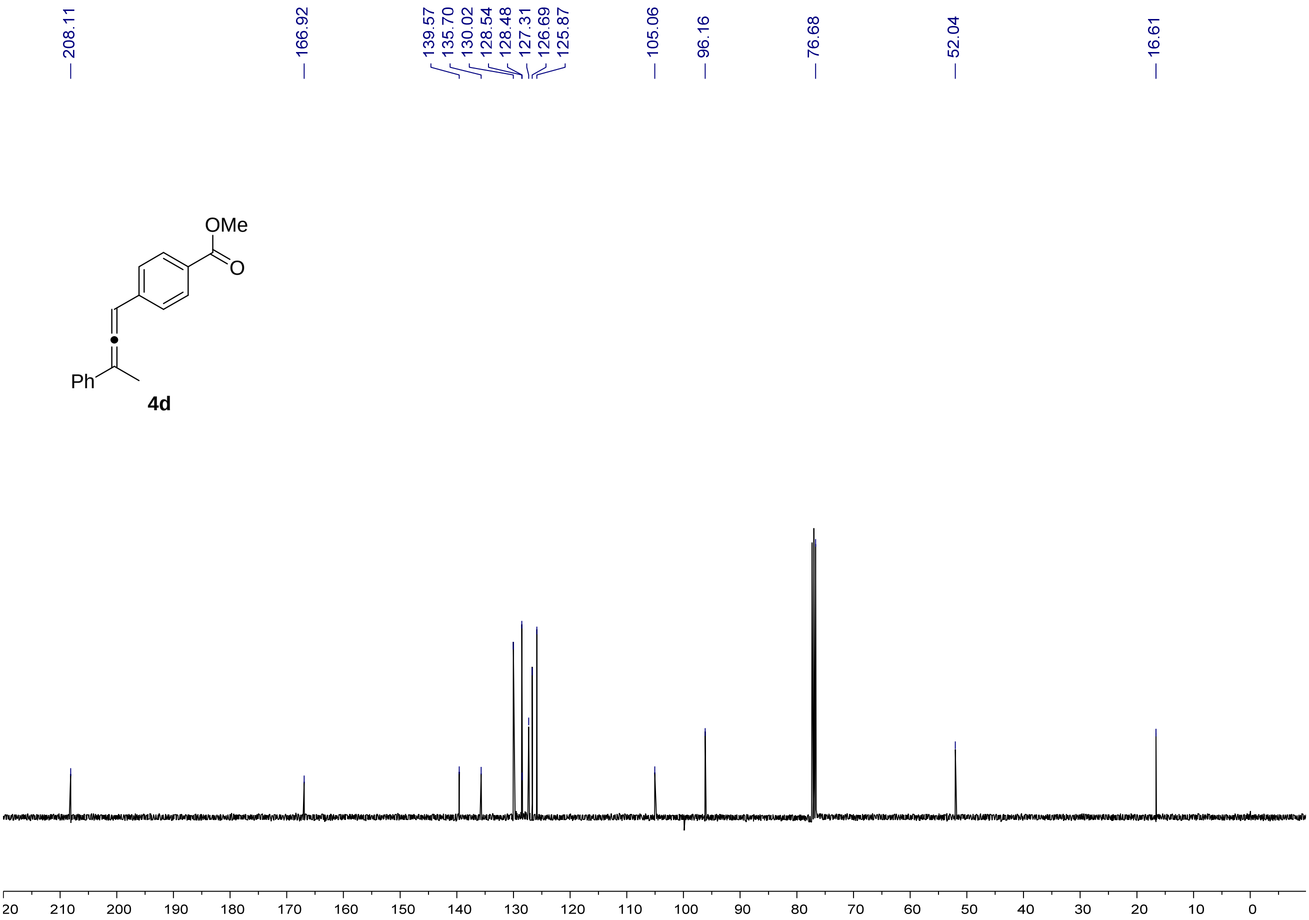
208.11
166.92
139.57 135.70 130.02 128.54 128.48 127.31 126.69 125.87
105.06
96.16
76.68
52.04
16.61
OMe
O
Ph
4d
20 210 200 190 180 170 160 150 140 130 120 110 100 90 80 70 60 50 40 30 20 10 0

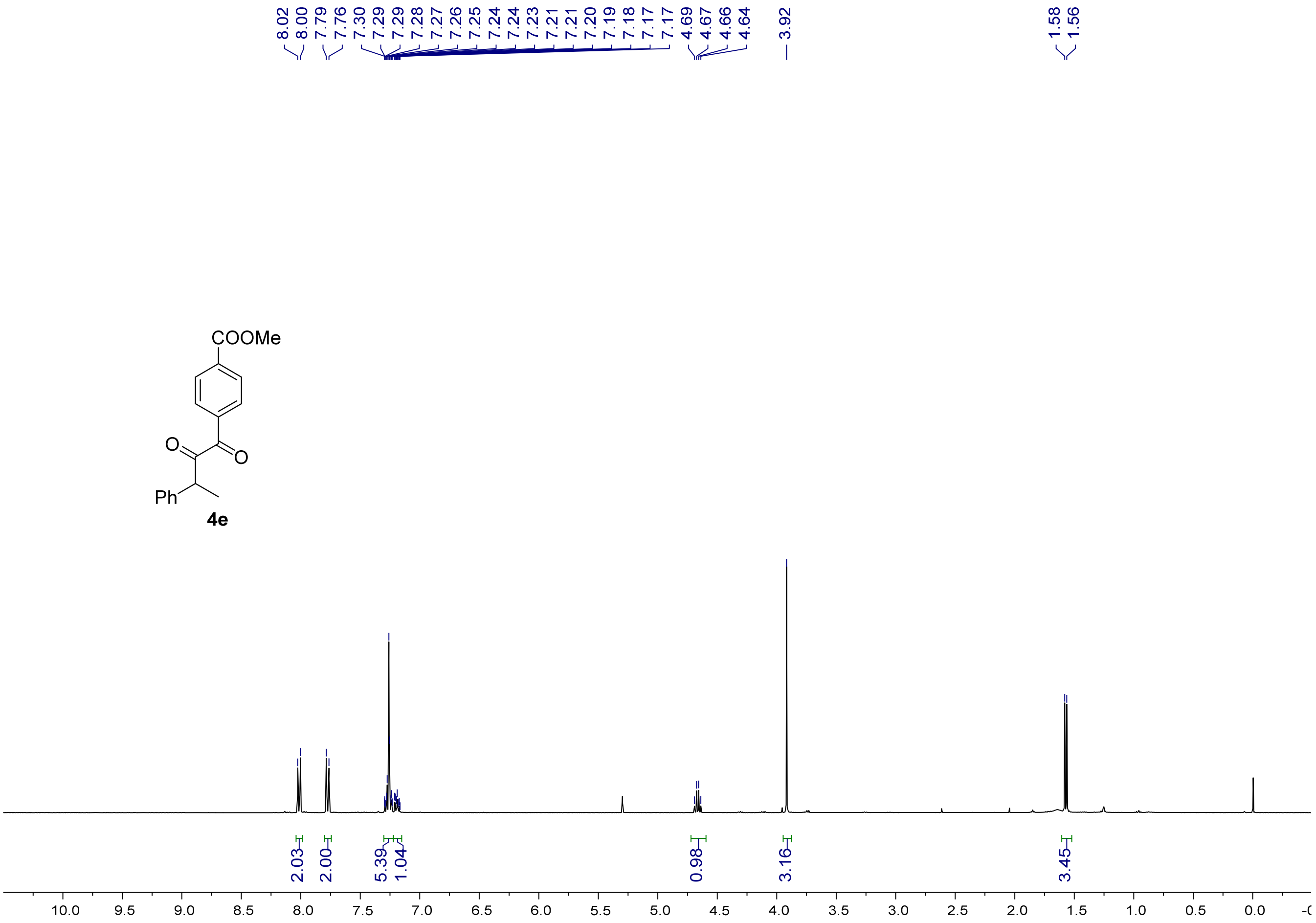
8.02
8.00
7.79
7.76
7.30
7.29
7.29
7.28
7.27
7.26
7.25
7.24
7.24
7.23
7.21
7.21
7.20
7.19
7.18
7.17
7.17
4.69
4.67
4.66
4.64
3.92
1.58
1.56
COOMe
O
O
Ph
4e
2.03
2.00
5.39
1.04
0.98
3.16
3.45
10.0 9.5 9.0 8.5 8.0 7.5 7.0 6.5 6.0 5.5 5.0 4.5 4.0 3.5 3.0 2.5 2.0 1.5 1.0 0.5 0.0

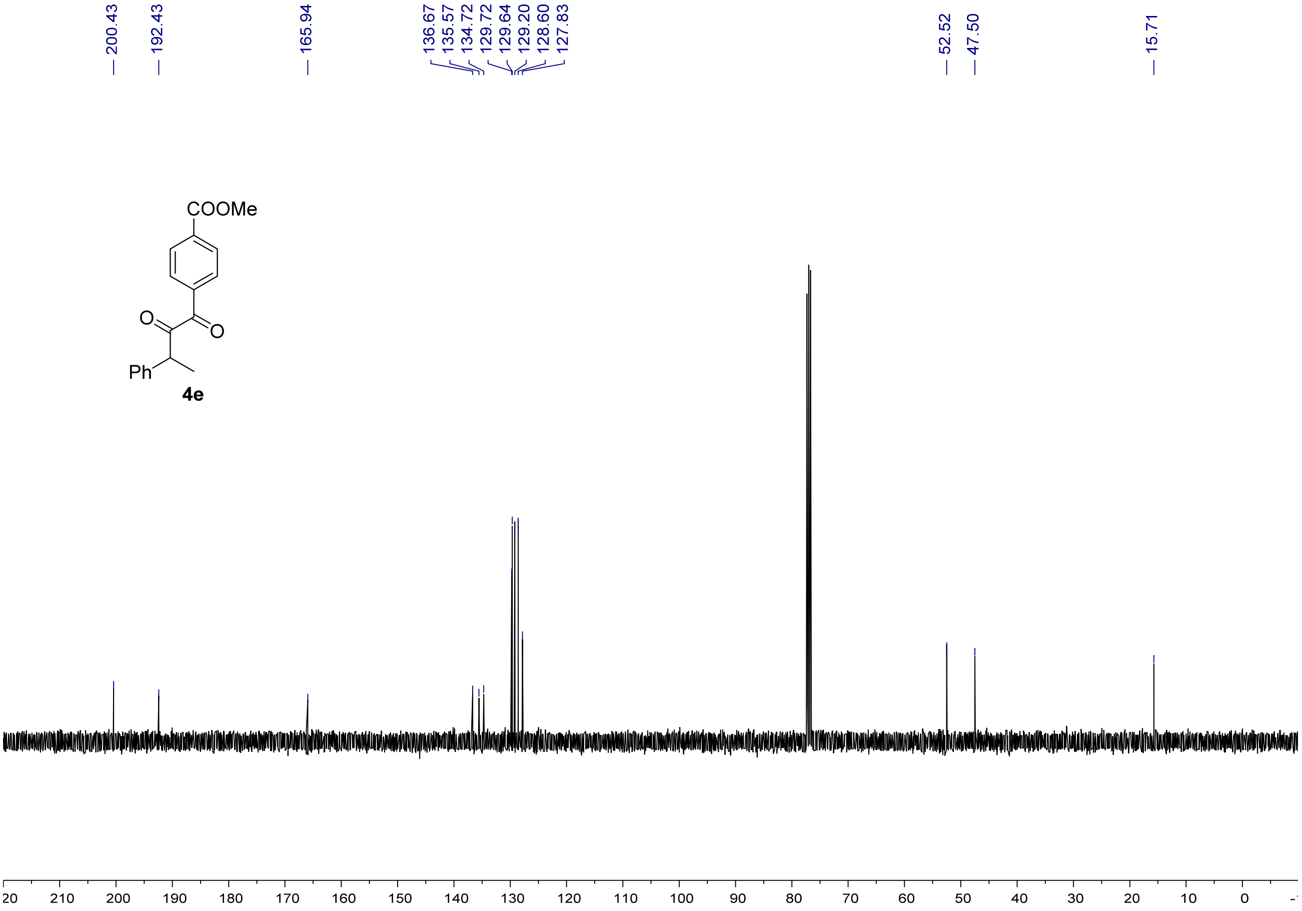
200.43
192.43
165.94
136.67
135.57
134.72
129.72
129.64
129.20
128.60
127.83
52.52
47.50
15.71
COOMe
O
O
Ph
4e
210 200 190 180 170 160 150 140 130 120 110 100 90 80 70 60 50 40 30 20 10 0

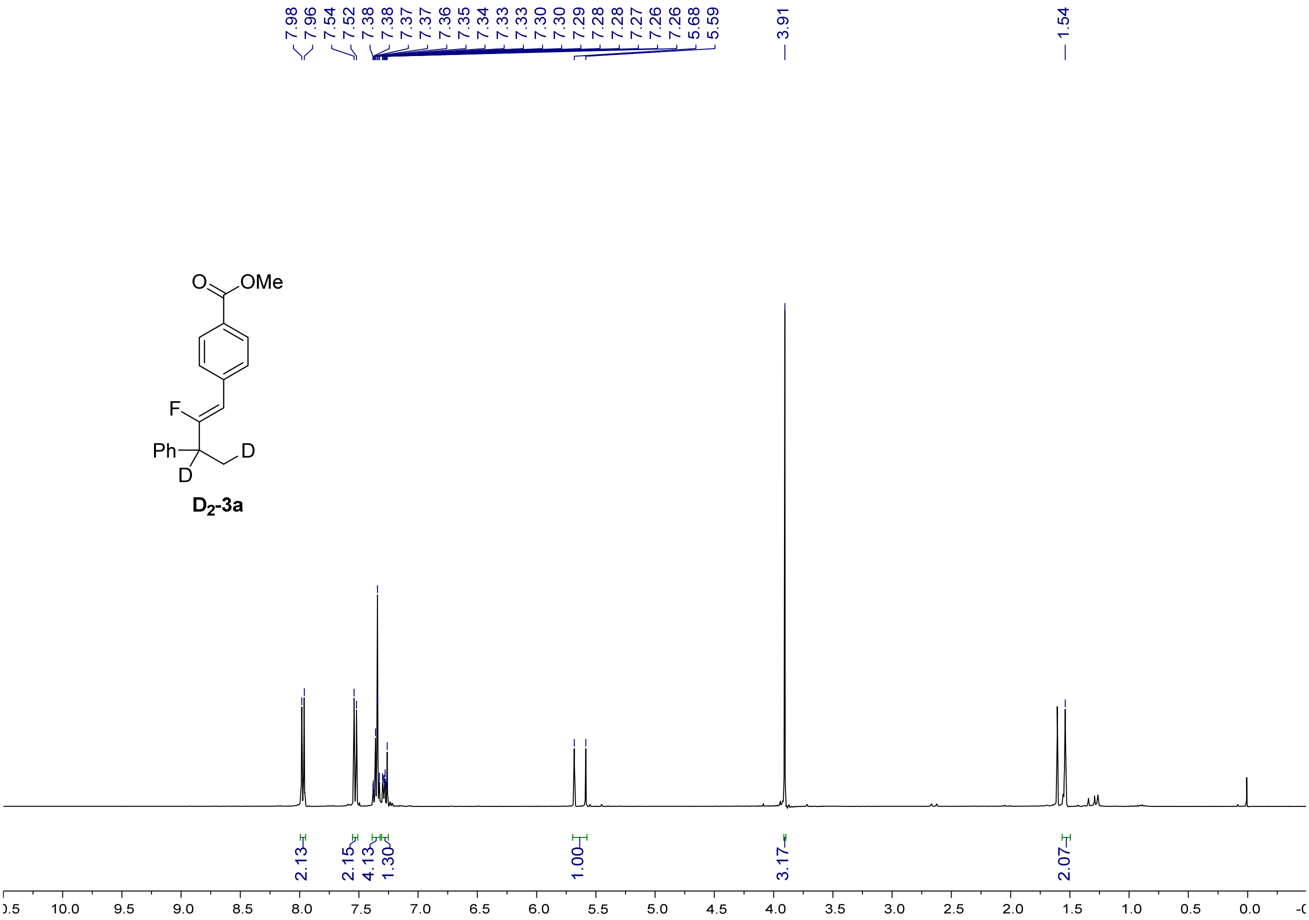
7.98
7.96
7.54
7.52
7.38
7.38
7.37
7.37
7.36
7.35
7.34
7.33
7.33
7.30
7.29
7.28
7.28
7.27
7.26
7.26
5.68
5.59
3.91
1.54
O
OMe
F
Ph
D
D
D2-3a
2.13
2.15
4.13
1.30
1.00
3.17
2.07

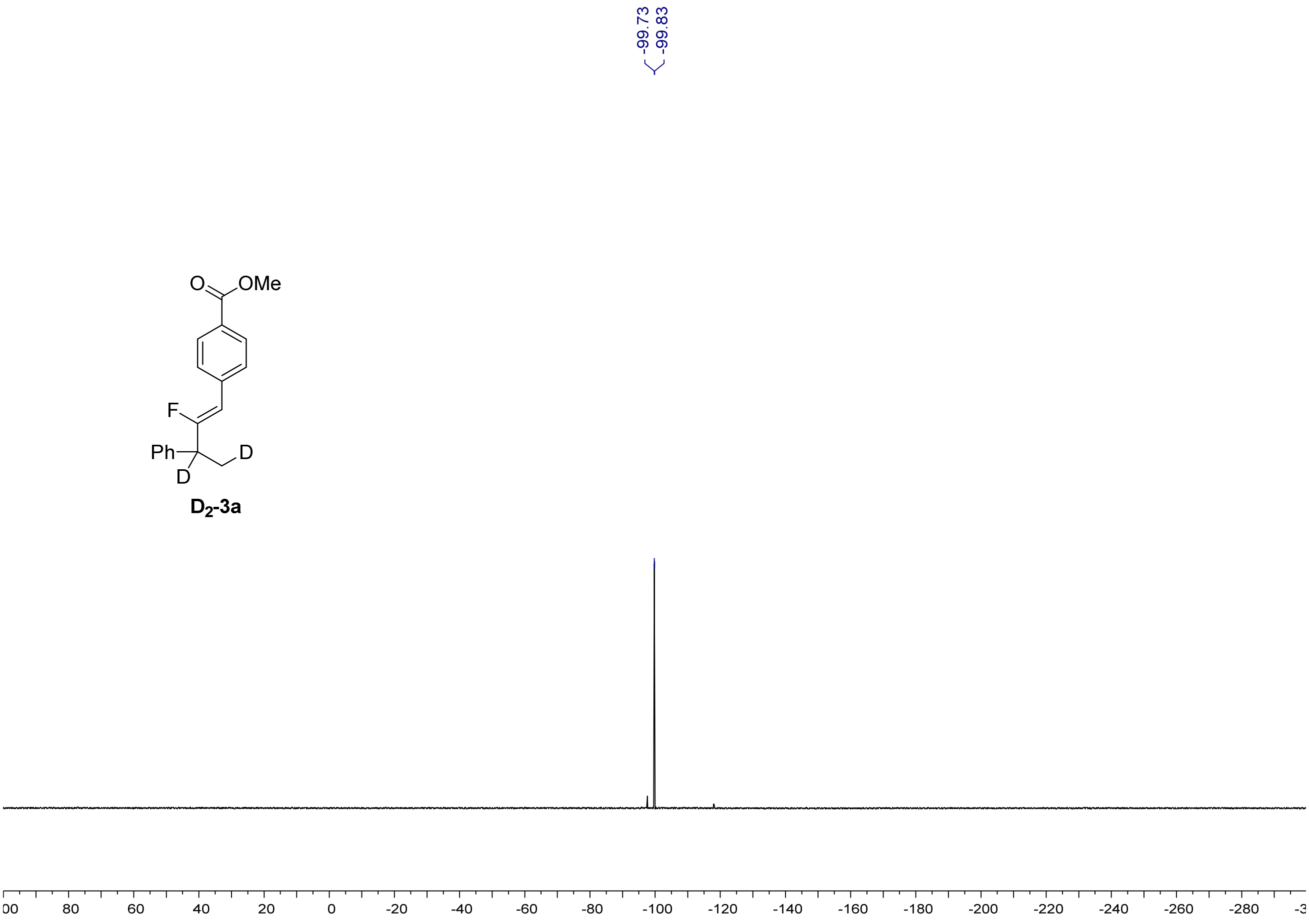
-99.73
-99.83
O
OMe
F
Ph
D
D
D2-3a

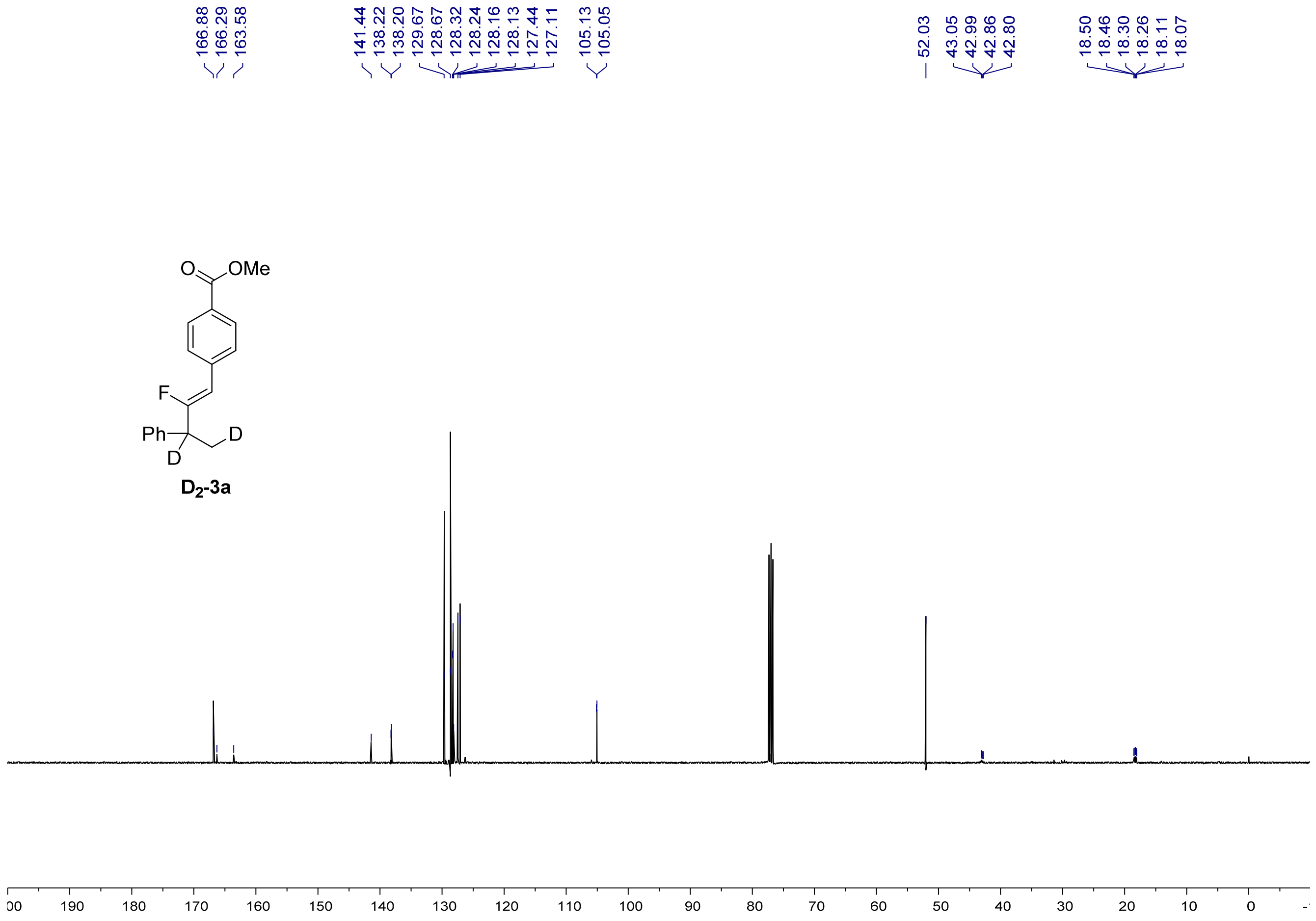
166.88
166.29
163.58
141.44
138.22
138.20
129.67
128.67
128.32
128.24
128.16
128.13
127.44
127.11
105.13
105.05
52.03
43.05
42.99
42.86
42.80
18.50
18.46
18.30
18.26
18.11
18.07
OMe
O
F
Ph
D
D
D2-3a
190
180
170
160
150
140
130
120
110
100
90
80
70
60
50
40
30
20
10
0

9. Determination of regioisomeric ratio

The regioisomeric ratio of **3a** was determined by ^{19}F NMR of the crude products comparing with the linear regioisomer of **3a** (**3a'**) which was prepared by Pd-catalysis. The regioisomeric ratios of **3b-3ac** were determined according to the ^{19}F NMR analysis of the crude products by analogy to **3a**/**3a'**.

Preparation of the methyl (Z)-4-(2-fluoro-4-phenylbut-1-en-1-yl)benzoate (**3a'**)

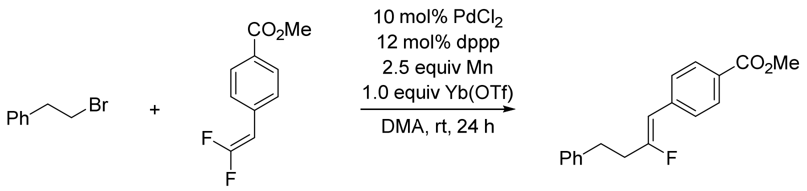

1a **2a** **3a'**

To an oven-dried schlenk tube equipped with a magnetic stir bar was added $PdCl_2$ (3.7 mg, 0.01 mmol, 5.0 mol%), L1 (2.2 mg, 0.012 mmol, 6.0 mol%), **1a** (0.50 mmol, 2.5 equiv), the *gem*-difluoroalkene **2a** (0.20 mmol, 1.0 equiv) and Mn powder (26 mg, 0.50 mmol, 2.5 equiv). The Schlenk tube was evacuated and filled with nitrogen for three times. To these solids, DMA (1.0 mL) was added under N_2 atmosphere. After stirring at room temperature for 24 h. The reaction mixture was diluted with ethyl acetate (10 mL) and filtered through a short pad of silica gel. The filtrate was washed with water (10 mL × 2), brine (10 mL) and dried over Na_2SO_4. After solvent was removed under reduced pressure, the crude residue was purified by column chromatography or preparative TLC on silica gel (petroleum ether/ethyl acetate = 50 : 1) to afford the **3a'** (23.8 mg, 0.084 mmol) in 42% yield as pale yellow oil. **^{1}H NMR (400 MHz, CDCl$_3$):** δ = 7.90 (d, *J* = 8.4 Hz, 2H), 7.42 (d, *J* = 8.4 Hz, 2H), 7.26 – 7.20 (m, 2H), 7.18 – 7.12 (m, 3H), 5.42 (d, *J* = 39.2 Hz, 1H), 3.83 (s, 3H), 2.86 (t, *J* = 7.6 Hz, 2H), 2.58 (dt, *J* = 18.0, 7.6 Hz, 2H). **^{19}F NMR (376 MHz, CDCl$_3$):** δ = -97.59 (dt, *J* = 39.2, 17.7 Hz). **^{13}C NMR (100 MHz, CDCl$_3$):** δ = 166.9, 161.8 (d, *J* = 270.2 Hz), 140.3, 138.2 (d, *J* = 3.0 Hz), 129.7, 128.5, 128.3, 128.14 , 128.06, 126.3, 105.9 (d, *J* = 8.0 Hz), 52.0, 35.3, 32.6. **HRMS (ESI, m/z):** calcd. for $C_{18}H_{17}FO_2$ [M+H]$^+$: 285.1291, found: 285.1300.

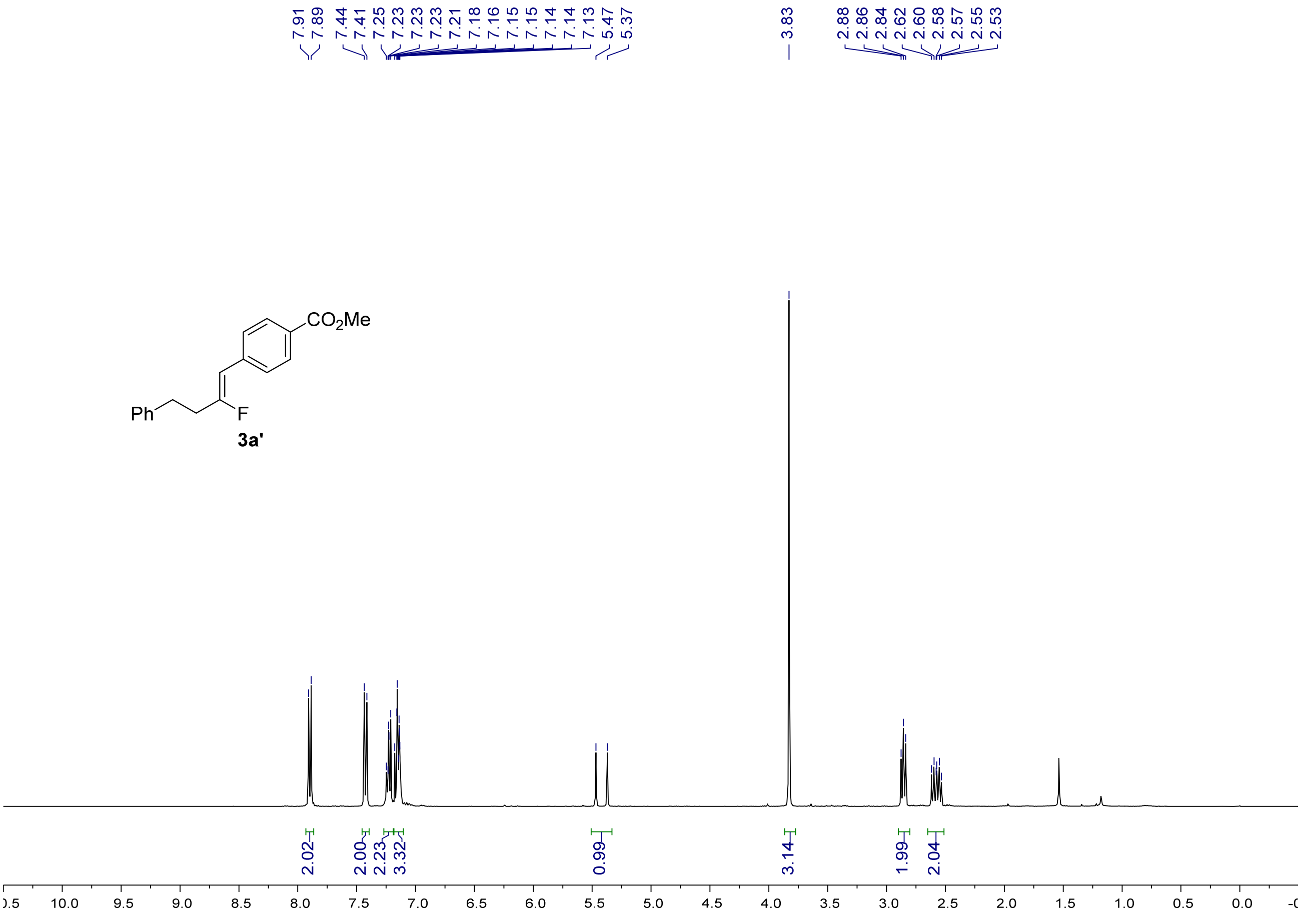
7.91 7.89 7.44 7.41 7.25 7.23 7.23 7.23 7.21 7.18 7.16 7.15 7.15 7.14 7.14 7.13 5.47 5.37
3.83
2.88 2.86 2.84 2.62 2.60 2.58 2.57 2.55 2.53
CO2Me
Ph
F
3a'
2.02 2.00 2.23 3.32 0.99 3.14 1.99 2.04
10.0 9.5 9.0 8.5 8.0 7.5 7.0 6.5 6.0 5.5 5.0 4.5 4.0 3.5 3.0 2.5 2.0 1.5 1.0 0.5 0.0

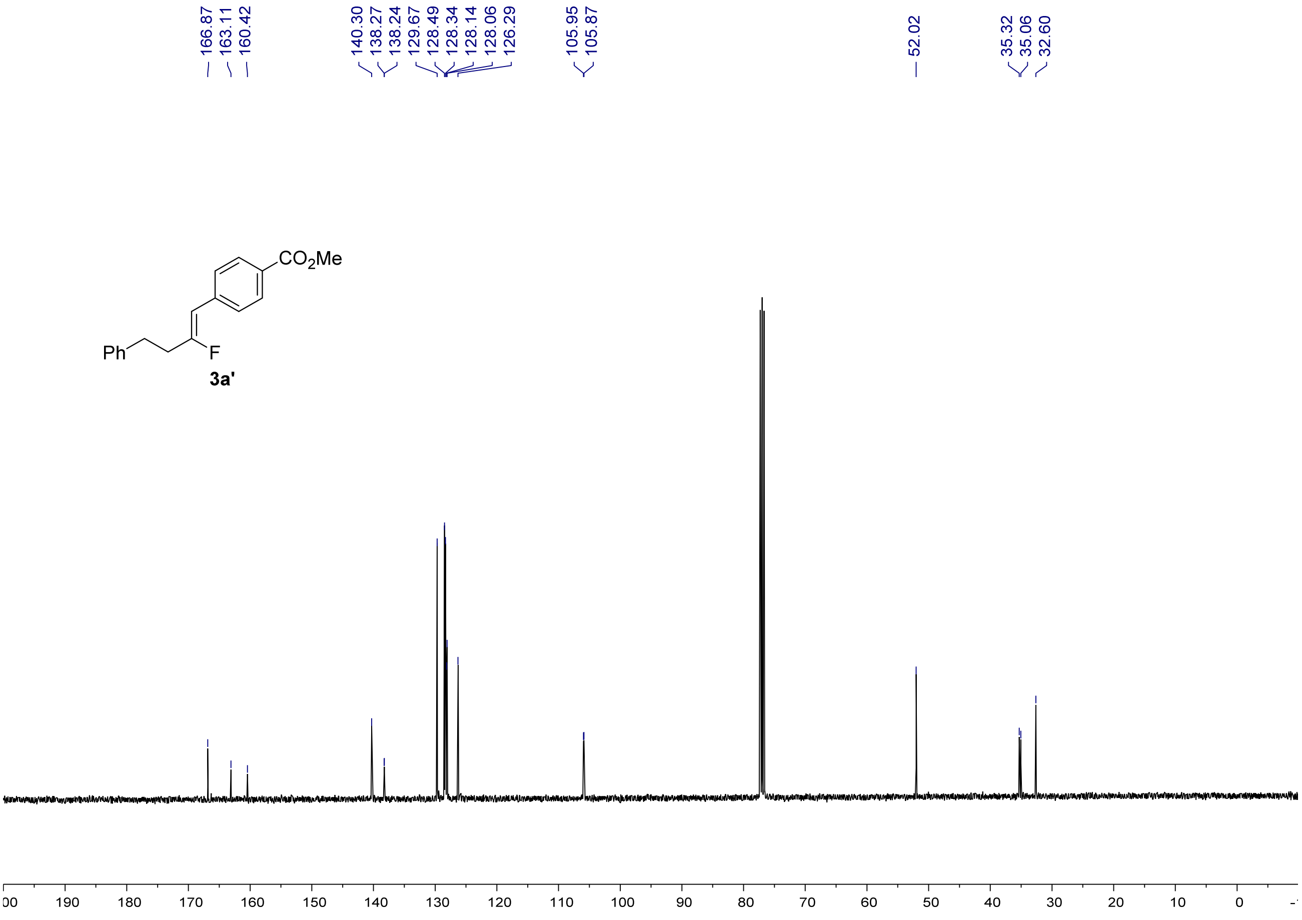
166.87 163.11 160.42
140.30 138.27 138.24 129.67 128.49 128.34 128.14 128.06 126.29
105.95 105.87
52.02
35.32 35.06 32.60
CO2Me
Ph
F
3a'
190 180 170 160 150 140 130 120 110 100 90 80 70 60 50 40 30 20 10 0

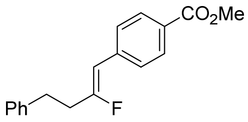
CO2Me
Ph
F

3a'

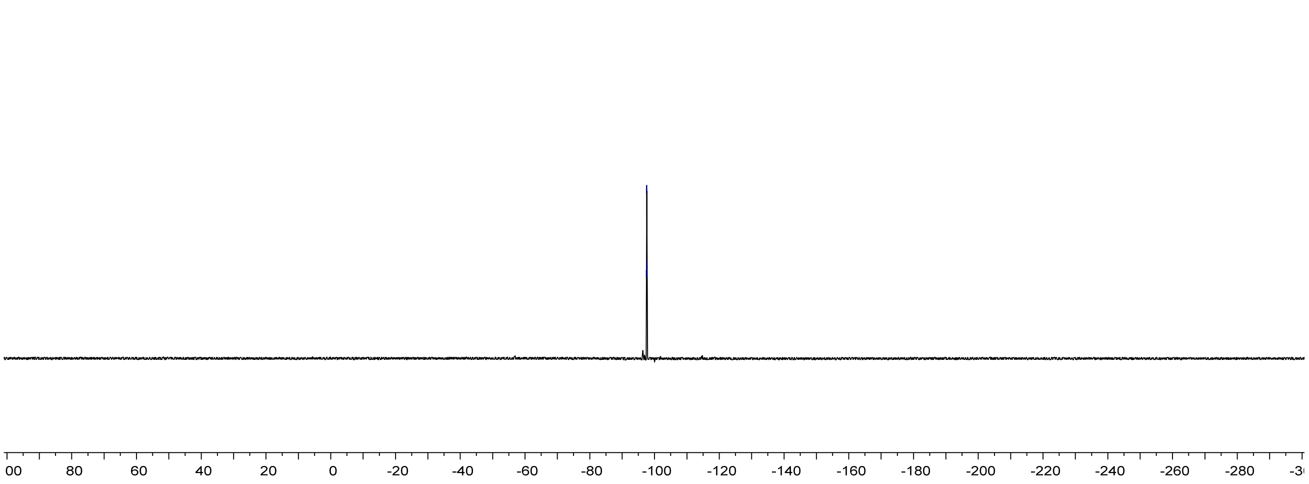
-97.49
-97.54
-97.58
-97.59
-97.64
-97.69
00 80 60 40 20 0 -20 -40 -60 -80 -100 -120 -140 -160 -180 -200 -220 -240 -260 -280

3a: ^{19}F NMR analysis

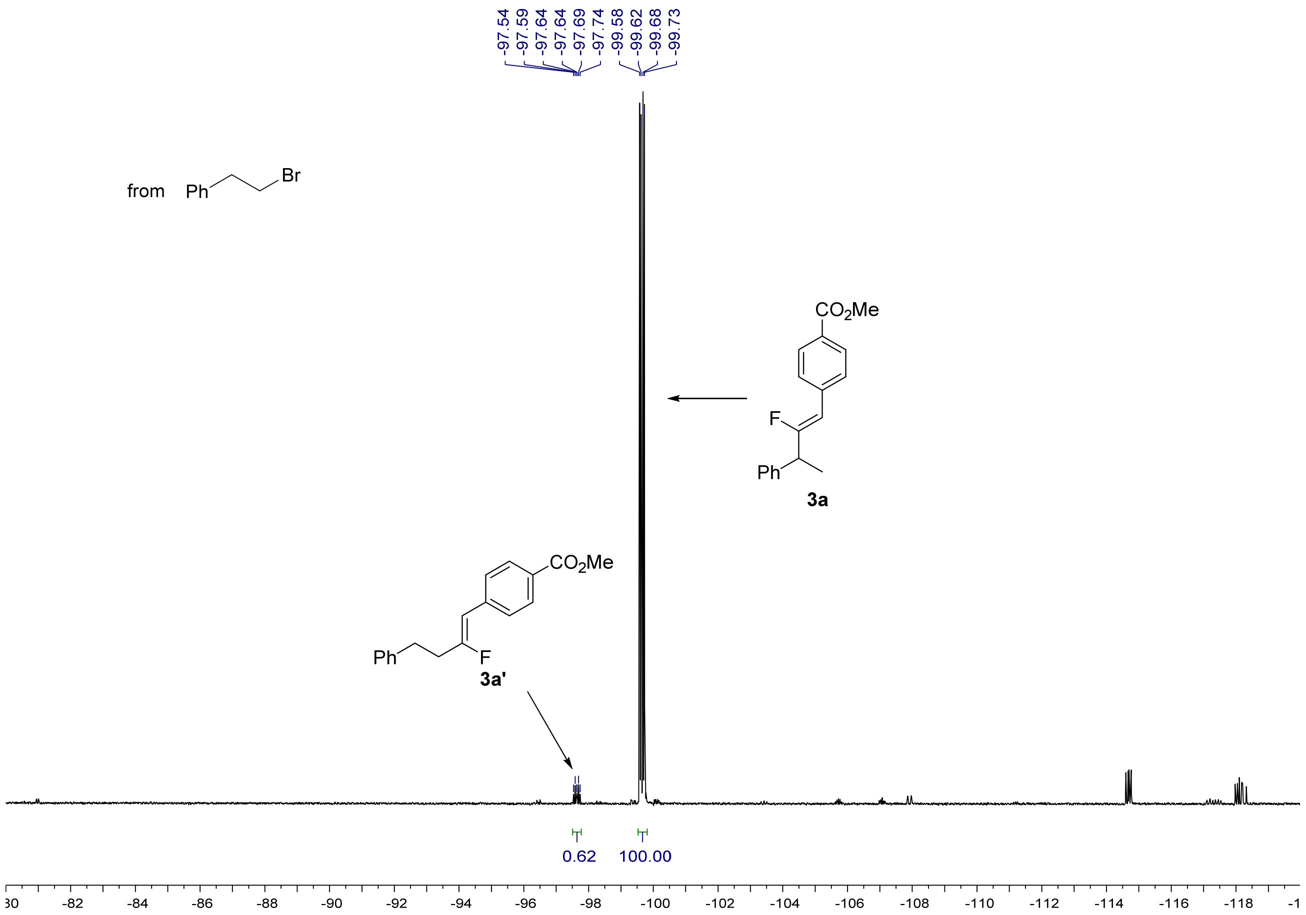

3a: ^{19}F NMR analysis

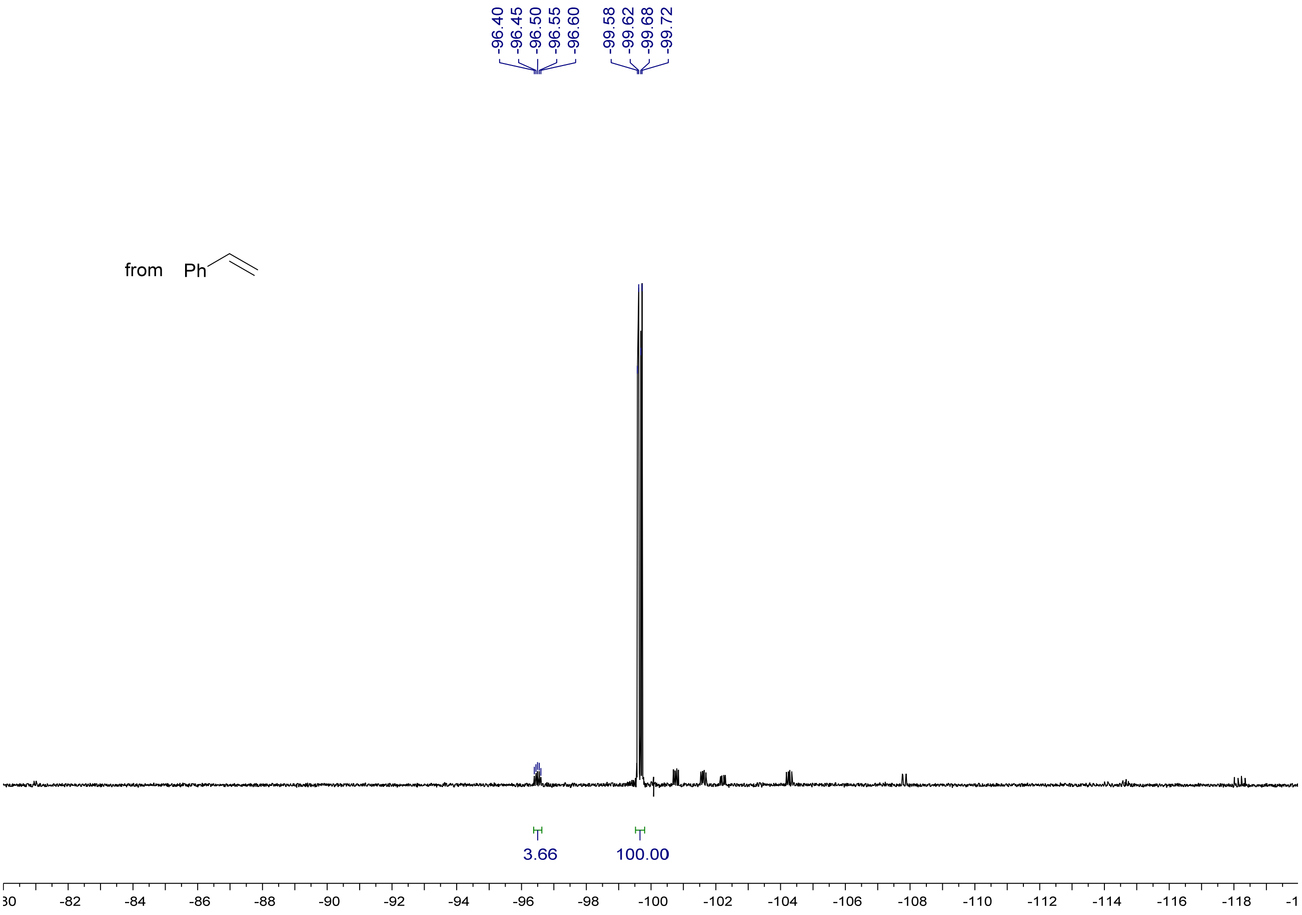

3b: ^{19}F NMR analysis

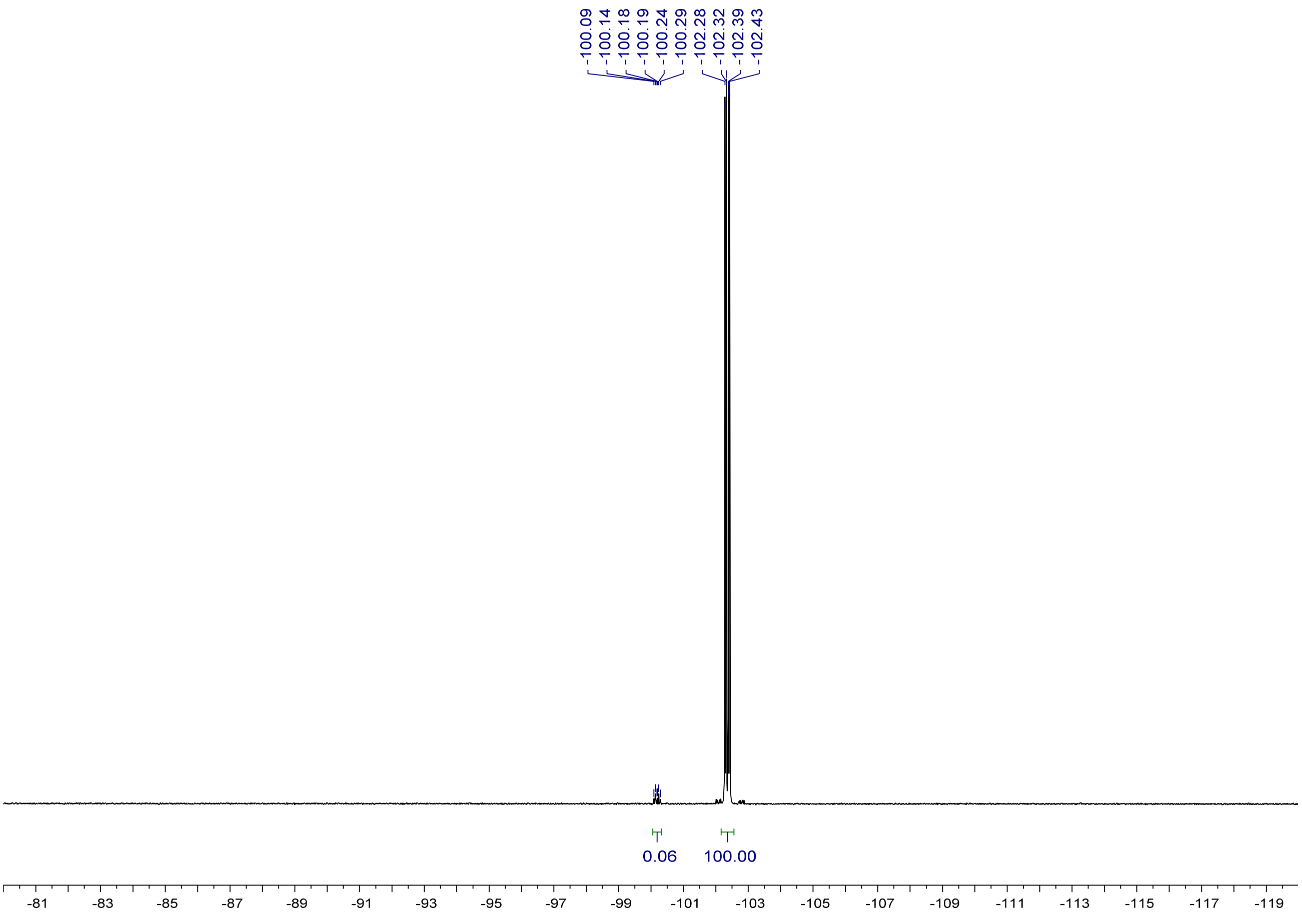

3c: ^{19}F NMR analysis

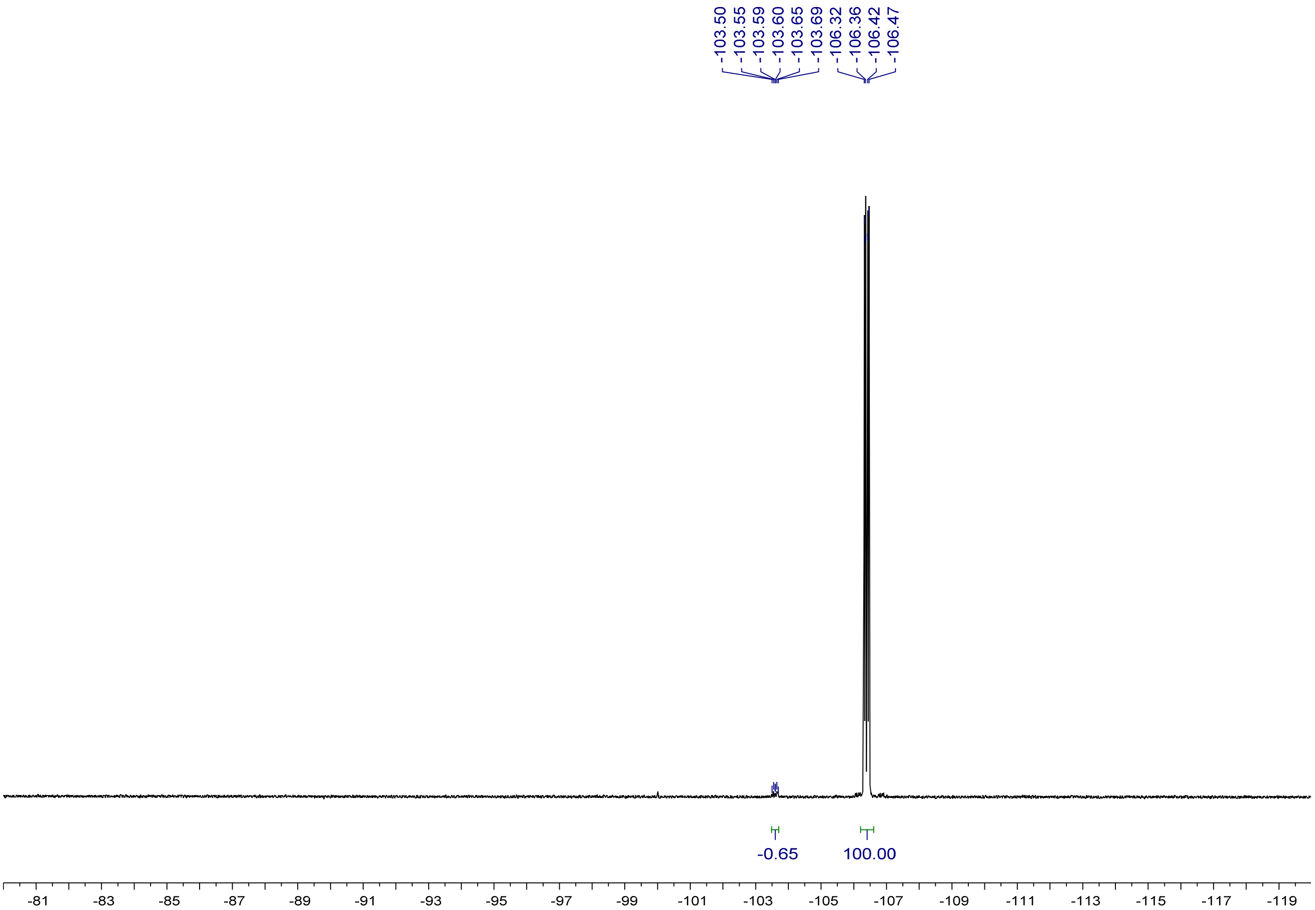

3d: ^{19}F NMR analysis

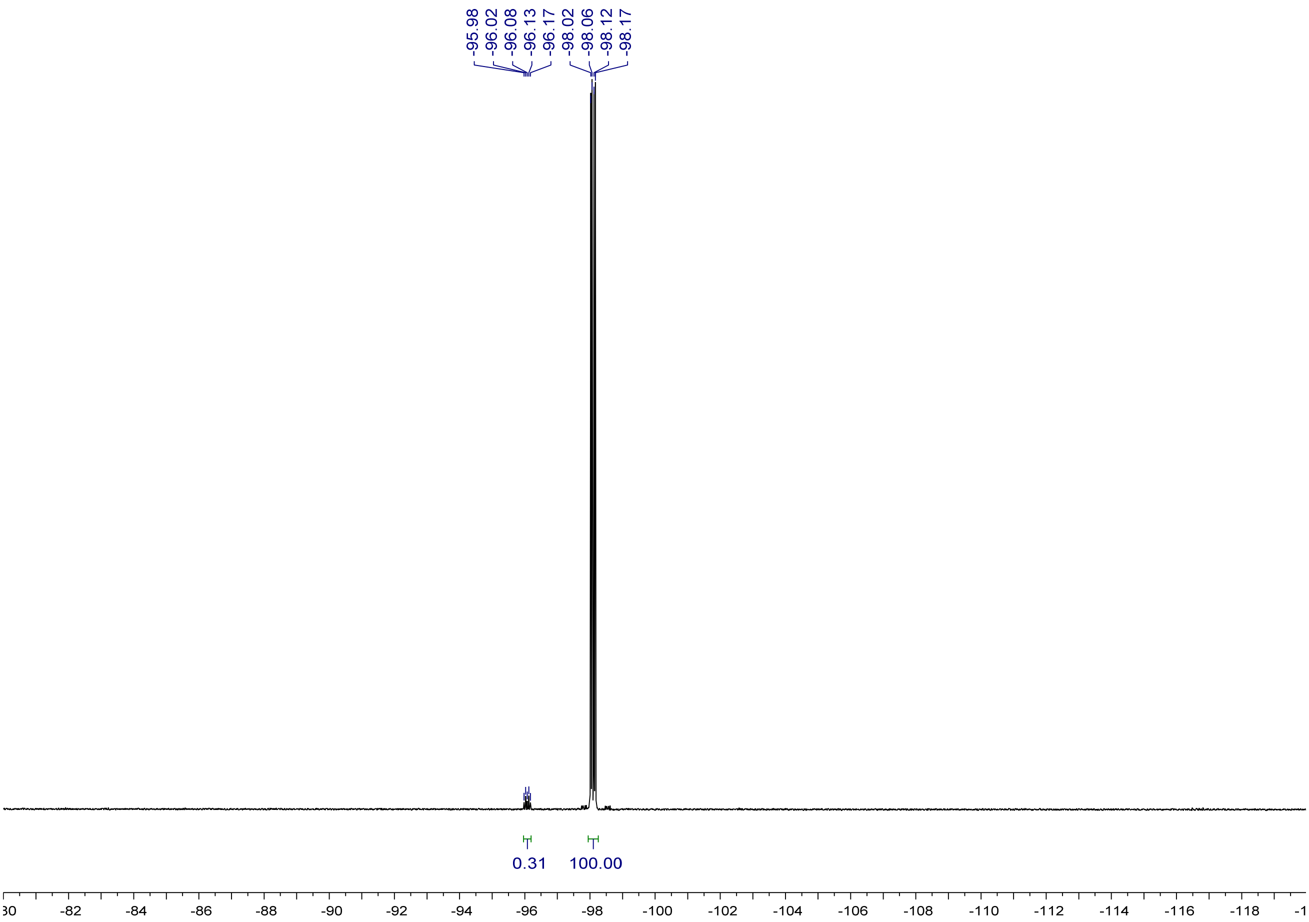

3e: ^{19}F NMR analysis

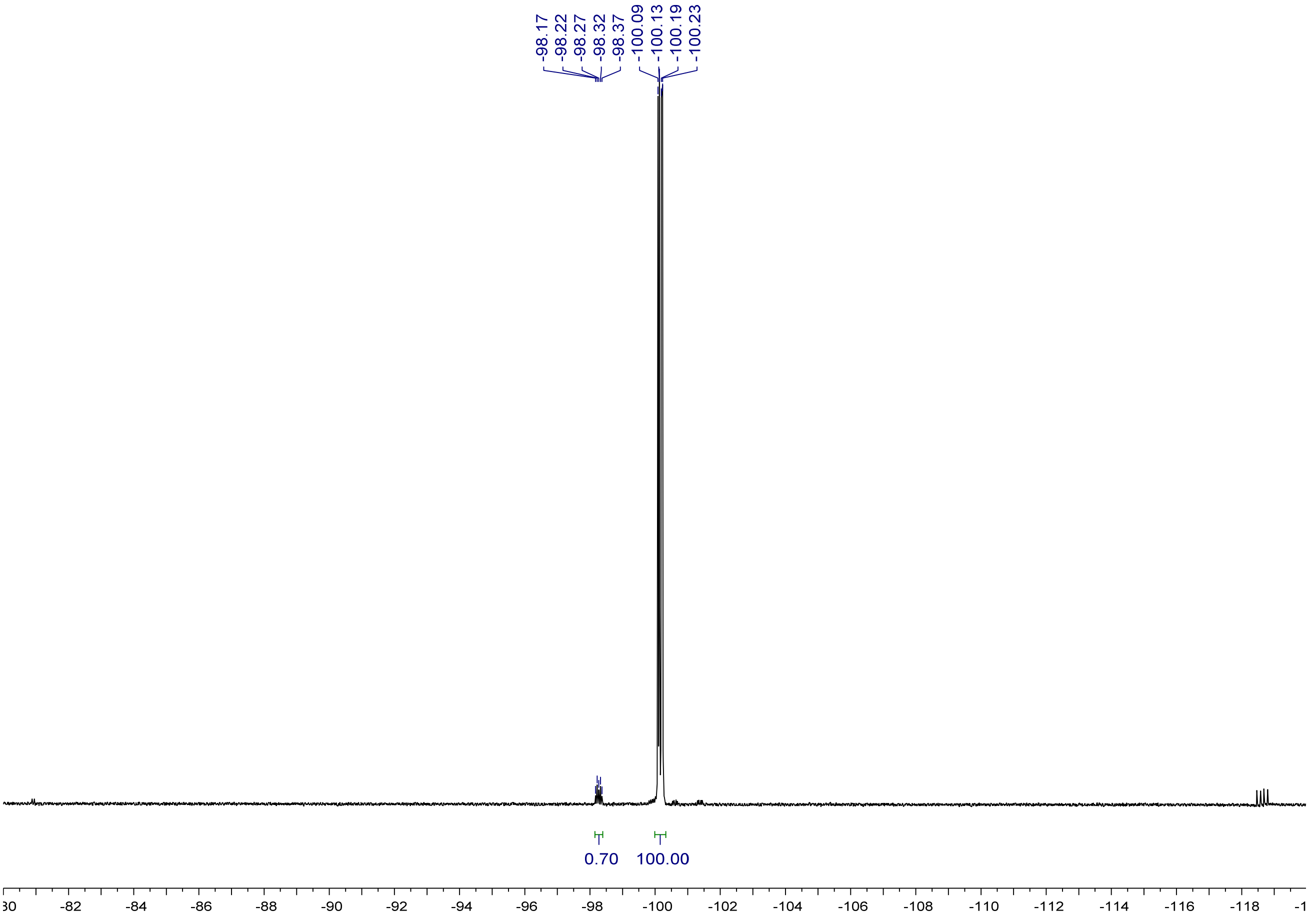

3f: ^{19}F NMR analysis

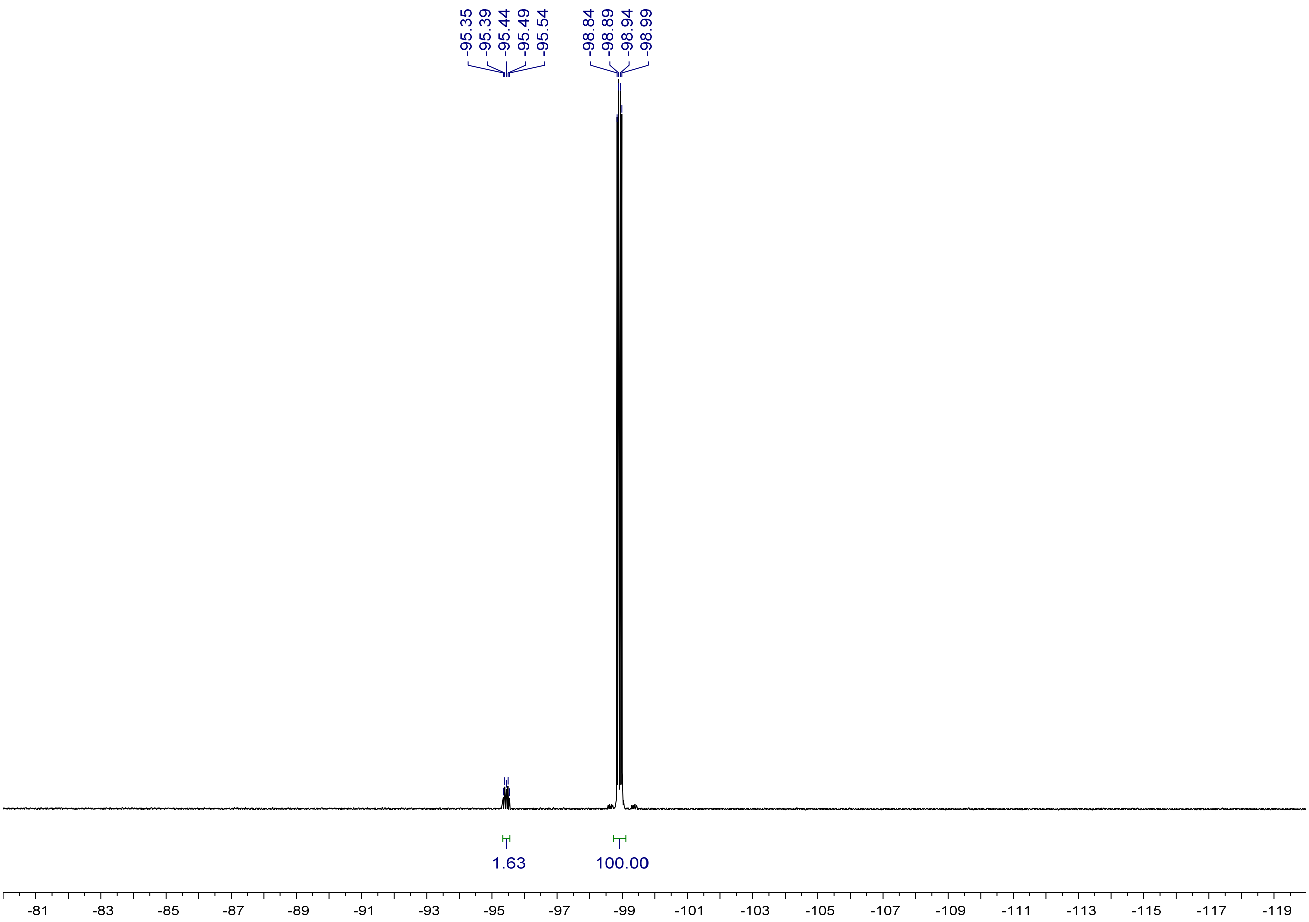

3g: ^{19}F NMR analysis

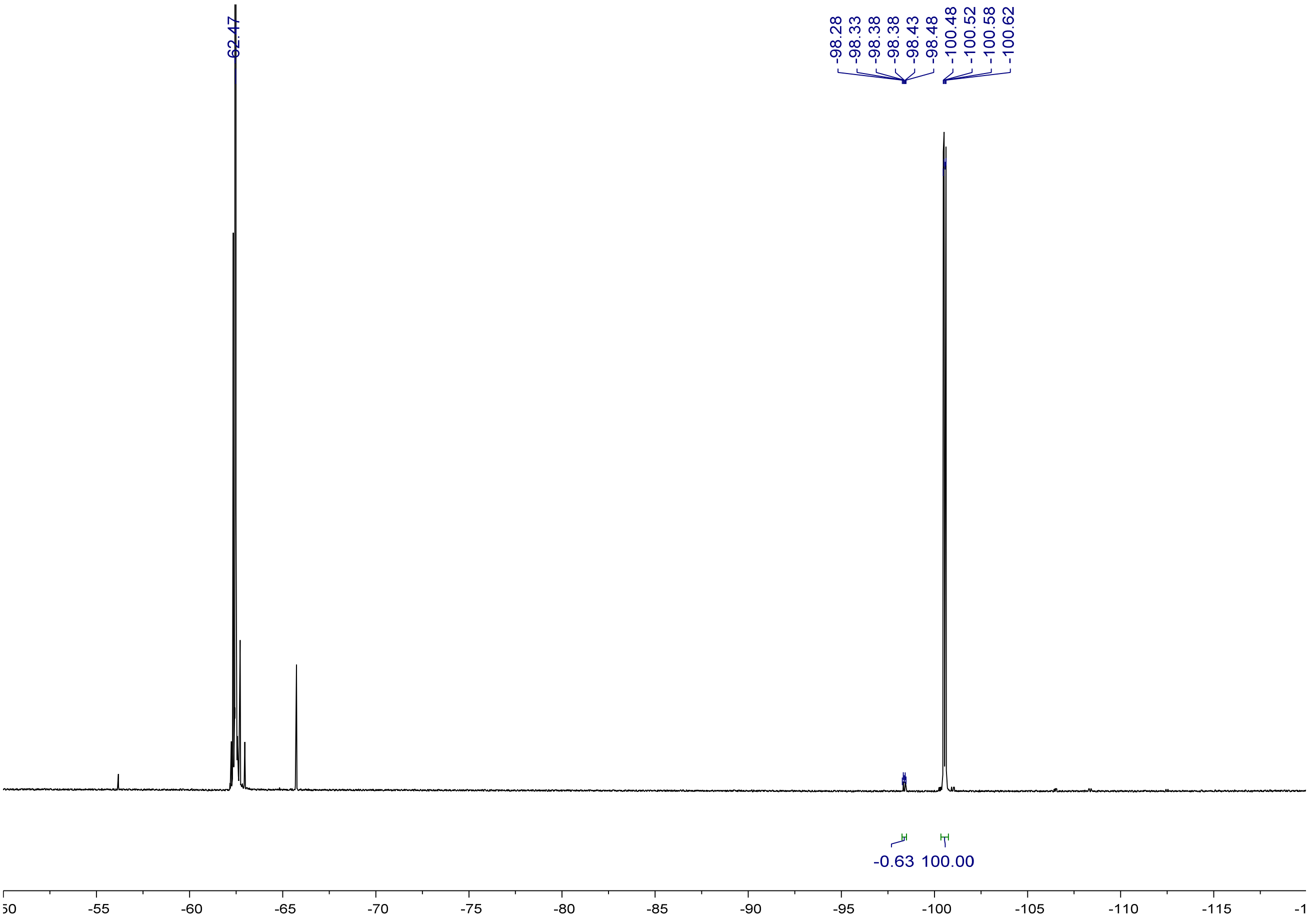

3h: ^{19}F NMR analysis

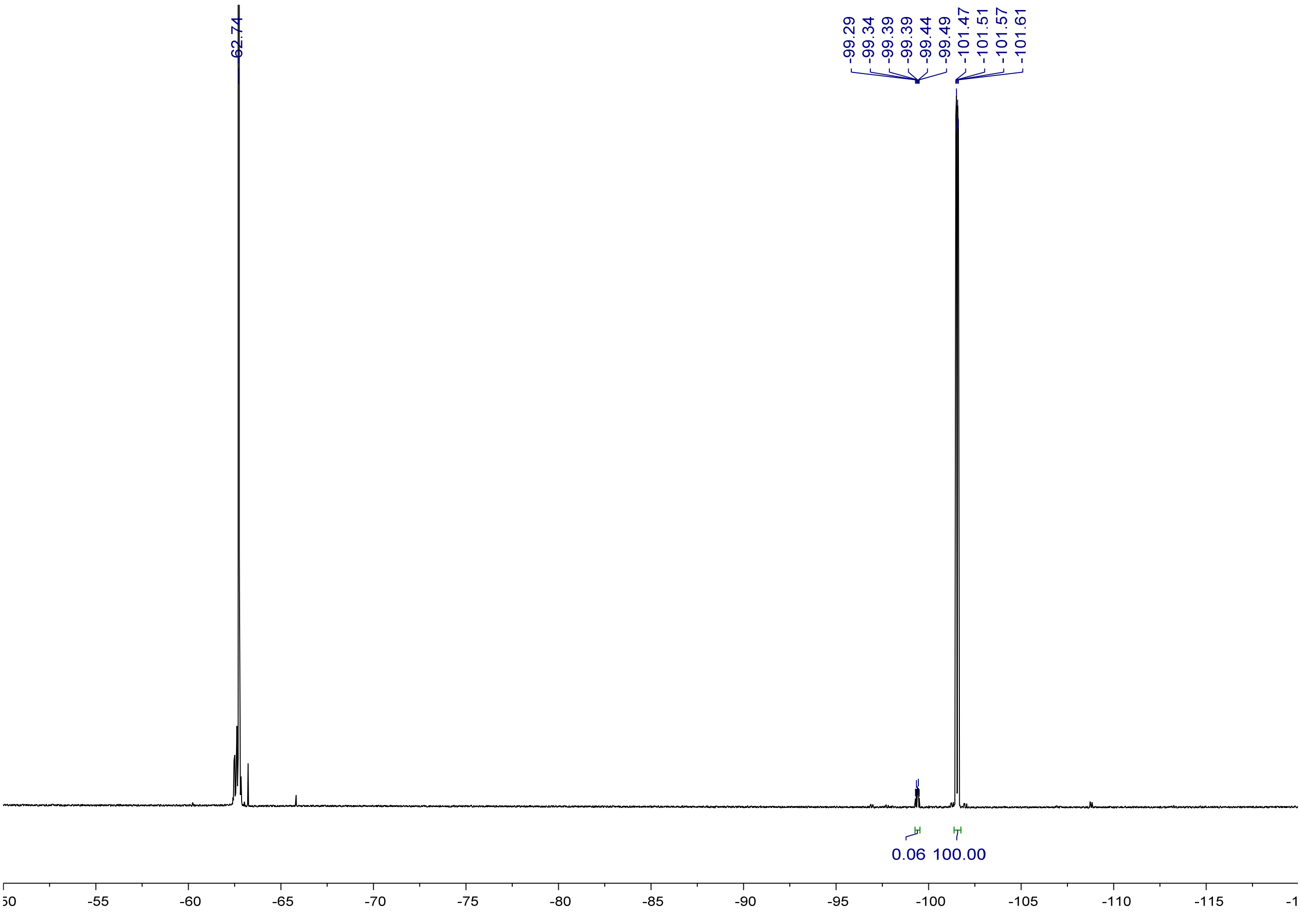

3i: ^{19}F NMR analysis

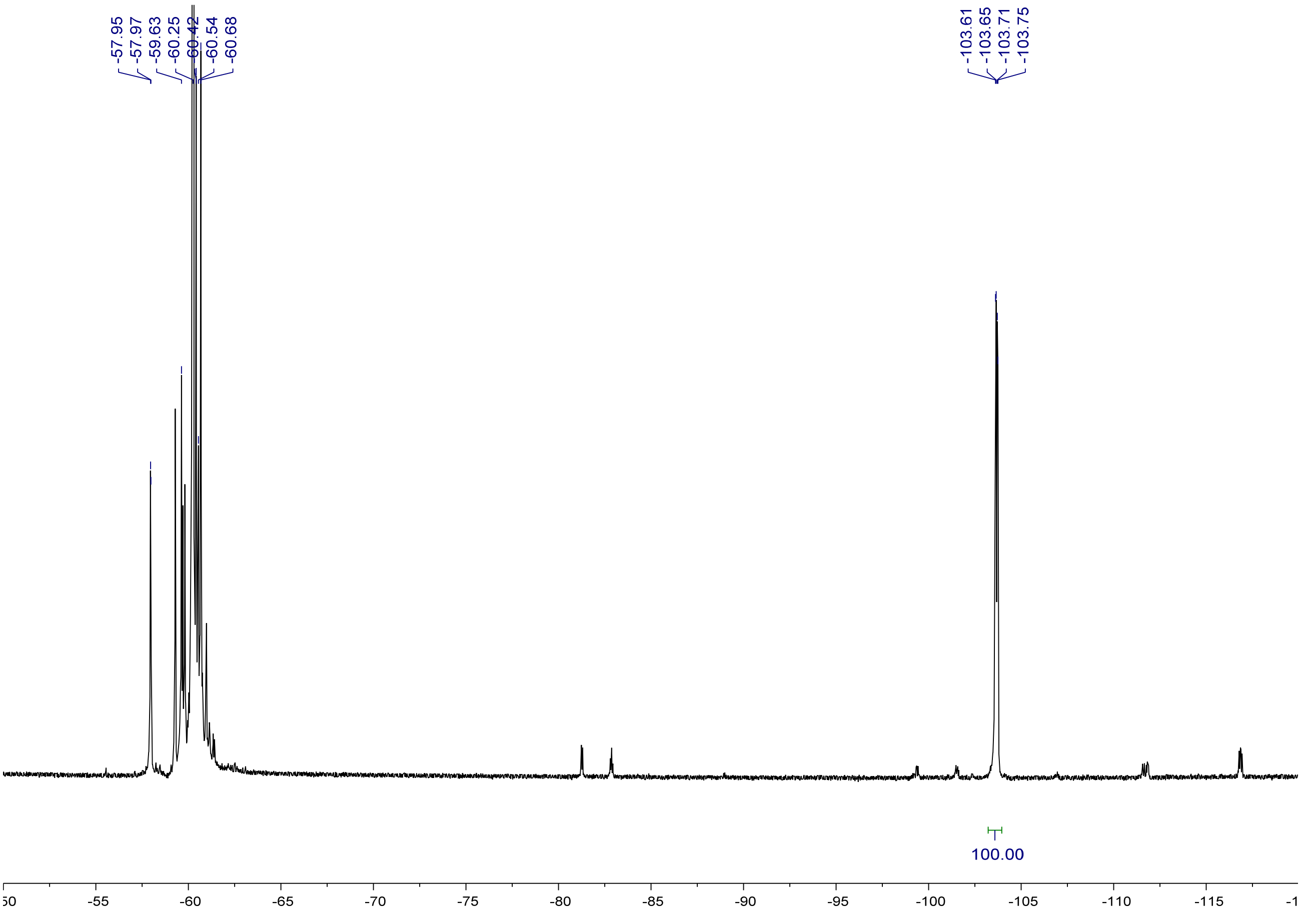

3j: ^{19}F NMR analysis

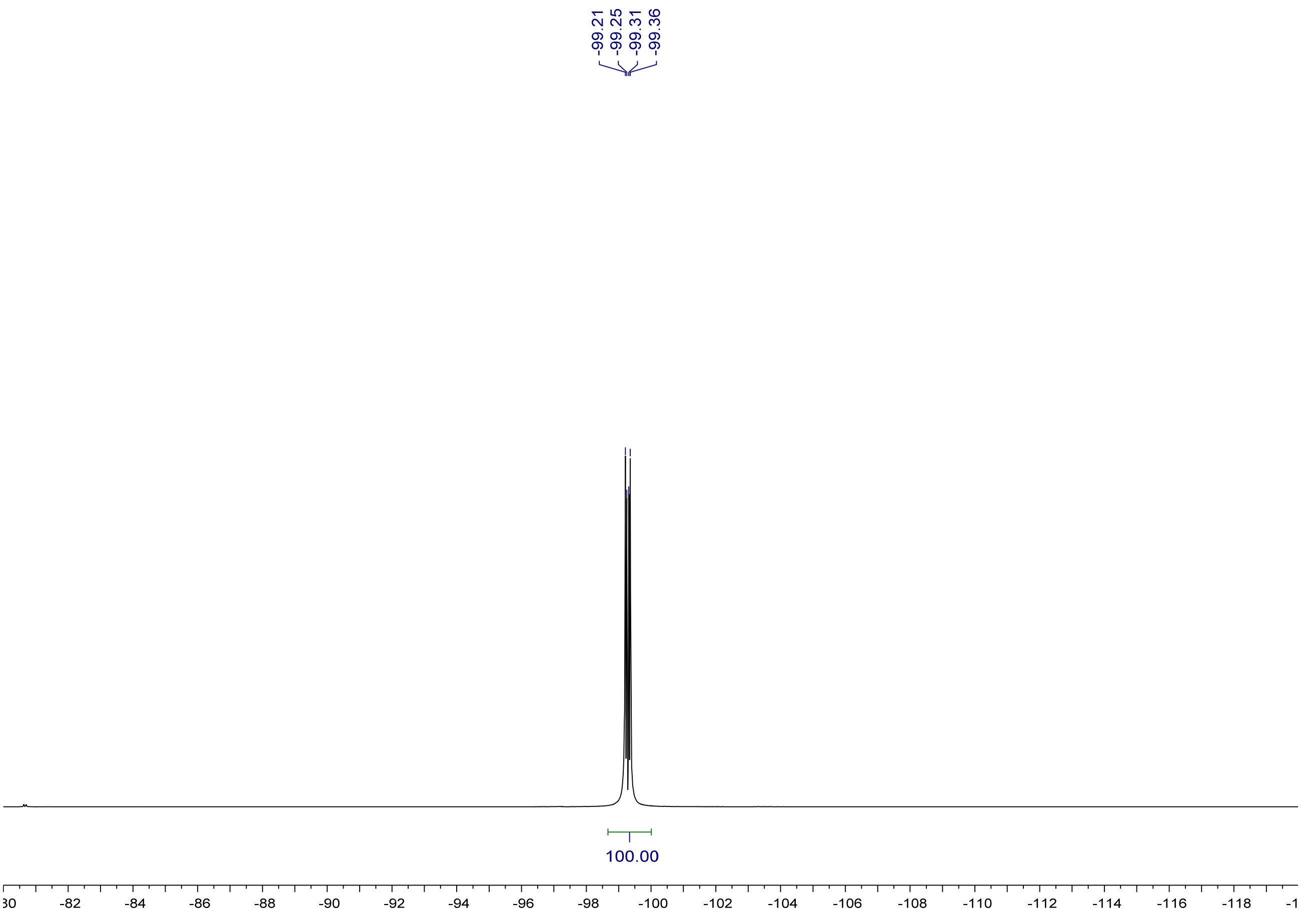

3k: ^{19}F NMR analysis

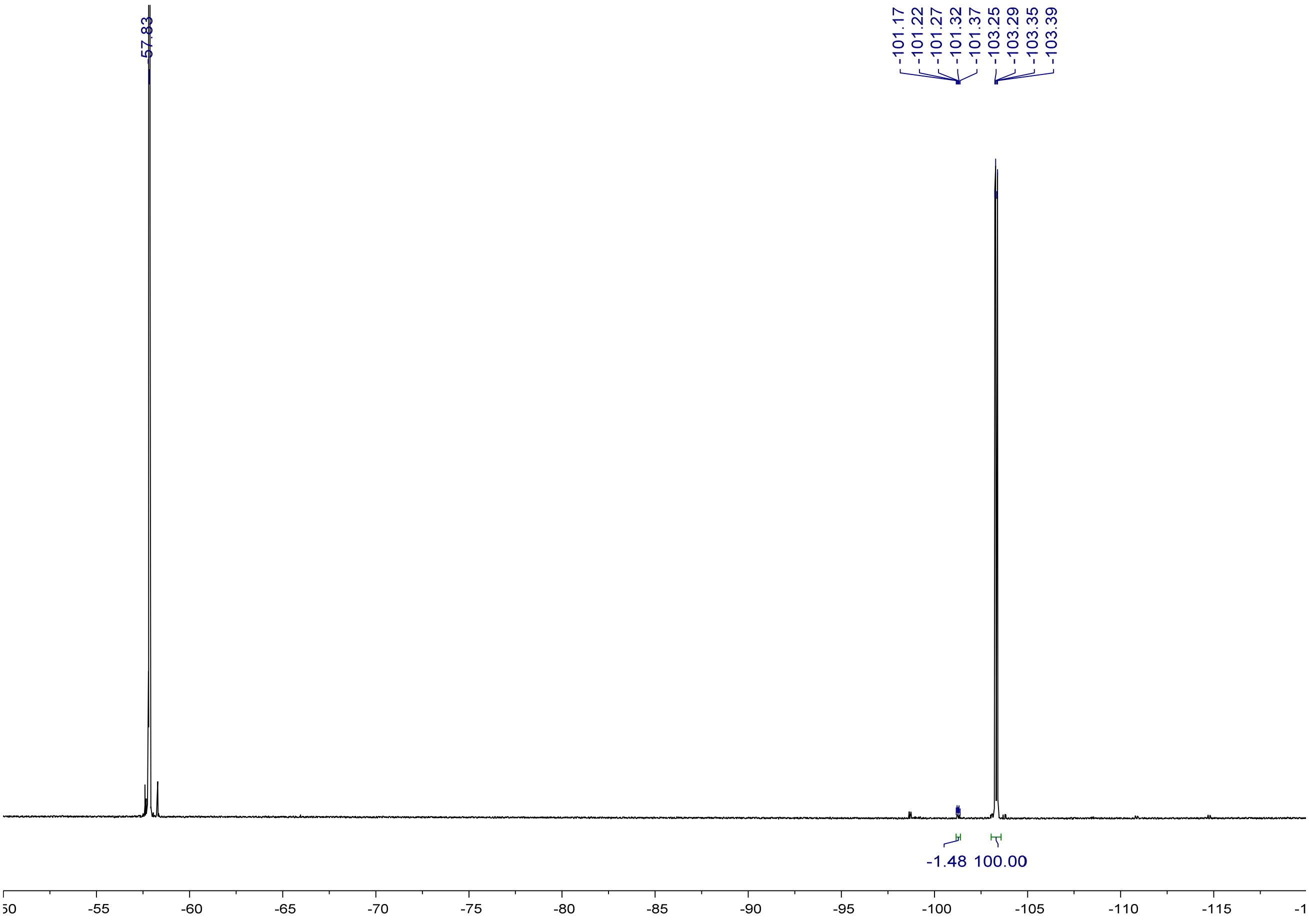

3l: ^{19}F NMR analysis

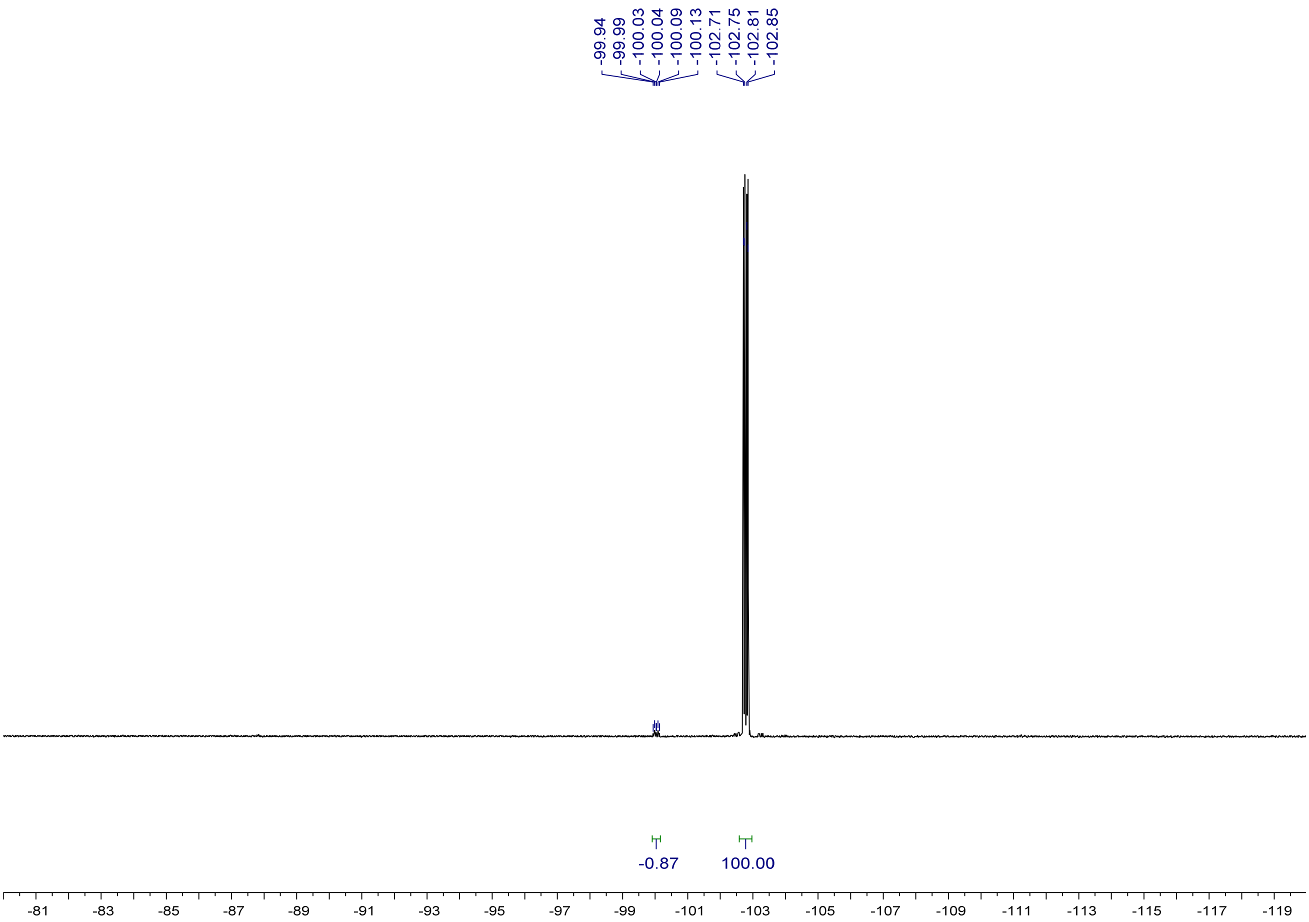

3m: ^{19}F NMR analysis

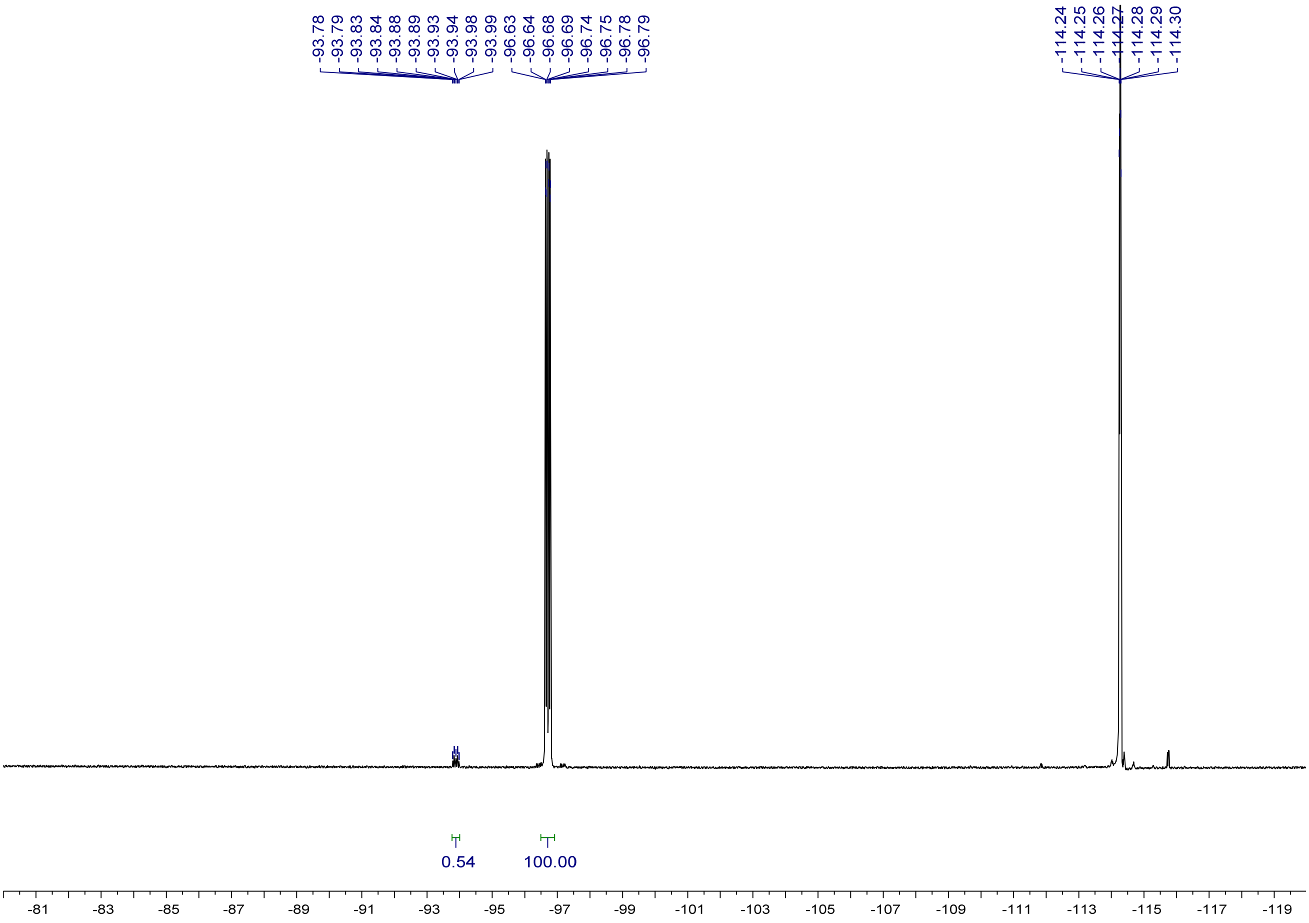

3n: ^{19}F NMR analysis

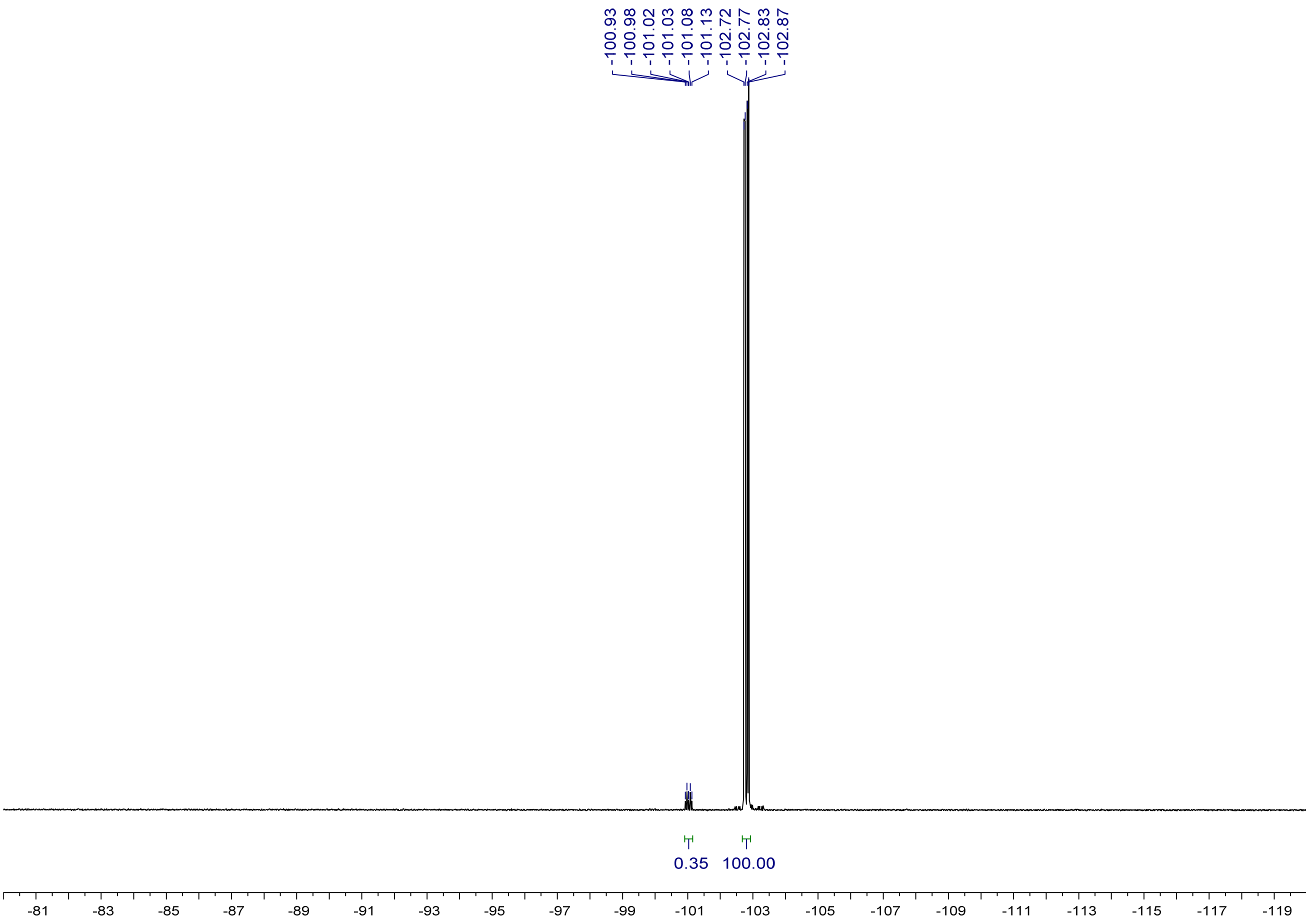

3o: ^{19}F NMR analysis

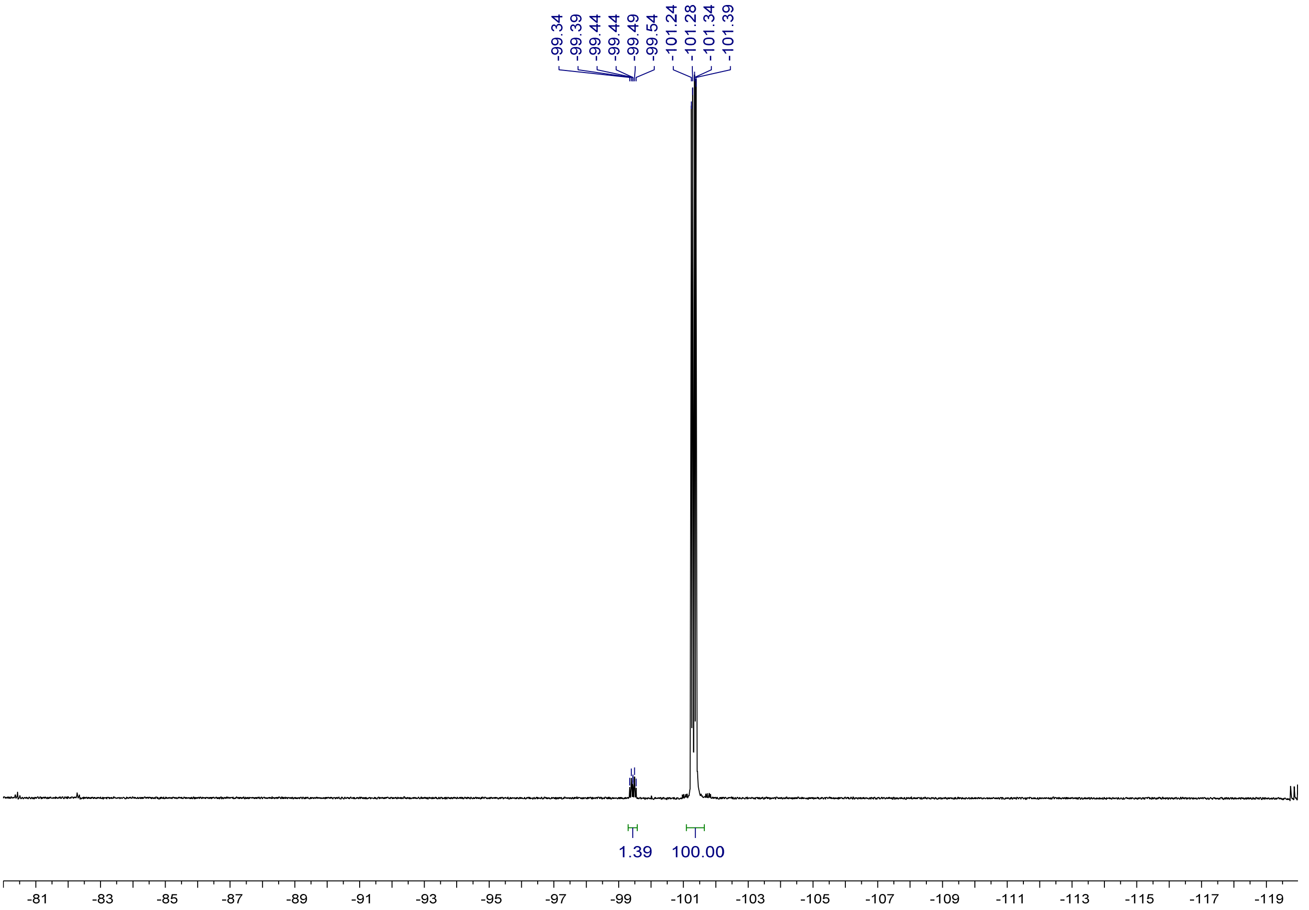

3p: ^{19}F NMR analysis

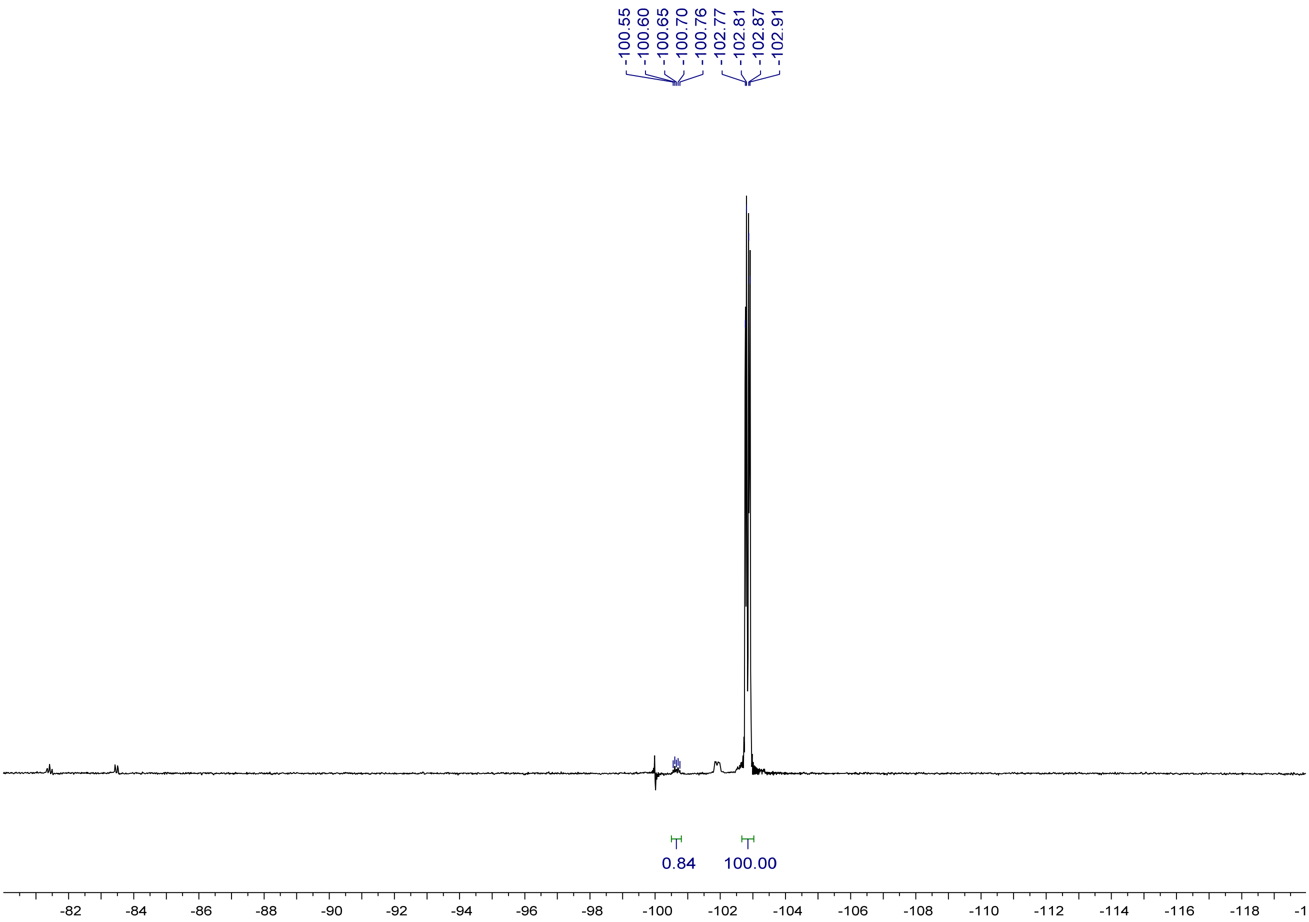

3q: ^{19}F NMR analysis

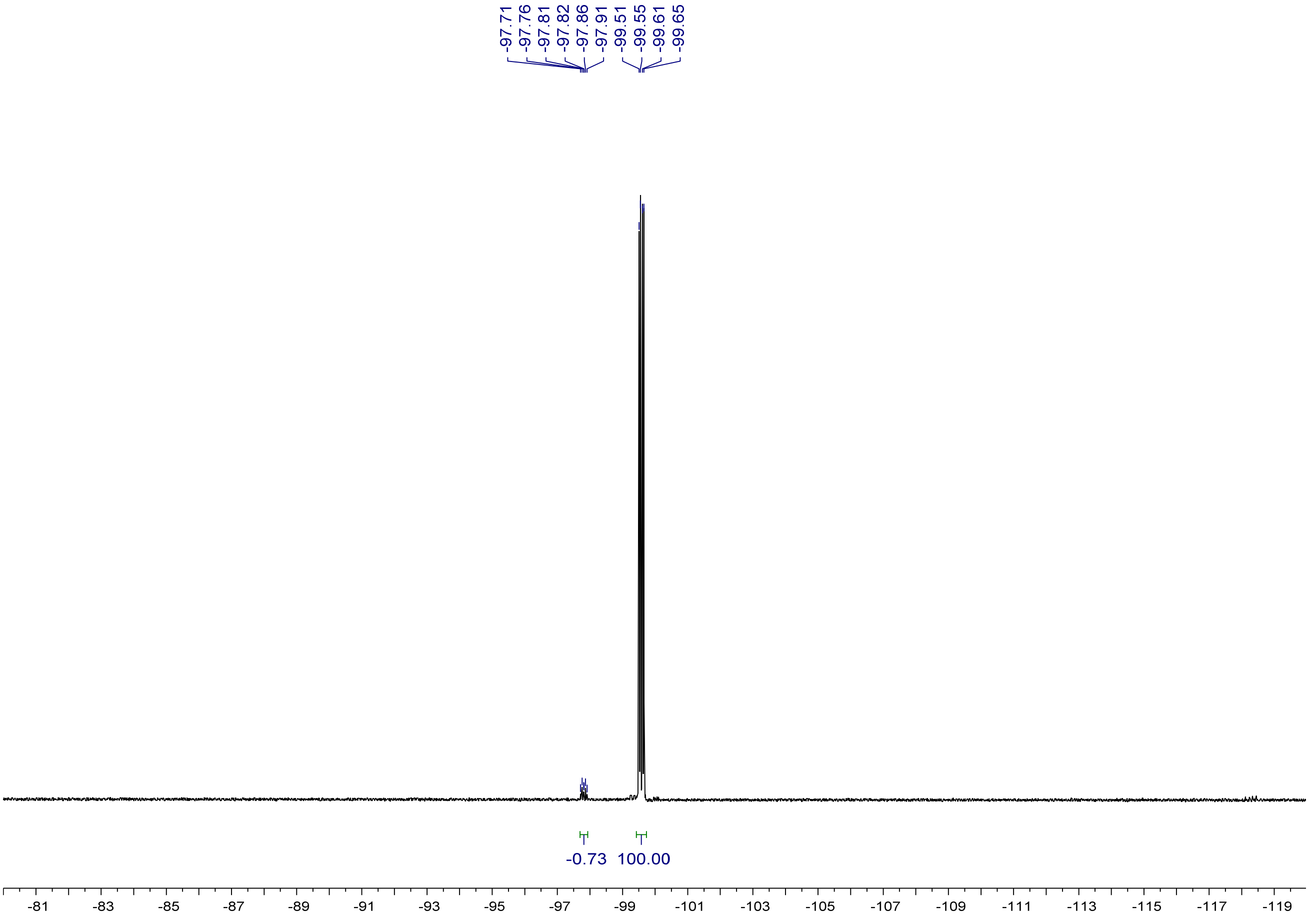

3r: ^{19}F NMR analysis

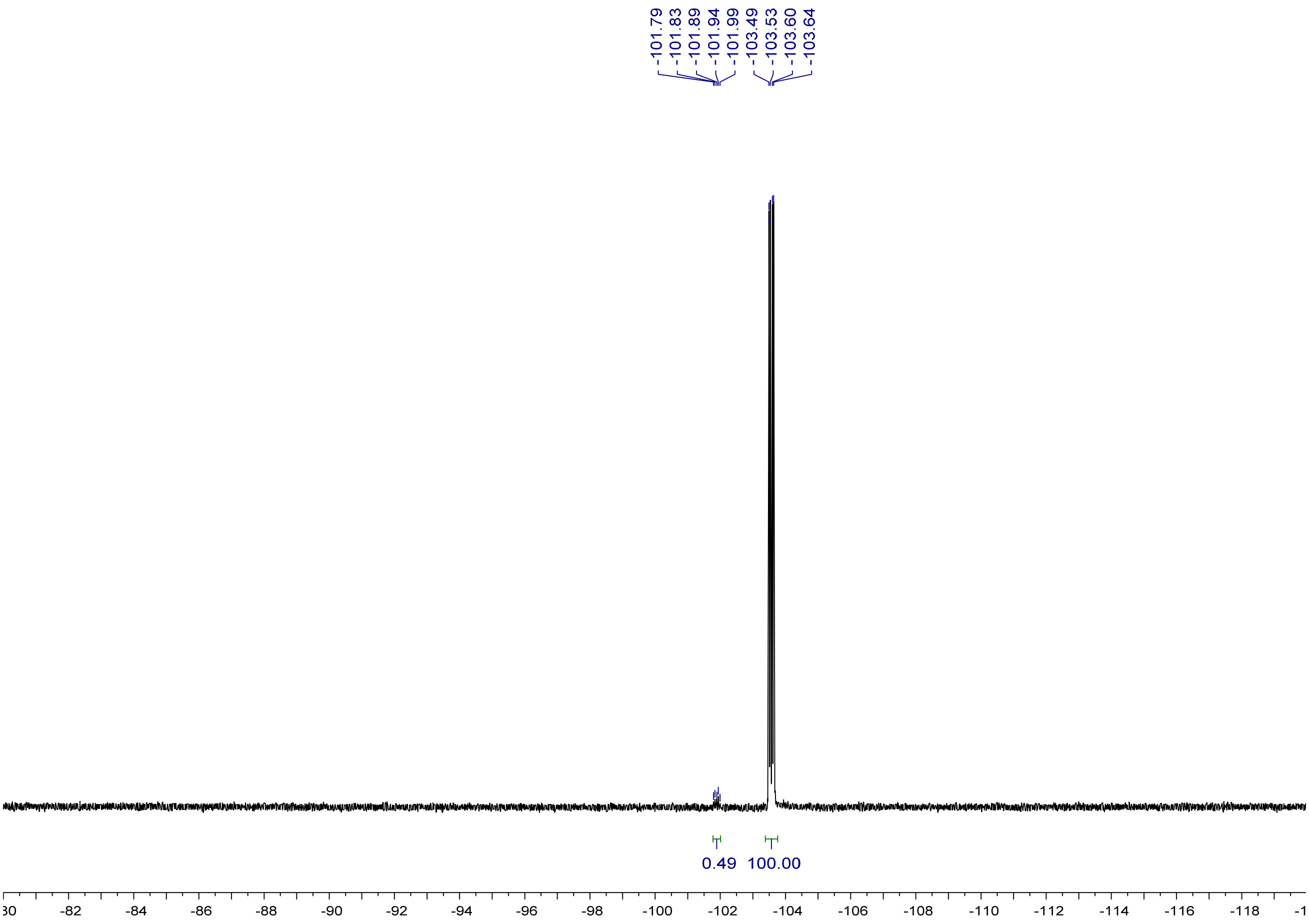

3s: ^{19}F NMR analysis

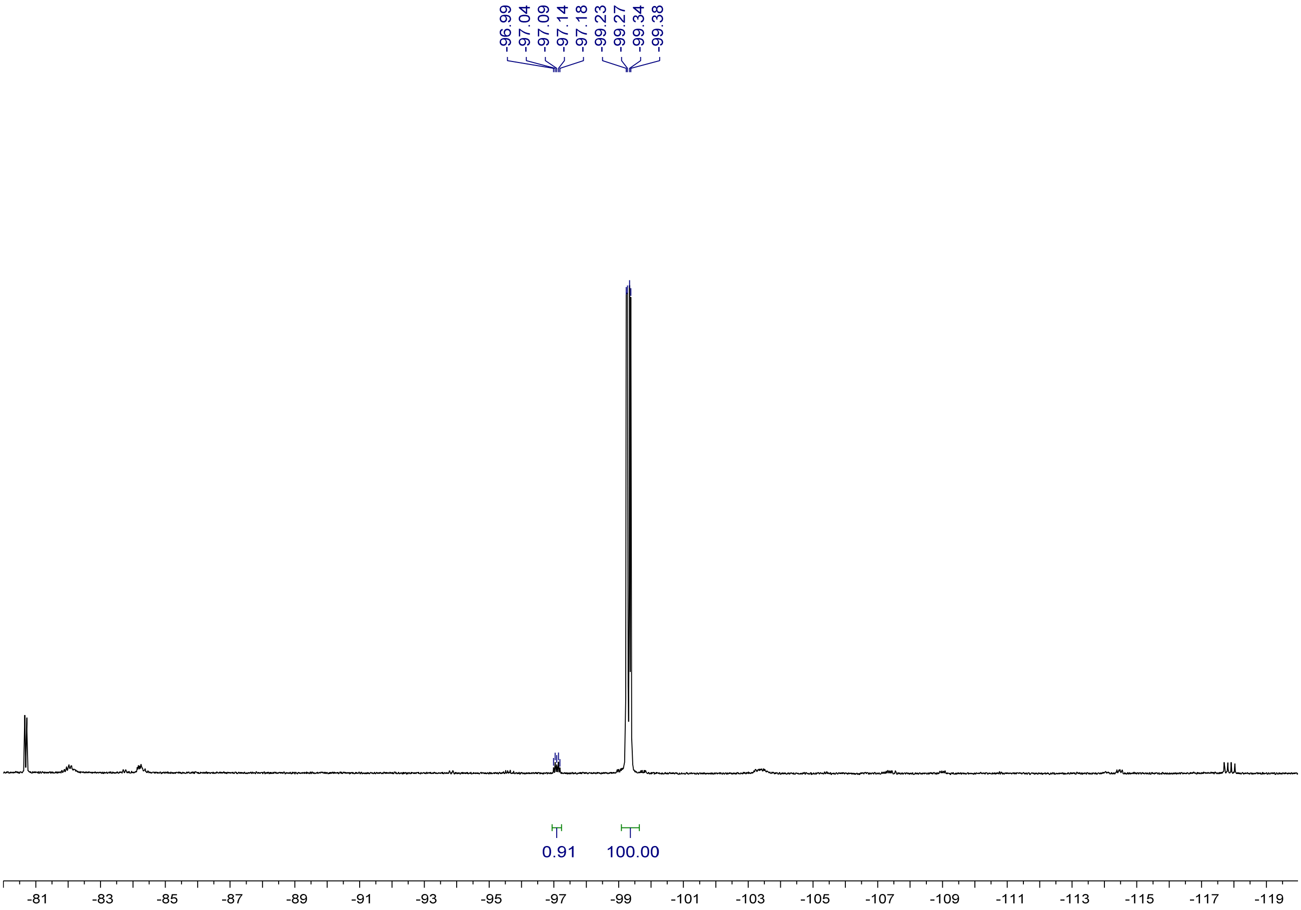

3t: ^{19}F NMR analysis

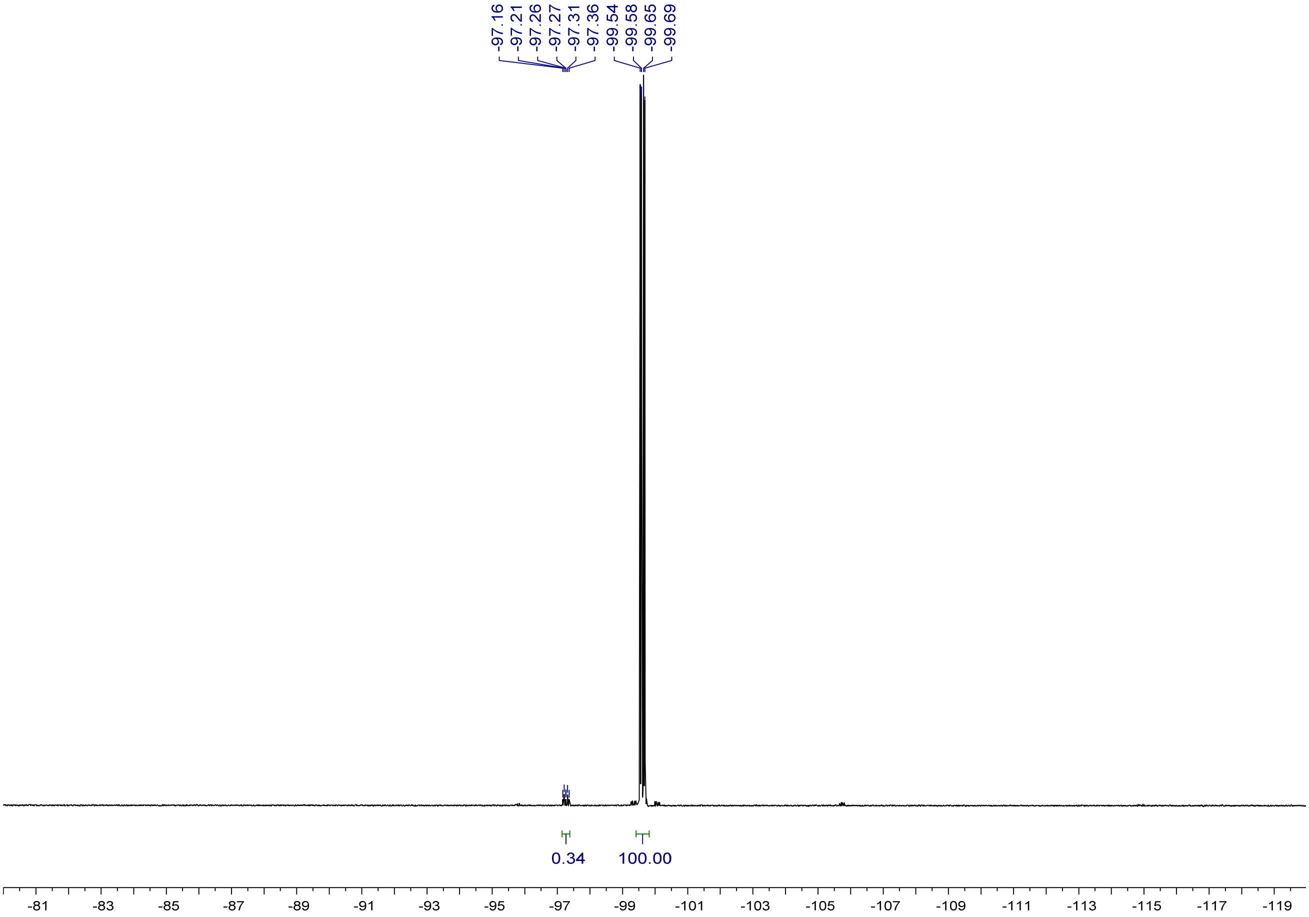

3u: ^{19}F NMR analysis

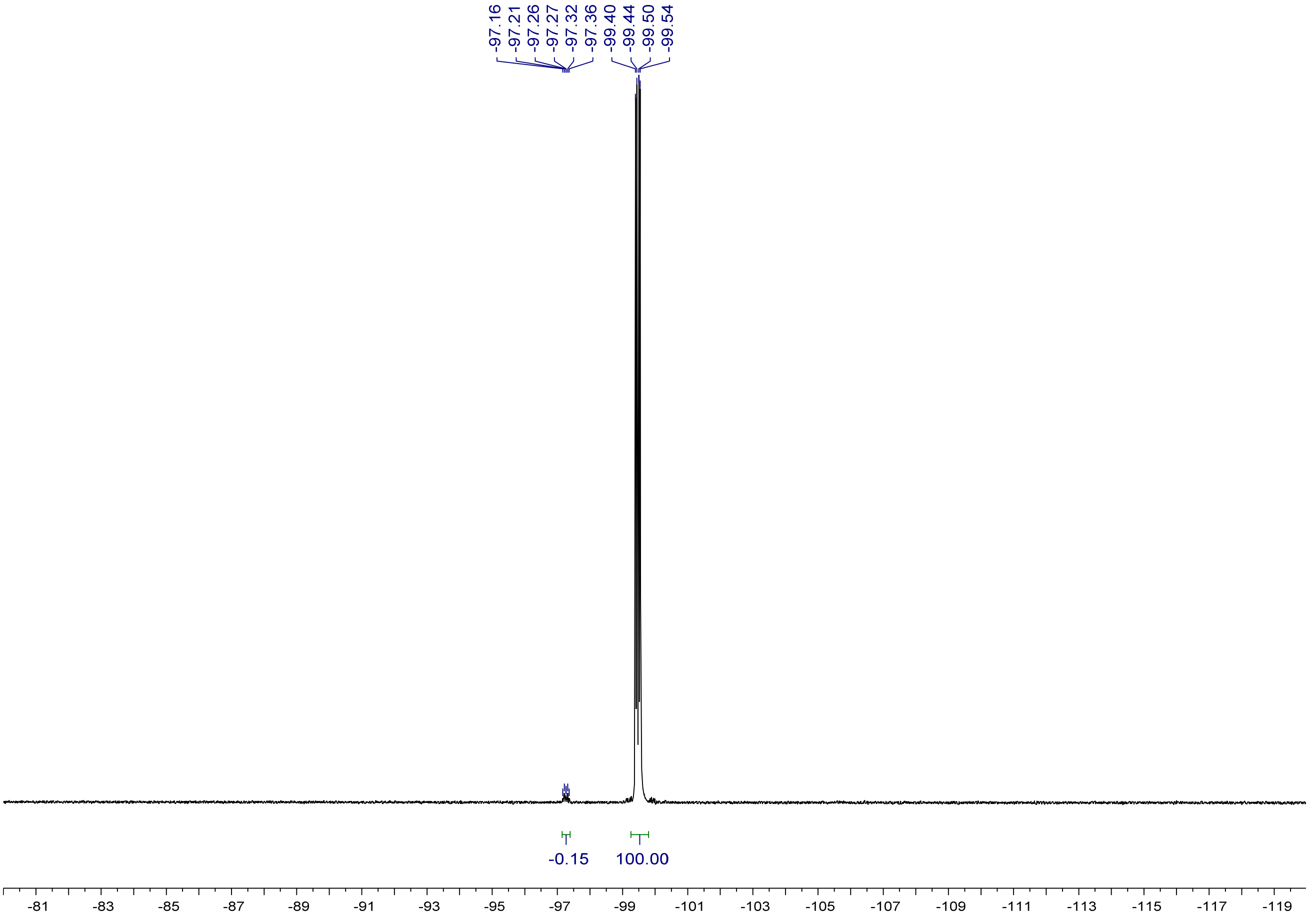

3v: ^{19}F NMR analysis

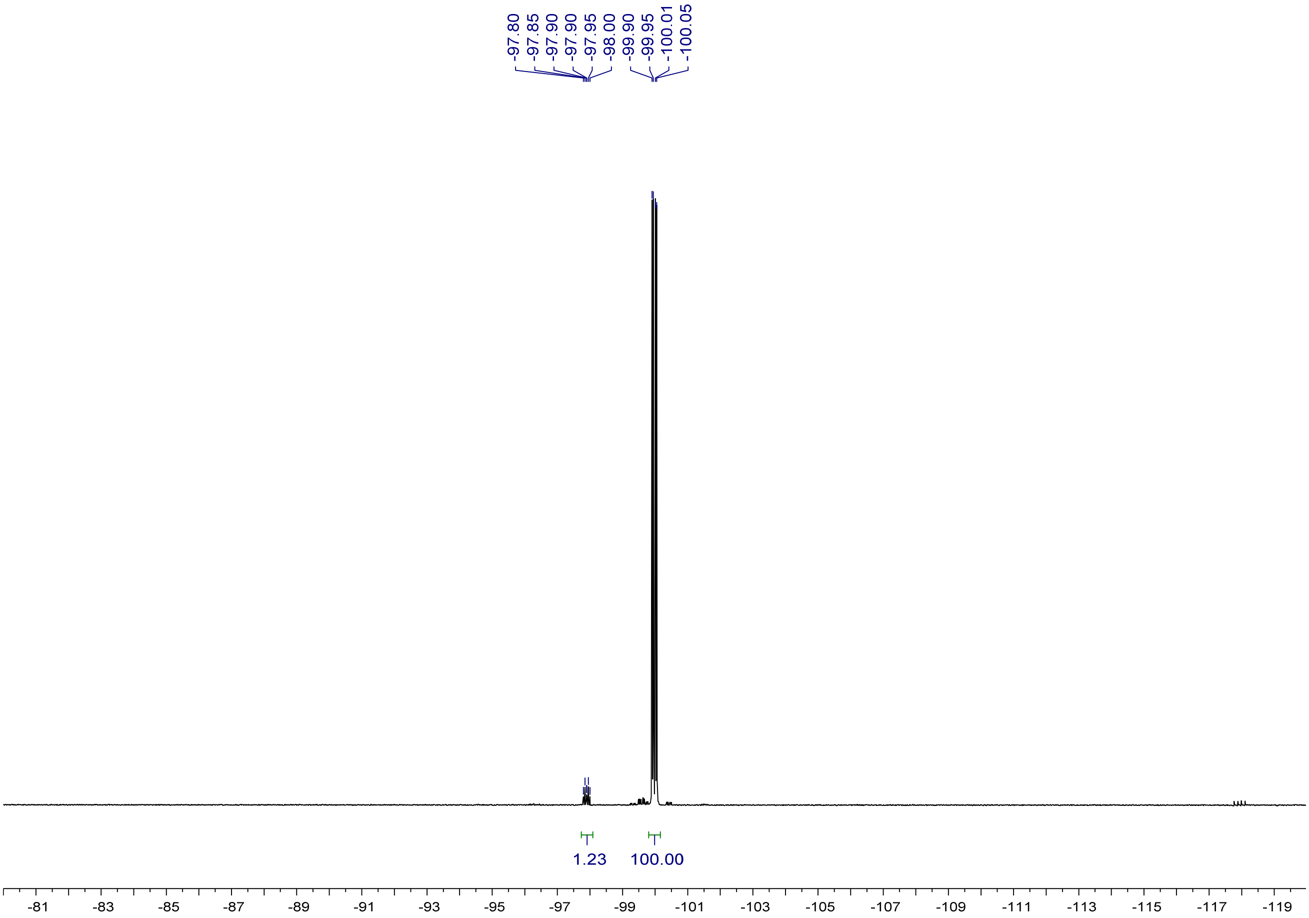

3w: ^{19}F NMR analysis

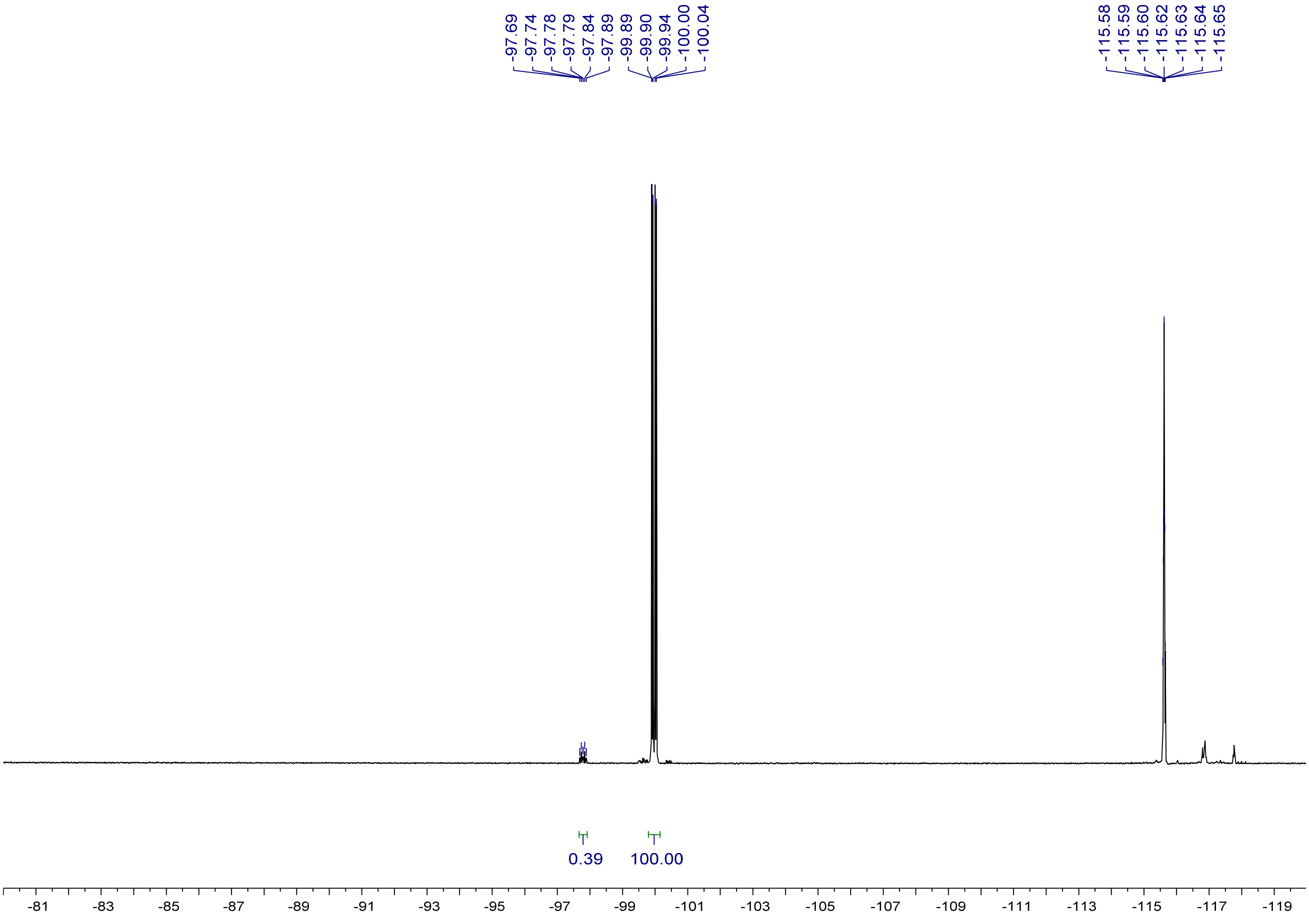

3x: ^{19}F NMR analysis

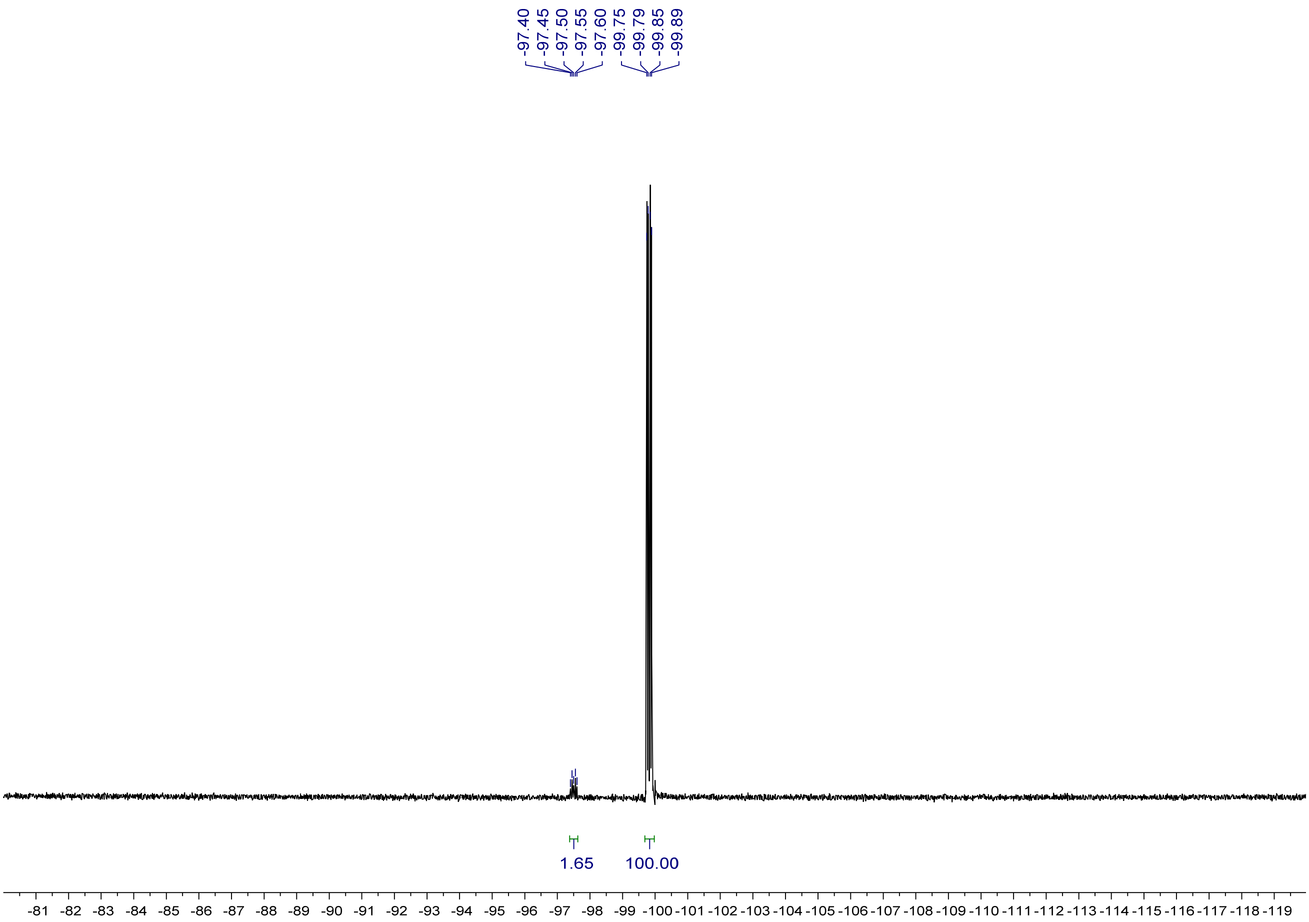

3y: ^{19}F NMR analysis

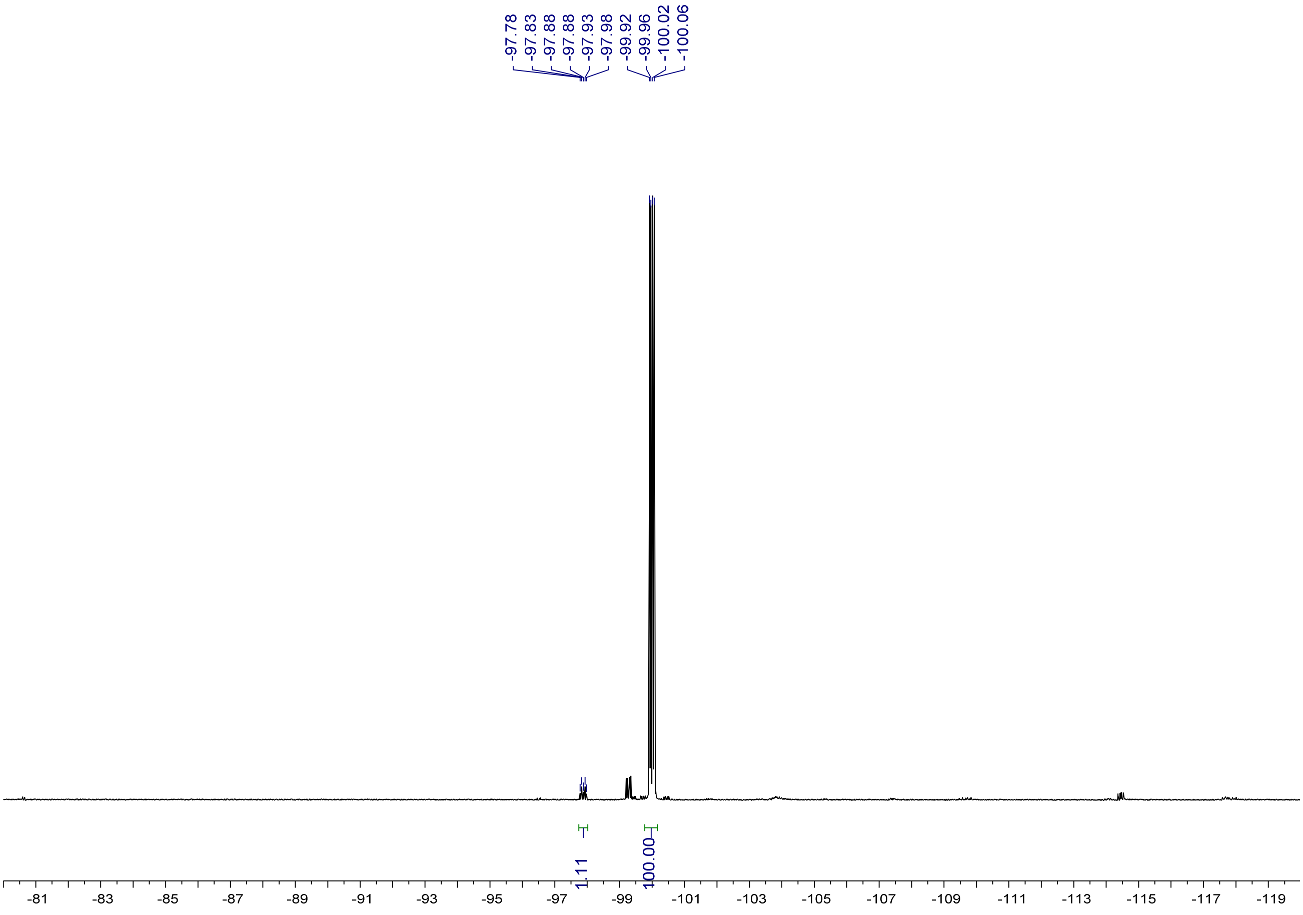

3z: ^{19}F NMR analysis

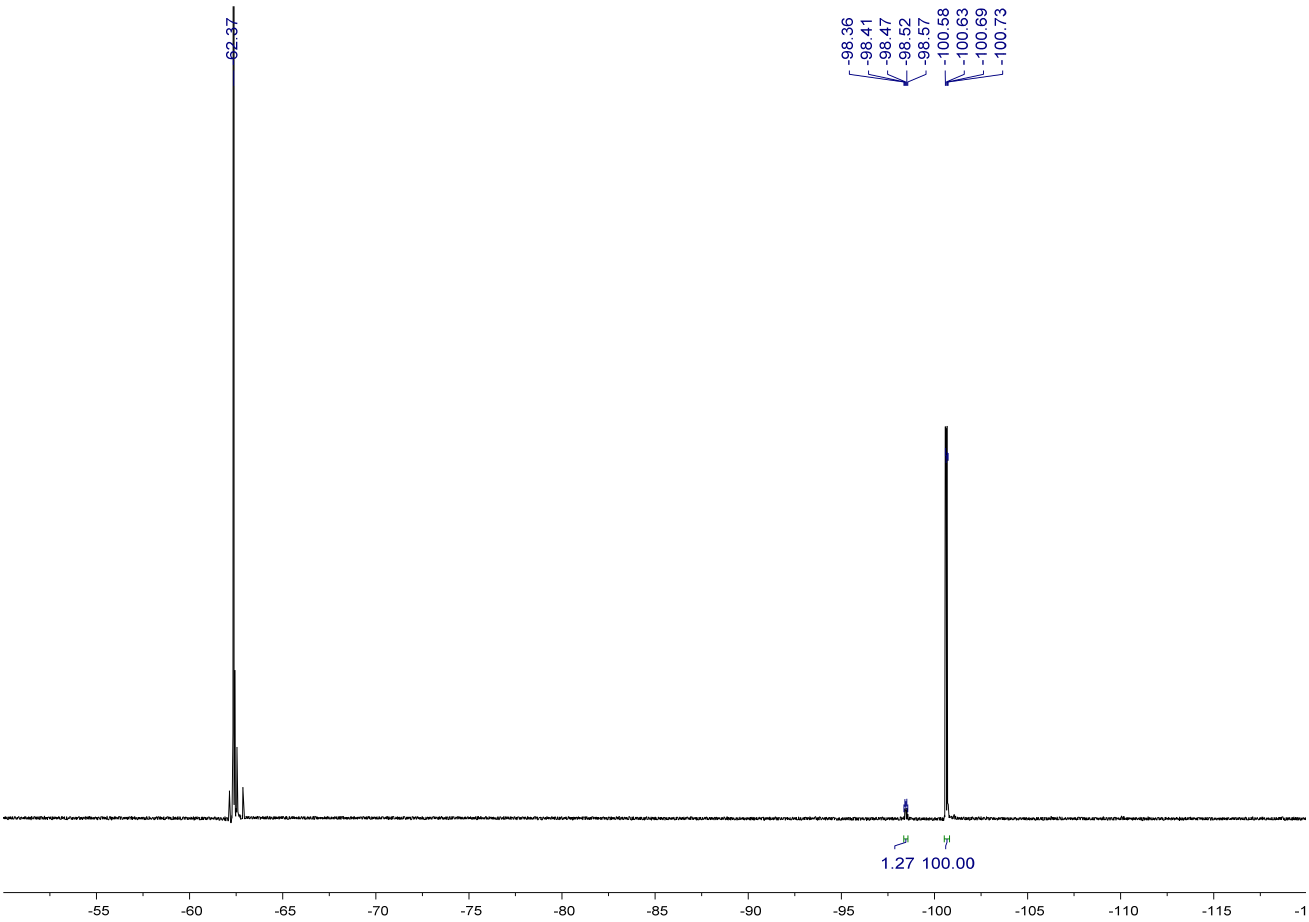

3aa: ^{19}F NMR analysis

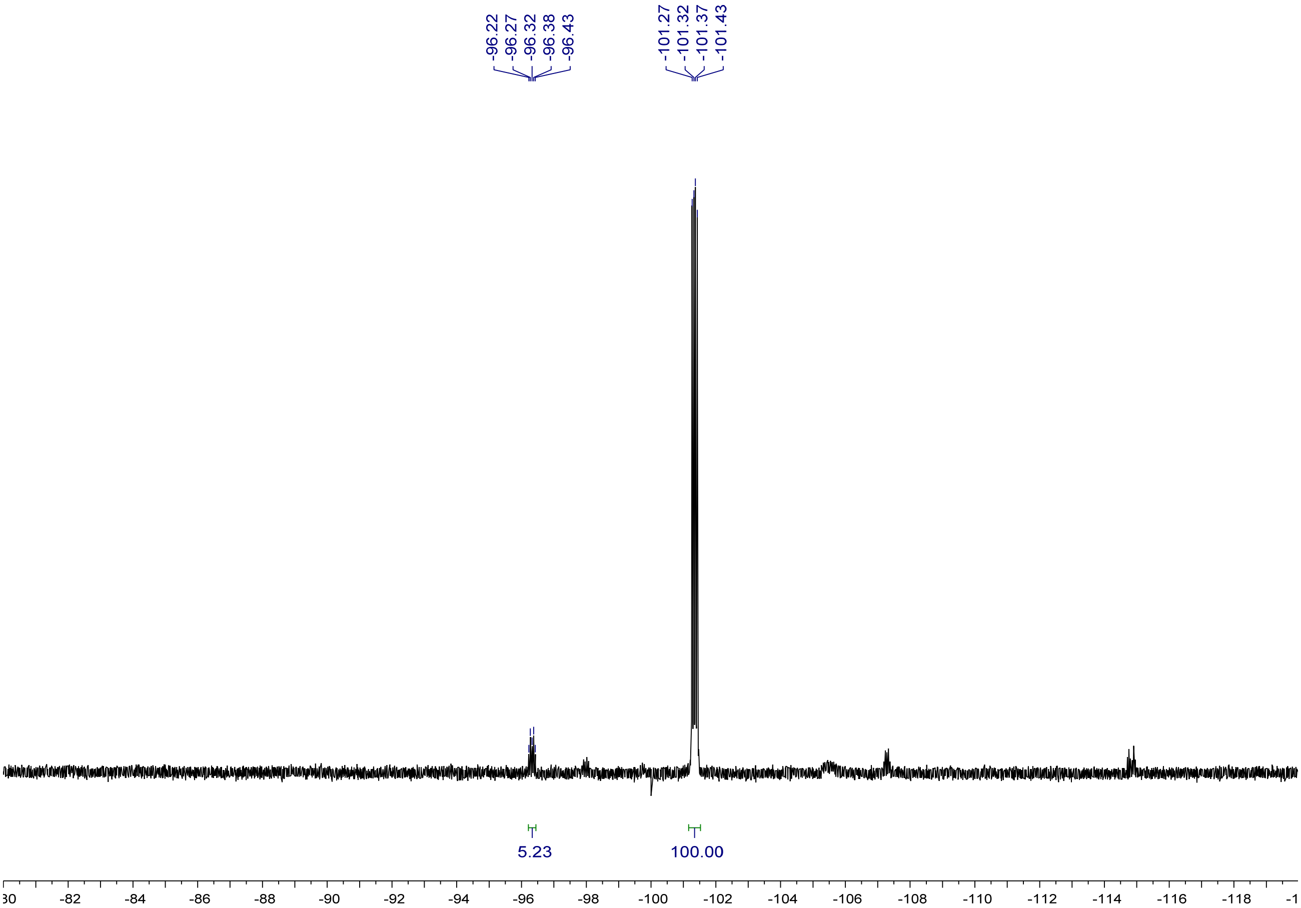

3ab: ^{19}F NMR analysis

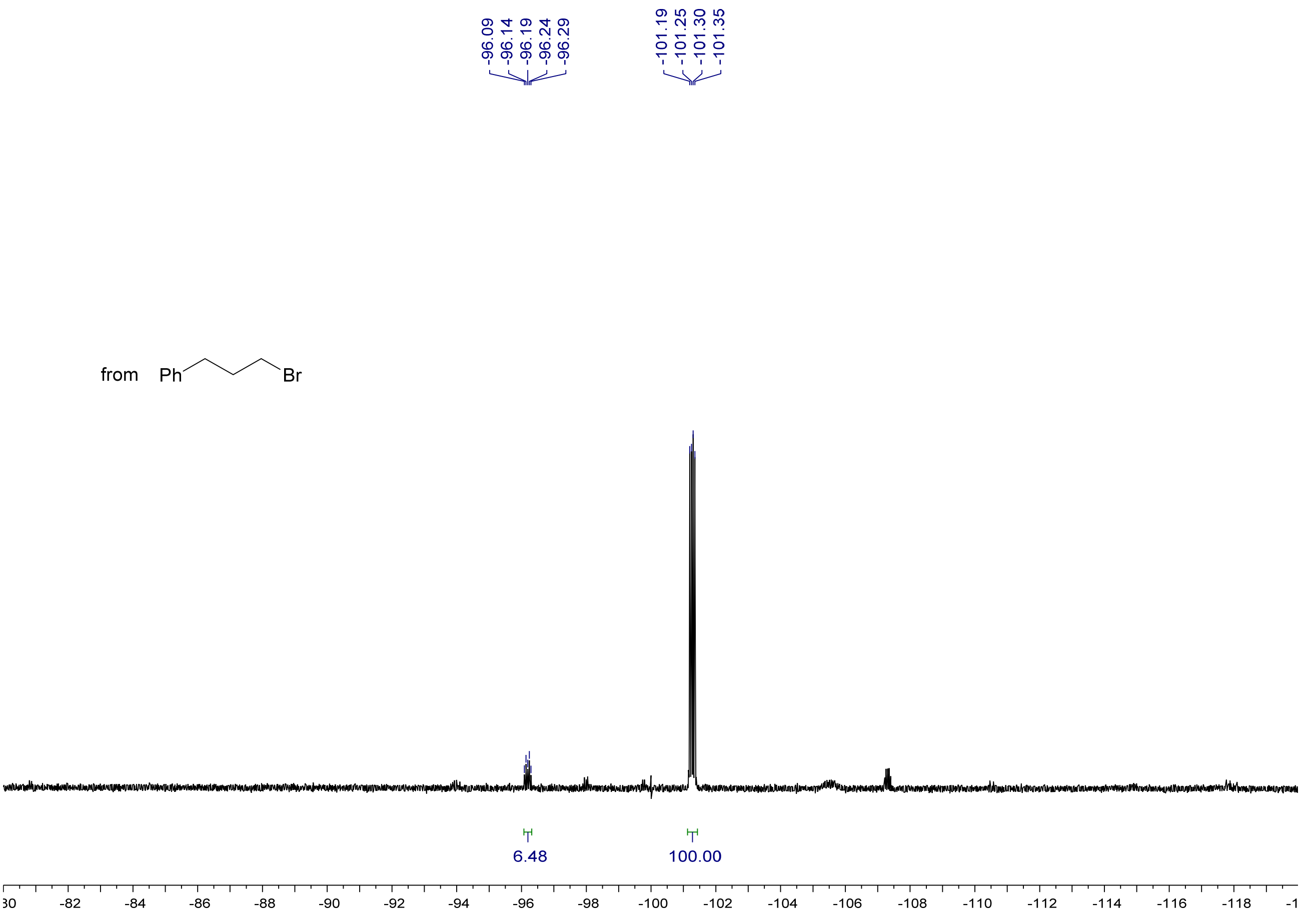

3ac: ^{19}F NMR analysis

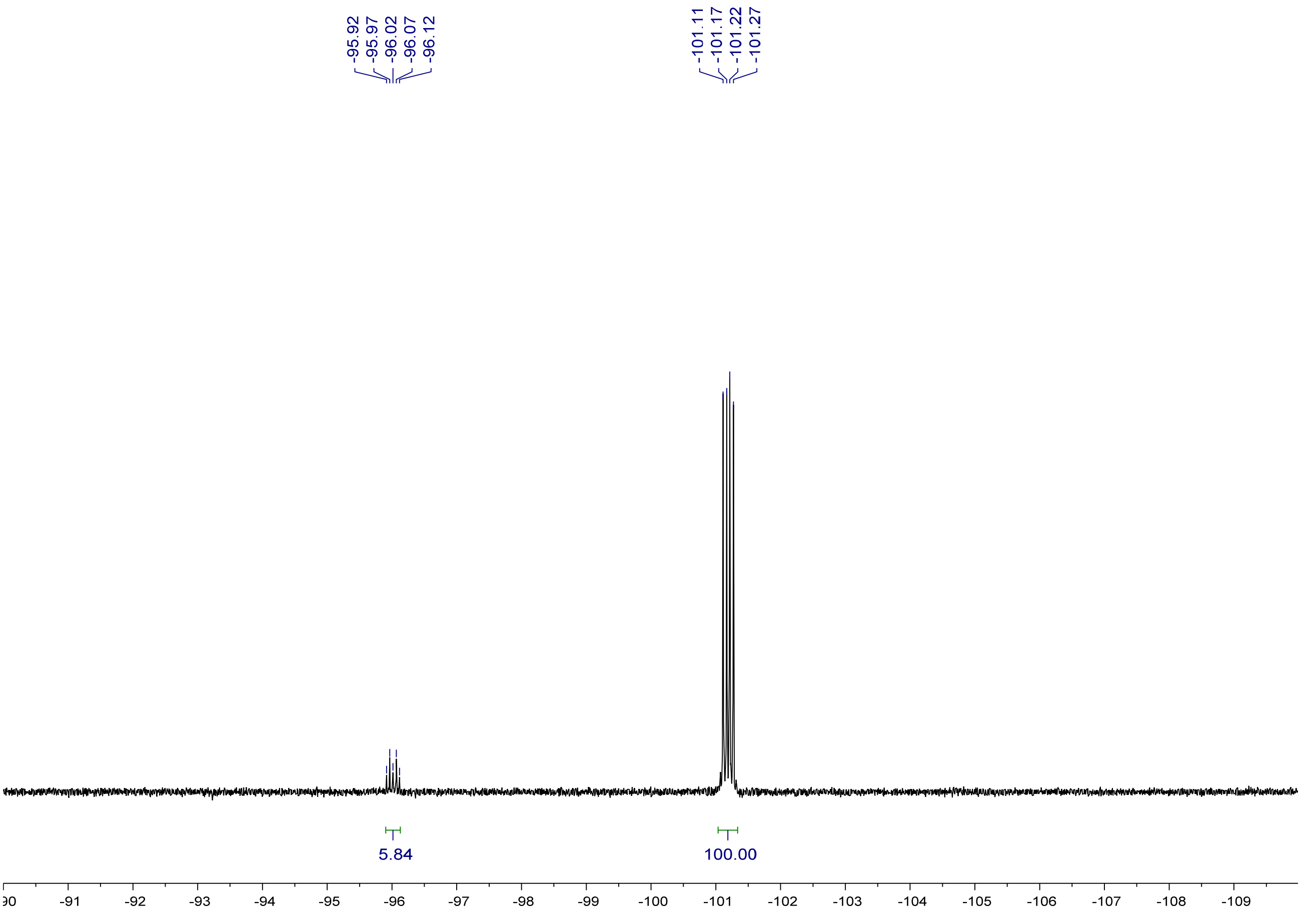

3ab: ^{19}F NMR analysis

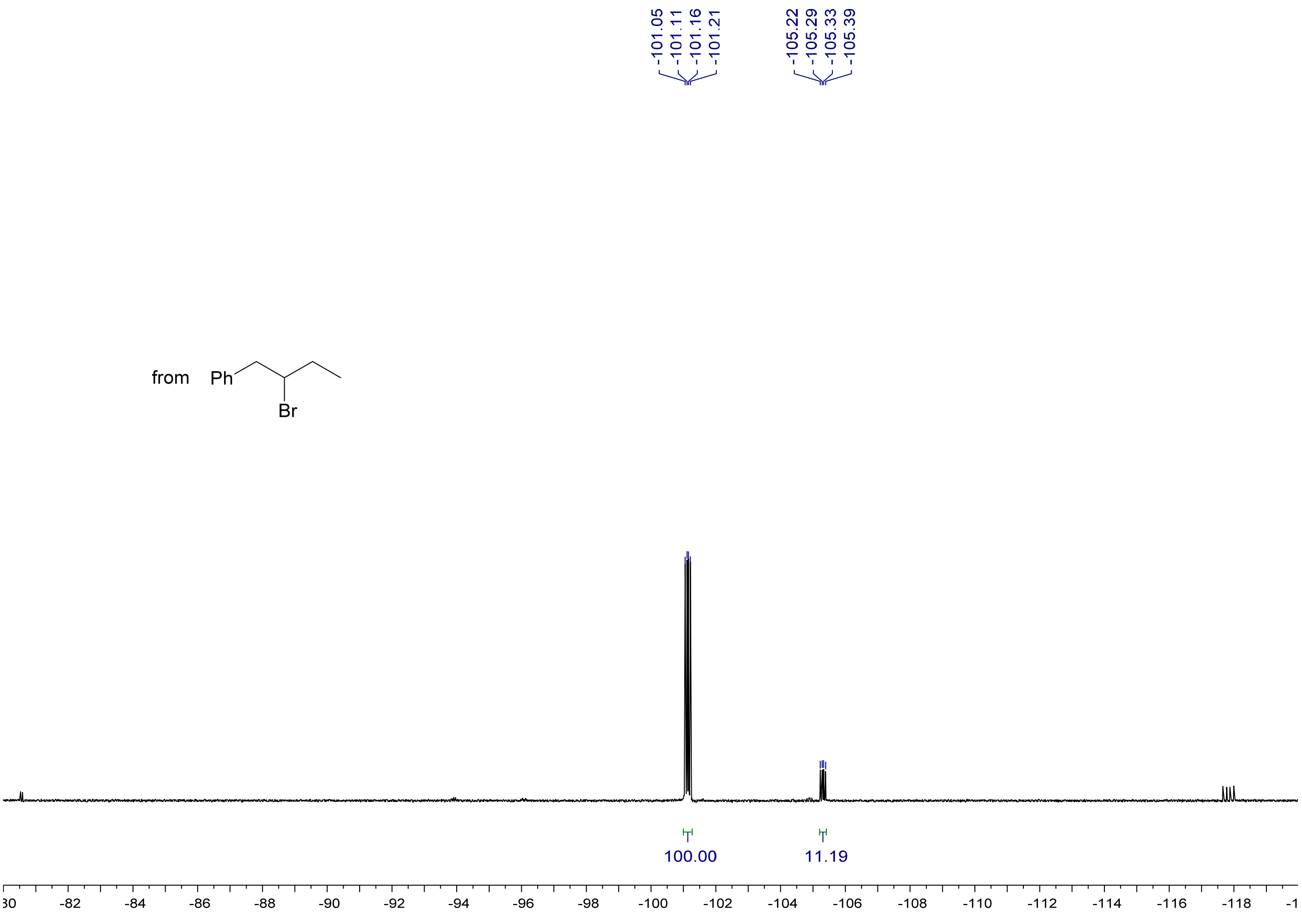

3ab: ^{19}F NMR analysis

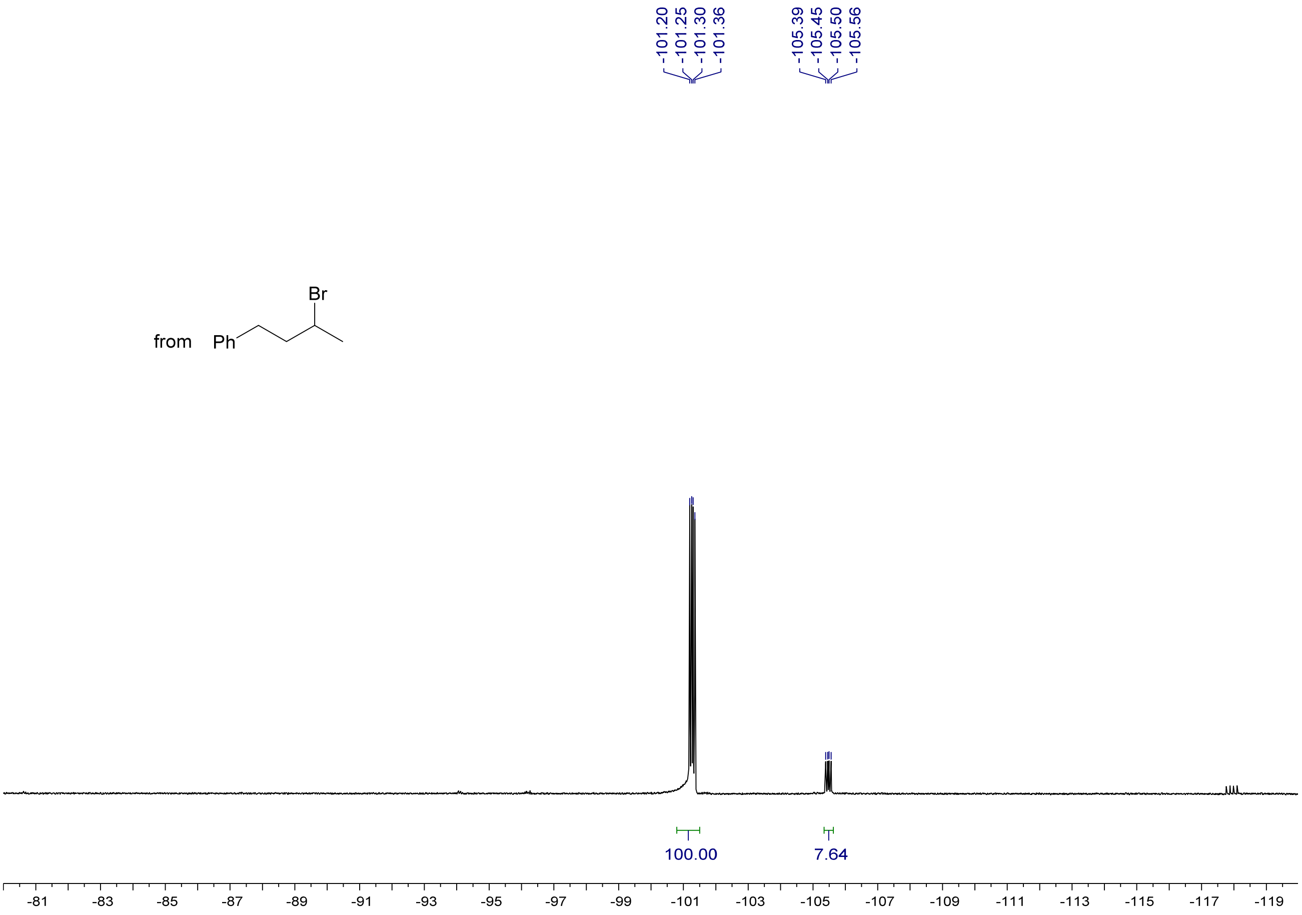

Supplement: Supplementary file 1 [file SC-010-C8SC04162H-s001.pdf]
